# Scaling up production of recombinant human basic fibroblast growth factor in an *Escherichia coli* BL21(DE3) plysS strain and evaluation of its pro-wound healing efficacy

**DOI:** 10.3389/fphar.2023.1279516

**Published:** 2024-02-05

**Authors:** Le Li, Bingjie Yu, Yingji Lai, Siyuan Shen, Yawei Yan, Guojun Dong, Xiangyun Gao, Yanrong Cao, Caojie Ge, Liqin Zhu, Huan Liu, Shanhui Tao, Zhiang Yao, Shijun Li, Xiaojie Wang, Qi Hui

**Affiliations:** ^1^ School of Pharmacy, Wenzhou Medical University, Wenzhou, China; ^2^ Engineering Laboratory of Zhejiang Province for Pharmaceutical Development of Growth Factors, Biomedical Collaborative Innovation Center of Wenzhou, Wenzhou, China; ^3^ Alberta Institute, Wenzhou Medical University, Wenzhou, China; ^4^ Institute of Life Science, Wenzhou University, Wenzhou, China

**Keywords:** hbFGF, *Escherichia coli* BL21(DE3) plysS, optimized production, 500-L fermentation, purification, wound healing

## Abstract

**Introduction:** Human basic fibroblast growth factor (hbFGF) is a highly valuable multifunctional protein that plays a crucial role in various biological processes. In this study, we aim to accomplish the scaling-up production of mature hbFGF (146aa) by implementing a high cell-density fermentation and purification process on a 500-L scale, thereby satisfying the escalating demands for both experimental research and clinical applications.

**Methods:** The hbFGF DNA fragment was cloned into a mpET-3c vector containing a kanamycin resistance gene and then inserted into *Escherichia coli* BL21 (DE3) plysS strain. To optimize the yield of hbFGF protein, various fermentation parameters were systematically optimized using BOX-Behnken design and further validated in large-scale fermentation (500-L). Additionally, a three-step purification protocol involving CM-Sepharose, heparin affinity, and SP-Sepharose column chromatography was developed to separate and purify the hbFGF protein. Isoelectric focusing electrophoresis, MALDI-TOF/MS analysis, amino acid sequencing, CD spectroscopy, and Western blotting were performed to authenticate its identity. The biological efficacy of purified hbFGF was evaluated using an MTT assay as well as in a diabetic deep second-degree scald model.

**Results:** The engineered strain was successfully constructed, exhibiting high expression of hbFGF and excellent stability. Under the optimized fermentation conditions, an impressive bacterial yield of 46.8 ± 0.3 g/L culture with an expression level of hbFGF reaching 28.2% ± 0.2% was achieved in 500-L scale fermentation. Subsequently, during pilot-scale purification, the final yield of purified hbFGF protein was 114.6 ± 5.9 mg/L culture with RP-HPLC, SEC-HPLC, and SDS-PAGE purity exceeding 98%. The properties of purified hbFGF including its molecular weight, isoelectric point (pI), amino sequence, and secondary structure were found to be consistent with theoretical values. Furthermore, the purified hbFGF exhibited potent mitogenic activity with a specific value of 1.05 ± 0.94 × 106 AU/mg and significantly enhanced wound healing in a deep second-degree scald wound diabetic rat model.

**Conclusion:** This study successfully established a stable and efficient large-scale production process of hbFGF, providing a solid foundation for future industrial production.

## Introduction

Human basic fibroblast growth factor (hbFGF), also known as FGF-2, is a non-glycosylated single-chain protein involved in various biological processes (Powers et al., 2000; Barrientos et al., 2008; Beenken and Mohammadi, 2009; Korc and Friesel, 2009; Kuo et al., 2015). To date, hbFGF has been approved for skin wound repair in China and periodontitis, pressure sores, and skin ulcers in Japan (Hui et al., 2018a). In addition, accumulating evidence suggests that hbFGF is a potential therapeutic agent for several diseases, such as heart repair (Li et al., 2021), nerve injury (Li et al., 2017), bone regeneration (Novais et al., 2021), and asthma and chronic obstructive pulmonary disease (Tan et al., 2020), and a potential predictive biomarker for hematological and solid tumors (Akl et al., 2016). Due to its high clinical therapeutic value, large-scale preparation of hbFGF with high purity, activity, and yield is needed.

Human bFGF exhibits isoforms with high molecular weight (HMW) and low molecular weight (LMW). The HMW hbFGF isoforms, comprising 22 kDa (196 aa), 22.5 kDa (201 aa), 24 kDa (210 aa), and 34 kDa (288 aa) forms, are generated through four atypical CUG start sites (Florkiewicz and Sommer, 1989; Prats et al., 1989; Florkiewicz et al., 1991). These isoforms localize to the nucleus and function independently of FGF receptors (FGFRs) (Yu et al., 2007 **)**. Conversely, the LMW hbFGF isoform, known as hbFGF_155_, has a molecular weight of 18 kDa and comprises 155 amino acids. It is produced via typical AUG start sites (Ibrahimi et al., 2004a; Sørensen et al., 2006). In addition, the mature hbFGF (hbFGF_146_) is a 16.5 kDa protein consisting of 146 amino acids and derived from the LMW hbFGF through truncation of a 9 aa N-terminal prosegment (Okada-Ban et al., 2000). Typically, hbFGF_146_ or hbFGF_155_ are secreted directly into the extracellular environment and function by activating FGFR signaling, which holds significant clinical implications (Ibrahimi et al., 2004b).

In recent decades, successful expression of hbFGF_146_ or hbFGF_155_ has been achieved in various hosts, including *E. coli* (Feng et al., 2004; Chen et al., 2012; Rassouli et al., 2013; Soleyman et al., 2016), *Bacillus subtilis* (Kwong et al., 2013; Hu et al., 2018), yeast (Le et al., 2020), silkworm (Masuda et al., 2018), and plants (Ding et al., 2006; Yang et al., 2018). Of these, *E. coli* is an exemplary choice for the industrial-scale production of hbFGF due to its time-saving and cost-effective nature. Furthermore, various fusion strategies have been employed to enhance the soluble expression of hbFGF, including the utilization of glutathione S-transferase (GST) (Sheng et al., 2003; Sekiguchi et al., 2018), thioredoxin (Trx) (Imsoonthornruksa et al., 2015; Soleyman et al., 2016), maltose-binding protein (MBP) (Lemaitre et al., 1995), and collagen-like protein (Scl2) (Rahman et al., 2020). Nevertheless, challenges such as insufficient fusion tag cleavage, high costs associated with protease usage, and prolonged processing times have hindered the implementation of these strategies in large-scale production settings. However, it has been reported that while hbFGF_155_ can be produced at a fermentation scale ranging from 40 to 150 L, hbFGF_146_ is still limited to shake flask level production. Additionally, the commercial availability of hbFGF is accompanied by significantly high prices ranging from US$1,300 to US$2000 per mg. Therefore, in this study, to meet the increasing demands for both experimental research and clinical applications, large-scale production of mature hbFGF (146 aa) was conducted by implementing a high cell-density fermentation and purification process on a 500-L scale. Subsequently, the characteristics and biological activity of the purified hbFGF expressed in 500-L scale fermentation were determined *in vitro*, as well as its pro-wound healing effects on a deep second-degree scald wound diabetic rat model *in vivo*.

## Materials and methods

### Materials

Yeast powder and tryptone were purchased from Oxoid Co., Ltd. (Hampshire, England). The modified pET-3c plasmid (mpET-3c) containing a kanamycin resistance gene, *E. coli* BL21 (DE3) plysS (Catalog No. CD701), and the NIH-3T3 cell line was provided by BGI, Transgen Biotechnology Co., Ltd. (Beijing, China) and ATCC. Restriction enzymes, gel extraction kit, PCR purification kit, and plasmid micro-preparation kit were obtained from Dalian Takara Corporation. Kanamycin sulfate and isopropyl-β-D-thiogalactoside (IPTG) were purchased from Beijing Dingguo Changsheng Biotechnology Co., Ltd., and Dalian Meilunbio Co., Ltd., respectively. The 200-L (Model BIOTECH-200JS) and 500-L (Model Biostat D500) fermentor were acquired from Shanghai Baoxing Bio-Engineering Equipment Co., Ltd., and B. Braun (Germany), respectively. CM-Sepharose, heparin-Sepharose CL-6B, and SP-Sepharose columns were supplied by GE Healthcare (United States). The polyclonal rabbit anti-human bFGF antibody (Cat.No: 3196S) was acquired from Cell Signaling Technology (United States), while the secondary antibody (goat anti-rabbit lgG/HRP antibody, Cat.No: HS101-01) was procured from Transgene Biotech (Beijing, China). Additionally, the human bFGF standard was sourced from the National Institutes for Food and Drug Control.

### Construction and identification of the mpET-3c/hbFGF expression vector

**FIGURE 1:**
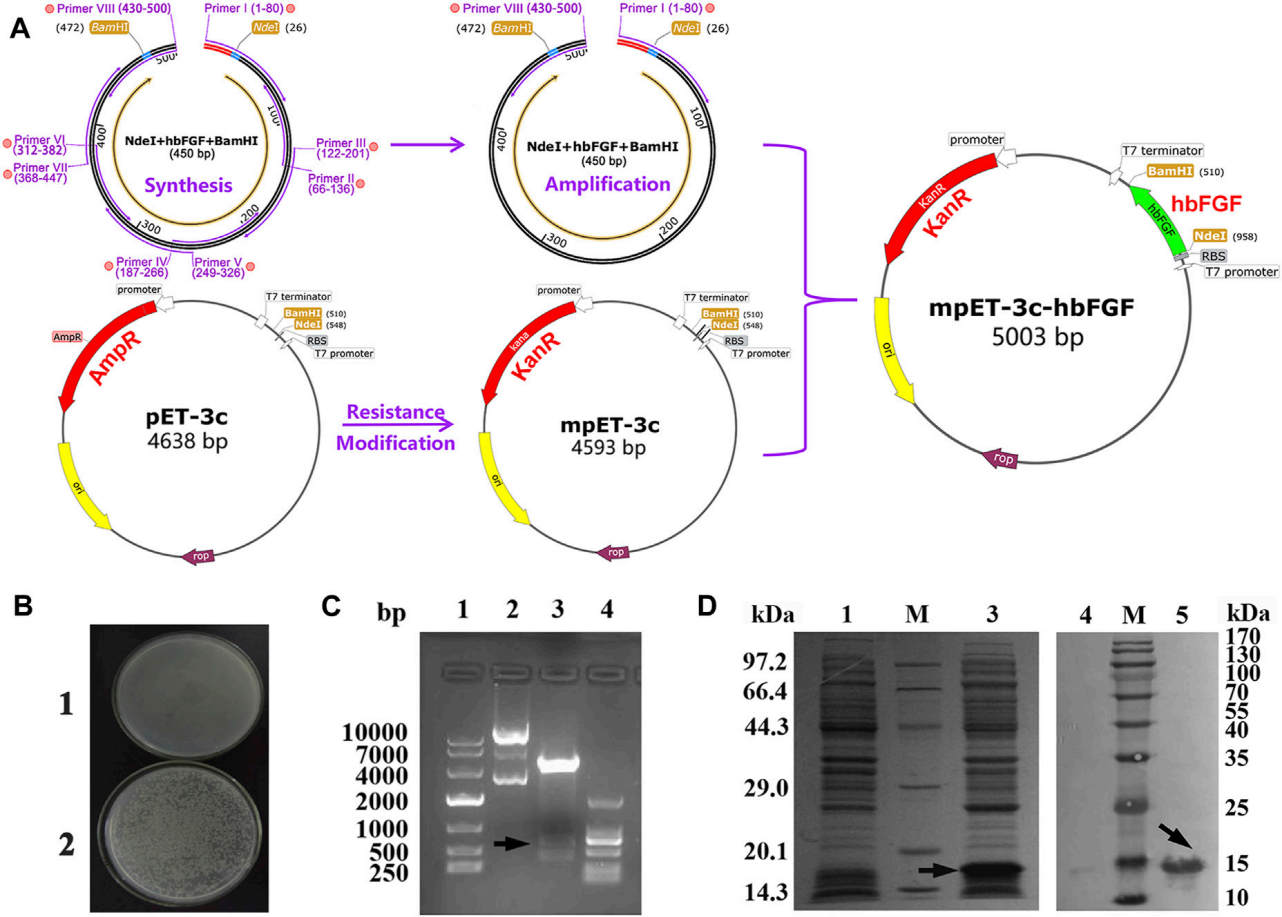
Recombinant plasmid construction and expression of the hbFGF protein. **(A)** Schematic diagram of the mpET3c-hbFGF recombinant plasmid construction. **(B)** Kanamycin resistance detection of the mpET3c-hbFGF recombinant plasmid. 1, *E. coli* BL21 (DE3) plysS-pET3c strain. 2, *E. coli* BL21 (DE3) plysS-mpET3c/hbFGF engineered strain. **(C)** Restriction enzyme analysis of the mpET3c-hbFGF recombinant plasmid. Lanes 1 and 4, DNA molecular weight marker. Lane 2, plasmid before digestion. Lane 3, plasmid after digestion. **(D)** SDS-PAGE (left) and Western blot (right) analyses of the expressed hbFGF protein in *E. coli* BL21(DE3) plysS. Lanes 1 and 4, non-induced. Lanes 3 and 5, induced by IPTG for 4 h. Lane M, molecular weight marker. Black arrows indicate hbFGF.

According to the human bFGF cDNA sequence retrieved from the NCBI database (GenBank accession number NM_002006.5), a refined gene construct of 438 bp encoding hbFGF (GenBank accession number OQ447501) was generated through overlap PCR and subsequently amplified using standard PCR techniques (Supplementary Tables S1, S2). The resulting amplicons were then subjected to digestion with *Nde* I and *Bam*H I restriction enzymes at 37°C for 4 h, followed by subcloning into the mpET-3c plasmid, thereby generating the recombinant plasmid mpET-3c/hbFGF (Figure 1A). The authenticity of this construct was confirmed through restriction enzyme analysis and sequencing conducted by Tsingke Biotechnology Co., Ltd. Subsequently, the mpET-3c/hbFGF plasmid was transformed into the *E. coli* BL21 (DE3) plysS strain, and positive clones were selected via kanamycin resistance screening at a concentration of 300 μg/mL.

### Screening and evaluation of the engineered expression strain

The positive colonies were inoculated into 5 mL of LB sterile medium containing 100 μg/mL kanamycin sulfate and then cultured at 37 °C with shaking at 200 rpm. Once the OD_600_ reached 0.8–1.2, IPTG was added to a final concentration of 1 mM and the culture was incubated for an additional 4 h at 37°C and 200 rpm. Subsequently, the expression level of hbFGF was evaluated using 12% SDS-PAGE with Coomassie Blue G-25 staining and densitometry analysis with Image Lab software 6.0. Furthermore, Western blotting analysis was employed for hbFGF identification. After a comprehensive evaluation of colony morphology, kanamycin resistance, Gram staining, electron microscopy, and gene sequencing, the colony exhibiting the highest expression level of hbFGF was chosen as the seed strain for subsequent studies on optimizing fermentation parameters.

### Optimization of hbFGF fermentation parameters on a flask scale

The optimization process was performed at shake flask level (250 mL). The seed strain was added to 30 mL of LB medium containing 100 μg/mL kanamycin sulfate and cultivated overnight at 37°C and 150 rpm. Subsequently, the seed culture was inoculated into fresh LB medium (30 mL) at a ratio of 1:100 (v/v) and incubated at 37°C and 200 rpm. The culture conditions and induction conditions were then optimized as follows:

#### Optimization of culture conditions on a flask scale

All experiments were conducted in triplicate, and the corresponding levels of factors were presented in Table 1. Growth curves were generated for the hbFGF-engineered strain under different temperatures, pH values, glucose concentrations, and dissolved oxygen. Furthermore, induction of the culture with IPTG (1.0 mM) occurred at different OD_600_ values followed by a 4-h incubation period at 37°C and 200 rpm.

**TABLE 1:** Tested cultural conditions for hbFGF production on a flask scale.

	Factors	Level					
		1	2	3	4	5	6
Growth parameter optimization	inoculum volume (%, v/v)	5	10	15	—	—	—
	Temperature (°C)	32	34	36	38	—	—
	Dissolved oxygen^a^ (mL)	25	50	75	100	—	—
	pH	6.6	6.8	7.0	7.2	7.4	—
	Glucose (g/L)	0.5	1	2	5	10	20
	Induced OD_600_	0.2	0.4	0.8	1.2	1.8	2.4

^a^ During optimization, following measurement with dissolved oxygen electrode, the dissolved oxygen ≥ 25%, when the volume of medium was 30 mL in 250-mL shake flask; the dissolved oxygen < 25%, when the volume of medium was 50, 70, or 100 mL in 250-mL shake flask.

#### Optimization of induction conditions on a flask scale

A 5-factor-3-level BOX-Behnken design (BBD) based response surface methodology (RSM) was conducted to optimize the effect of induction factors on bacterial yield (OD_600_) and expression level of hbFGF. This experimental design consisted of 46 runs, including five center point replications (Table 2; Supplementary Table S3). These induction strategies were implemented when the OD_600_ ranged from 0.8 to 1.2. The Design-Expert 8.0 software was used for experimental design, RSM model regression analysis, and RSM model optimization. Subsequently, the optimized culture and induction parameters were evaluated in the 200-L and 500-L fermentation.

**TABLE 2:** Independent variables and their levels used in the Box–Behnken design (BBD).

		Level		
		−1	0	1
Expression parameter optimization (RSM)	Temperature (°C)	30	34	38
	pH	6	7	8
	IPTG (mmol/L)	0.2	1	1.8
	NH4Cl (g/L)	0	0.09	0.18
	Induction time (h)	3	4	5

### Large-scale fermentation of hbFGF

The seed strain was inoculated into 650 mL of LB medium containing 100 μg/mL kanamycin sulfate (1:100, v/v) and incubated for 4–5 h at 37°C and 200 rpm. When OD_600_ reached 0.8–1.5, the culture was added to a 6.5-L seed amplification medium (1:10, v/v) containing 10.0 g/L tryptone, 4.0 g/L NaCl, 3.0 g/L K_2_HPO_4_, 10.0 g/L yeast extract and 1.0 g/L KH_2_PO_4_. The mixture was then incubated at 37°C and 170 rpm for 10–12 h. Subsequently, the culture was transferred to a 130-L fermentation medium in a 500-L fermentor (1:20, v/v; containing the following components in g/L: 17.0 tryptone, 4.0 NaCl, 23.0 yeast extract, 3.0 K_2_HPO_4_, 1.0 KH_2_PO_4_, 0.08 NH_4_Cl, 2.0 glucose, 0.013 CaCl_2_, 0.6 MgSO_4_, and 0.005 vitamin B1). The fermentor culture was incubated at 37°C and pH 6.8–7.0. After 2-h incubation, based on the growth status of cells, a 30% (w/v) glucose solution was introduced at varying rates. Subsequently, IPTG was added to a final concentration of 0.2 mM after 5 h of incubation (OD_600_, 19–24). The induction was performed at 38 °C and pH 6.3–6.7, and then the nitrogen source (containing the following components in g/L: 4.0 NaCl, 17.0 tryptone, 23.0 yeast extract, 4.0 MgSO_4_, 3.0 K_2_HPO_4_, and 1.0 KH_2_PO_4_) was added after 1 h induction. The dissolved oxygen (DO) was always maintained above 25%. The cell density (OD_600_) and protein expression level were measured hourly in the culture medium. After 5 h of induction, the cells were harvested by centrifuging at 16,000 rpm for 30 min at 4°C, and the pellets were stored at −70°C ± 5°C.

### Purification of hbFGF

The cell pellets were resuspended in 20 mM ice-cold Tris–HCl buffer (pH 8.0) containing 0.05 M NaCl, 10 mM EDTA-2Na, and 10% glycerol at a ratio of 1:10 (w/v). After high-pressure homogenization at 200–300 bar once and 800–900 bar twice, the supernatant was collected by centrifuging for 30 min at 9,000 rpm. Subsequently, the supernatant was applied onto a pre-balanced CM-Sepharose column (φ10 × 13 cm, 1,000 mL bed volume) at 50 mL/min, followed by washing with 5 column volumes (CVs) of buffer A (20 mM PB, pH 7.5, 0.1 M NaCl, and 10% glycerol) at 50 mL/min. Protein was eluted with 1.5–3 CVs of buffer B (20 mM PB, pH 7.5, 0.36 M NaCl, and 10% glycerol) at 50 mL/min and subsequently loaded onto a pre-equilibrated heparin affinity column (φ5.0 × 25 cm, 500 mL bed volume) at 20 mL/min. Following washing with 3.5 CVs of buffer C (20 mM PB, pH 7.5, 0.60 M NaCl, and 10% glycerol), the protein was eluted with 1.4–2.0 CVs of buffer D (20 mM PB, pH 7.5, 2.0 M NaCl, and 10% glycerol) at 20 mL/min. The pooled protein solution was mixed with buffer E (20 mM PB, 10% glycerol) at a ratio of 1:7 (v/v). The resulting mixture was loaded onto a pre-equilibrated SP-Sepharose column (φ5.0 × 13 cm, 250 mL bed volume) at 20 mL/min and subsequently washed with 2.5 CVs of buffer F (20 mM PB, pH 7.5, 0.25 M NaCl, and 10% glycerol). Finally, the target protein was harvested by employing 3.0–4.0 CVs of buffer H (20 mM PB, pH 7.5, 0.50 M NaCl, and 10% glycerol) and reposited at −70°C ± 5 °C. The concentration of hbFGF protein was determined using the bicinchoninic acid (BCA) method, while purity was assessed by SDS-PAGE, RP-HPLC, and SEC-HPLC. Subsequently, the pI of purified hbFGF was measured via isoelectric focusing electrophoresis (IEF), while the biological activity was evaluated on NIH-3T3 cells using an MTT assay. Moreover, the authenticity of purified hbFGF was further confirmed by Applied Protein Technology Co., Ltd. (Shanghai, China) through MALDI-TOF/MS, N/C-terminal sequencing, sequence coverage, and circular dichroism (CD) spectrum.

### Measurement of biological activity of hbFGF by MTT assay

NIH/3T3 cells were planted at degree 37°C and 5% CO_2_ in a complete medium composed of an 89% DMEM low glucose (1.0 g/L) medium, 10% fetal bovine serum (FBS; Gibco; Thermo Fisher Scientific, Inc.), and 1% penicillin/streptomycin (pen/strep). The cells were then transferred to a 96-well plate at a density of 5,000–7,000 cells/100 μL/well. Following incubation for 24 h, the cells were serum starved for 24 h by replacing the culture medium with 100 μL of fresh maintenance medium (containing 98.5% DMEM low glucose medium, 0.5% FBS, and 1% pen/strep). Subsequently, the culture medium was replaced with 120 μL of fresh maintenance medium, followed by the addition of 40 μL of hbFGF solution in a four-fold gradient dilution (100 ng/mL to 0.024 ng/mL). Each well was duplicated. After 48 h incubation, 20 µL of MTT solution (5 mg/mL) was added to each well and further incubated for 4 h. Subsequently, the medium was discarded, and 100 µL of dimethyl sulfoxide (DMSO) was added to each well and oscillated for 5 min. Ultimately, an MD Spectramax 190 microplate reader was used to record the absorbance of signal and background readings at 570 and 630 nm, respectively. The biological activity of hbFGF was calculated using SoftMax Pro 6.3 software.

### Animal model and experimental design

Male SD rats (250 g) with high blood glucose levels (≥11.1 mmol/L) were selected and a deep second-grace scald wound model (diameter, 2.2 cm; area, 3.8 cm^2^) was established as previously described (Hui et al., 2018b; Yu et al., 2021). The rats were individually housed in separate cages and randomly assigned to one of three groups: the negative c-group, positive c-group, and hbFGF group (*n* = 12 each). The negative control group received 0.2 mL of physiological saline every 2 days. The positive control group and hbFGF group were treated with a marketed drug of hbFGF (Gaifu^®^) and the purified hbFGF, respectively, at a dose of 150 IU/cm^2^ once every 2 days. The wound healing progress was monitored daily and photographed on days 0, 7, 10, 14, 21, 24, and 28 for subsequent wound area calculation by ImageJ software. The wound healing rate was calculated using the following equation: *R*_*i*_ = (*A*_*0*_ - *A*_*i*_)/*A*_*0*_, where *A*_*0*_ represents the initial wound area at day 0 and *Ai* represents the wound area on each photographed day. On day 14, six rats were randomly selected from each group and euthanized with 15% chloral hydrate (4.0 L/kg, i.p.). The skin tissue around the wound was excised and divided into two parts: one for biochemical analysis of factors such as FGF-1, TGF-β1, and hydroxyproline; while the other part was fixed in precooled 10% formaldehyde solution and processed into transverse paraffin sections (5 μm) for histopathological evaluation via hematoxylin-eosin (H&E), Masson’s trichrome staining, and immunohistochemistry (IHC).

### Statistical methods and analysis

Results were reported as mean ± standard deviation. Statistical analysis of quantifiable data was performed by employing Student’s t-test or one-way ANOVA with GraphPad Prism 8.0 software. The significance levels were denoted as: *p < 0.05, 0.001 < **p < 0.01, 0.0001 < ***p < 0.001, and ****p < 0.0001.

## Result

### Construction and identification of the hbFGF expression vector

The construction process of the hbFGF expression vector is illustrated in Figure 1A, wherein the *hbFGF* gene was synthesized and amplified using overlap PCR and standard PCR, respectively (Supplementary Tables S1, S2). By replacing the ampicillin resistance gene with a kanamycin resistance gene, we successfully constructed the modified plasmid pET3c-Kan (mpET-3c). The resistance detection assay confirmed that the *E. coli* BL21 (DE3) plysS strain transformed with pET3c-Kan exhibited growth in an LB medium containing 300 μg/mL kanamycin sulfate (Figure 1B). Furthermore, as depicted in Figure 1C (Lane 3), a band corresponding to the *hbFGF* gene was observed between 250 and 500 bp after restriction enzyme digestion, indicating the successful integration of the *hbFGF* gene into the mpET-3c plasmid. Moreover, upon induction, robust expression of the hbFGF protein was achieved with an apparent protein band detected between 14.3 and 20.1 kDa (Figure 1D, Lane 3). In addition, validation of its authenticity was confirmed by specific recognition of the expressed hbFGF protein using a monoclonal antibody against human bFGF (Figure 1D, Lane 5). Moreover, Moreover, genetic stability analysis after undergoing 30 generations revealed that the engineered strain *E. coli* BL21 (DE3) plysS-mpET3c/hbFGF exhibited excellent stability with no loss of plasmids observed (Supplementary Table S6; Supplementary Figure S1). Notably, there were no alterations in the plasmid structure or reduction in hbFGF expression level for this engineered strain. Furthermore, this engineered strain was stable for 12 months at −70°C ± 5°C (Supplementary Table S8).

### Fermentation condition optimization

**FIGURE 2:**
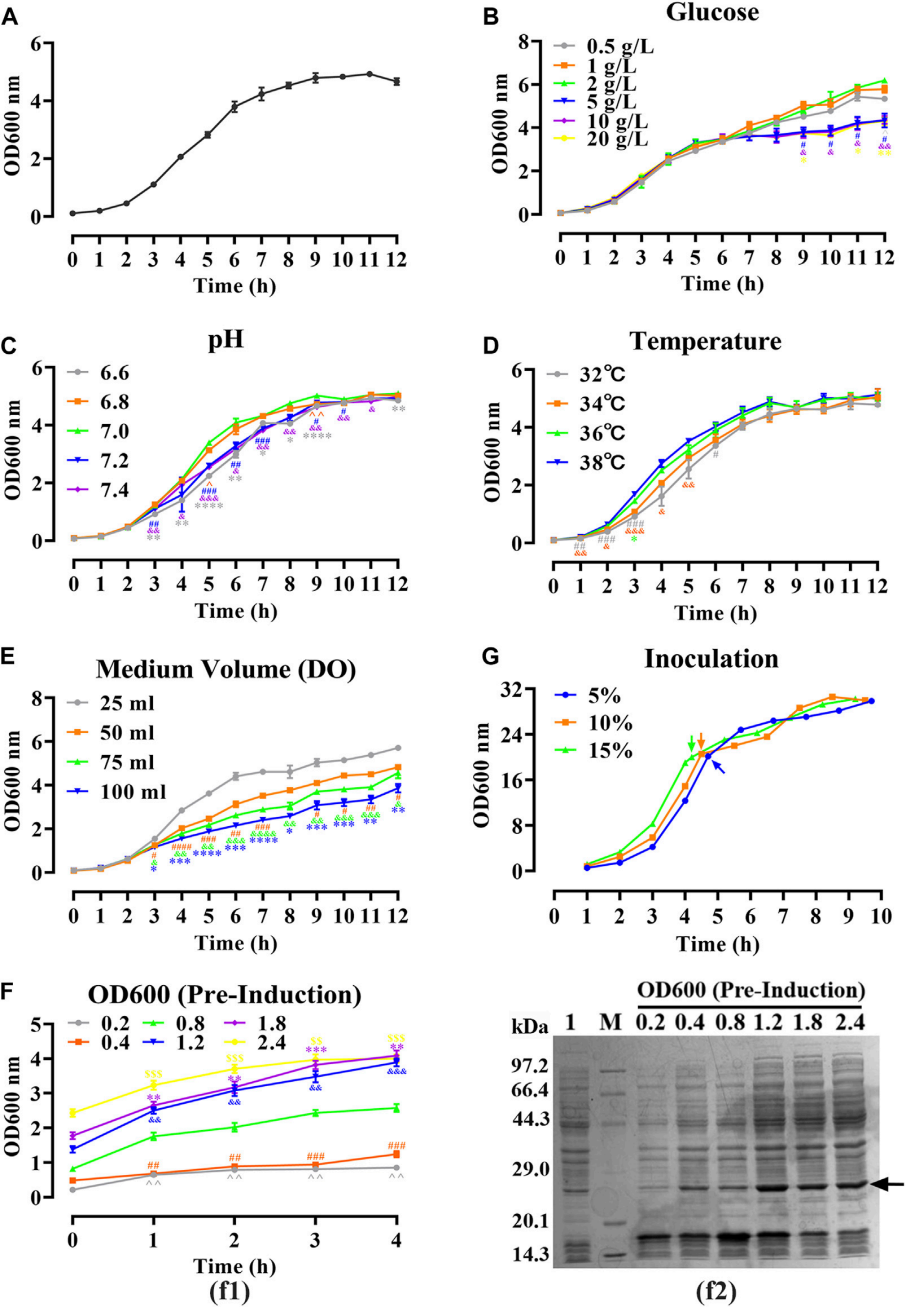
Optimizing of fermentation parameters of hbFGF *E. coli* strain in 30 mL of LB medium. **(A)** The 12-h growth curve of the hbFGF *E. coli* strain in 30 mL of LB medium at 37°C and 200 rpm. **(B–E)** The 12-h growth curve of hbFGF *E. coli* strain under different conditions, including **(B)** glucose concentrations in the range of 0.5–20 g/L, **(C)** pH in the range of 6.6–7.4, **(D)** temperatures of 32°C, 34°C, 36°C, and 38°C, and **(E)** medium volume (30, 50, 75, and 100 mL). **(F)** The expression level of hbFGF (f1) and bacterial density (f2) after induction at different OD_600_ values with 0.8 mM IPTG for 1–6 h. **(G)** The 10-h growth curve of the hbFGF *E. coli* strain under different inoculations. All experiments were performed in a 250-mL shake flask. Asterisks indicate significant difference (*p < 0.05, **p < 0.01, ***p < 0.001, *n* = 3).

**FIGURE 3:**
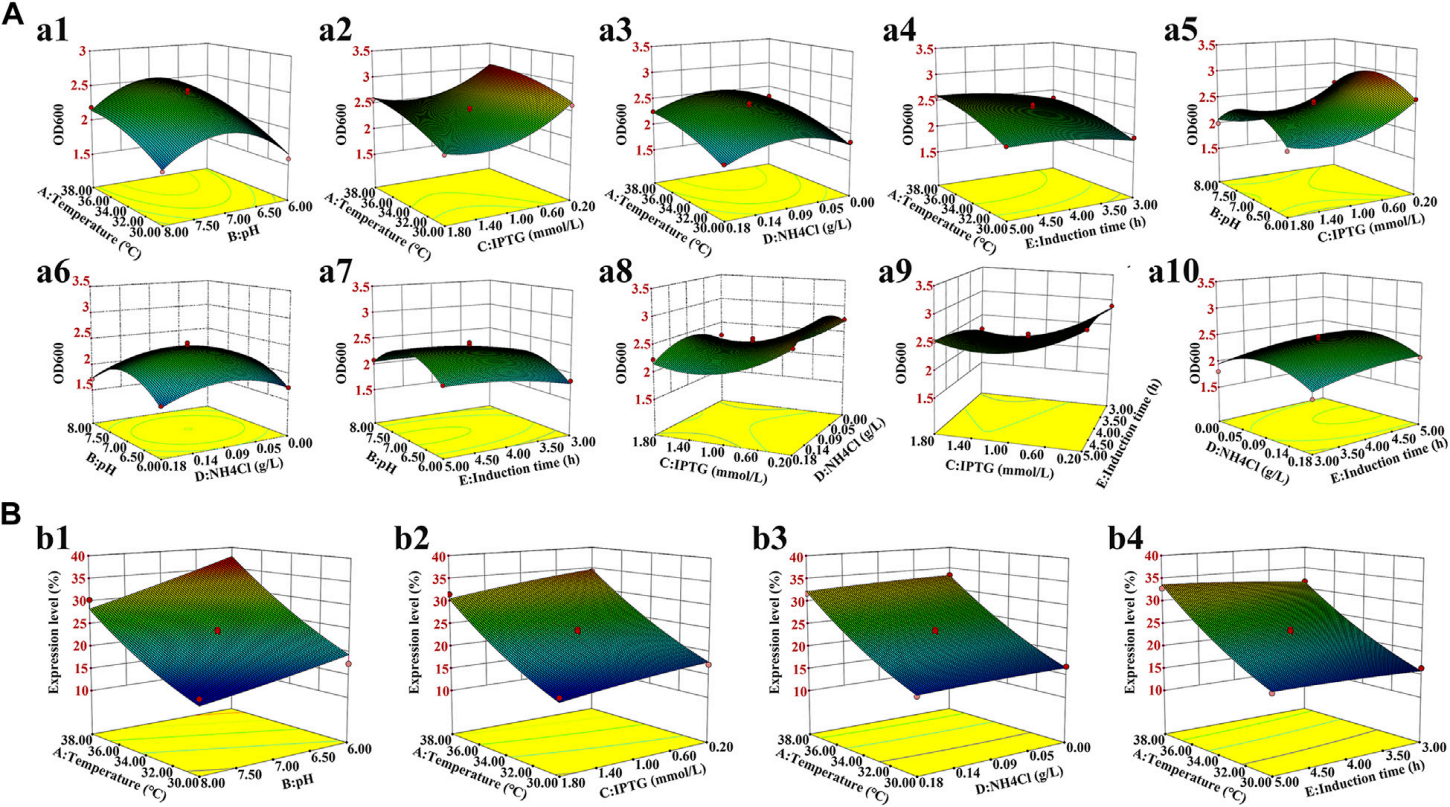
Three-dimensional response surface for the bacterial density and hbFGF expression level. **(A)** Effects on the bacterial density of **(a1)** temperature and pH; **(a2)** temperature and IPTG concentration; **(a3)** temperature and NH_4_Cl concentration; **(a4)** temperature and induction time; **(a5)** pH and IPTG concentration; **(a6)** pH and NH_4_Cl concentration; **(a7)** pH and induction time; **(a8)** IPTG concentration and NH_4_Cl concentration; **(a9)** IPTG concentration and induction time; and **(a10)** NH_4_Cl concentration and induction time. **(B)** Effect on the hbFGF expression level of **(b1)** temperature and pH; **(b2)** temperature and IPTG concentration; **(b3)** temperature and NH_4_Cl concentration; and **(b4)** temperature and induction time.

As shown in Figure 2A, a 12-h S-shaped growth curve was plotted to comprehend the growth characteristics of the hbFGF-engineered strain, which can be classified into four phases: lag phase (0–2 h), logarithmic growing phase (2–6 h), stationary phase (6–12 h), and decline phase (post-12 h). The optimum pH range, temperature range, and glucose concentration for the growth of the hbFGF strain were 6.8–7.0, 36°C–38°C, and 2 g/L, respectively (Figures 2B–D; Figure 3). It was observed that maintaining the DO level above 25% resulted in enhanced growth of the hbFGF strain (Figure 2E). Furthermore, for achieving higher bacterial density and protein expression level, induction at the mid-logarithmic growth stage (3 h, OD_600_ = 0.8–1.2) proved to be more suitable (Figure 2F). Moreover, as shown in Figure 2G; Supplementary Table S9, an increase in inoculum volume resulted in a gradual reduction of the lag phase and fermentation period (up to a maximum of 0.5 h). However, it was observed that the expression level consistently declined while the harvest yield of bacteria remained unaffected. Thus, a 5% (v/v) inoculum was applied in the 500 L fermentation.

Induction parameters, including temperature (A), pH (B), IPTG (C), NH_4_Cl (D), and induction time (E), were optimized as the five independent variables in the BBD experiment to achieve enhanced cell growth with maximum expression of hbFGF (Supplementary Table S3). Here, the response surface quadratic models were significant (*p* < 0.0001), with the regression coefficient (*R* ^*2*^) and adjusted *R* ^*2*^ over 0.95, and no significant lack-of-fit (*p* > 0.05), indicating that these models were adequately suitable for predicting the induction conditions (Supplementary Tables S4, S5). Notably, temperature, IPTG concentration, and induction time were all found to be significantly associated with OD_600_ and the expression level of hbFGF protein. Conversely, pH was only found to be significant concerning OD_600_ (Supplementary Table S4, S5; Figure 3). The optimal values for these five parameters that maximized both the hbFGF expression level and cell growth were as follows: temperature 38°C, pH 6.5, IPTG 0.2 mM, induction time 5 h, and NH_4_Cl 0.08 g/L, which were pre-estimated by the optimization utilities of Design Expert 8.0 software (Table 3). Next, these optimized fermentation conditions were preliminarily validated in a 200-L fermentor (containing 50 L medium). Compared to the conventional fermentation (temperature 37°C, pH 7.0, induction time 4 h, IPTG 1.0 mM), the utilization of these optimized fermentation parameters resulted in a significant enhancement in final hbFGF expression level, bacterial density, and bacterial yield (Table 4).

**TABLE 3:** The optimal induction conditions for the hbFGF fermentation.

Temperature (°C)	pH	IPTG (mmol/L)	NH_4_Cl (g/L)	Induced time (h)
38.00	6.515	0.2	0.079	4.858

**TABLE 4:** Summary of scale-up fermentation data for hbFGF (Mean ± SD, *n* ≥ 3).

		Expression level (%)	Bacterial density (g/L)	Bacterial wet weight (g)
Conventional	200-L	18.2 ± 2.2	40.4 ± 2.1	2624 ± 129
Post-optimization	200-L	27.2 ± 0.8****	43.6 ± 0.6**	2912 ± 137**
	500-L	28.2 ± 0.2****	46.8 ± 0.3***	7,797 ± 73****

Compared with the 200-L conventional fermentation (temperature 37°C, pH 7.0, IPTG, 1.0 mM, induction time 4 h), 0.001 < **p < 0.01, 0.0001 < ***p < 0.001, ****p < 0.0001.

### Large-scale fermentation of hbFGF

**FIGURE 4:**
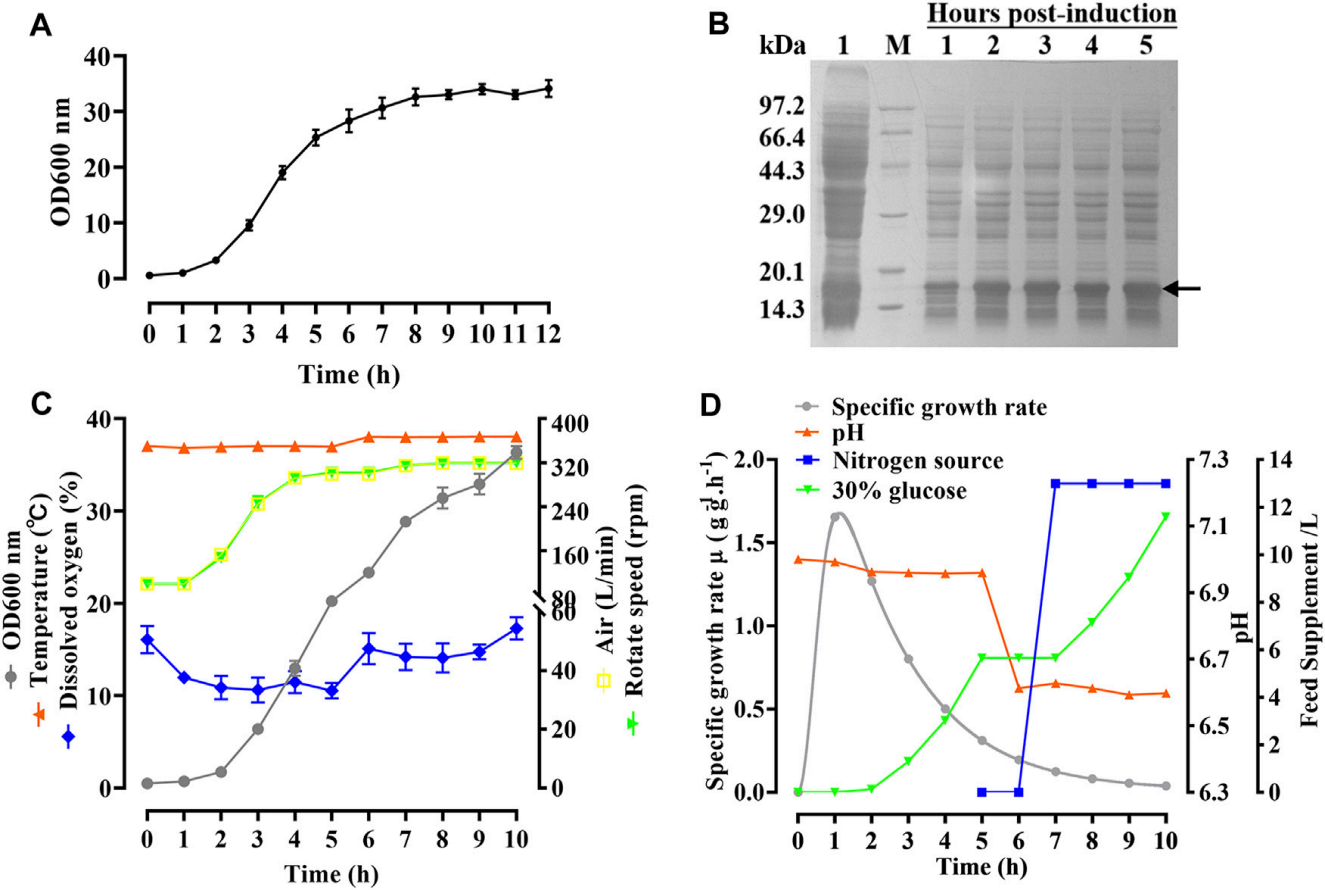
Process of fermentation of *E. coli* BL21 (DE3) plysS-mpET3c/hbFGF. **(A)** Growth curve of hbFGF engineered strain in a 200-L fermentation. **(B)** SDS-PAGE analysis of the hbFGF expression at the 500-L scale. #1 Lane, non-induced. Lane M, molecular weight marker. **(C)** Variations in the parameters for hbFGF production at the 500-L scale, including wet cell concentration (OD_600_), temperature, dissolved oxygen (DO) levels, rotation speed, and air ventilation rate. **(D)** The relative curves of the specific growth rate, pH, glucose addition, and nitrogen source addition with time in a 500-L fermentation. Black arrows indicate hbFGF.

As shown in Figure 4A, the growth curve of the hbFGF engineered strain in a 200-L fermentor demonstrated that the mid-logarithmic phase occurred approximately 5 h after culture. This time point served as the timing of induction for scaling up fermentation at 500 L (Figure 4C). After making minor adjustments, the optimized parameters mentioned above were subsequently applied in four consecutive batches of 500-L fermentations, as depicted in Figures 4C, D. During the fermentation growth stage, the temperature was maintained at 37 °C (Figure 4C). Furthermore, the inclusion of 2 g/L glucose in the fermentation medium facilitates the rapid transition of the hbFGF engineered strain through the lag phase within a mere 2 h. Subsequently, after 2 h of incubation, a 30% (w/v) glucose solution (carbon source) was introduced to maintain pH values between 6.8 and 7.0 (Figure 4D).

During the induction period, the temperature, pH, rotation speed, and air ventilation rate were maintained at 38°C, 6.5–6.6, 300 rpm, and 300 L/min, respectively (Figures 4C, D). After 1 h of induction, a slight expression of hbFGF was observed (Figure 4B). Subsequently, between the time interval of 1 and 2 h post-induction, a nitrogen source of 13 L was introduced, effectively inducing a transition in the cellular state from growth to targeted protein expression (Figure 4D). The expression level of hbFGF reached its peak and then remained stable at 4.0–4.5 h after induction (Figures 3b4, 4B). Additionally, after 2 h induction, approximately 6 L of 30% (w/v) glucose solution was added over a 3-h period (Figure 4D). Throughout the fermentation process, the DO level was effectively maintained at 30%–60% by gradually increasing the rotational speed from 100 to 300 rpm and enhancing the air ventilation rate from 100 to 300 L/min, without any addition of pure oxygen (Figure 4C). Furthermore, the plasmid loss rate was measured below 10% (Supplementary Table S7). Subsequently, at 5 h post-induction, the bacterial density reached 46.8 ± 0.3 g/L with an hbFGF expression level of 28.2% ± 0.2%, (Table 4; Figure 4B). Finally, after centrifugation, the bacteria weighing 7,797 ± 73 g were collected and stored at −20°C ± 5 °C (Table 4), remaining viable for up to 9 months (Supplementary Table S8).

### Pilot-scale purification and identification of hbFGF

**FIGURE 5:**
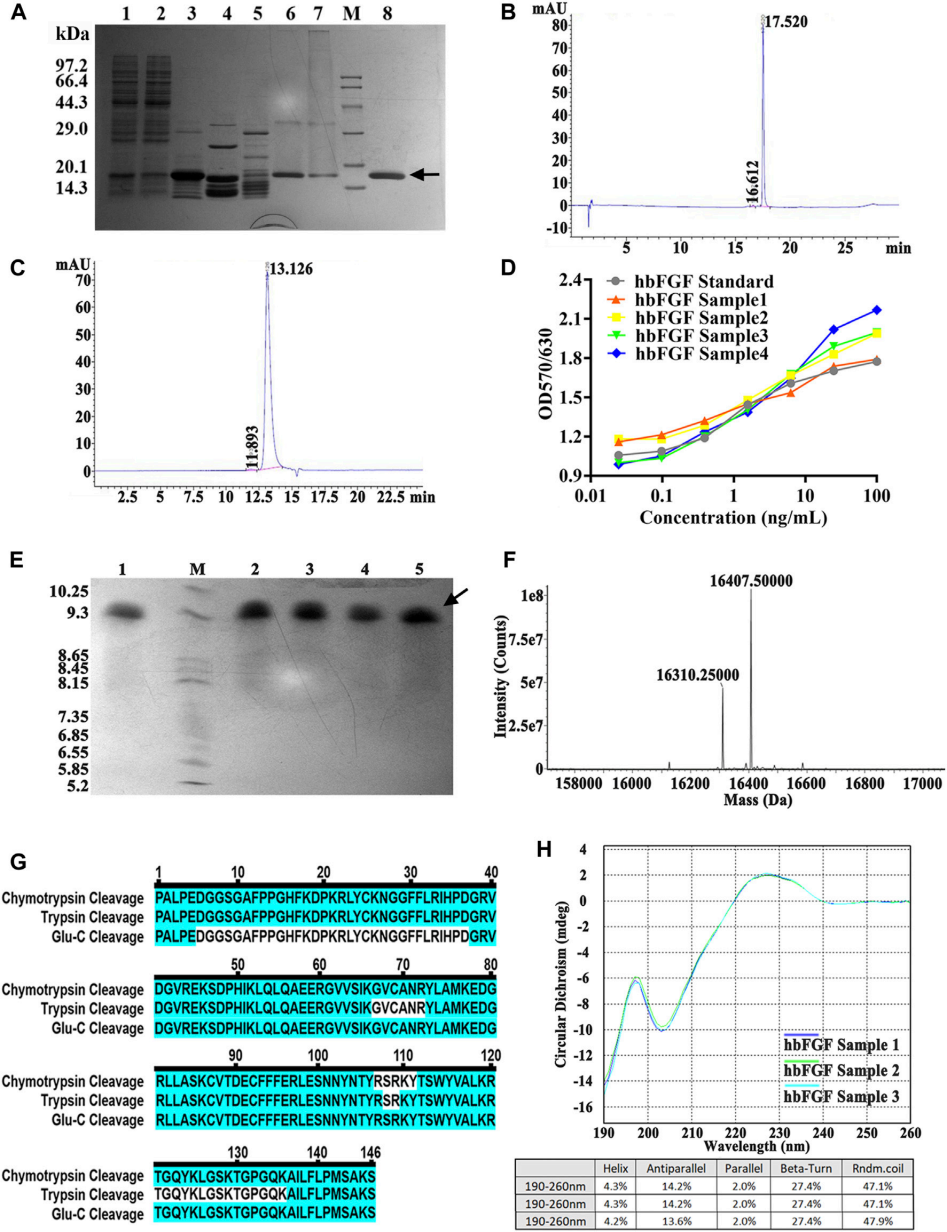
Identification and analysis of hbFGF in the purification process. **(A)** SDS-PAGE analysis of hbFGF after ion exchange and affinity chromatography. Lane 1, supernatant. Lane 2, the flow-through sample from CM-Sepharose. Lane 3, 0.36 M NaCl-eluted sample from CM-Sepharose. Lane 4, 2.0 M NaCl-regenerated sample from CM-Sepharose. Lane 5, flow-through fraction from heparin affinity Sepharose. Lane 6, 2.0 M NaCl-eluted sample from heparin affinity Sepharose. Lane 7, 2.0 M NaCl-regenerated sample from SP-Sepharose. Lane 8, purified hbFGF eluted with 0.5 M NaCl from SP-Sepharose. Lane M, molecular weight marker. **(B)** RP-HPLC and **(C)** SEC-HPLC analysis of purified hbFGF. **(D)** The biological activity of hbFGF on NIH-3T3 cells. **(E)** Analysis of Isoelectric point, **(F)** Mass spectrum, **(G)** molecular peptide mapping coverage, and **(H)** CD spectrum analysis of purified hbFGF. Black arrows indicate hbFGF.

Due to the mainly soluble expression of hbFGF, the supernatant was collected after lysing the stored frozen cell pellets. However, it was observed that hbFGF dimerization or multimerization tended to increase after heparin affinity chromatography (Supplementary Figures S2A). To address this issue, glycerol was added to the buffer solutions at a concentration of 10% (w/v) to reduce or eliminate hbFGF dimerization by increasing viscosity (Supplementary Figures S2B, C). Additionally, considering that glycerol may decrease the adsorption capacity of heparin resin for hbFGF, the NaCl concentration in the equilibration solution was reduced from 0.72 M to 0.6 M during the pilot-scale purification process to improve recovery. Subsequently, SP-Sepharose column chromatography was employed as the final purification step, effectively separating hbFGF monomers and multimers while ensuring that the content of hbFGF multimers in the final protein stock solution remained below 2% **(** Supplementary Figure S3). Finally, the hbFGF protein was successfully obtained through sequential utilization of CM-Sepharose, heparin affinity, and SP-Sepharose column chromatography (Table 5). It exhibited stable storage for 9 months at −70°C ± 5°C and 2 weeks at 5°C ± 3 C (data not shown). The recovery rates for each step were 2.2% ± 0.4%, 77.4% ± 5.6%, and 81.7% ± 7.8%, respectively (Table 5). SDS-PAGE analysis demonstrated that the purity of hbFGF obtained from each purification step was 75.9% ± 5.0%, 91.2% ± 5.9%, and 98.9% ± 0.9% (Table 5; Figure 5A). Notably, the final yield of hbFGF reached 114.6 ± 5.9 mg/L of culture, which is higher than prior studies (Tables 5, 6).

**TABLE 5:** Summary of the purification process for hbFGF (Mean ± SD, *n* = 4).

Steps of purification	Volume of purification (mL)	Total protein (mg)	Target protein (mg)	SDS-PAGE purity (%)	Recovery (%)
Bacteria lysis	8000 ± 0 (893.6 ± 23.2 g)^a^	153,420 ± 28,430.8	37,532.5 ± 6097.8	24.6 ± 1.8	—
CM-Sepharose	1422 ± 278.3	3236.6 ± 97.8	2458.4 ± 223.6	75.9 ± 5.0	2.2 ± 0.4
Heparin affinity	274.8 ± 23.1	2501.7 ± 141.9	2279.0 ± 133.4	91.2 ± 5.9	77.4 ± 5.6
SP-Sepharose	858.5 ± 88.3	2036.3 ± 92.9	2013.9 ± 93.6	98.9 ± 0.9	81.7 ± 7.8
Protein yield (mg/1 L culture): 114.6 ± 5.9					

^a^ The wet weight of bacteria for a single batch purification process.

**TABLE 6:** Comparison of hbFGF expressed for various hosts.

Host	Fermentation scale	Yield	Purity	References
*E. coli* DH5α^a^	5 L	—	—	Shen et al. (1999)
*E. coli* JM109^a^	40 L	63.7 mg/L	98.4%	Zhang et al. (2002)
*E. coli* DH5α^a^	30 L	—	—	Bai et al. (2002)
*E. coli* JM109^a^	40 L	97.5 mg/L	—	Feng et al. (2004)
*E. coli* JM109^a^	150 L	—	—	Wang et al. (2007)
*E. coli* BL21 (DE3)^a^	2 L	105.3 mg/L	98%	Chen et al. (2012)
*E. coli* BL (DE3)plysS^a^	5 L	94.8 mg/L	—	Liao. (2002)
*E. coli* BL21 (DE3)^b^	1 L	25–35 mg/L	—	Sheng et al. (2003)
*E. coli* BL21 (DE3)^c^	—	60–80 mg/L	95%	Imsoonthornruksa et al. (2015)
*E. coli* BL21 (DE3)^d^	—	—	—	Rassouli et al. (2013)
*E. coli* BL21 (DE3)^d^	—	—	—	Soleyman et al. (2016)
*E. coli* BL21 (DE3)^e^	0.25 L	—	—	Dong et al. (2021)
*E. coli* BL21 (DE3)^f^	5 L	1.42 g/L	96%	Rahman et al. (2020)
*Pichia pastoris* ^g^	—	91 mg/L	>94%	Mu et al. (2008)
*Pichia pastoris* ^g^	—	0.85 mg/L	98.8%	Le et al. (2020)
*Bacillus subtilis* ^a^	—	40 mg/L	—	Kwong et al. (2013)
*Bacillus subtilis* ^g^	2 L	84 mg/L	—	Hu et al. (2018)
*A. thaliana* ^g^	—	89.95 ng/mg oil body	—	Yang et al. (2018)
Soybean seed^h^	—	—	—	Ding et al. (2006)
*E. coli* BL (DE3)plysS	500 L	114.6 ± 5.9 mg/L	>99%	Present study

^a^ hbFGF_155_.

^b^ GST-hbFGF_155_.

^c^ 6His-hbFGF & Trx-6His-hbFGF.

^d^ His-hbFGF_146_.

^e^ Trx-hbFGF_146_.

^f^ Scl2-M-hbFGF.

^g^ hbFGF_146_.

^h^ bbFGF_155_.

As illustrated in Figures 5B, C, a distinct main peak was present at 17.520 and 13.126 min in RP-HPLC and SEC-HPLC analyses, respectively, indicating the attainment of high-purity hbFGF protein (>99%). The purified hbFGF exhibited a strong mitogenic activity similar to that of the hbFGF standard, reaching 1.05 ± 0.94 × 10^6^ AU/mg (Figure 5D). Consistent with theoretical values, the molecular weight and isoelectric point (pI) of purified hbFGF were measured as 16,407.5 Da and 9.8, respectively (Figures 5E, F). Furthermore, peptide mapping after trypsin, Glu-C, and chymotrypsin digestion resulted in 100% sequence coverage of purified hbFGF protein (Figure 5D). Additionally, the CD spectrum of purified hbFGF exhibited a broad positive signal near 227 nm and a negative signal around 204 nm (Figure 5H), which is characteristic of β-rich proteins with a β-II type consistent with previous observations (Alibolandi and Mirzahoseini, 2011; Dvorak et al., 2018; Krzyscik et al., 2022). In addition, the N-terminal 15 amino acid sequence was PALPEDGGSGAFPPGHFK, matching the sequence from the NCBI database (data not shown).

### Evaluation of the efficacy of hbFGF in promoting wound repair

**FIGURE 6:**
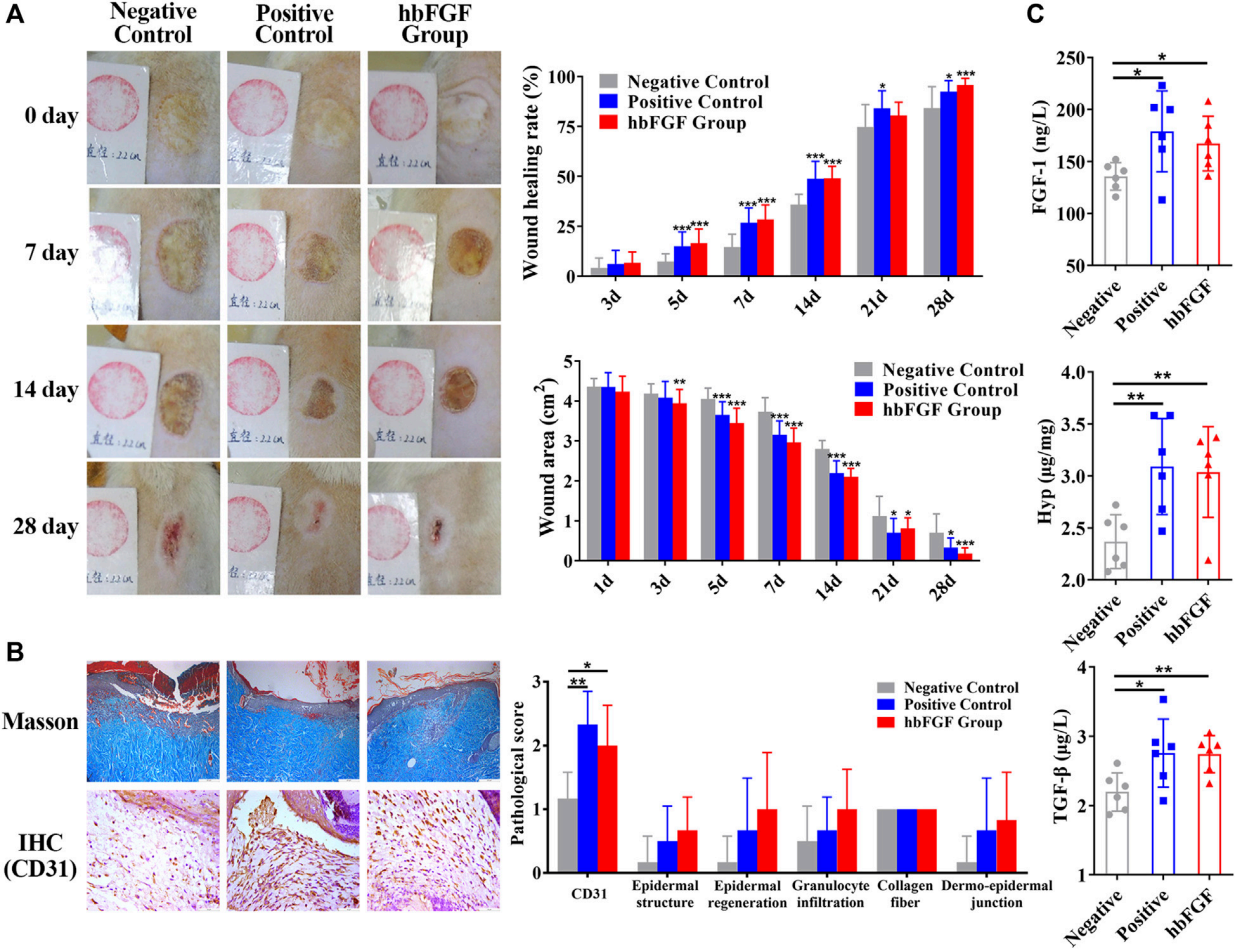
The healing effect of hbFGF on a deep second-degree scald wound model in rats. **(A)** Photos of the skin wound and wound healing rates of STZ-induced SD rats with deep second-degree scald wounds. **(B)** Results of Masson (scale bar, 200 μm) and IHC staining (scale bar, 50 μm) and the pathological score of a deep second-degree scald wound model in rats. **(C)** The expression level of FGF-1, TGF-β1, and hydroxyproline in a deep second-degree scald wound model in rats. Compared with the control group, *p < 0.05, **p < 0.01, ***p < 0.001, *n* = 7.

All rats survived after scalding and showed no evidence of infection during the period of this experiment. As shown in Figure 6A; Supplementary Figure S4, purified hbFGF and Gaifu^®^ (positive drug) showed a more significant and comparable effect on promoting wound healing when compared to the negative control group. Starting From day 3, the wound area gradually reduced and the wounding healing rate improved in the both positive and hbFGF groups, becoming more pronounced over time. On day 28, similar to the positive group, the hbFGF group demonstrated a statistically significant difference compared to the negative control group (0.70 ± 0.47 cm^2^ and 84.2% ± 10.7%), with a wound area of only 0.18 ± 0.14 cm^2^ and a wounding healing rate of 95.7% ± 3.42%.

After a 14-day treatment, pathological evaluation and biochemical factor detection were performed on the healing skin tissue. The results of the positive control group and hbFGF group were similar. Figure 6B; Supplementary Figure S5 demonstrate that hbFGF treatment promoted the recovery of epidermal structure, epidermal regeneration, and reduction of granulocyte infiltration; however, there was no statistical significance with the negative control group. CD31-positive cells significantly increased in the hbFGF group, indicating enhanced angiogenesis. Similarly, expression of FGF-1, TGF-β1, and Hyp was significantly increased in the hbFGF group, promoting the growth of fibroblasts, capillary angiogenesis, collagen production, and granulation tissue growth to accelerate wound healing (Figure 6C).

## Discussion

Due to the considerable pharmacological potential but the challenge in obtaining hbFGF from natural sources in quantity, many endeavors have been undertaken to efficiently produce hbFGF. Nowadays, hbFGF has been successfully produced in various hosts, such as *E. coli, Bacillus subtilis*, yeast, *A.thaliana*, and soybean seed. Some of these proteins were expressed with fusion tags, including GST, His, Trx, and Scl2. Zhang et al. (2002) and Feng et al. (2004) elucidated the manufacturing process of hbFGF_155_ at a 40-L fermentation in *E. coli*, while Wang et al. (2007) achieved the production of hbFGF_155_ at the 150-L scale in *E. coli*. Rassouli et al. (2013), Dong et al. (2021), and Soleyman et al. (2016) respectively expressed hbFGF_146_, hbFGFK18S/S69C 146, and Trx-hbFGF_146_ at the shake flask scale. Recently, Yang et al. (2018), Hu et al. (2018), and Le et al. (2020) have also expressed hbFGF_146_ in *A. thaliana*, *Bacillus subtilis*, and *Pichia pastoris*, respectively. However, there is a dearth of studies on the large-scale high-density fermentation and purification of hbFGF in *E. coli*, particularly for hbFGF_146_. In the study, we present a robust and efficient 500-L scale fermentation process, coupled with a pilot-scale purification strategy, to enable the large-scale production of high purity and high activity hbFGF_146_ protein in *E. coli*.

The commercial *E. coli* BL21 (DE3)plysS strain and pET3c plasmid have been extensively employed for the production of recombinant proteins, including hbFGF. Previous studies have reported that the plasmid carrying T7 promoter achieved maximum expression levels of hbFGF *in E.coli* compared to *trc*, *tac*, and *λ*PR promoter under IPTG induction (Mirzahoseini et al., 2004). However, it is important to note that according to the regulations of Pharmacopoeia (Chinese Pharmacopoeia Commission, 2020 edition), the use of β-lactam antibiotics such as ampicillin during the recombinant protein production process is prohibited, and it is suggested to avoid incorporating the ampicillin-resistant gene while constructing expression vectors. Therefore, we made modifications to the pET3c plasmid by substituting its ampicillin resistance gene with a kanamycin resistance gene in this study.

Nutrients play a pivotal role in the high-density fermentation of *E.coli*. Glucose, a commonly used quick-acting carbon source for the high-density fermentation of *E. coli*, is particularly suitable for short-term (8–12 h) fermentations (Liao. 2002). A high-density fermentation medium with an appropriate initial concentration of glucose is more conducive to bacterial growth and the expression of the target protein. In contrast, excessive glucose induces the production of numerous acidic by-products, such as acetic acid, resulting in a decrease in pH inhibition of both bacterial growth and target protein expression (Li and Chen, 2000). Feng et al. (2004) recommended maintaining a glucose concentration below 1 g/L when producing hbFGF in *E. coli* JM-109. Herein, we maintained the glucose at a low level (within 2 g/L) using a glucose-pH-state strategy, which provided quick-acting energy for the rapid growth with a high specific growth rate of the engineered strain during the growth stage and the massive expression of hbFGF during the induction stage. Nitrogen is another limiting nutrient in the high-density fermentation of *E. coli.* The mixture of yeast extract and tryptone, as a nitrogen source, was found to be more beneficial to the growth of recombinant *E. coli* strains and the expression of the target protein (Yan et al., 2010; Pei et al., 2020). Previously, it was observed that the expression of hbFGF increased when the yeast extract: tryptone ratio was 1:1; however, changing the mixing ratio did not affect the growth of recombinant *E. coli* strains (Liao. 2002). Here, the mixture of yeast extract and tryptone with a ratio of 23:17, as a nitrogen source, was added to the fermentation medium (Figure 4D). This strategy has been successfully used for the large-scale preparation of hFGF21 (Hui et al., 2019) and haFGF_135_ (Yu et al., 2021) in *E. coli*. In addition, microelements and inorganic ions such as Ca^2+^, Mg^2+^, Na^+^, K^+^, NH^4+^, and vitamin B1 are essential for the normal growth and metabolism of microorganisms. These elements play a crucial role in regulating enzyme activity and maintaining cellular osmotic pressure to ensure the proper functioning of cells. Consistent with our previous studies, the fermentation medium was supplemented with 4.0 g/L NaCl, 1.0 g/L MgSO_4_.7H_2_O, 20 mM PO4^3−^, 13 mg/L CaCl_2_, and 5.0 mg/L vitamin B1 to enhance bacterial growth and protein expression while improving plasmid stability.

The regulation of dissolved oxygen (DO) is crucial in high-density fermentation due to its direct involvement in the oxidative catabolic process of *E. coli*. Inadequate DO levels can lead to the accumulation of excessive acetic acid through glycolysis, thereby inhibiting cell growth and reducing both specific growth rate and biosynthesis ability, as well as intracellular amino acid content (Zhong et al., 2005). Furthermore, plasmids exhibit rapid loss when DO levels fall below 5% (Cai et al., 2000). Shen et al. (1999) observed a remarkable 56% increase in the final yield of a recombinant hbFGF strain and a substantial 69% reduction in acetic acid concentration at the 12-h post-fermentation stage when DO was maintained above 30%, as compared to fermentations without DO control. Similarly, Bai et al. (2002) demonstrated significant enhancement in hbFGF expression during fermentation with a DO level exceeding 30%. We also precisely controlled the DO between 30% and 60% by gradually increasing agitation speed and ventilation rate during the 500-L fermentation of hbFGF.

As an important parameter for high-density fermentations, pH serves as a comprehensive indicator of cell metabolic activity under specific conditions. A previous study demonstrated that recombinant hbFGF/*E. coli* BL21 (DE3) plysS exhibited robust growth at pH 6.8–7.1, especially at pH 6.8, and variations in pH within the range of 6.5–7.5 did not significantly affect the expression of hbFGF (Liao. 2002). Similarly, in our study, the hbFGF engineered strain showed optimal growth within a range of pH 6.8–7.0, with an optimum observed at 7.0. However, we observed a gradual decrease in the expression of hbFGF as the pH decreased from 8.0 to 6.0. Furthermore, in *E. coli* high-density fermentations, a diminished concentration of acetic acid was observed under low pH culture conditions compared to high pH culture conditions (Zhang et al., 2021). Thus, we consistently controlled the pH values around 6.6 during the induction stage of 500-L scale fermentation. Notely, throughout the fermentation, pH regulation was solely accomplished by feeding glucose using a glucose-pH-state strategy. This finding indirectly suggests that the acetic acid generated through glucose metabolism was promptly neutralized or metabolized, maintaining a constant low-level dynamic equilibrium state without continuous accumulation to inhibit cell growth and hbFGF expression. Temperature is also a crucial factor that impacts the activity of various enzymes, thereby affecting the growth and metabolism of engineered bacteria, the formation of recombinant products, and the stability of plasmids. In this study, we observed the hbFGF engineered strain exhibited optimum growth at 37 °C and an increase in hbFGF expression level with rising temperature from 30°C to 38°C. This could be attributed to improved expression and activity of T7 RNA polymerase at higher temperatures. Therefore, optimal temperatures for growth and induction stages during the 500-L fermentation process were determined as 37°C and 38°C respectively.

IPTG, as a common inducer, has been applied to the induction expression of hbFGF and its fusion protein in recombinant *E. coli* high-density fermentations, within a concentration range of 0.1–1 mM. Rassouli et al. (2013) further demonstrated that 0.2 mM IPTG was adequate for the complete activation of the *lac* promoter system to achieve high expression of hbFGF. However, we observed a gradual inhibition of the growth of the hbFGF engineered strain in response to increasing concentrations of IPTG within the range of 0.2–1.0 mM, attributed to its inherent toxicity, despite achieving satisfactory induction efficiency. Moreover, due to its toxic effects, the premature addition of IPTG hindered the growth of hbFGF engineered strains and reduced their cell viability, consequently leading to decreased bacterial yield and hbFGF expression level. On the other hand, overly late induction leads to reduced or even no hbFGF expression in the recombinant strains, caused by excessive cell growth, nutrient depletion, and environmental deterioration (Figure 2F). Thus, it is recommended to add IPTG during the mid-logarithmic growth phase to ensure the final product yield. In addition, the inoculum volume was also optimized and a higher bacterial density and expression level of hbFGF was achieved with 5% (v/v) inoculum.

As we know, hbFGF is a high-affinity heparin-binding protein with a pI of 9.6. Therefore, the combination of CM-Sepharose and heparin affinity column chromatography was preferentially adopted to purify hbFGF, which is consistent with earlier studies (Feng et al., 2004). However, Platonova et al. (2014) reported that hbFGF purified with heparin affinity can dimerize, while hbFGF purified without heparin cannot. To eliminate the hbFGF dimers or multimers, we implemented two enhancements: firstly, the addition of 10% (w/v) glycerol to the purification buffer was employed to augment its viscosity and mitigate hbFGF multimer formation; secondly, leveraging the disparity in charge properties between hbFGF monomers and multimers, we incorporated SP column chromatography technique for their efficient separation, thereby further enhancing protein purity. Previously, Zhang et al. (2002) utilized Sephacryl S-100 column chromatography as the final purification step and successfully enhanced the monomeric content of hbFGF from 93.2% to 98.4%. However, this strategy has limitations in terms of large-scale production. In addition, in this study, as the purification process proceeded, the levels of host cell proteins, exogenous DNA residues, and antibiotic residues were gradually decreased to below 1,000 ng/mg, 2.5 × 10^−4^ ng/IU and 2 ng/mL, respectively, meeting the requirements of the Chinese Pharmacopoeia (2020th edition) (data not shown). Moreover, the total amount of hbFGF obtained after one batch purification was sufficient to support the production of approximately 40,000 bottles of Gaifu^®^ (approximately 50 µg per bottle), indicating that this purification protocol has the potential for scaling up to an industrial level.

The characteristics and authenticity of the purified hbFGF protein were identified by various methods, which were consistent with the theory. Cell and animal model validity evaluations were performed to confirm the actual biological effects of the purified hbFGF. In the MTT assay, the purified hbFGF and the standards had comparable mitogenic activity on NIH-3T3 cells. Furthermore, compared to Gaifu^®^, the purified bFGF demonstrated similar effects on wound healing in type 1 diabetic rats with deep second-degree scald wounds.

## Conclusion

In this study, we have successfully developed an engineered strain of *E. coli* BL21 (DE3)plysS- mpET3c/hbFGF and a large-scale production protocol for hbFGF that includes a stable and efficient 500-L scale high-density fermentation process as well as a purification protocol with high yield. These protocols lay the foundation for industrial-scale production of hbFGF. However, there are still certain aspects that require further investigation or exploration in the future, such as improving the expression level and yield of hbFGF, conducting metabolomics analysis on fermentation processes, and analyzing the removal effects of non-host-derived impurities.

## Data Availability

The original contributions presented in the study are included in the article/Supplementary Material, further inquiries can be directed to the corresponding authors.

## Abbreviations

BBD, optical density at 600 nm (OD600); BOX-Behnken design; CV, column volume; CD, circular dichroism; DMEM, Dulbecco’s modified Eagle medium; DO, dissolved oxygen; DMSO, dimethyl sulfoxide; FBS, fetal bovine serum; pen/strep, penicillin/streptomycin; hbFGF, Human basic fibroblast growth factor; HPLC, size-exclusion liquid chromatography; IHC, immunohistochemistry; H&E, hematoxylin and eosin staining; IEF, isoelectric focusing electrophoresis; IPTG, isopropyl-β-D-thiogalactoside; MTT, 3-(4,5-dimethyl-2-thiazolyl)-2,5-diphenyl-2-H-tetrazolium bromide; PCR, polymerase chain reaction; pI, isoelectric point; RSM, response surface methodology; RP-HPLC, reverse-phase high-performance liquid chromatography; SDS-PAGE, sodium dodecyl sulfate-polyacrylamide gel electrophoresis; SEC-PB, phosphate buffer; STZ, streptozocin.
