# Supplementary figures and images for: Scaling up production of recombinant human basic fibroblast growth factor in an Escherichia coli BL21(DE3) plysS strain and evaluation of its pro-wound healing efficacy
Source: Front Pharmacol. 2024 Feb 5;14:1279516. doi: 10.3389/fphar.2023.1279516 (PMC10875678; doi:10.3389/fphar.2023.1279516)

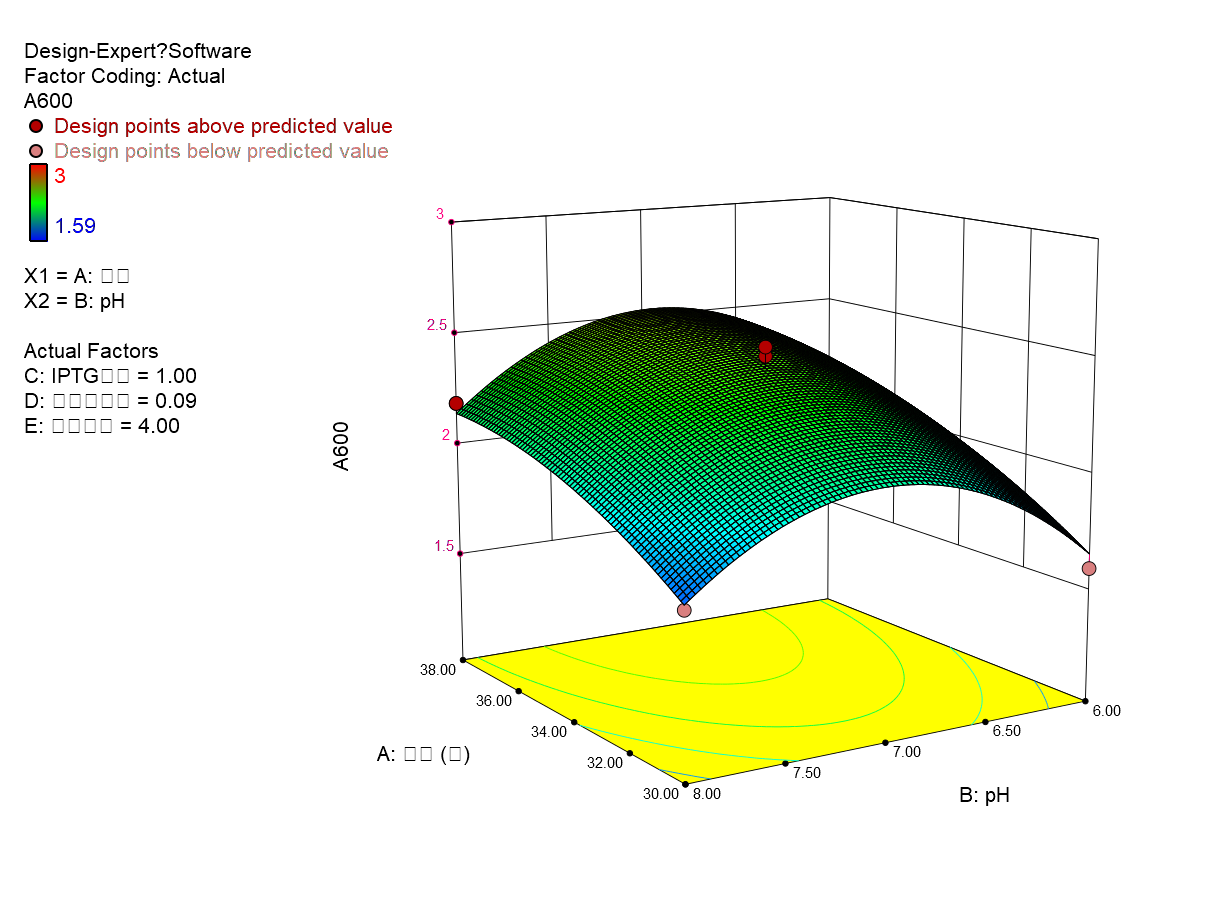

Supplement: Supplementary file 1 [file DataSheet3.ZIP › Figure 3/Fig 3a1.png]

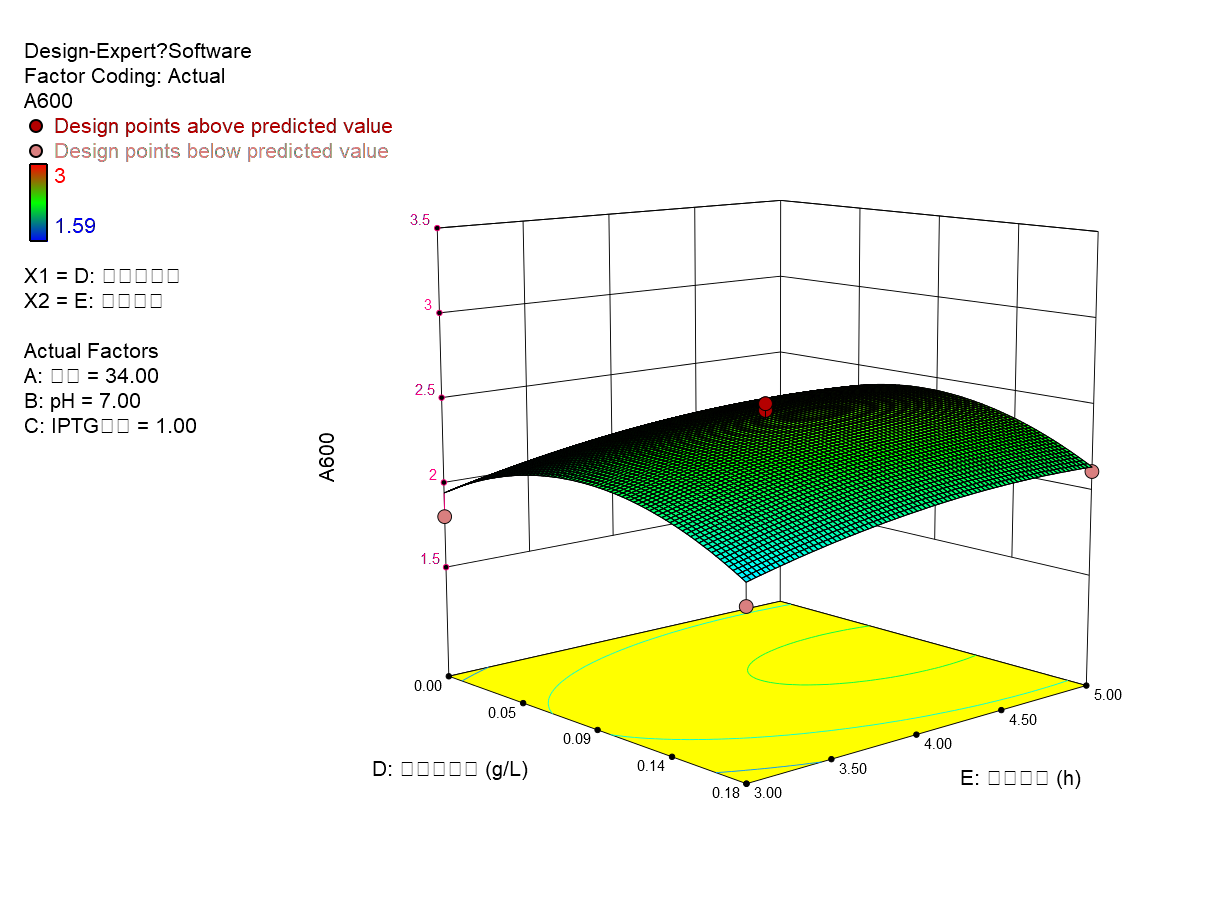

Supplement: Supplementary file 1 [file DataSheet3.ZIP › Figure 3/Fig 3a10.png]

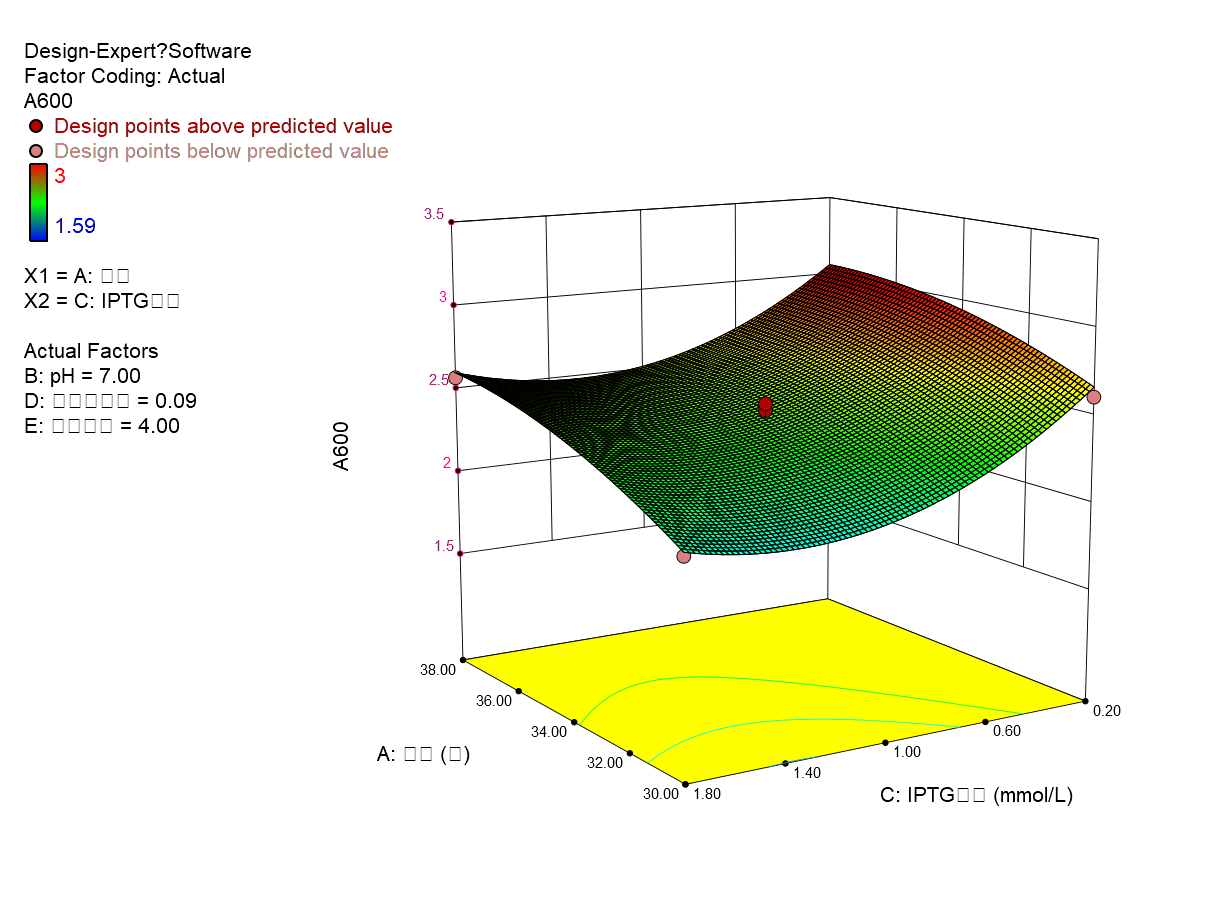

Supplement: Supplementary file 1 [file DataSheet3.ZIP › Figure 3/Fig 3a2.png]

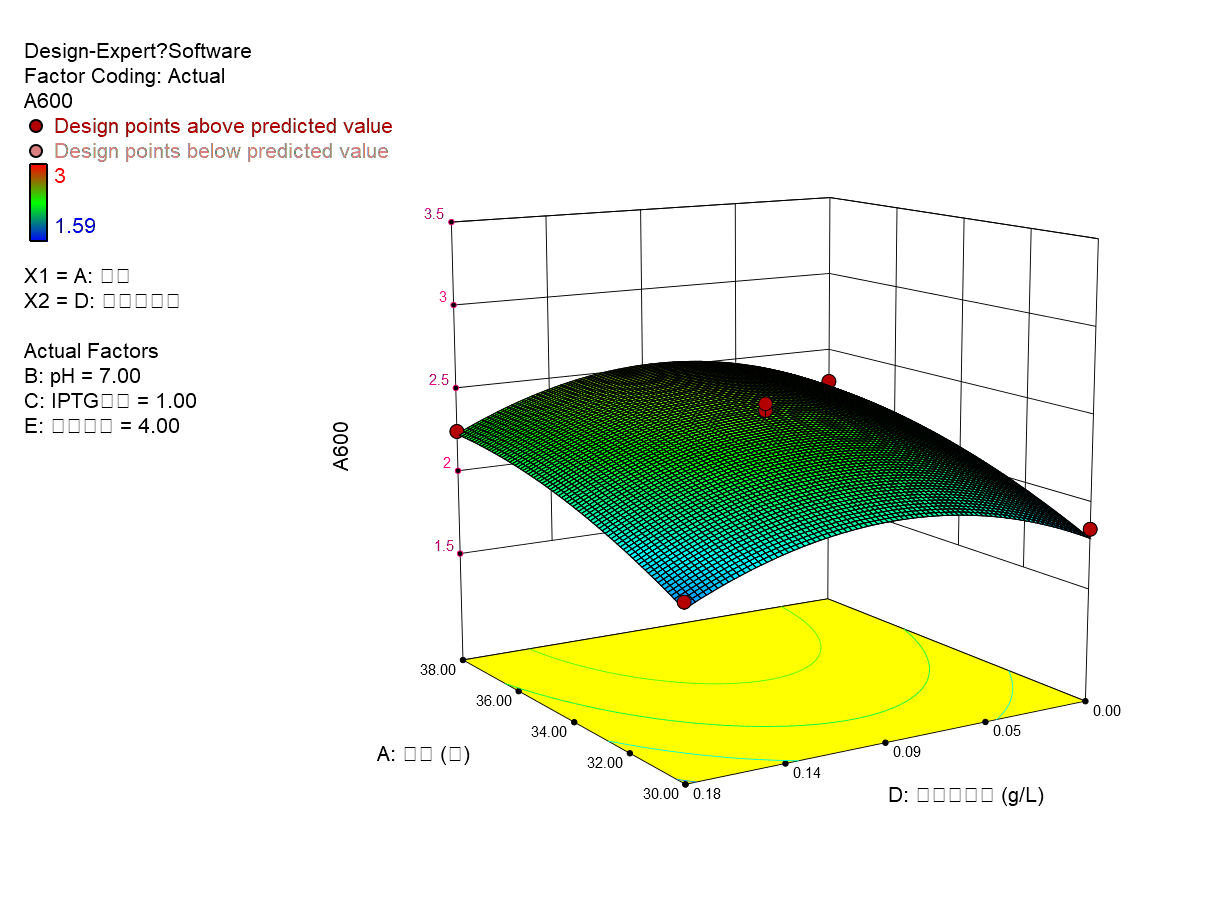

Supplement: Supplementary file 1 [file DataSheet3.ZIP › Figure 3/Fig 3a3.png]

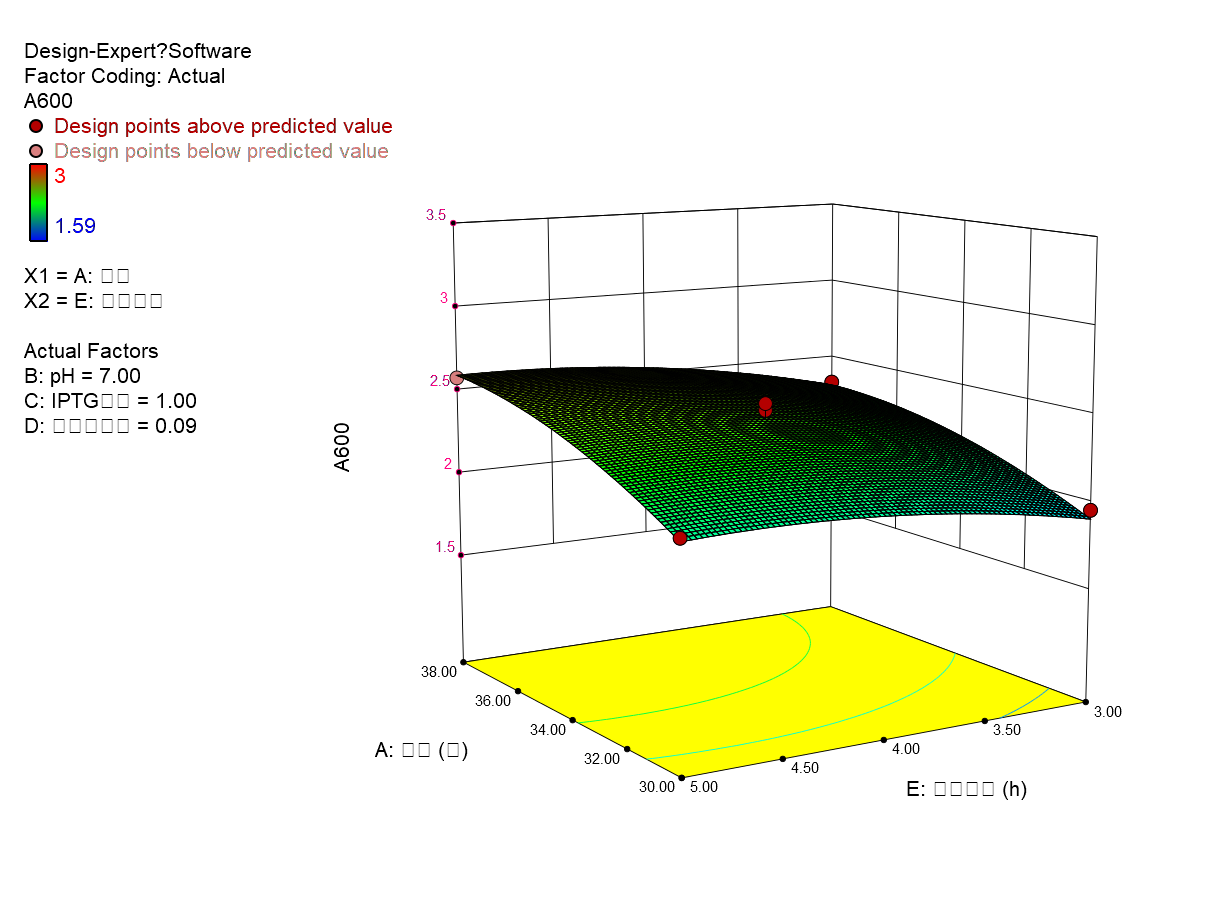

Supplement: Supplementary file 1 [file DataSheet3.ZIP › Figure 3/Fig 3a4.png]

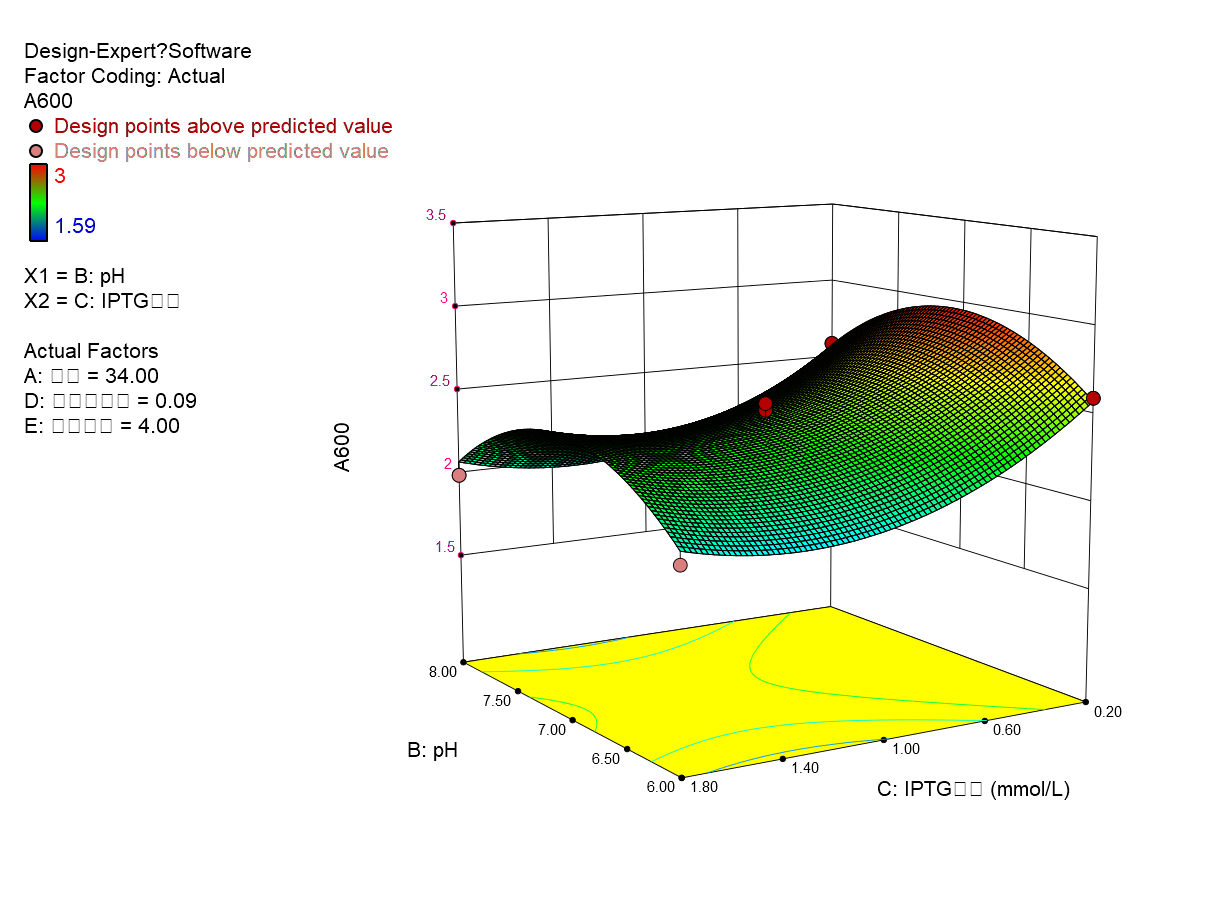

Supplement: Supplementary file 1 [file DataSheet3.ZIP › Figure 3/Fig 3a5.png]

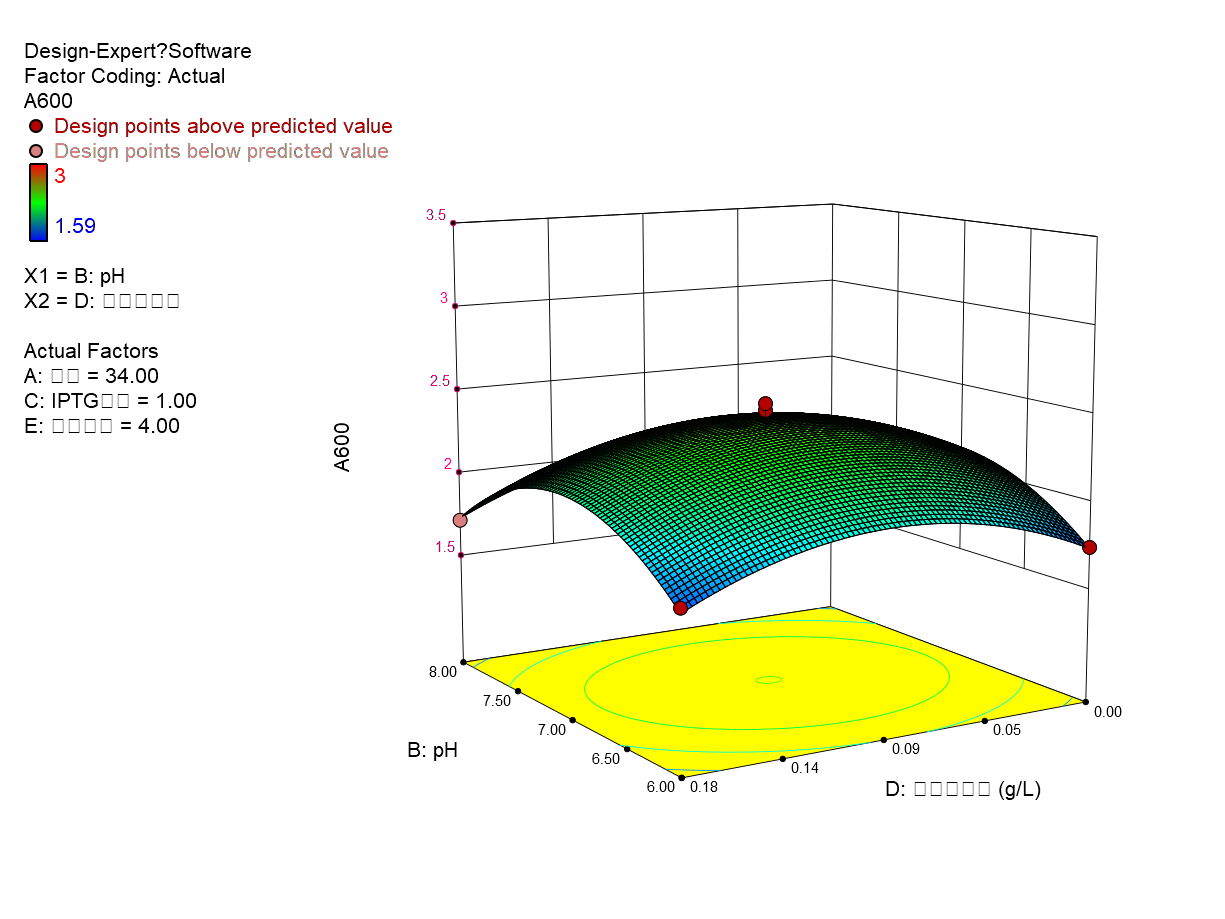

Supplement: Supplementary file 1 [file DataSheet3.ZIP › Figure 3/Fig 3a6.png]

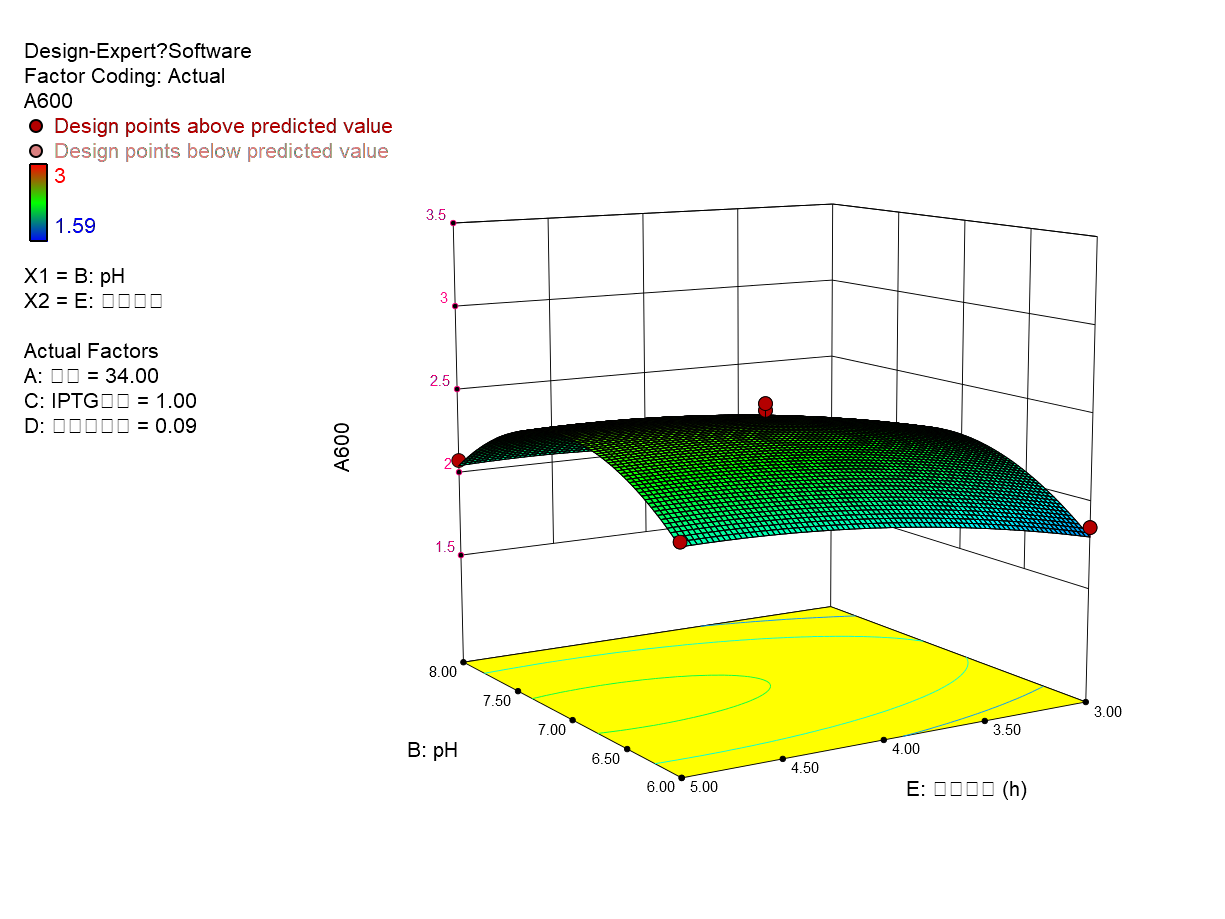

Supplement: Supplementary file 1 [file DataSheet3.ZIP › Figure 3/Fig 3a7.png]

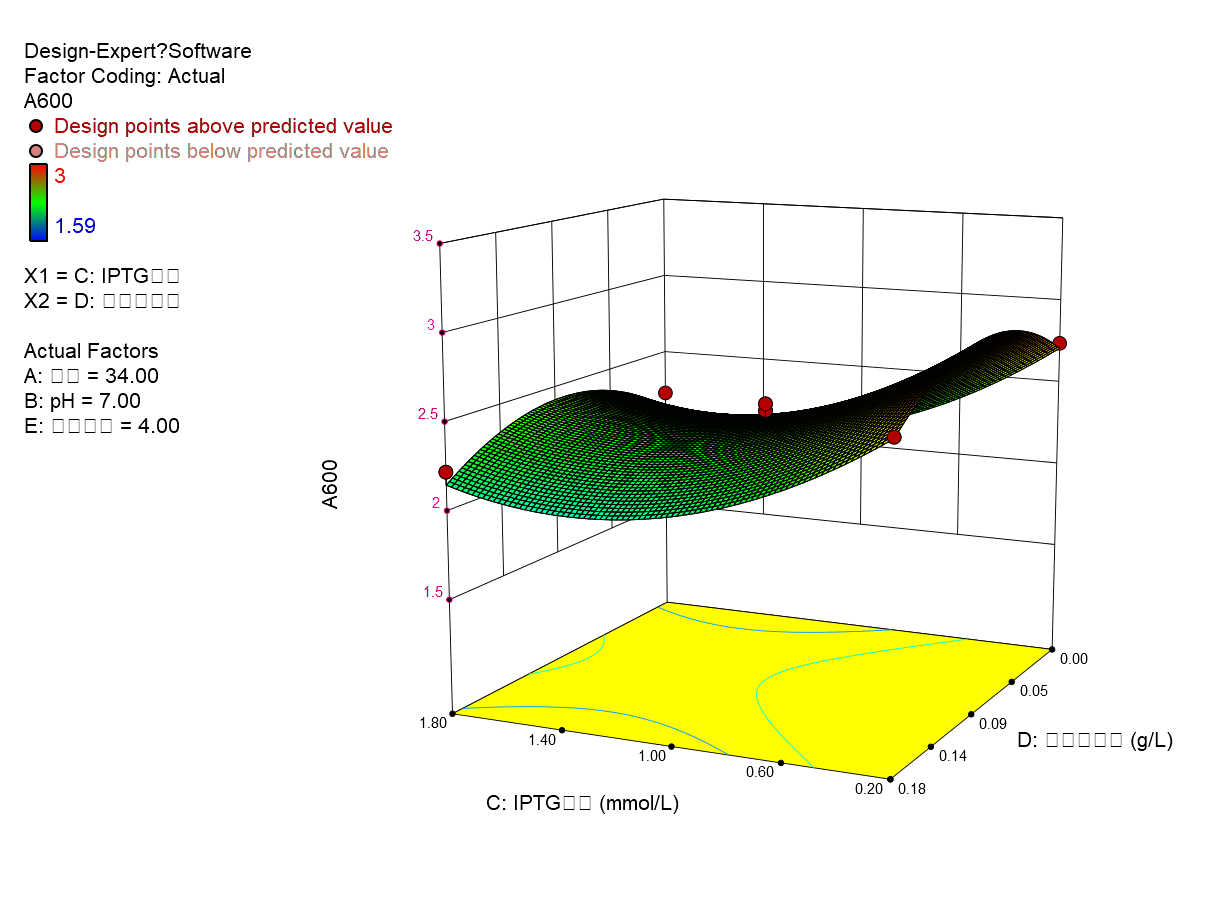

Supplement: Supplementary file 1 [file DataSheet3.ZIP › Figure 3/Fig 3a8.png]

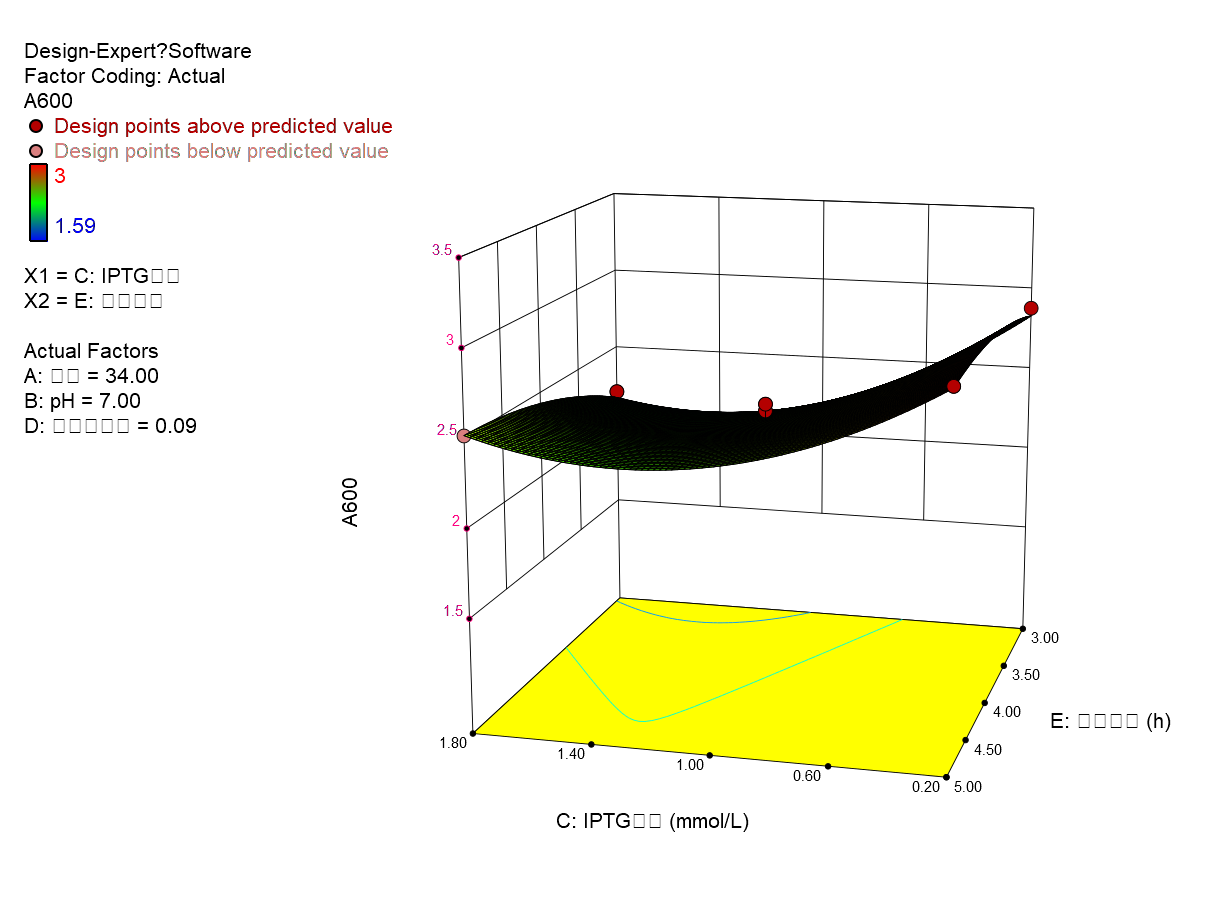

Supplement: Supplementary file 1 [file DataSheet3.ZIP › Figure 3/Fig 3a9.png]

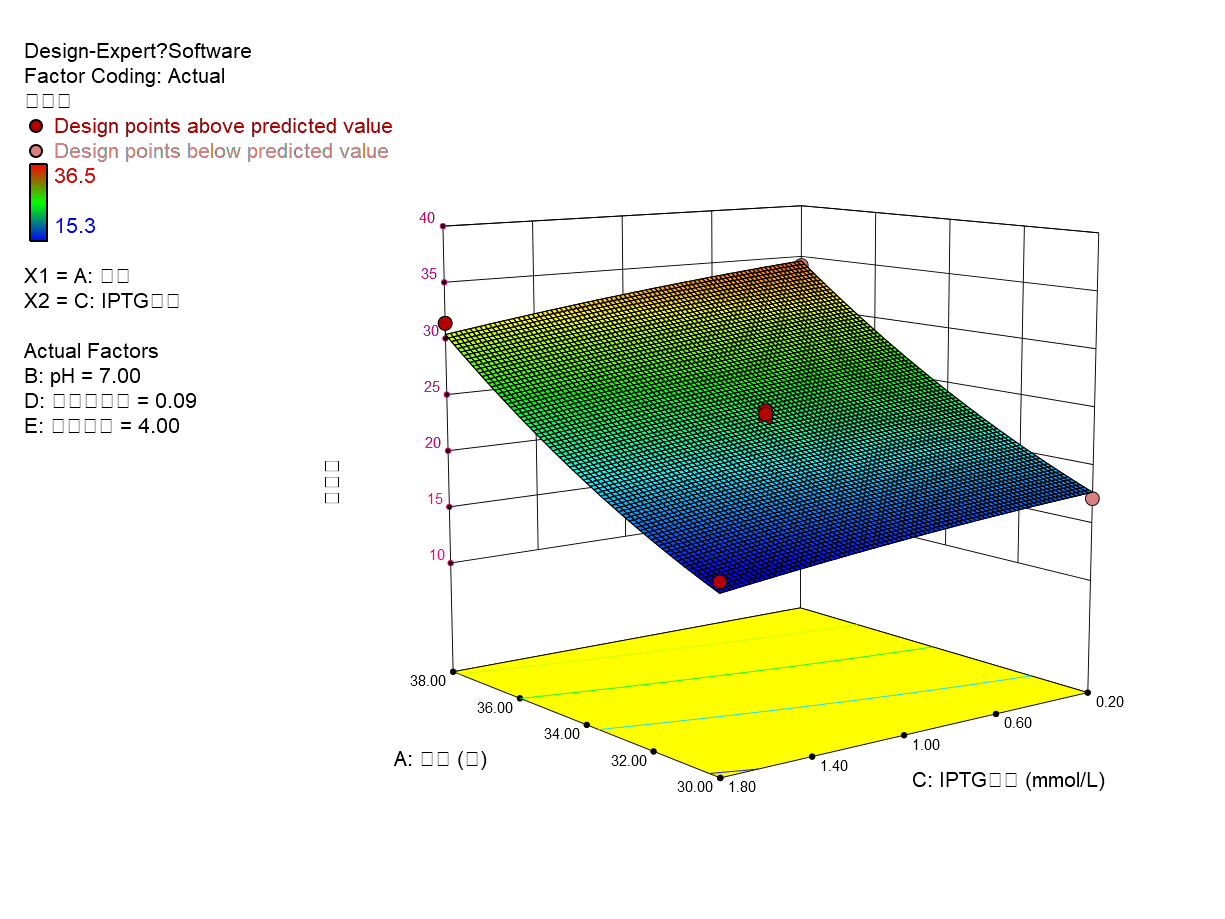

Supplement: Supplementary file 1 [file DataSheet3.ZIP › Figure 3/Fig 3b2.png]

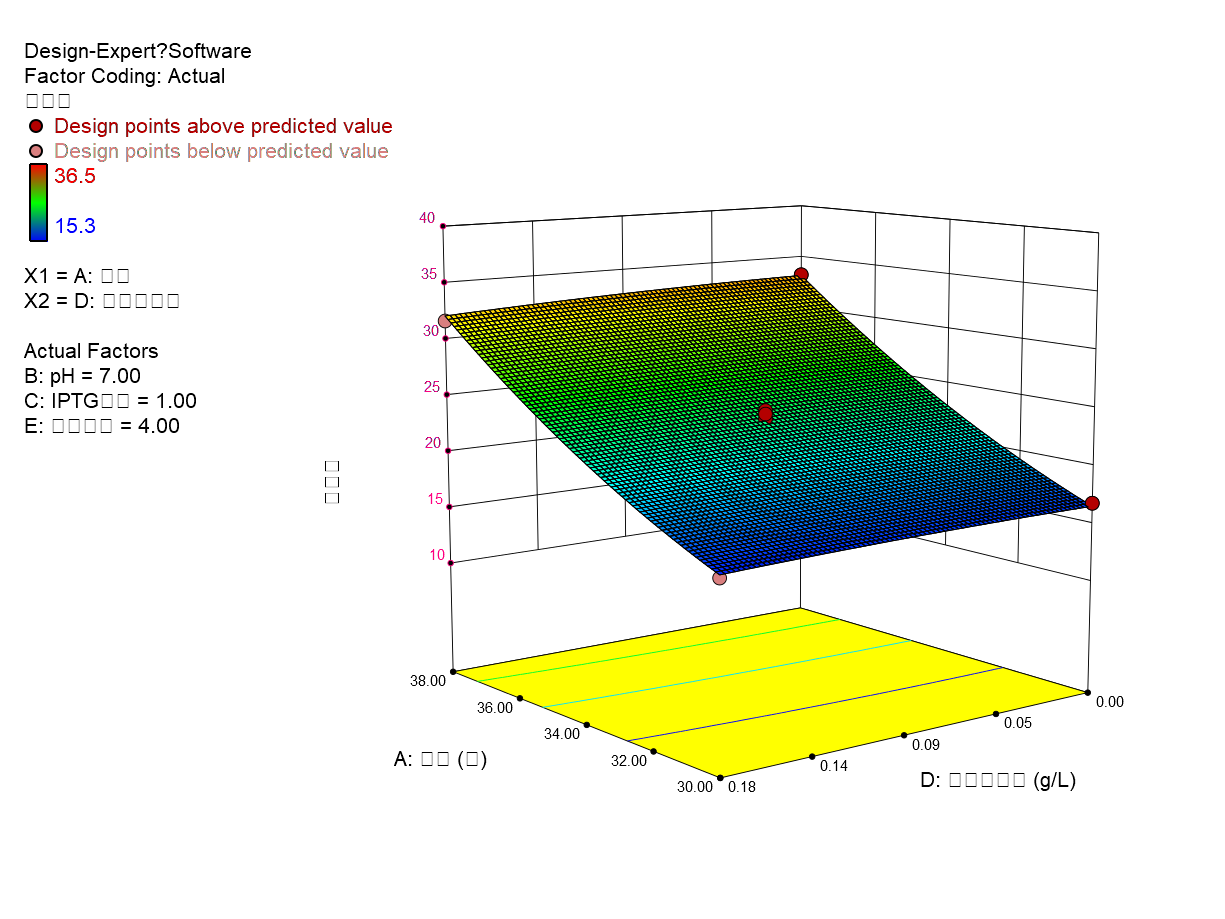

Supplement: Supplementary file 1 [file DataSheet3.ZIP › Figure 3/Fig 3b3.png]

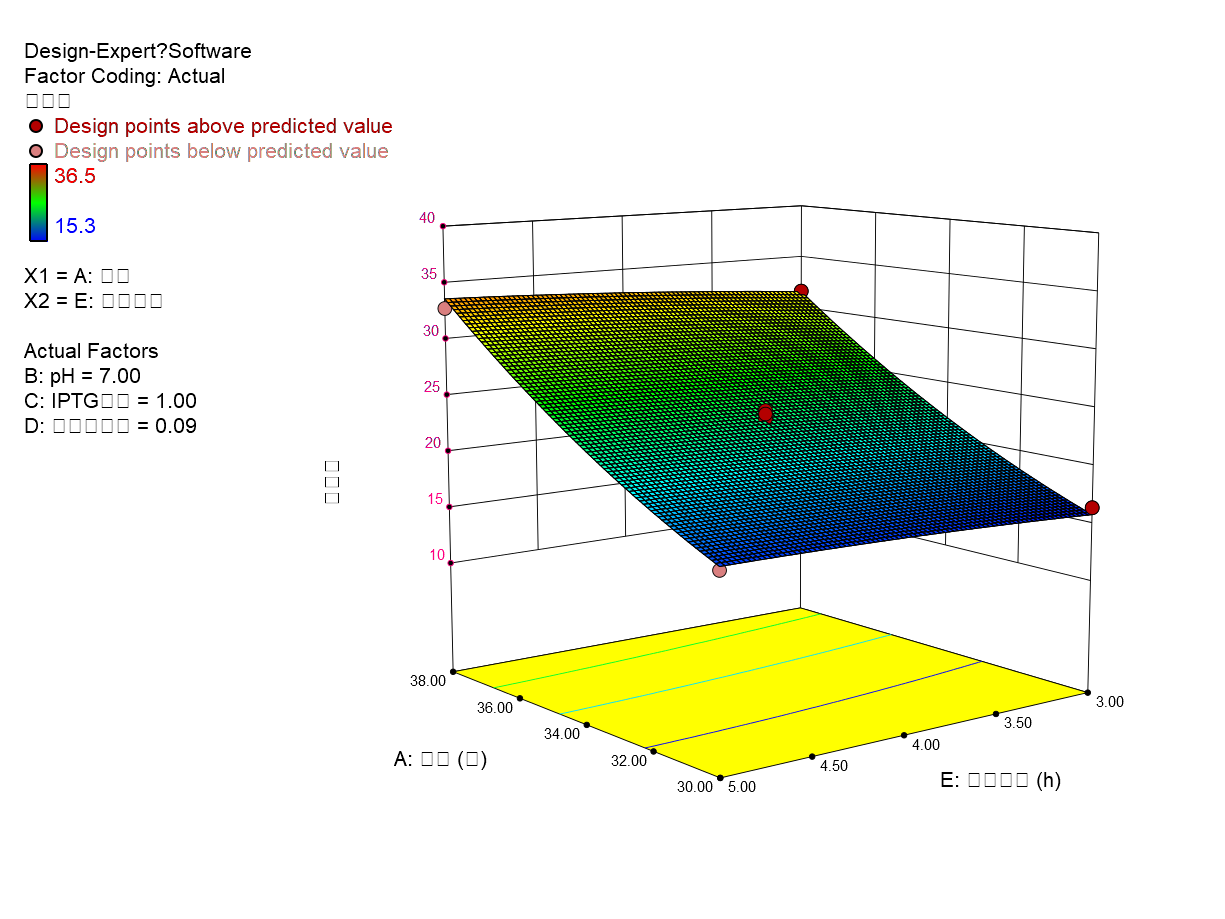

Supplement: Supplementary file 1 [file DataSheet3.ZIP › Figure 3/Fig 3b4.png]

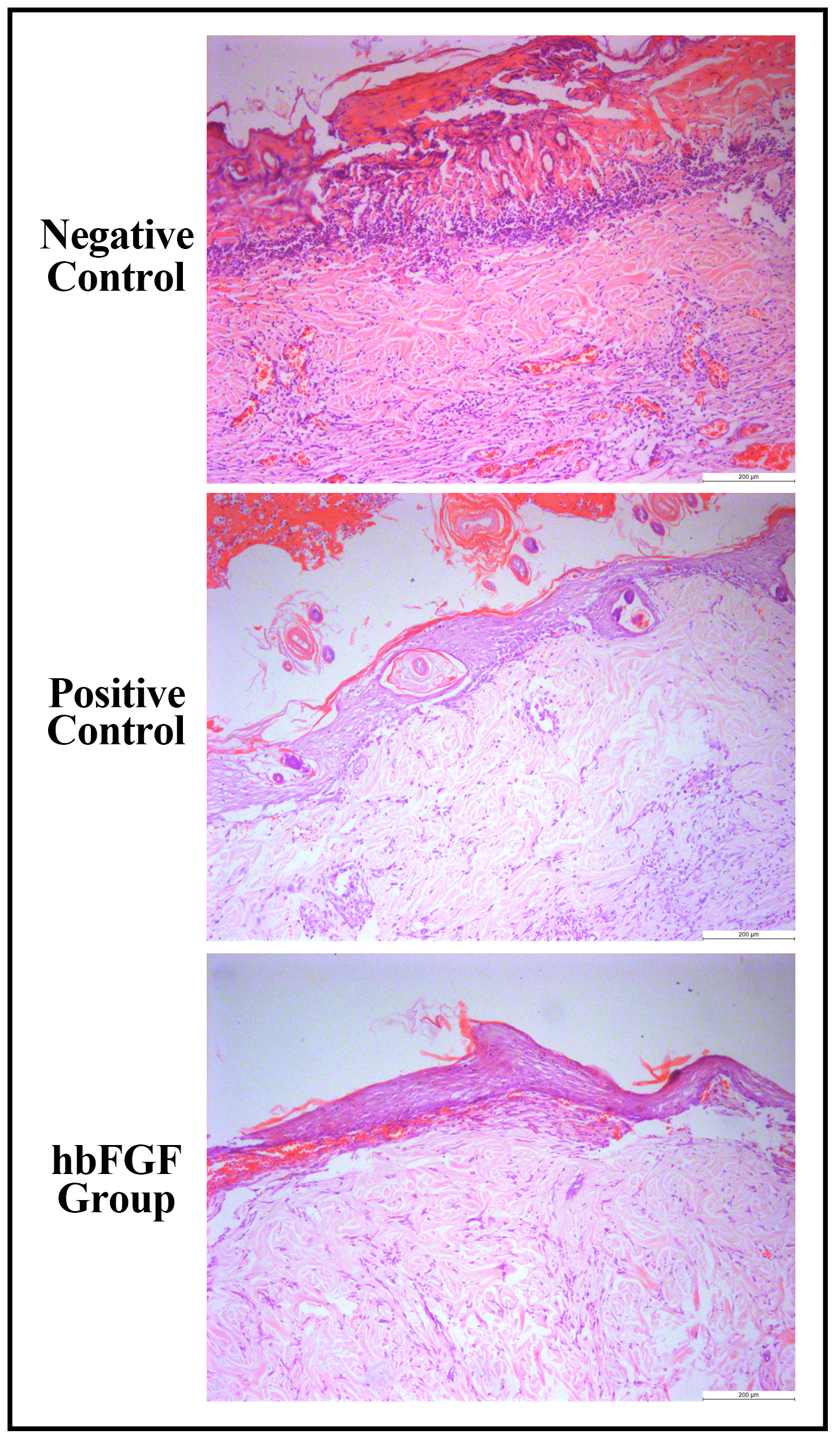

Supplement: Supplementary file 2 [file DataSheet11.ZIP › Supplementary Figure 5/Figure S5.tif]

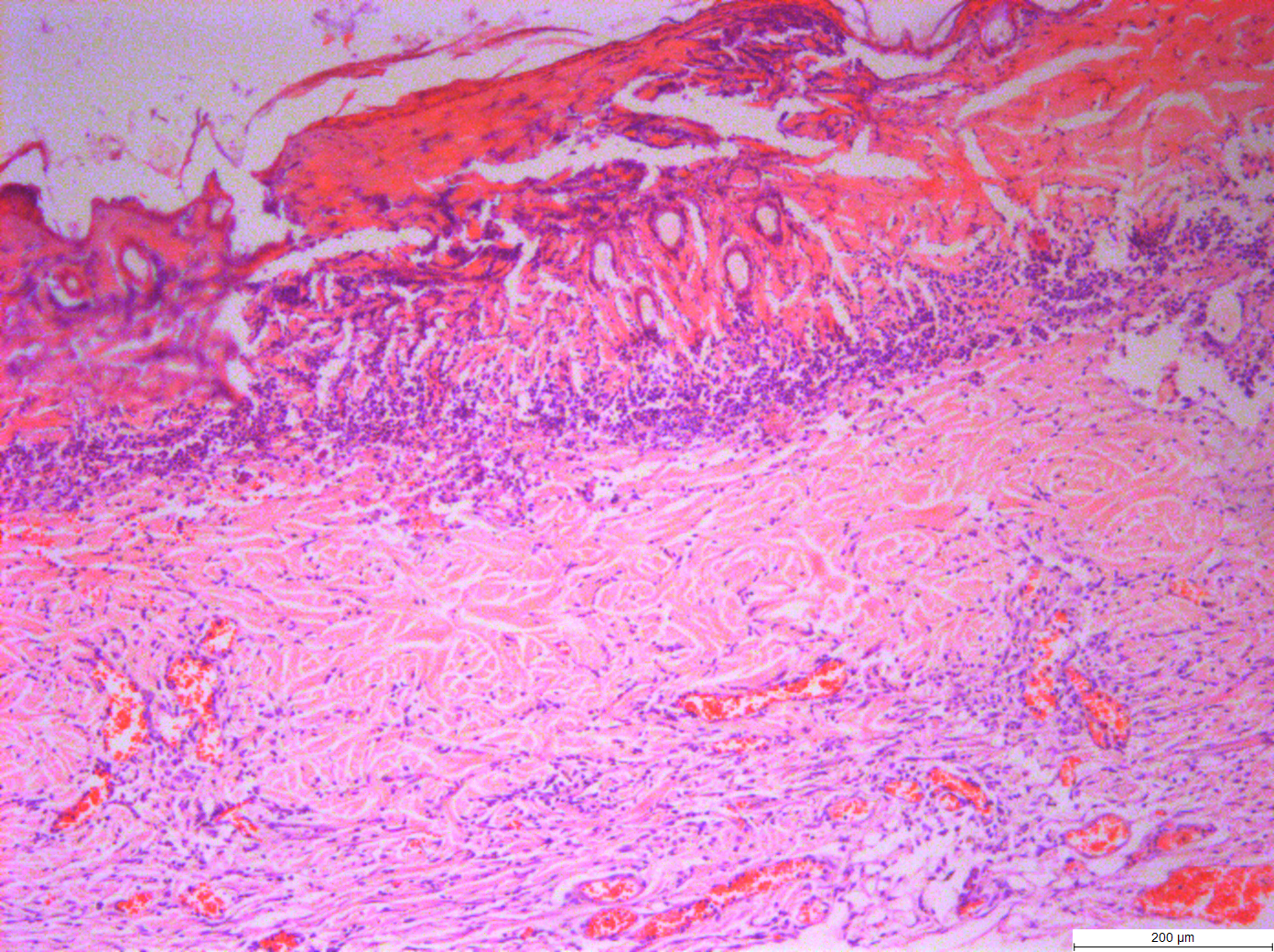

Supplement: Supplementary file 2 [file DataSheet11.ZIP › Supplementary Figure 5/Negative.tif]

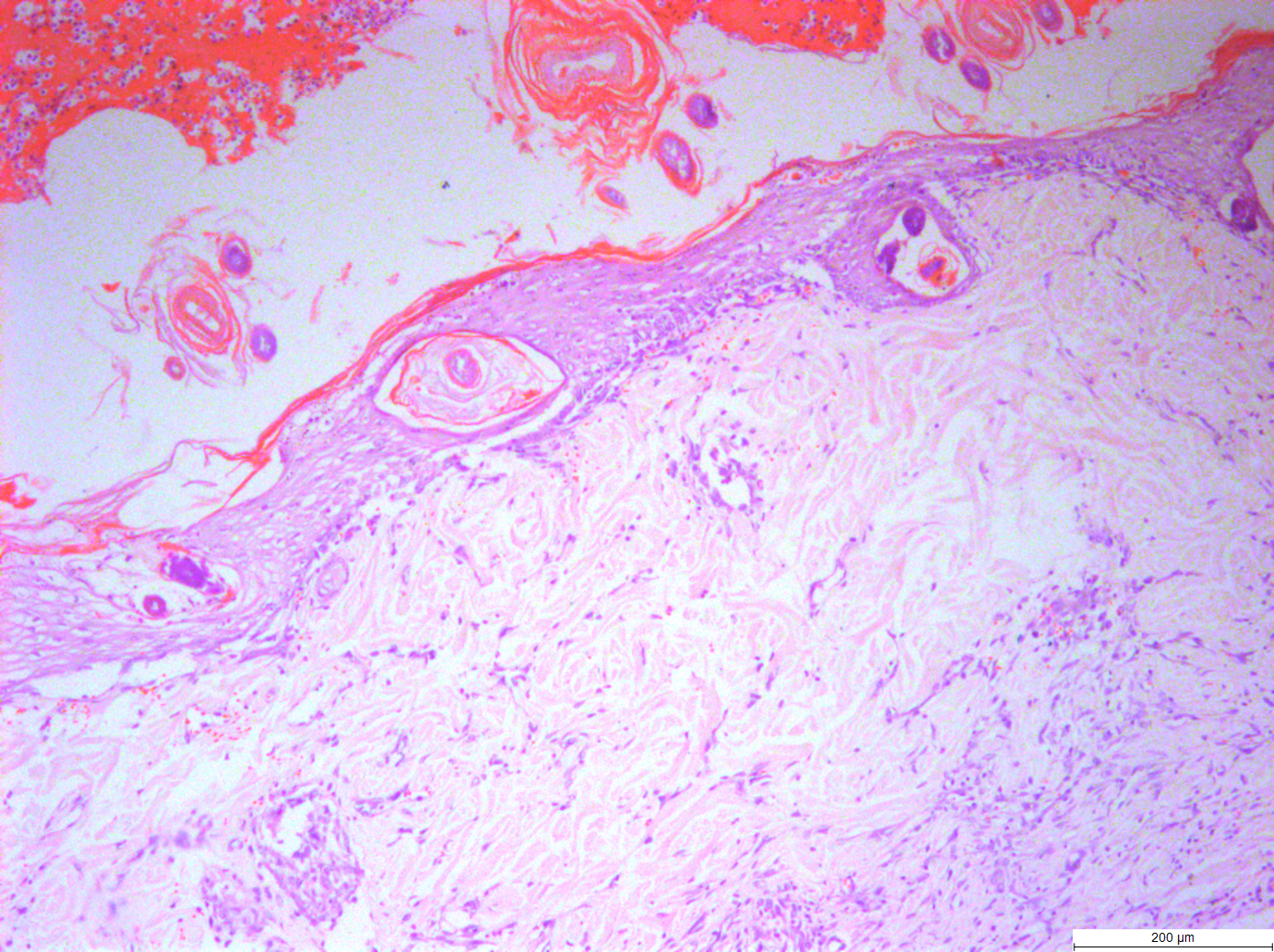

Supplement: Supplementary file 2 [file DataSheet11.ZIP › Supplementary Figure 5/Positive.tif]

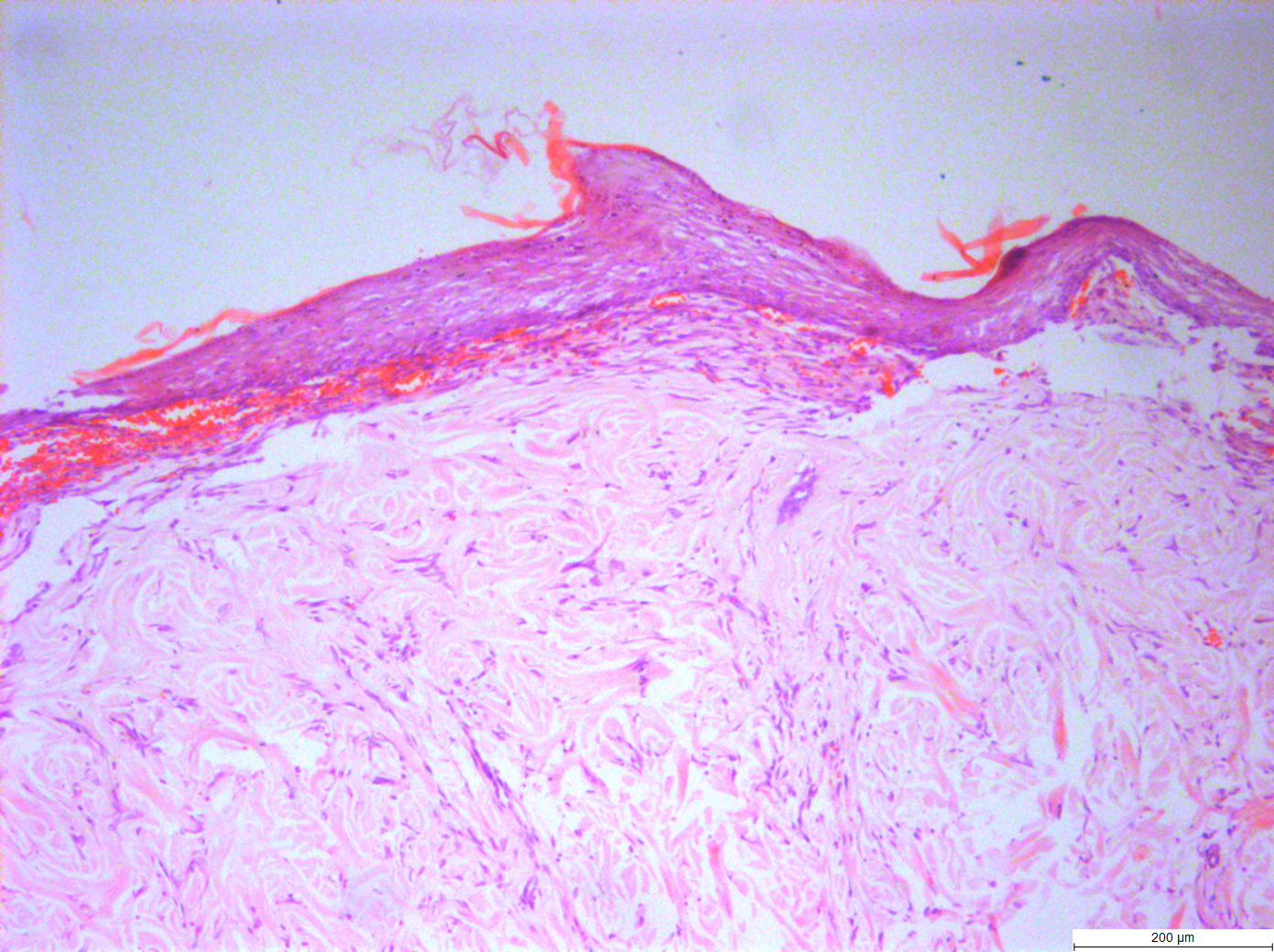

Supplement: Supplementary file 2 [file DataSheet11.ZIP › Supplementary Figure 5/hbFGF.tif]

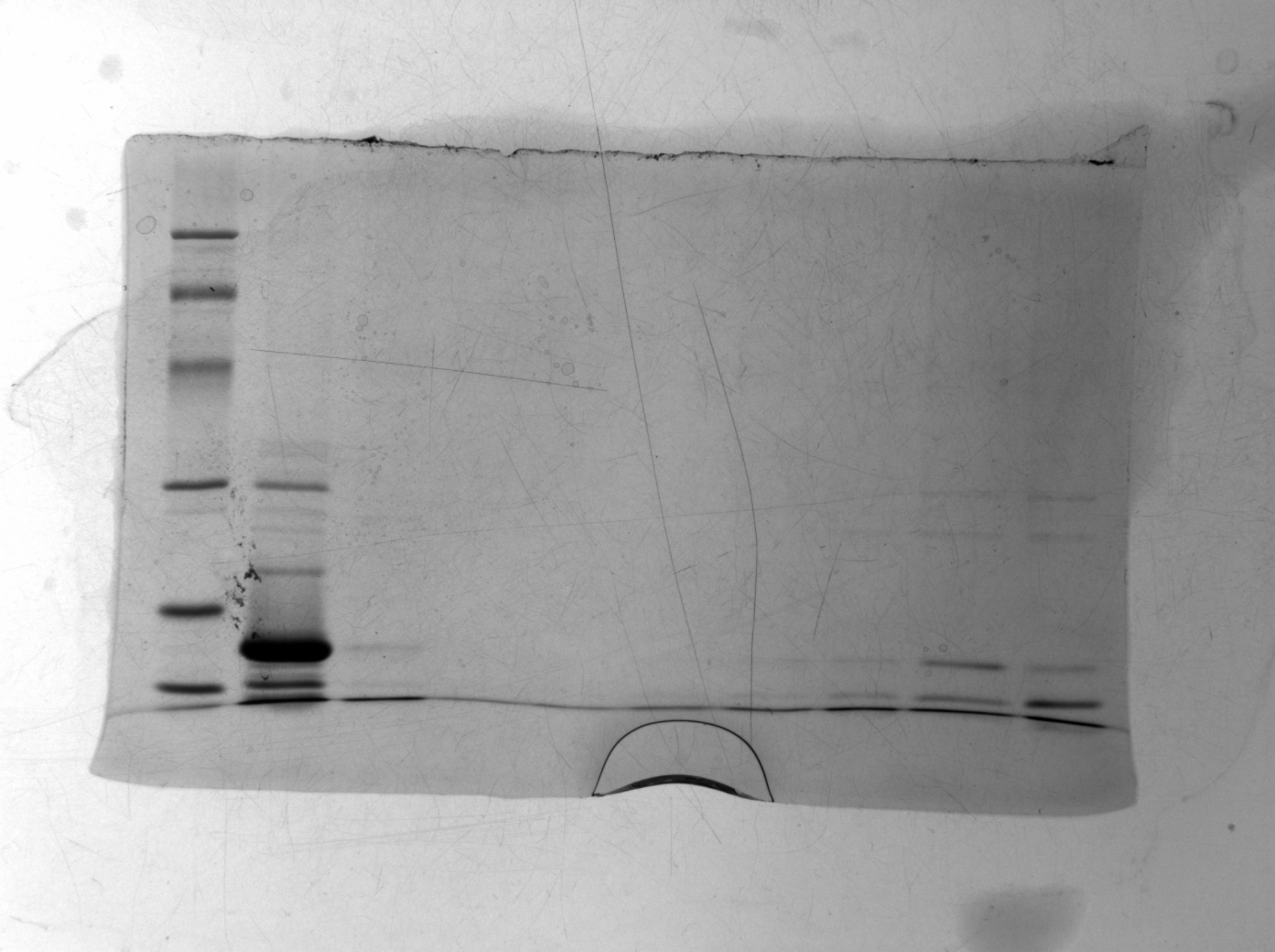

Supplement: Supplementary file 3 [file DataSheet8.ZIP › Supplementary Figure 2/Fig S2A1.tif]

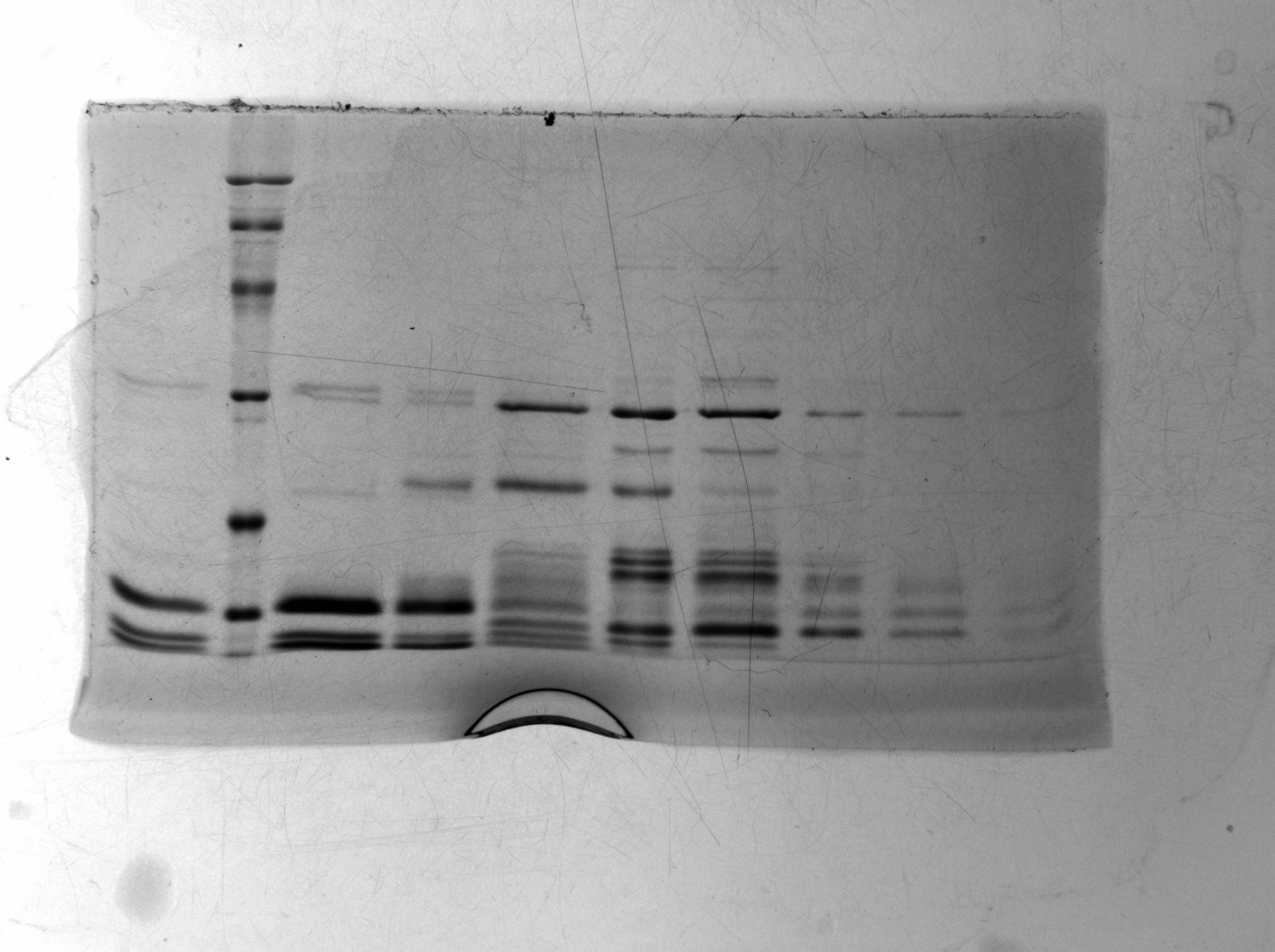

Supplement: Supplementary file 3 [file DataSheet8.ZIP › Supplementary Figure 2/Fig S2A2 .tif]

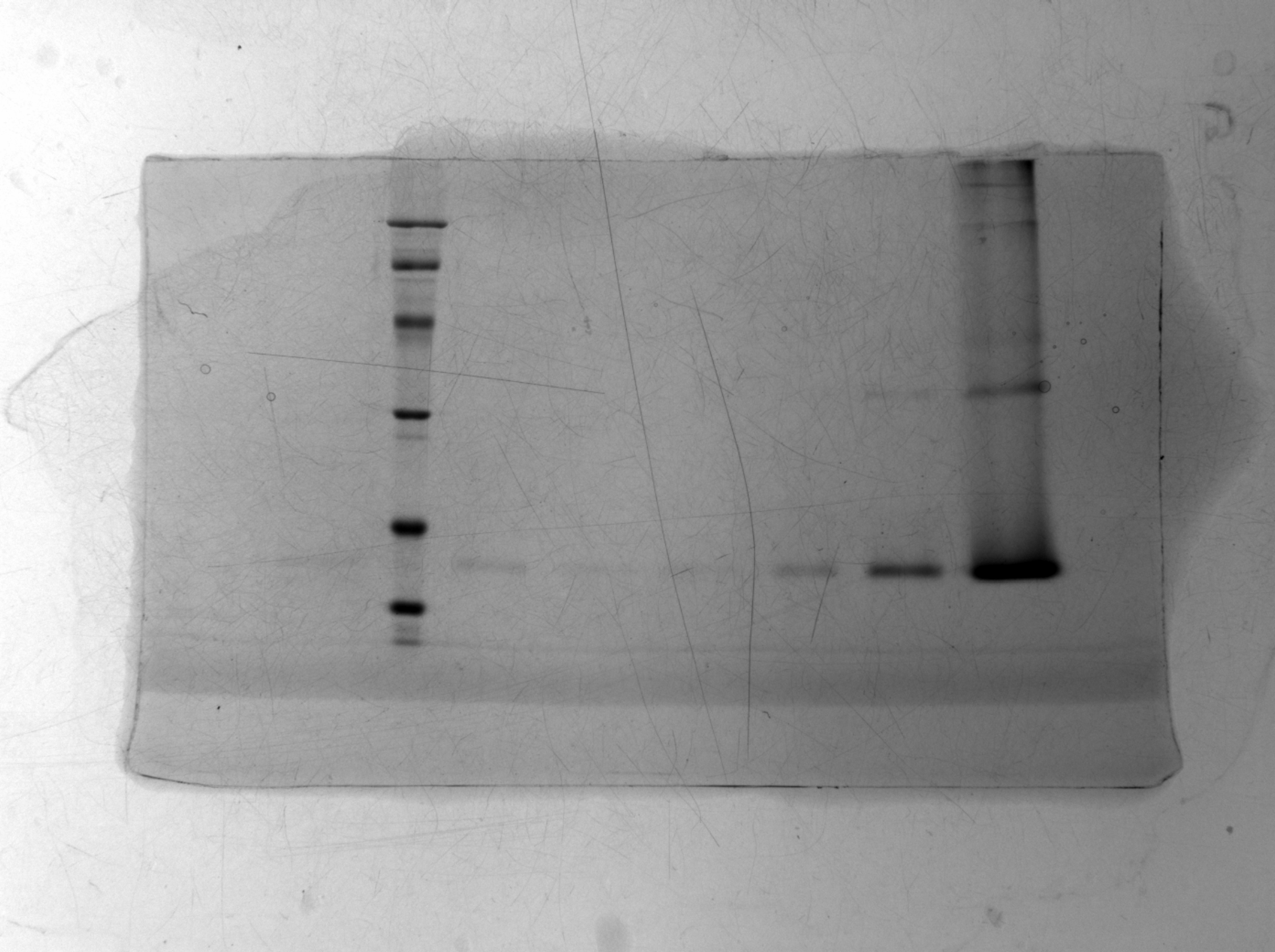

Supplement: Supplementary file 3 [file DataSheet8.ZIP › Supplementary Figure 2/Fig S2A3.tif]

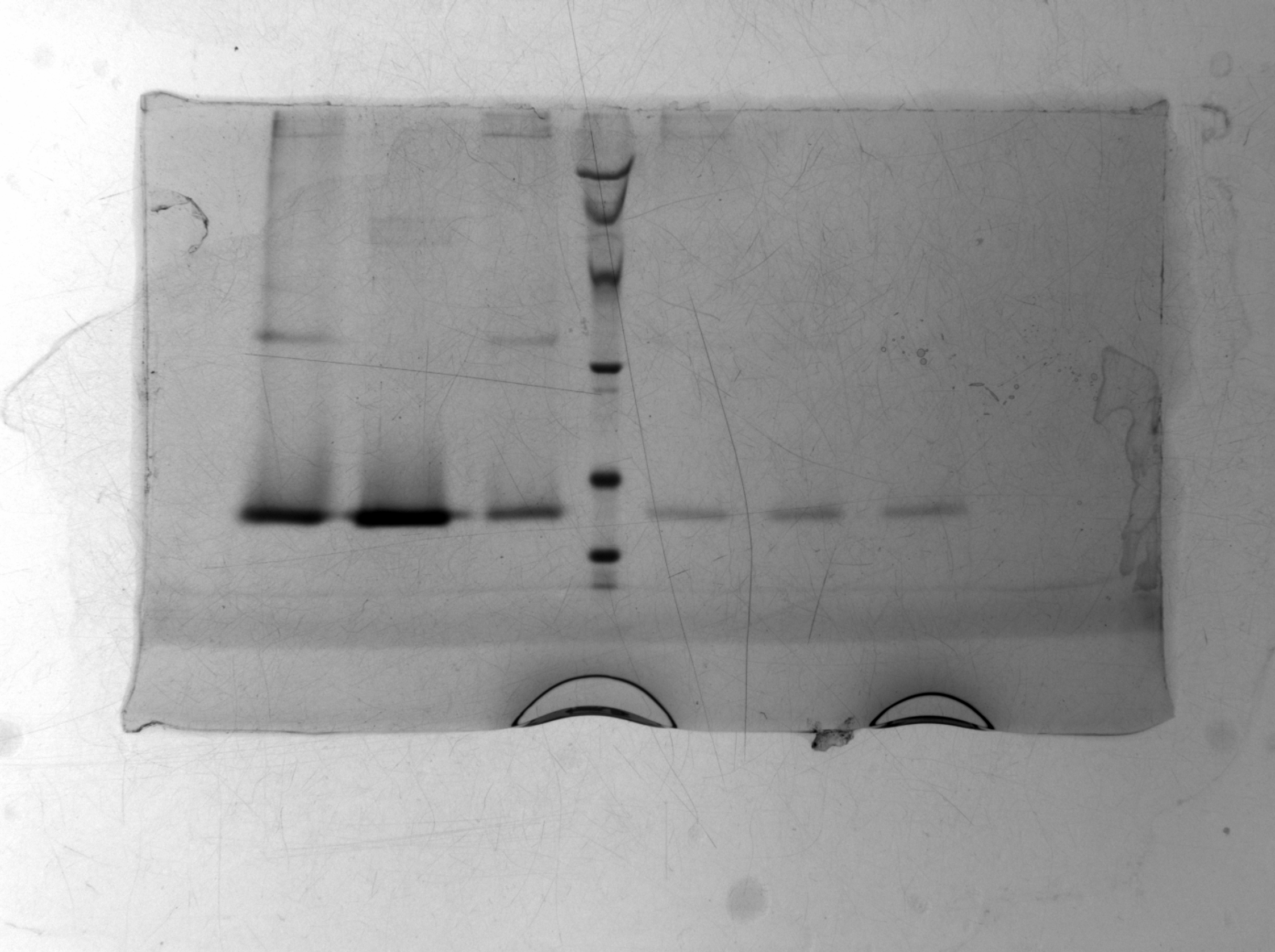

Supplement: Supplementary file 3 [file DataSheet8.ZIP › Supplementary Figure 2/Fig S2A4.tif]

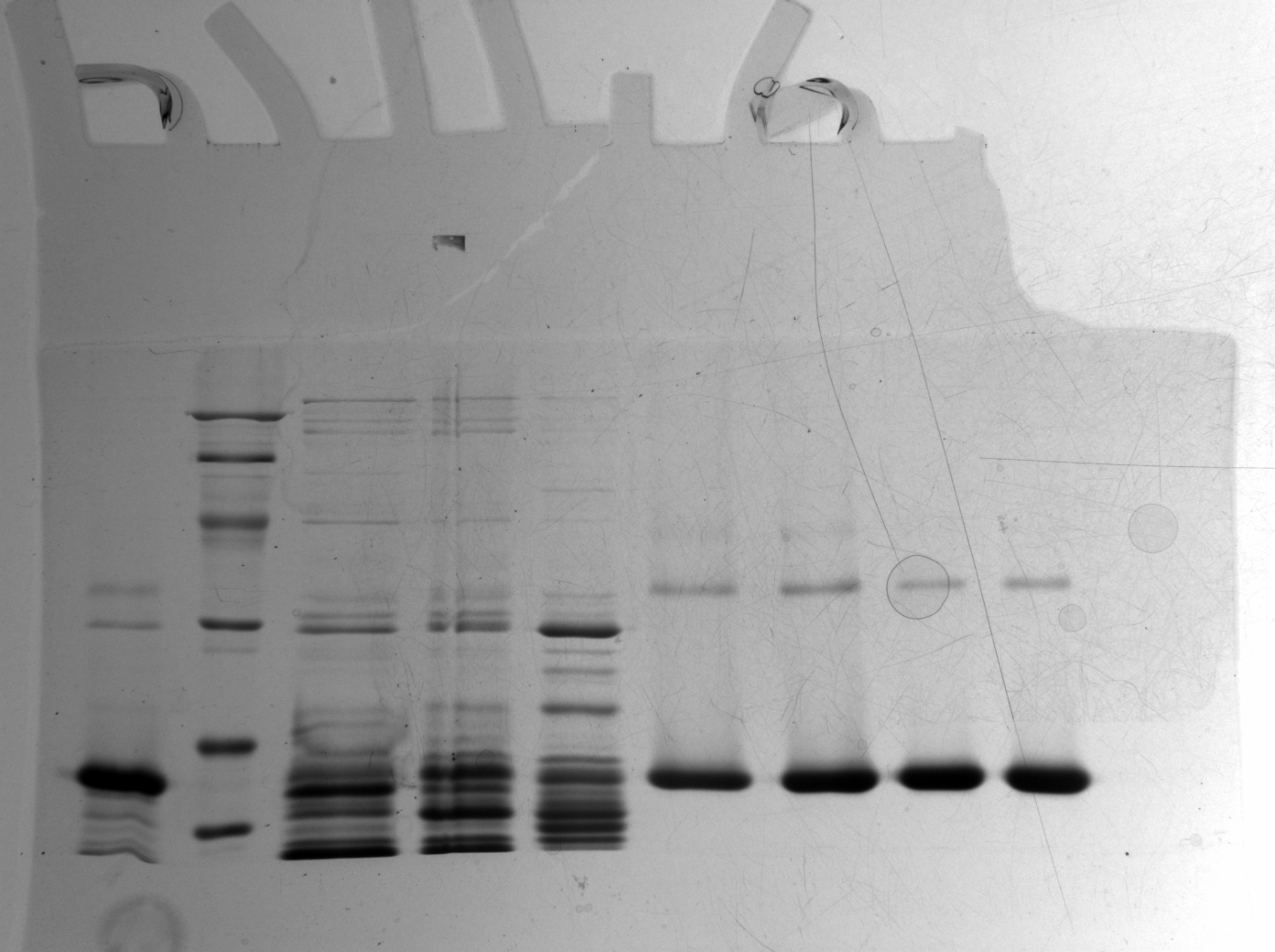

Supplement: Supplementary file 3 [file DataSheet8.ZIP › Supplementary Figure 2/Fig S2B.tif]

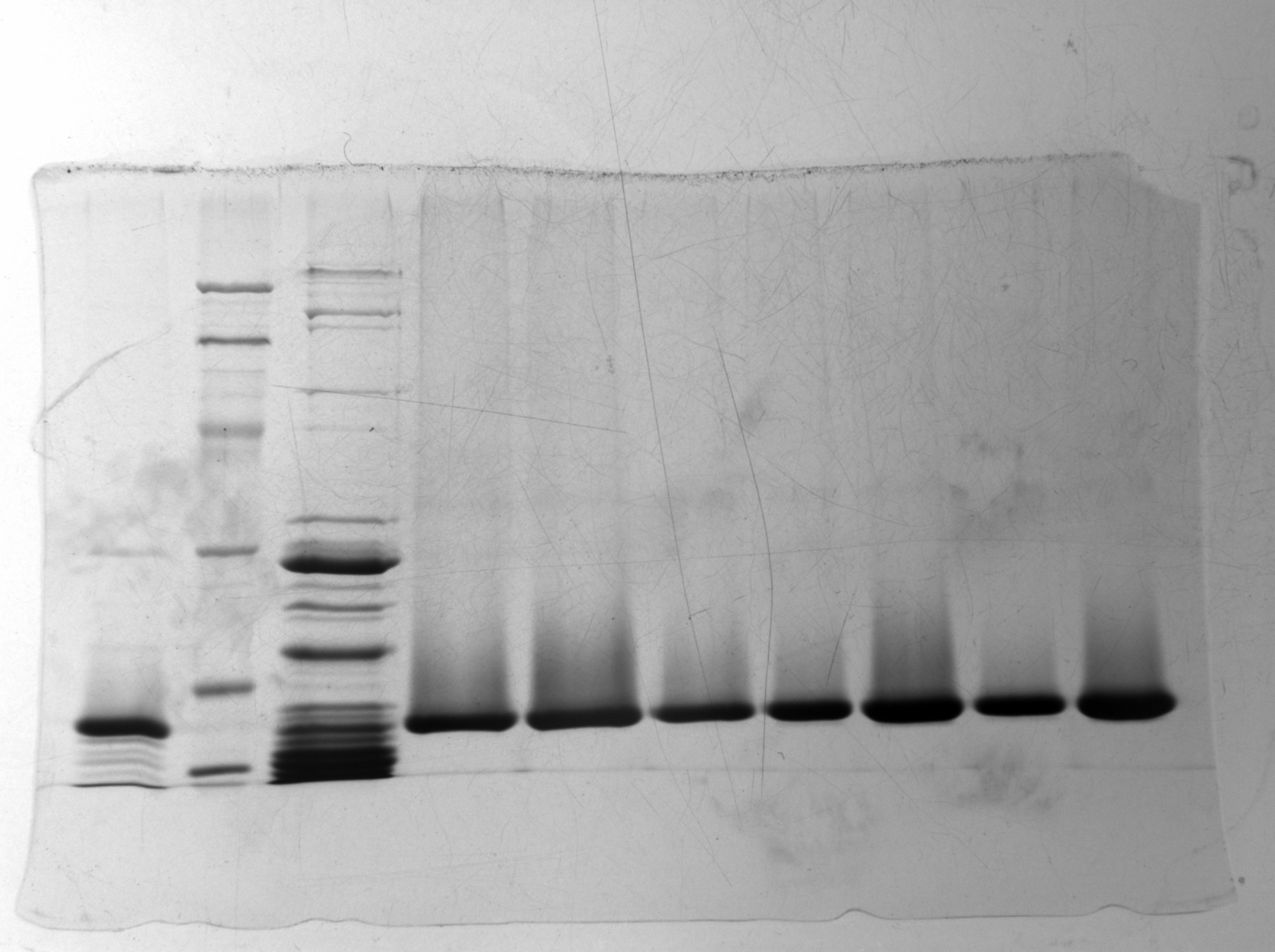

Supplement: Supplementary file 3 [file DataSheet8.ZIP › Supplementary Figure 2/Fig S2C.tif]

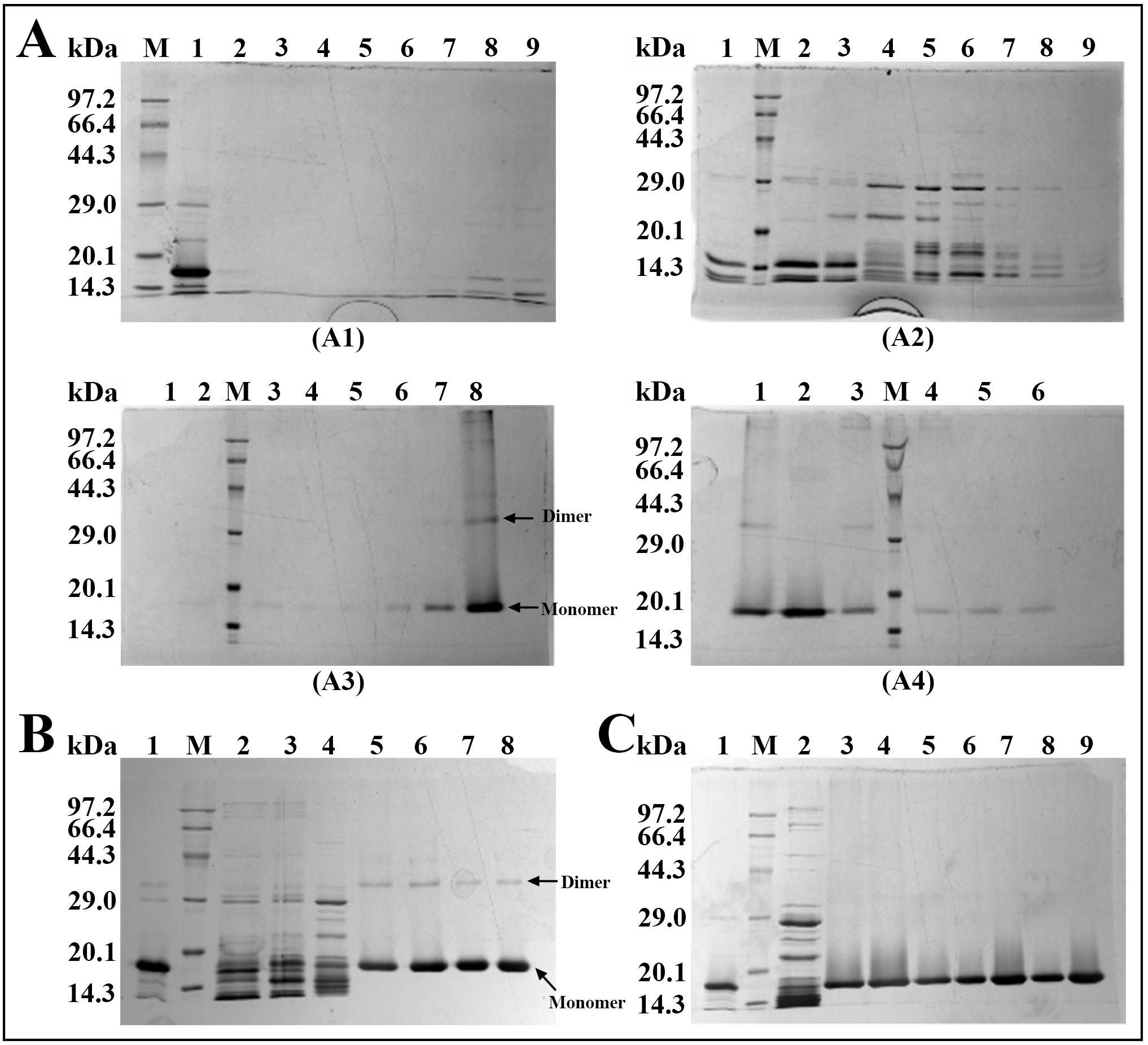

Supplement: Supplementary file 3 [file DataSheet8.ZIP › Supplementary Figure 2/Figure S2.tif]

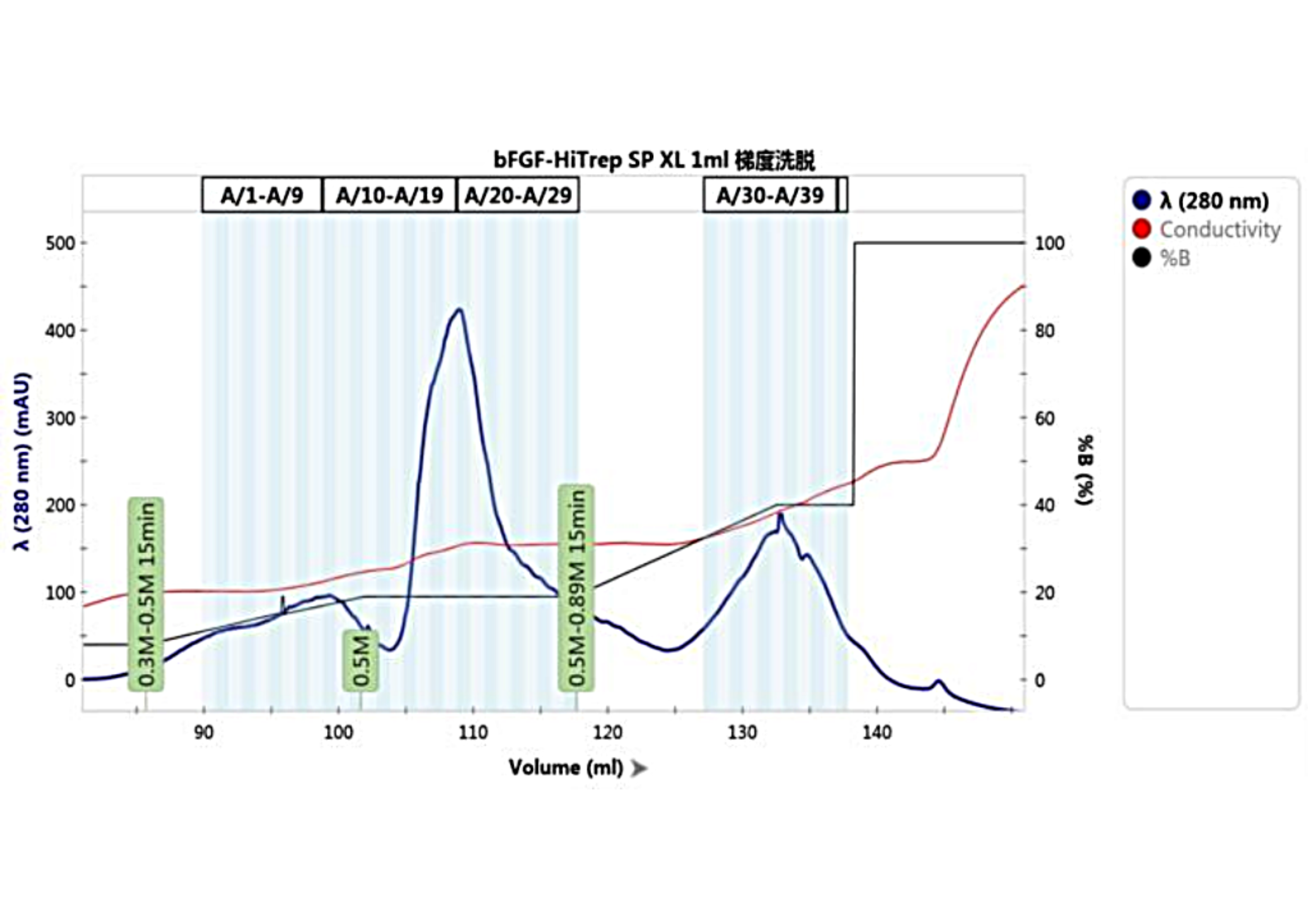

Supplement: Supplementary file 4 [file DataSheet9.ZIP › Supplementary Figure 3/Fig S3a1.png]

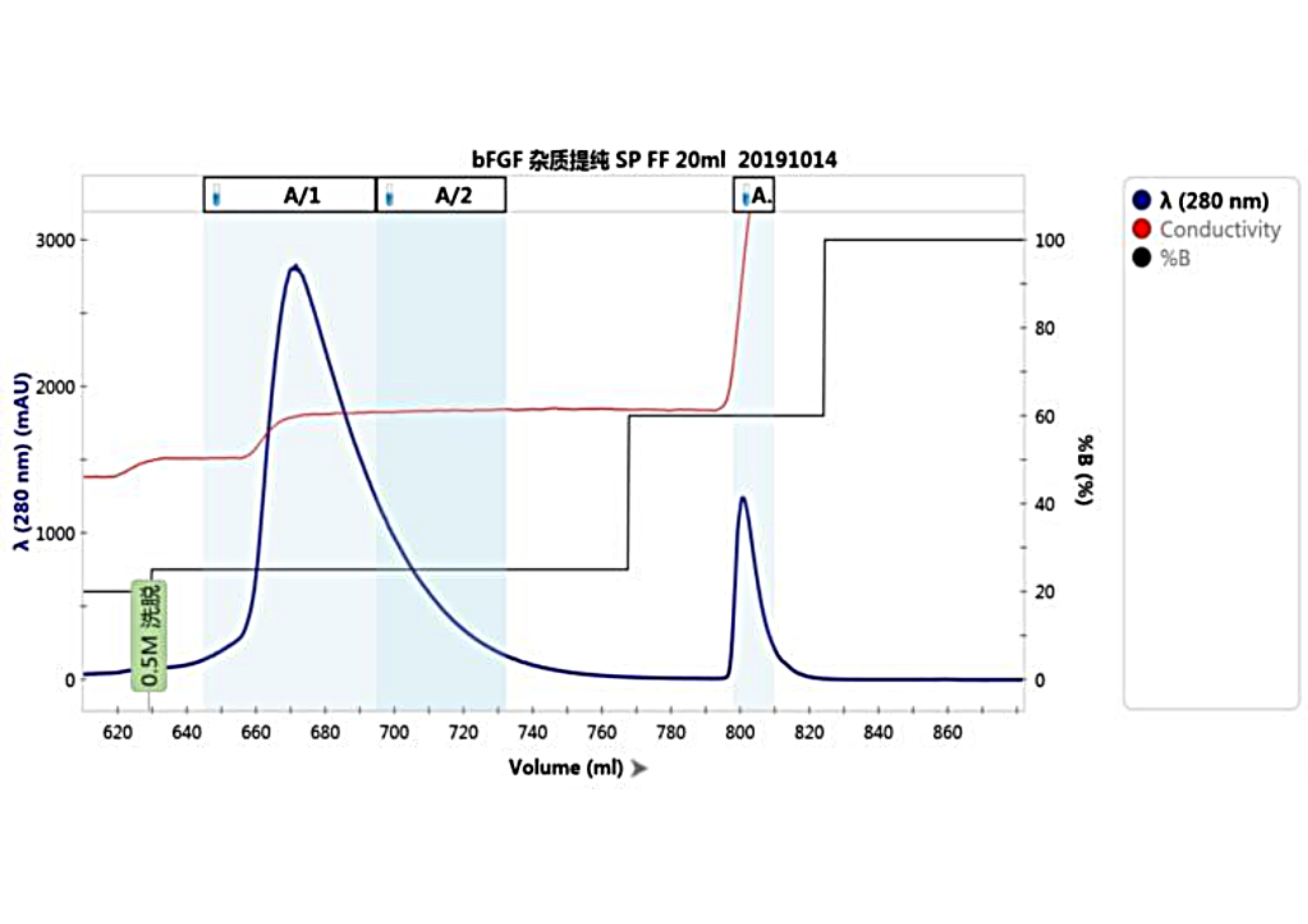

Supplement: Supplementary file 4 [file DataSheet9.ZIP › Supplementary Figure 3/Fig S3b1.png]

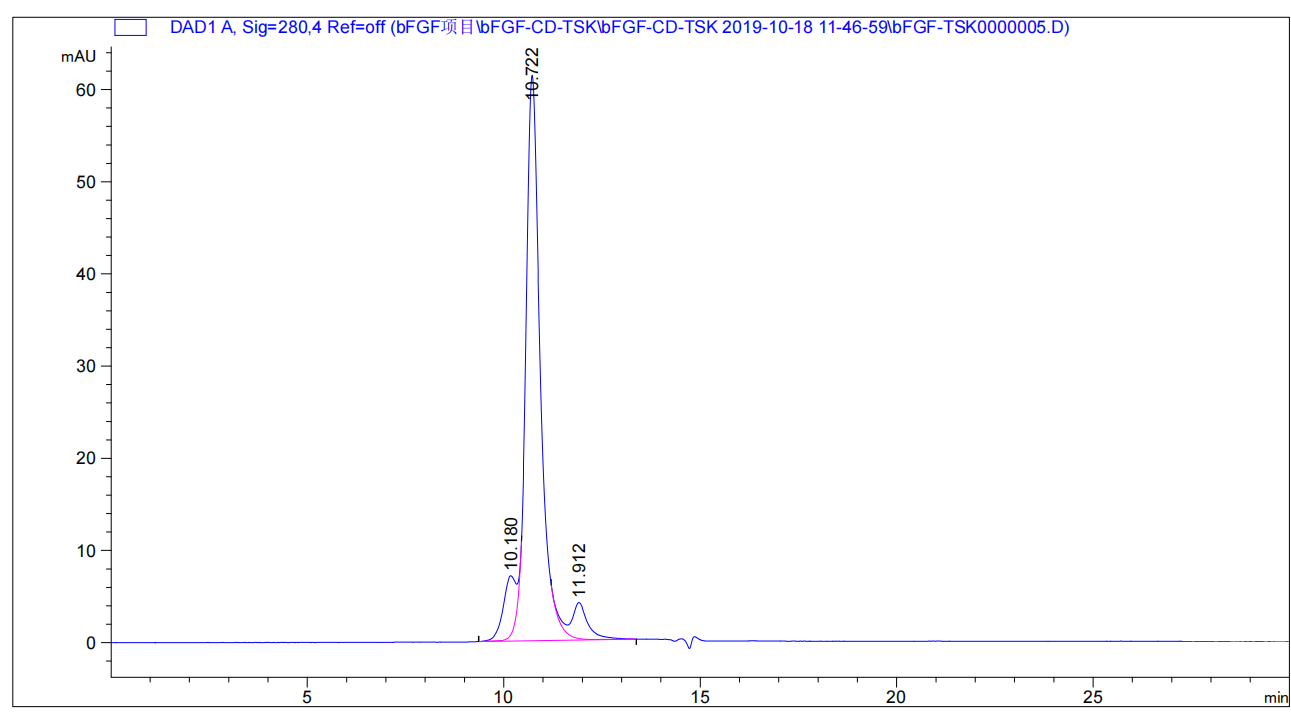

Supplement: Supplementary file 4 [file DataSheet9.ZIP › Supplementary Figure 3/Fig S3b3.tif]

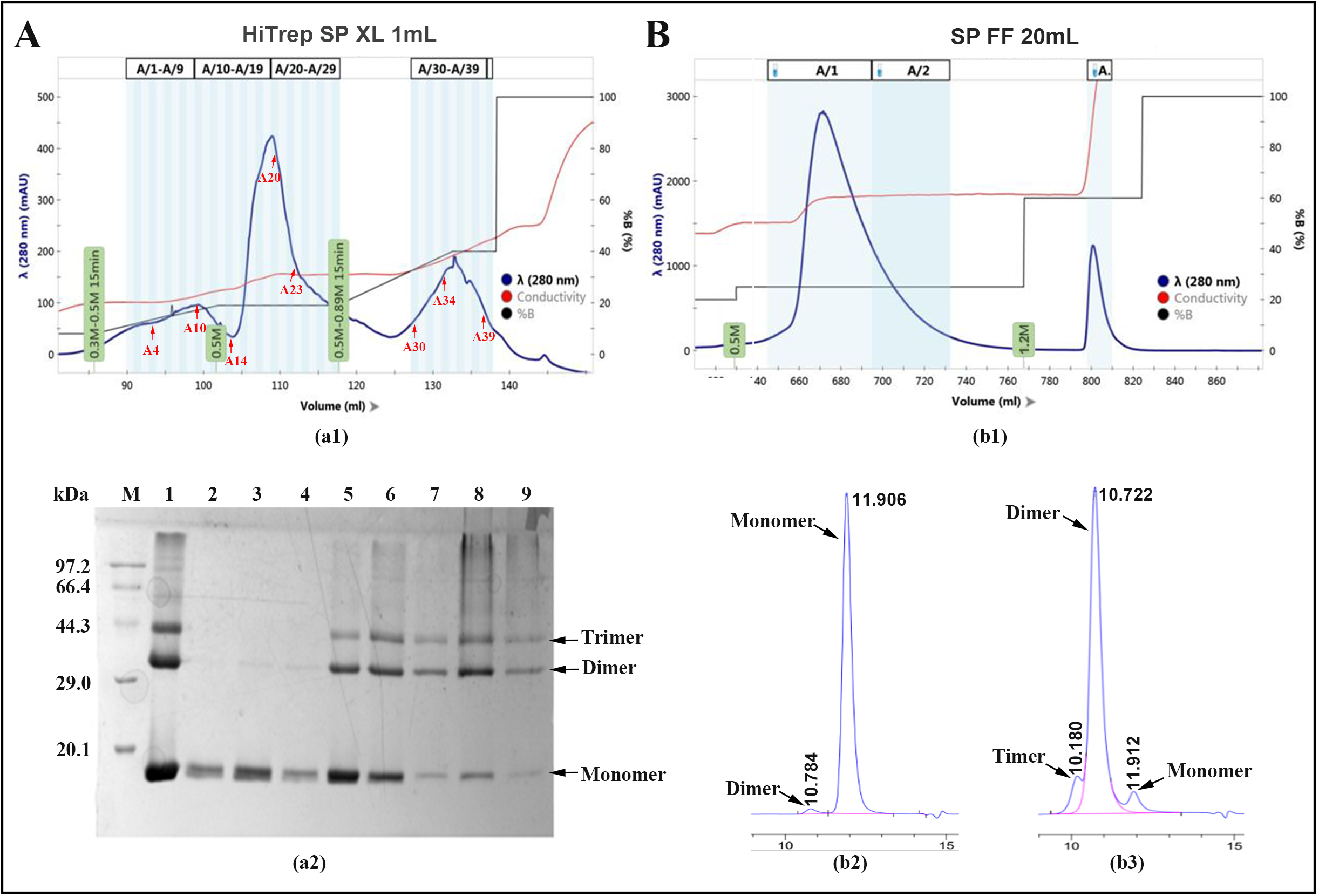

Supplement: Supplementary file 4 [file DataSheet9.ZIP › Supplementary Figure 3/Figure S3.tif]

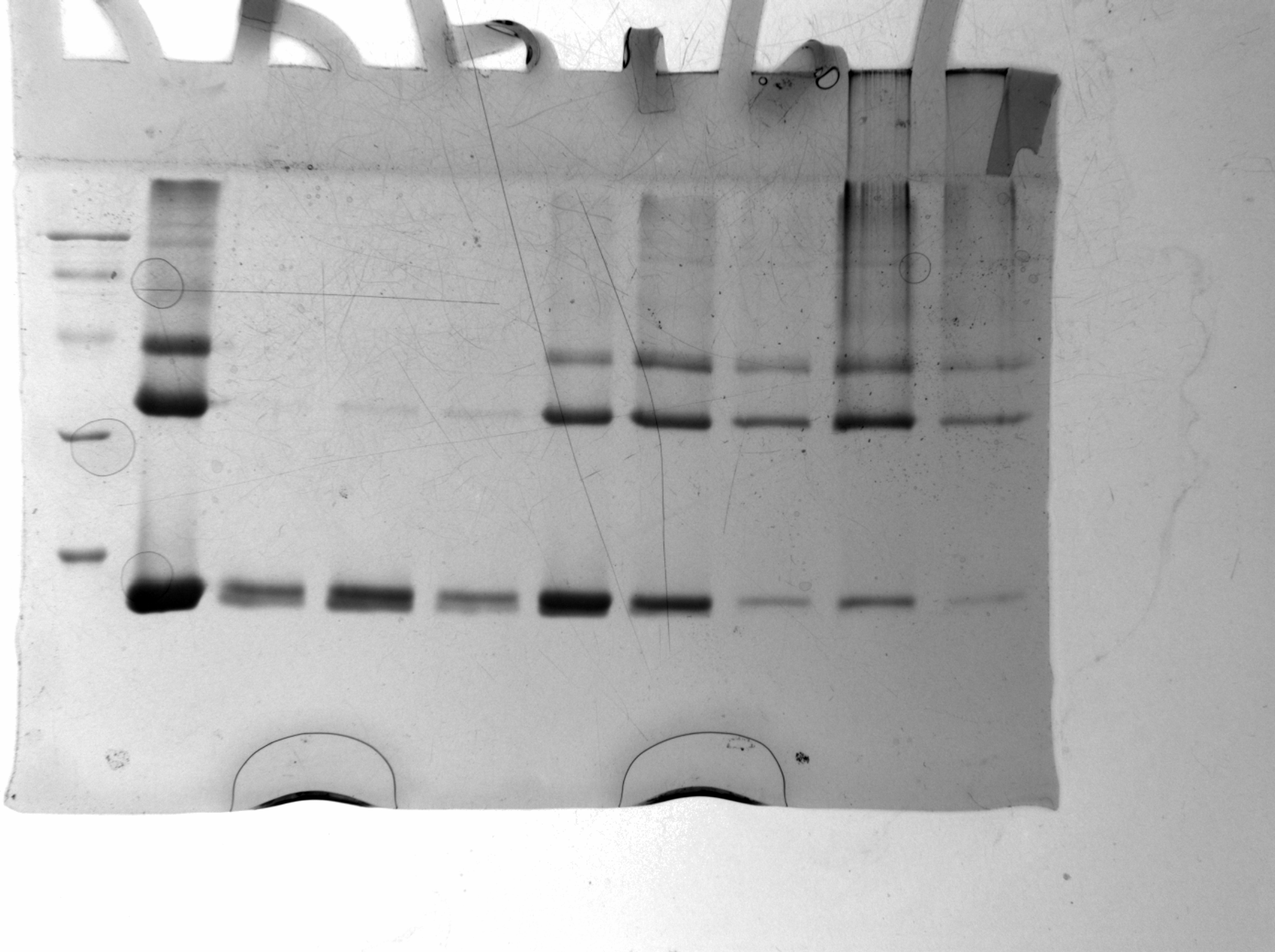

Supplement: Supplementary file 4 [file DataSheet9.ZIP › Supplementary Figure 3/fig S3a2.tif]

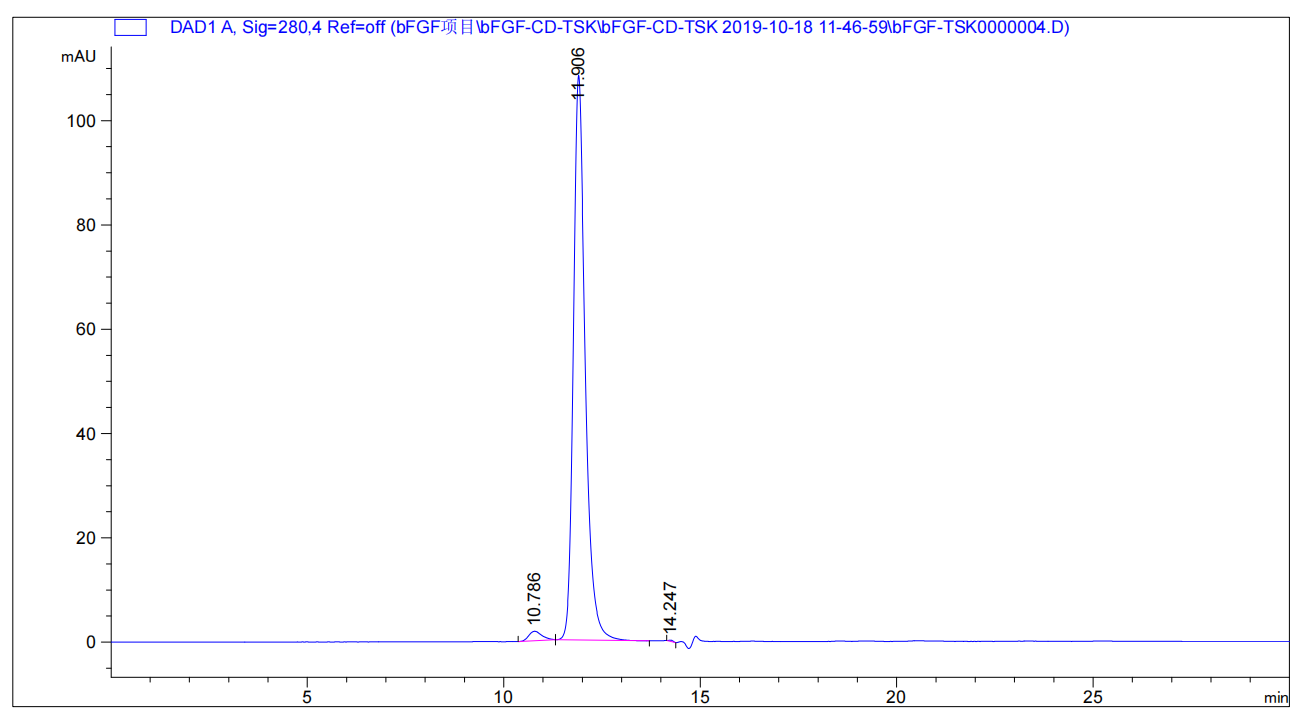

Supplement: Supplementary file 4 [file DataSheet9.ZIP › Supplementary Figure 3/fig S3b2.tif]

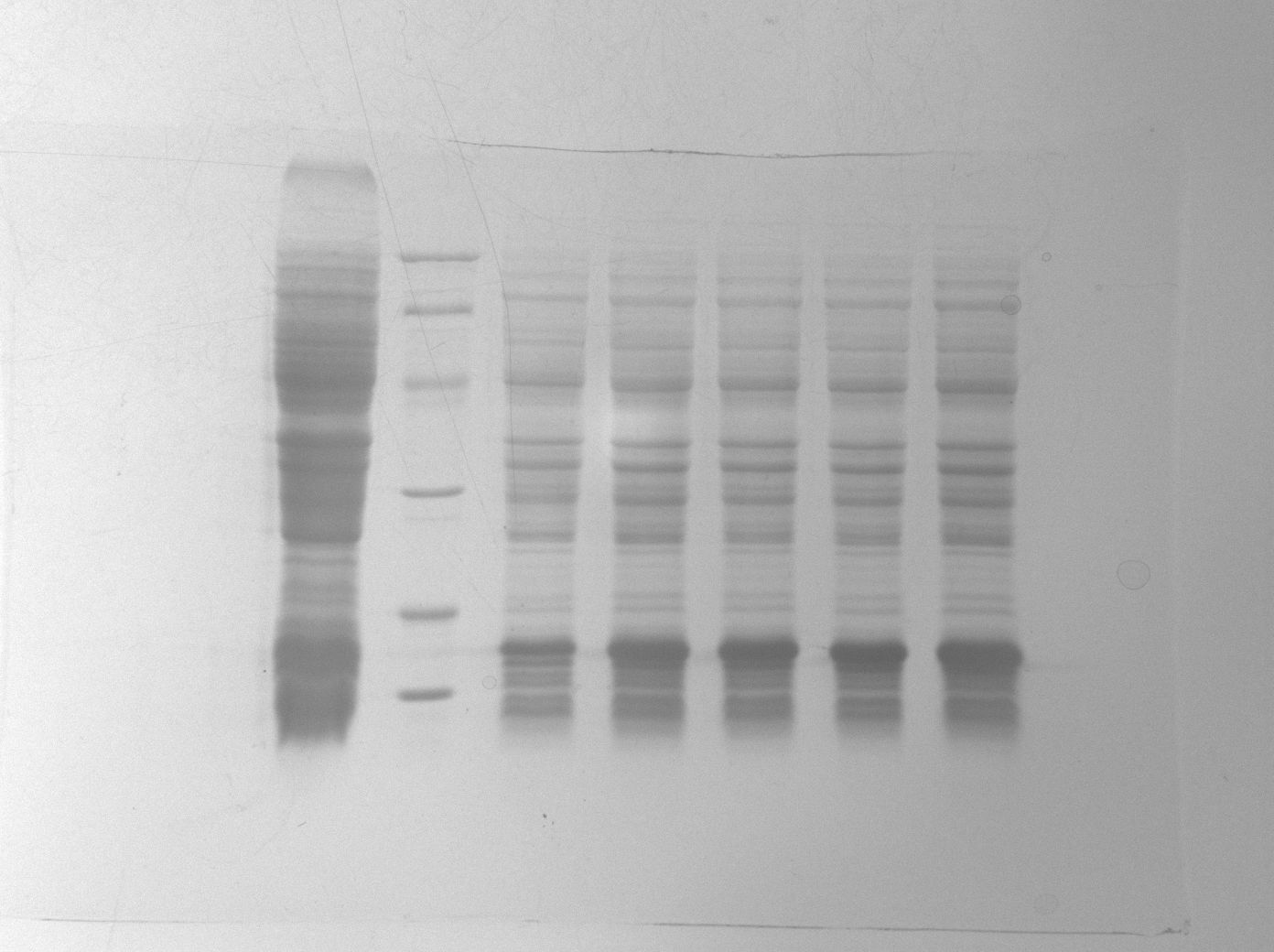

Supplement: Supplementary file 6 [file DataSheet4.ZIP › Figure 4/Fig 4B.tif]

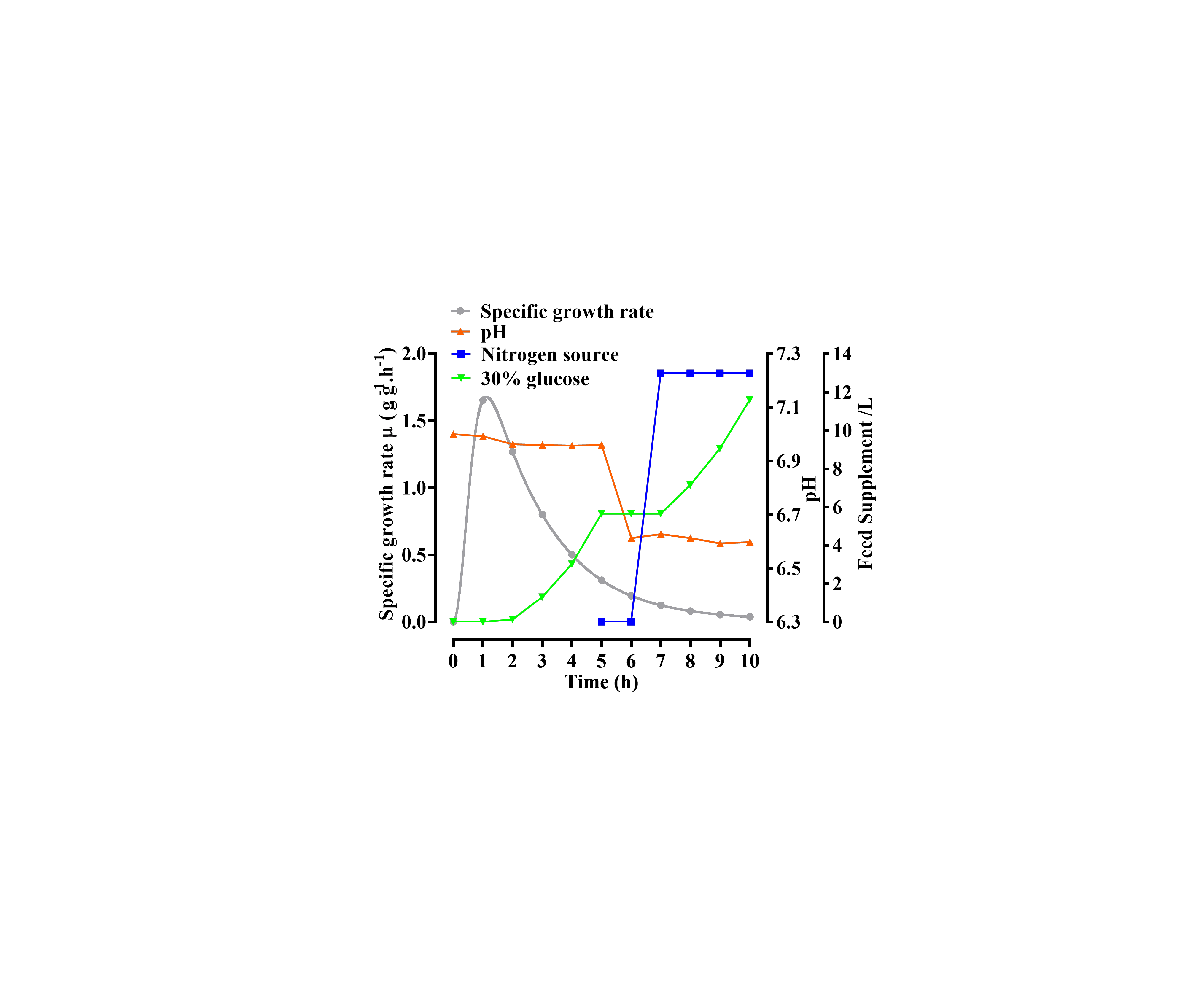

Supplement: Supplementary file 6 [file DataSheet4.ZIP › Figure 4/Fig 4D Merge.tif]

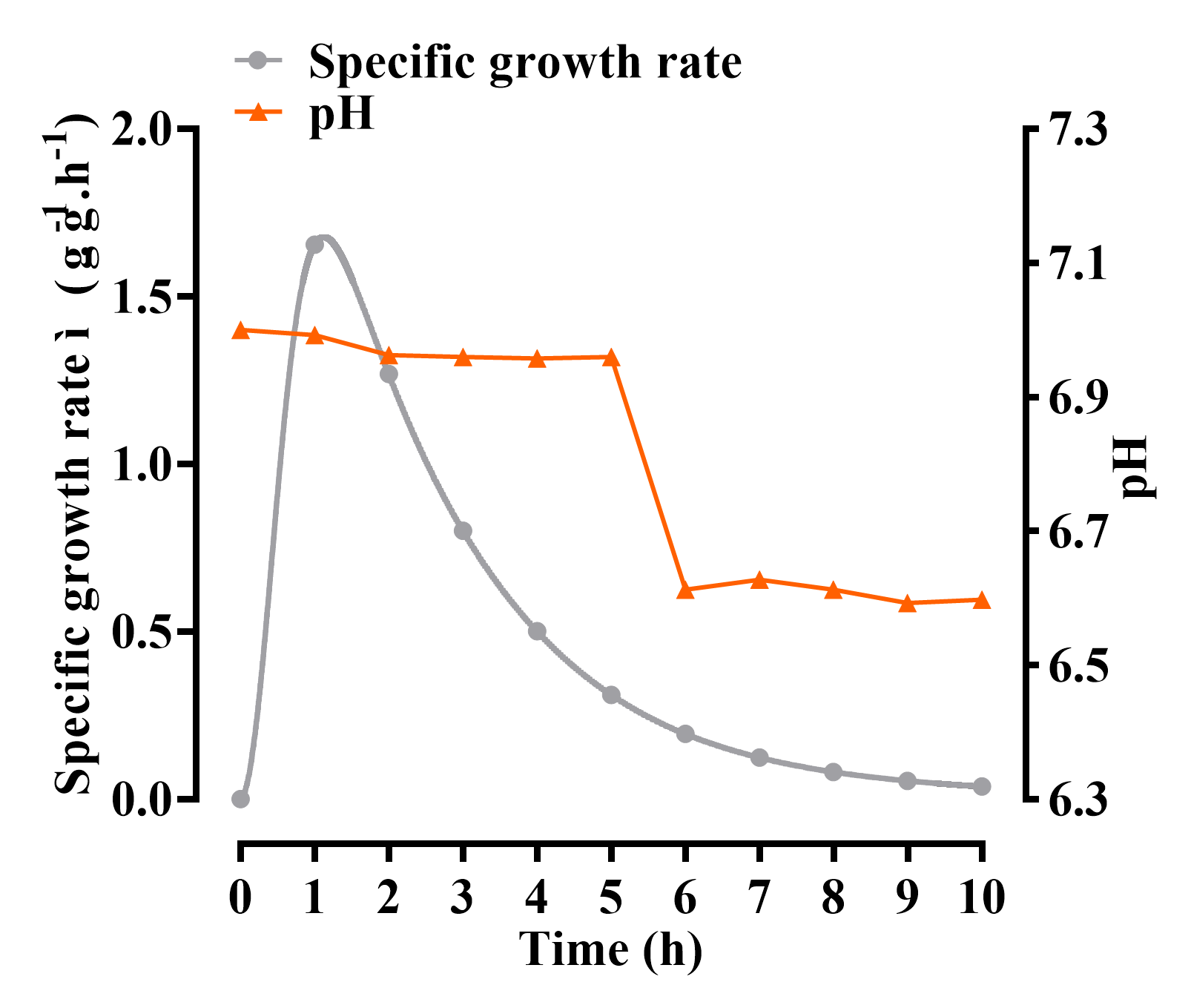

Supplement: Supplementary file 6 [file DataSheet4.ZIP › Figure 4/Fig 4D1.tif]

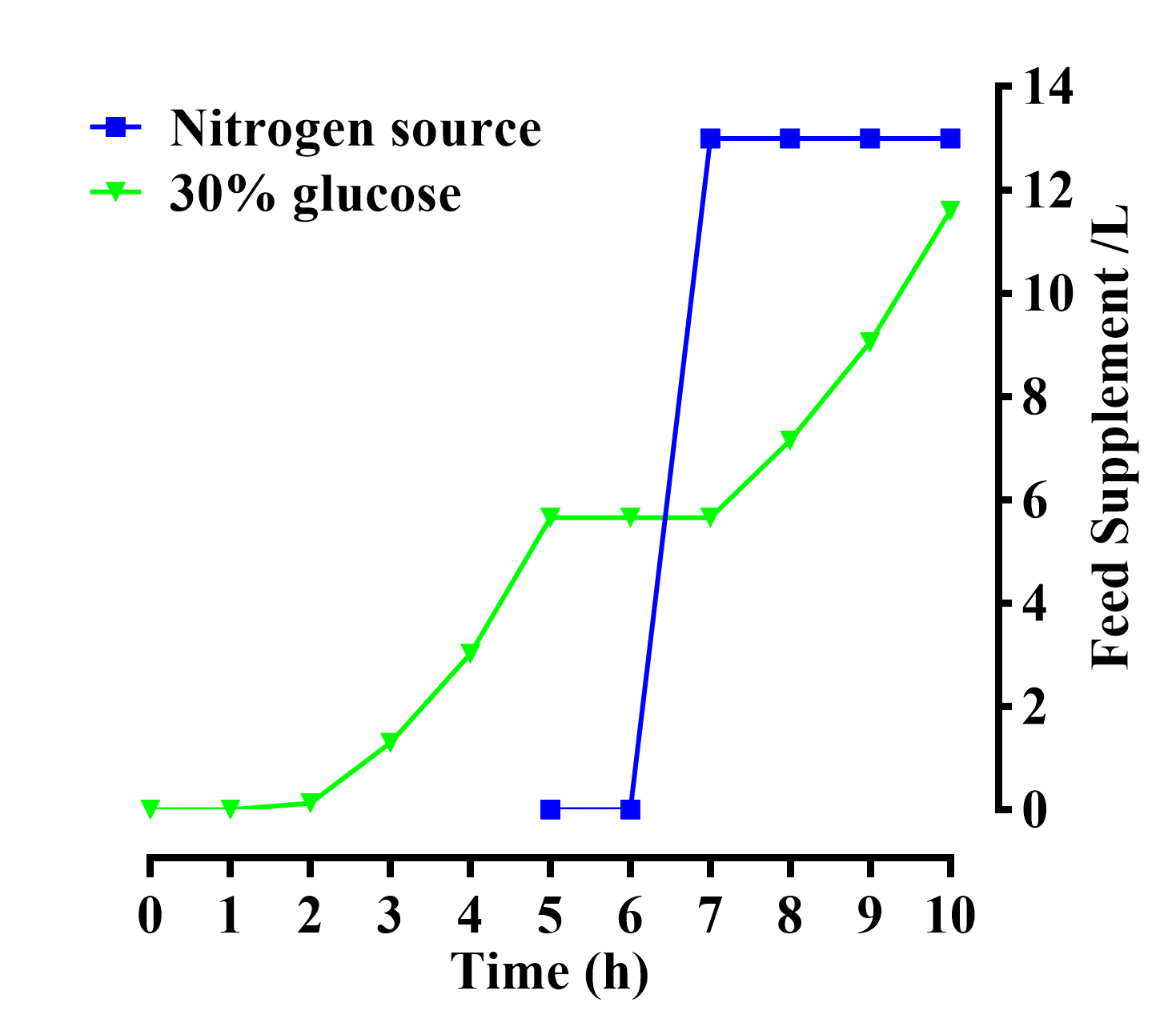

Supplement: Supplementary file 6 [file DataSheet4.ZIP › Figure 4/Fig 4D2.tif]

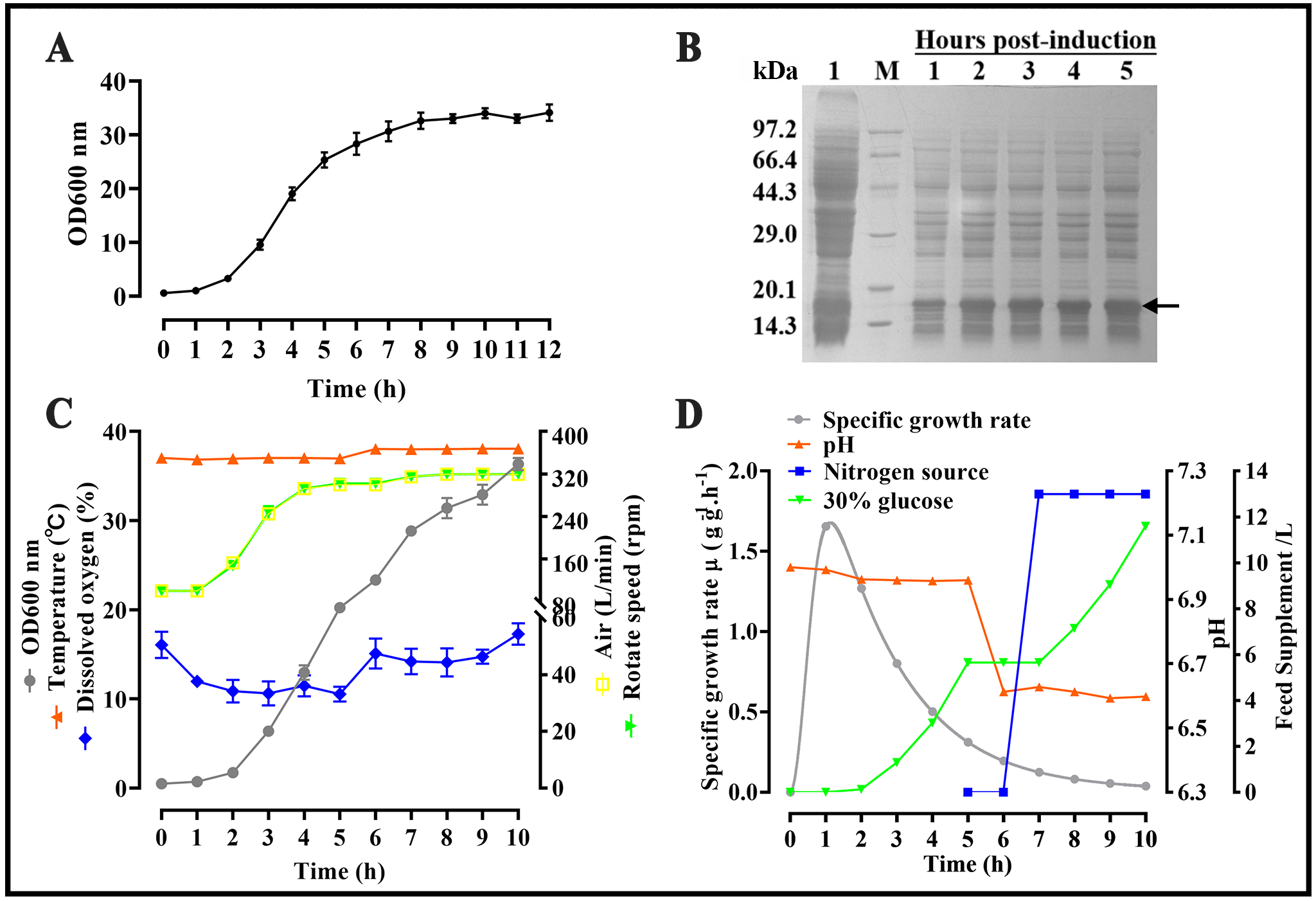

Supplement: Supplementary file 6 [file DataSheet4.ZIP › Figure 4/Figure 4.tif]

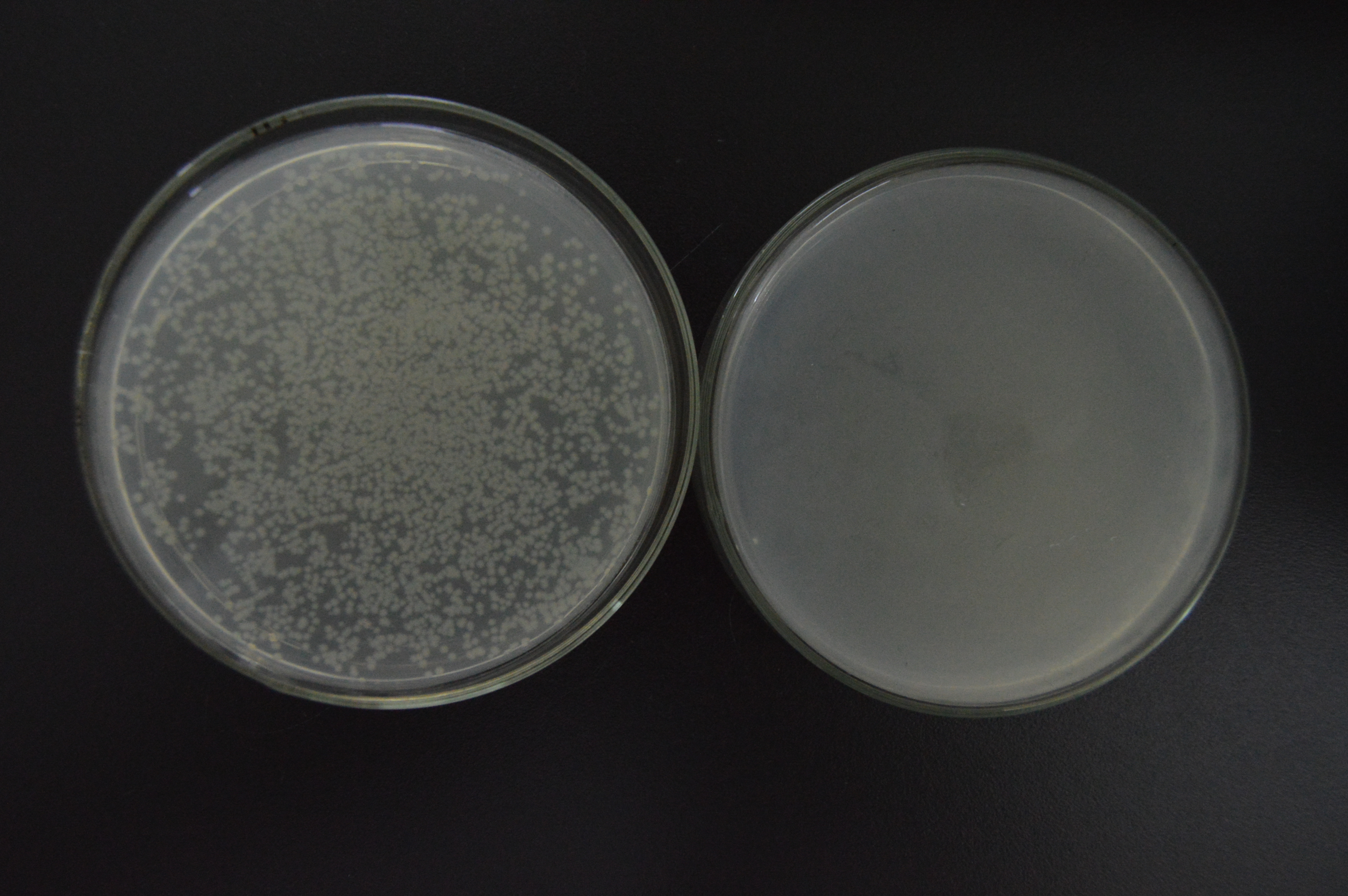

Supplement: Supplementary file 7 [file DataSheet1.ZIP › Figure 1/Fig1B.JPG]

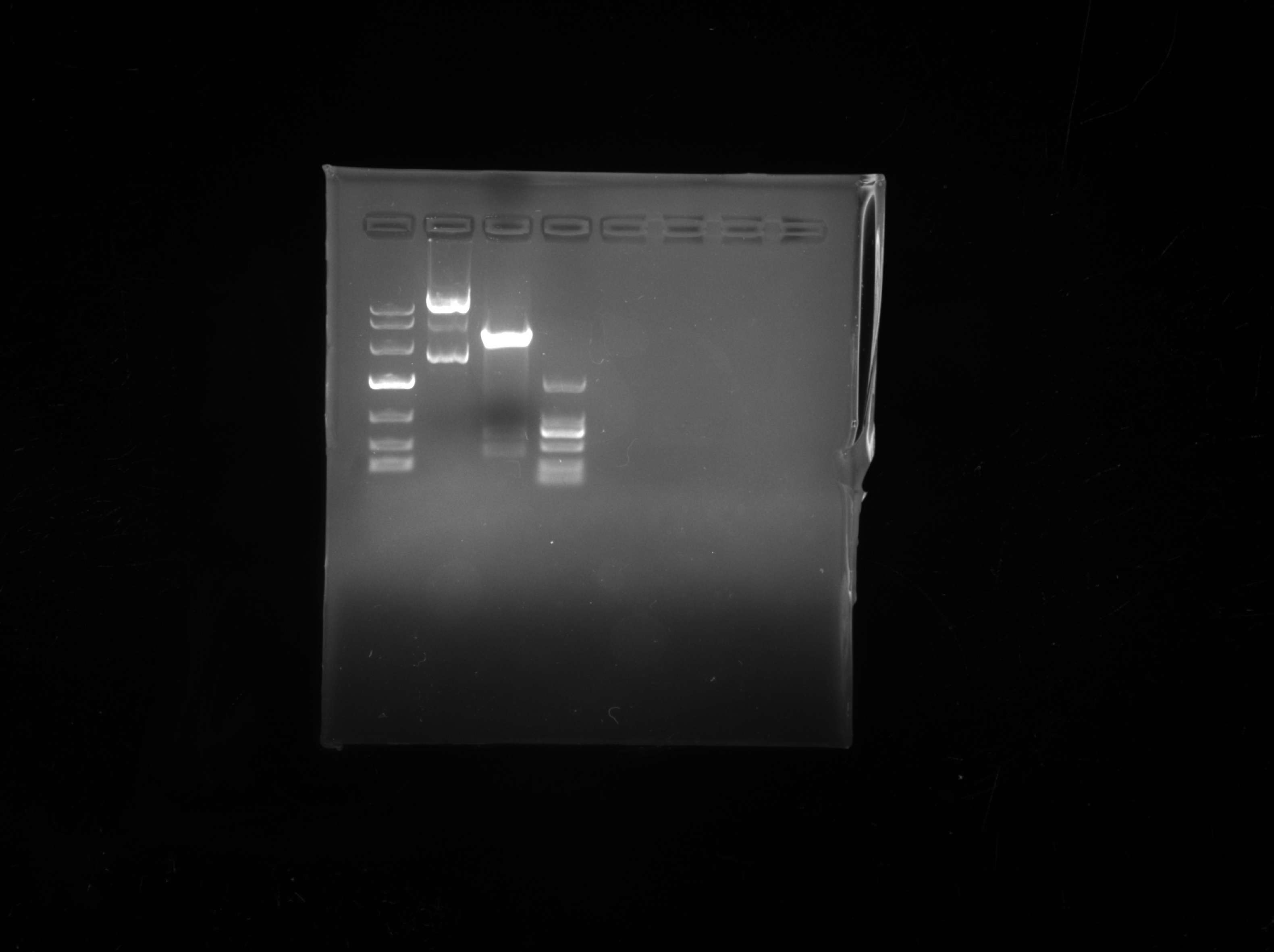

Supplement: Supplementary file 7 [file DataSheet1.ZIP › Figure 1/Fig1C.jpg]

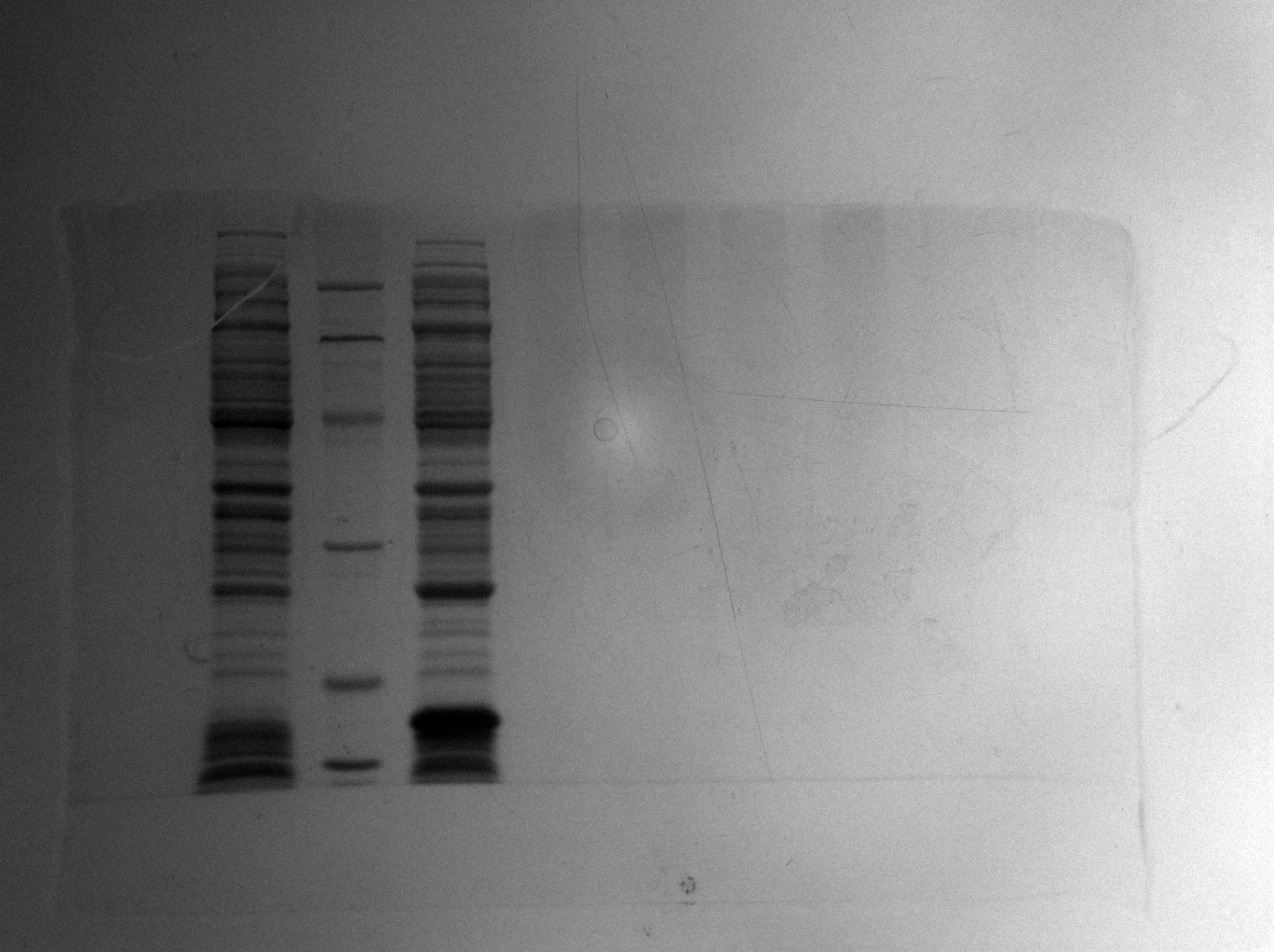

Supplement: Supplementary file 7 [file DataSheet1.ZIP › Figure 1/Fig1D-1.tif]

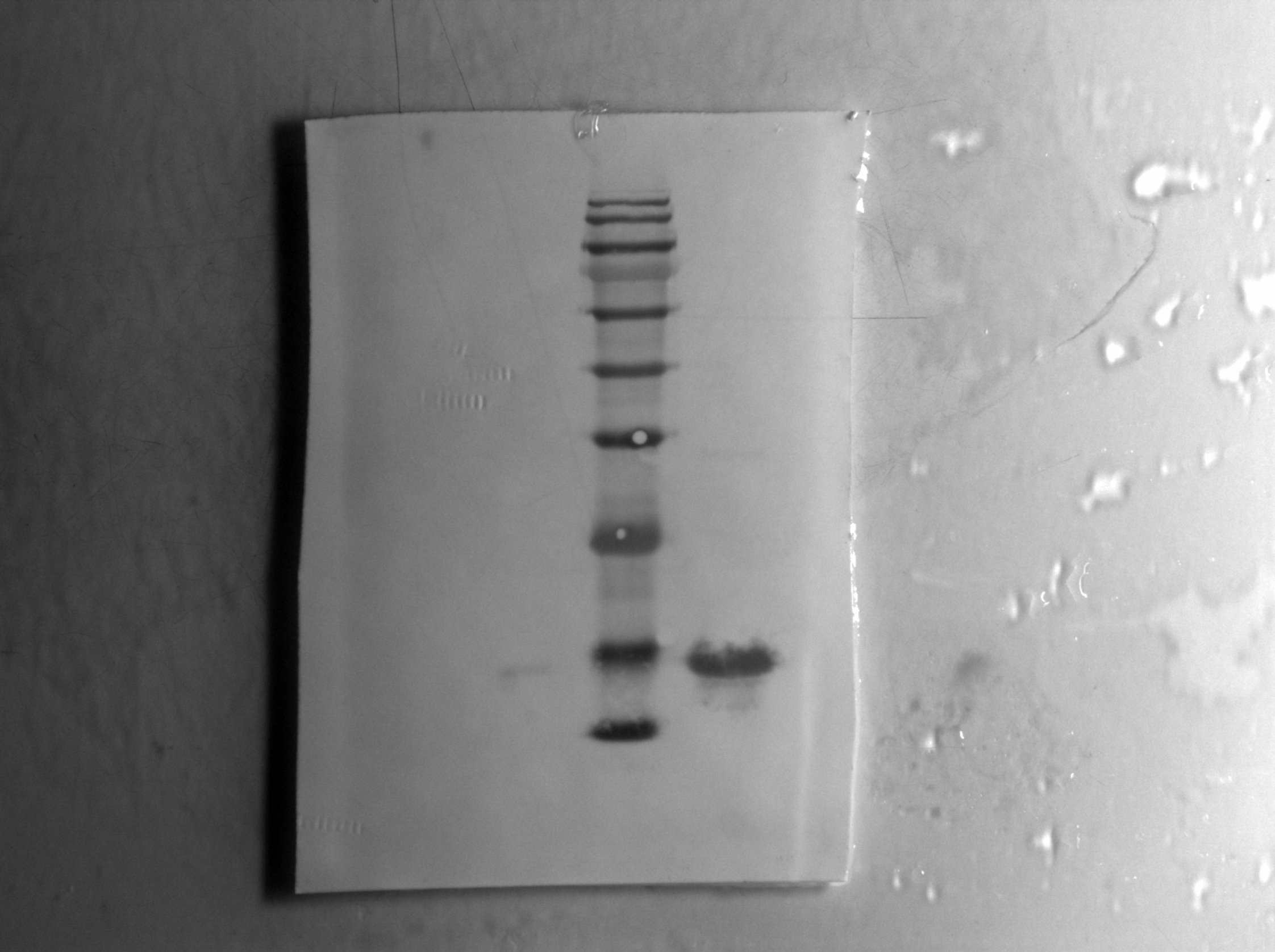

Supplement: Supplementary file 7 [file DataSheet1.ZIP › Figure 1/Fig1D-2.jpg]

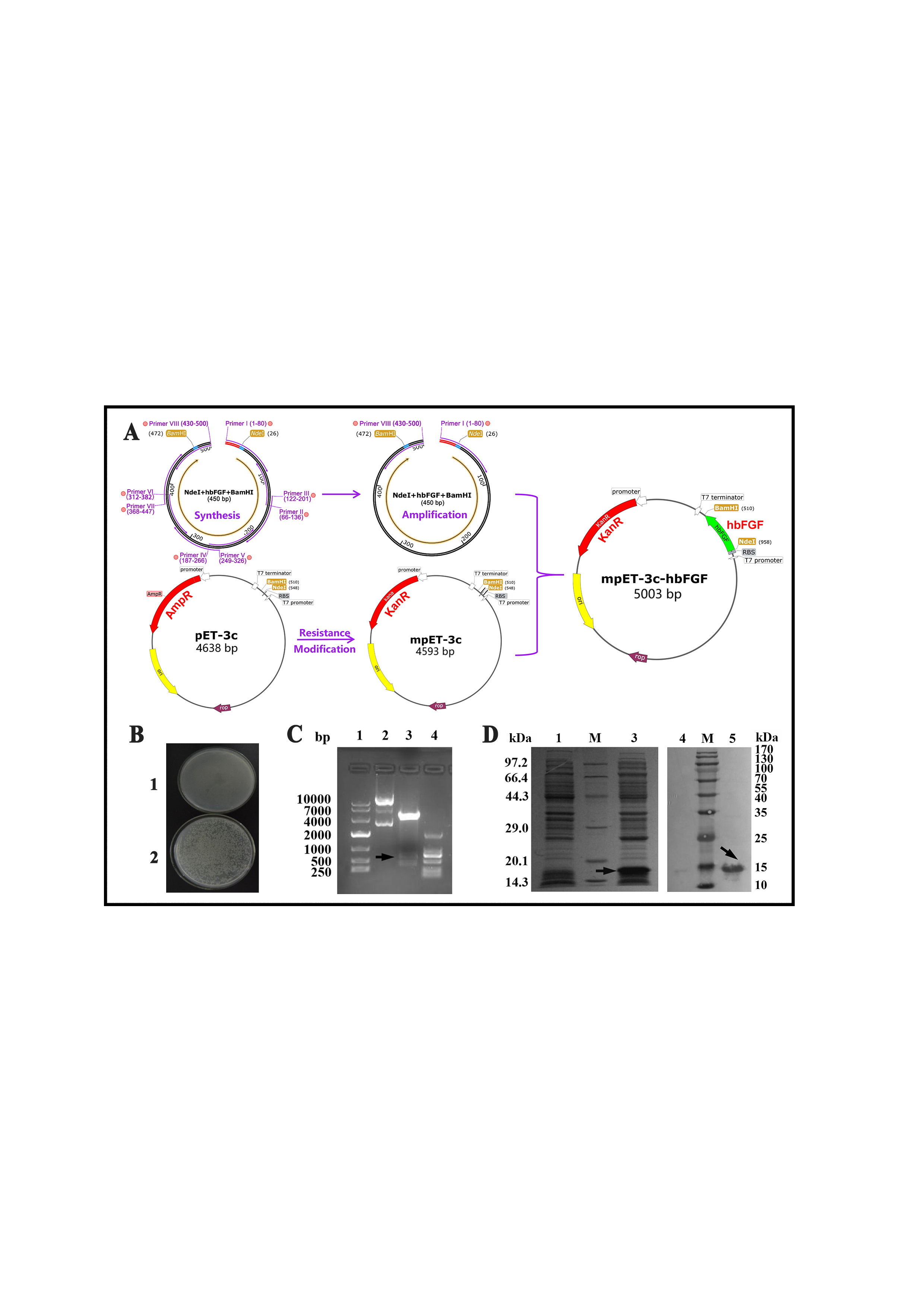

Supplement: Supplementary file 7 [file DataSheet1.ZIP › Figure 1/Figure 1.tif]

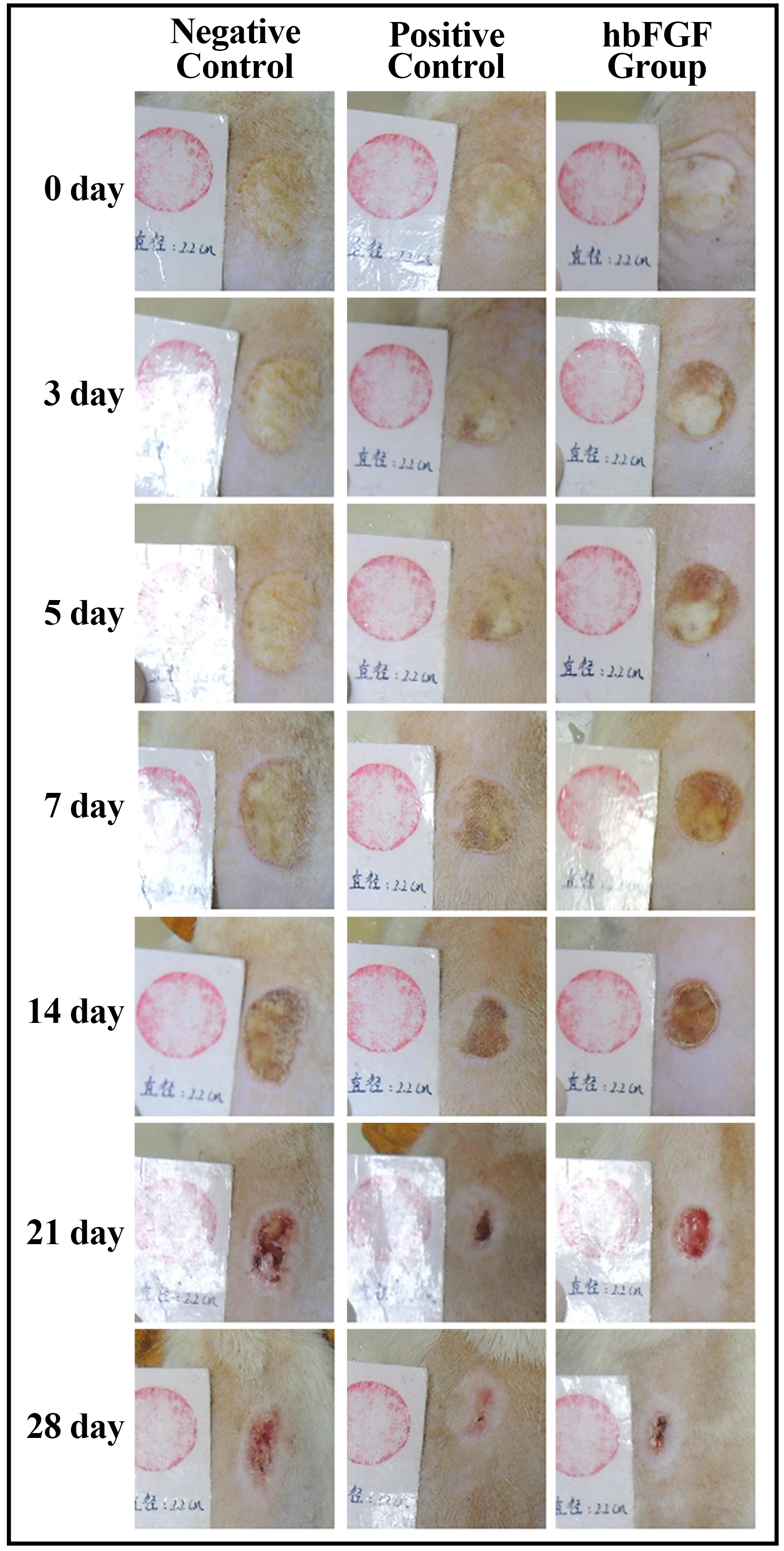

Supplement: Supplementary file 8 [file DataSheet10.ZIP › Supplementary Figure 4/Figure S4.tif]

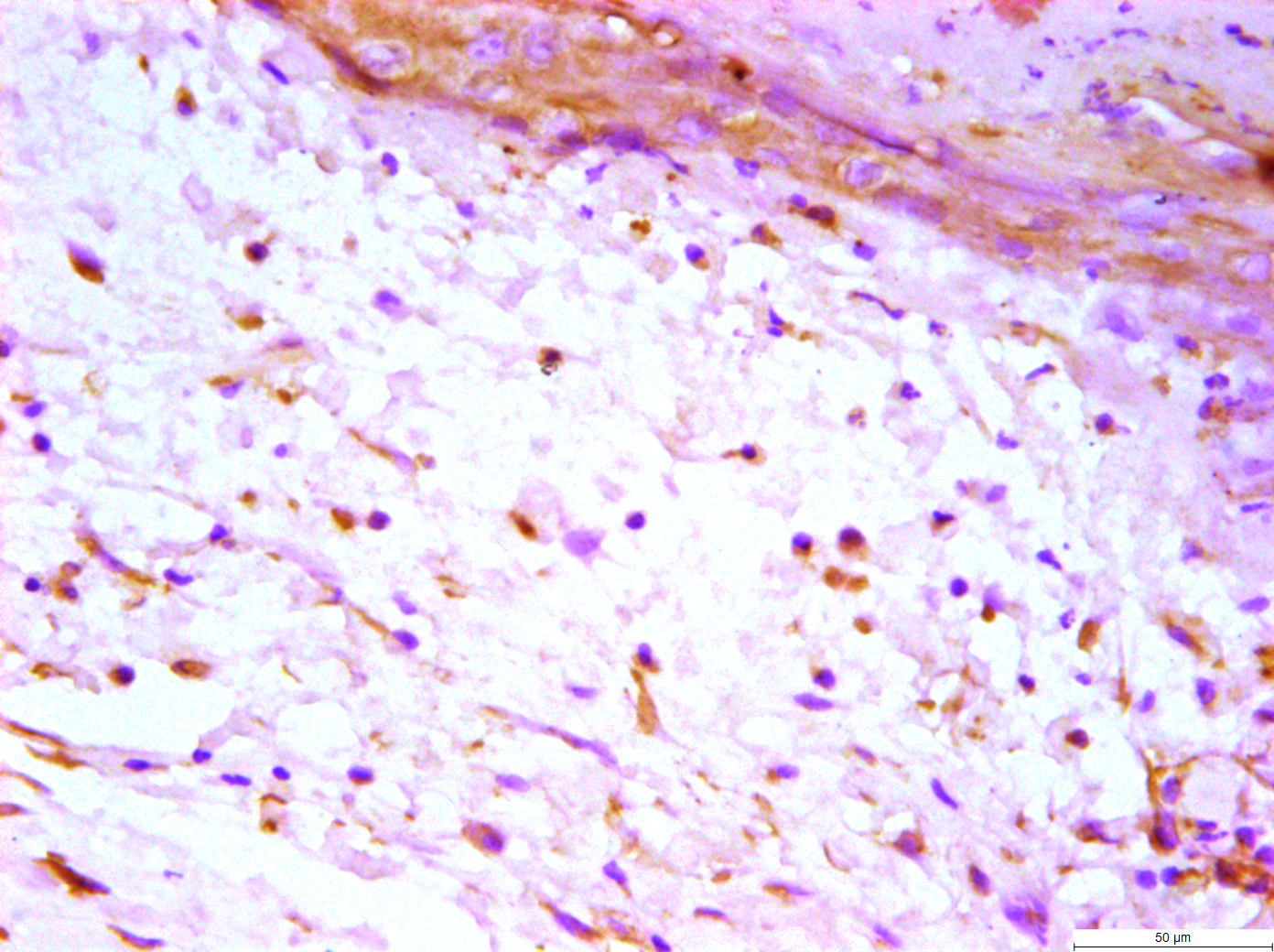

Supplement: Supplementary file 9 [file DataSheet6.ZIP › Figure 6/CD31/Negative.tif]

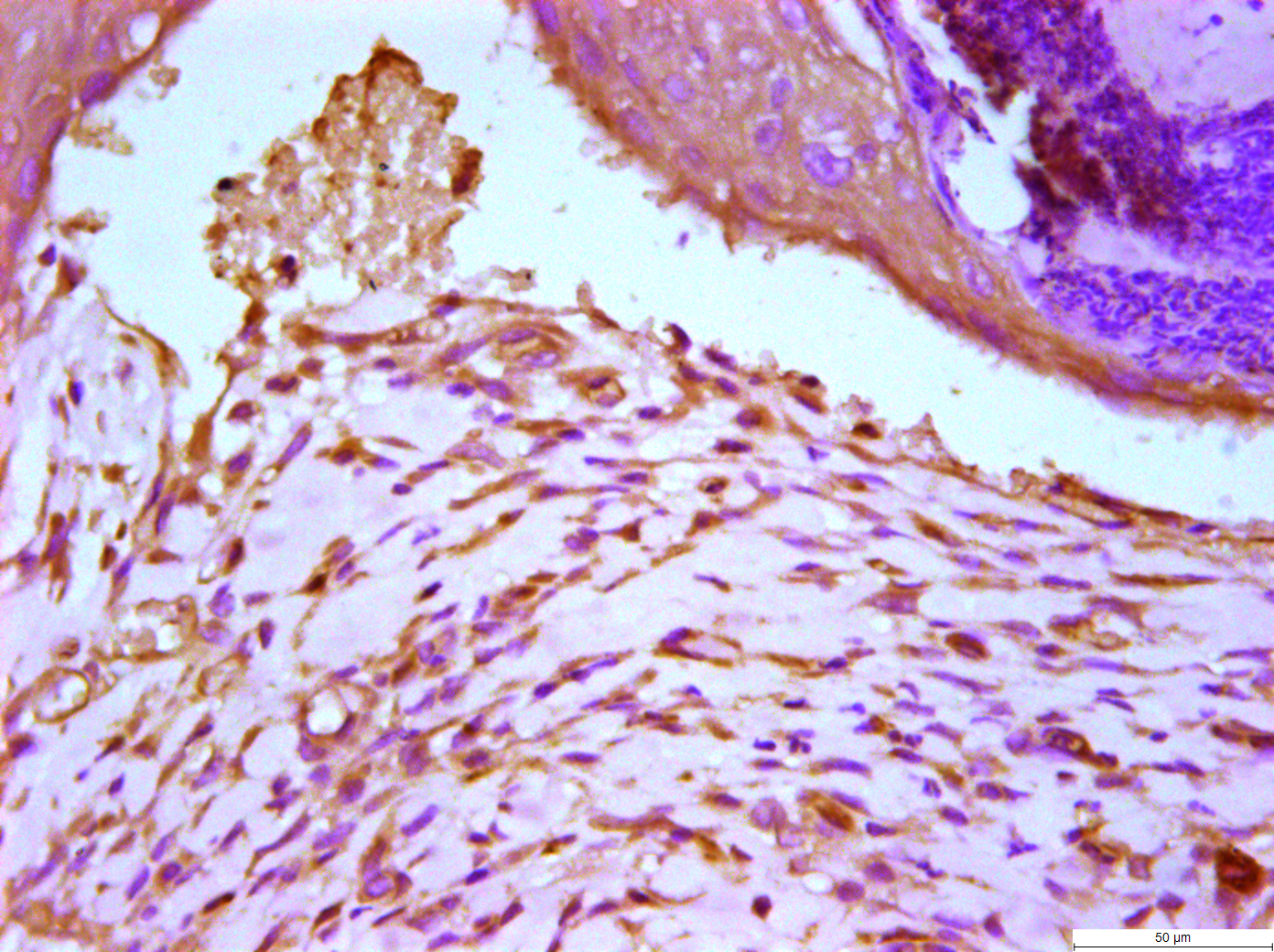

Supplement: Supplementary file 9 [file DataSheet6.ZIP › Figure 6/CD31/Positive.tif]

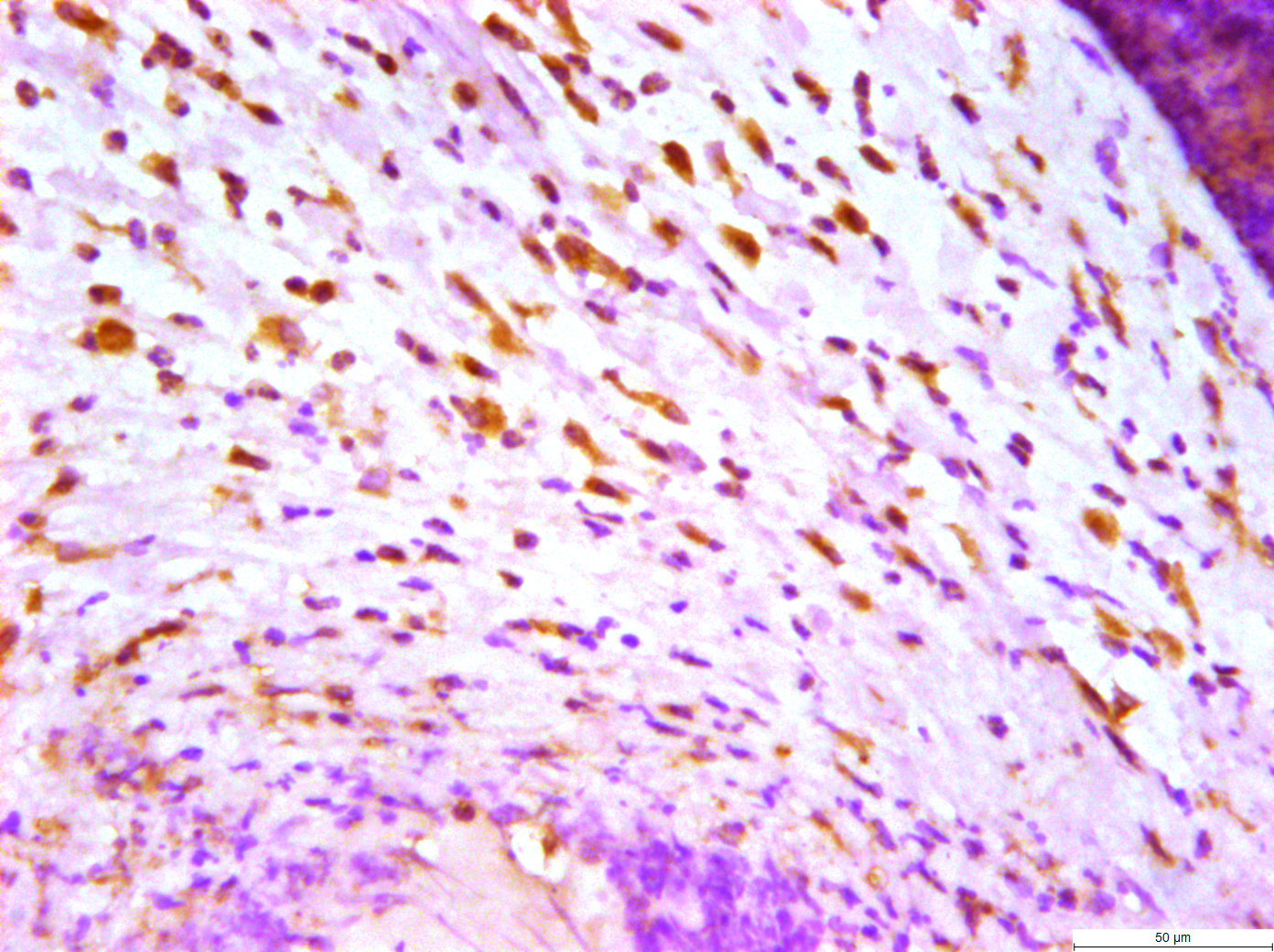

Supplement: Supplementary file 9 [file DataSheet6.ZIP › Figure 6/CD31/hbFGF.tif]

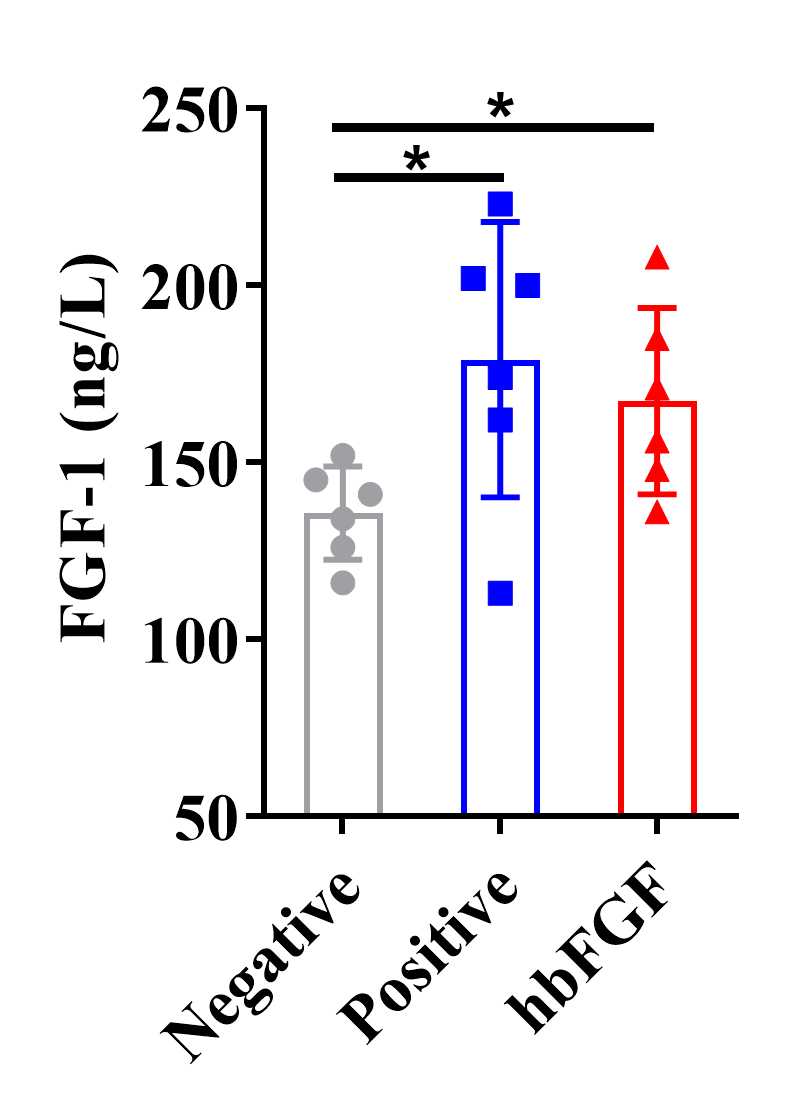

Supplement: Supplementary file 9 [file DataSheet6.ZIP › Figure 6/FGF1.tif]

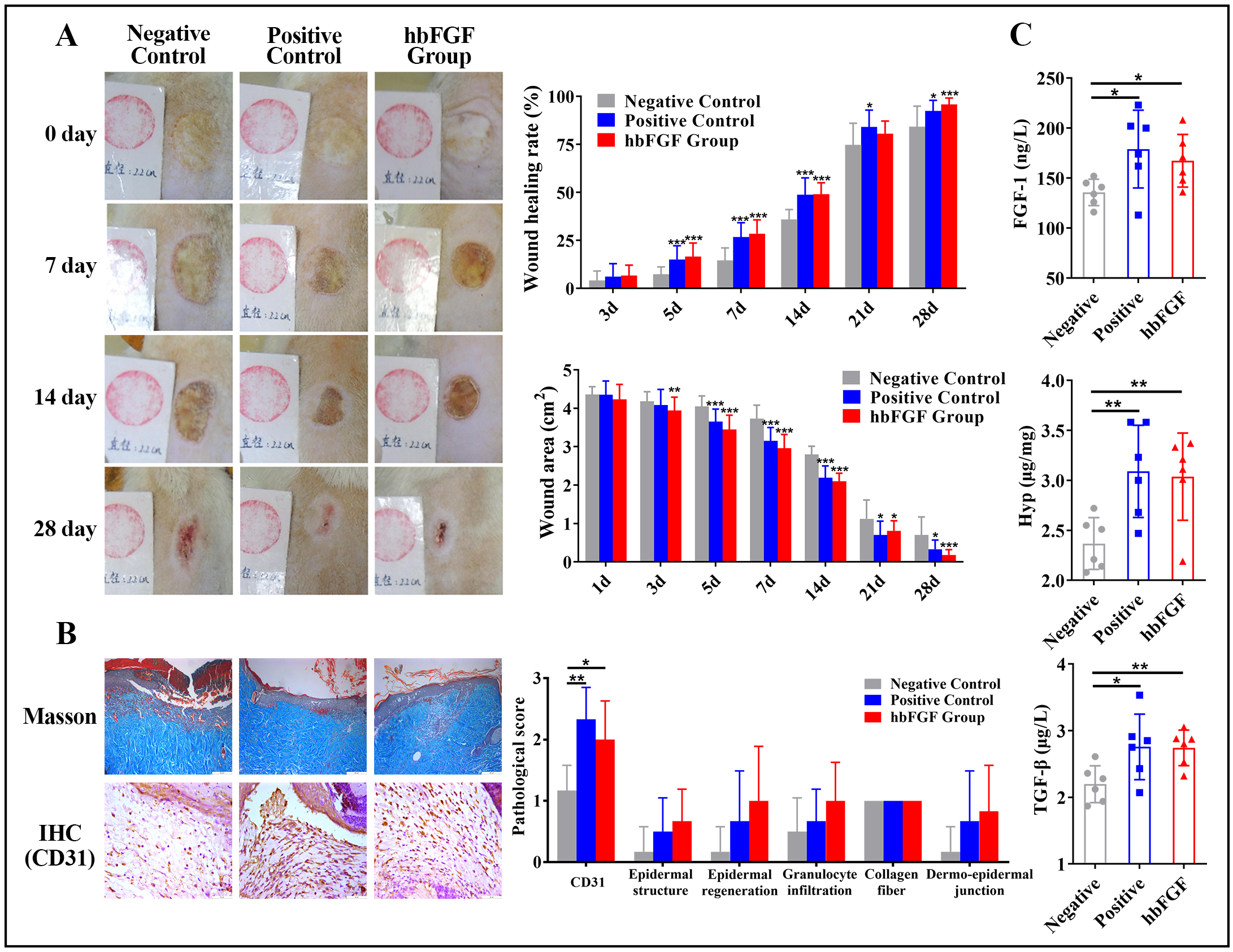

Supplement: Supplementary file 9 [file DataSheet6.ZIP › Figure 6/Figure 6.tif]

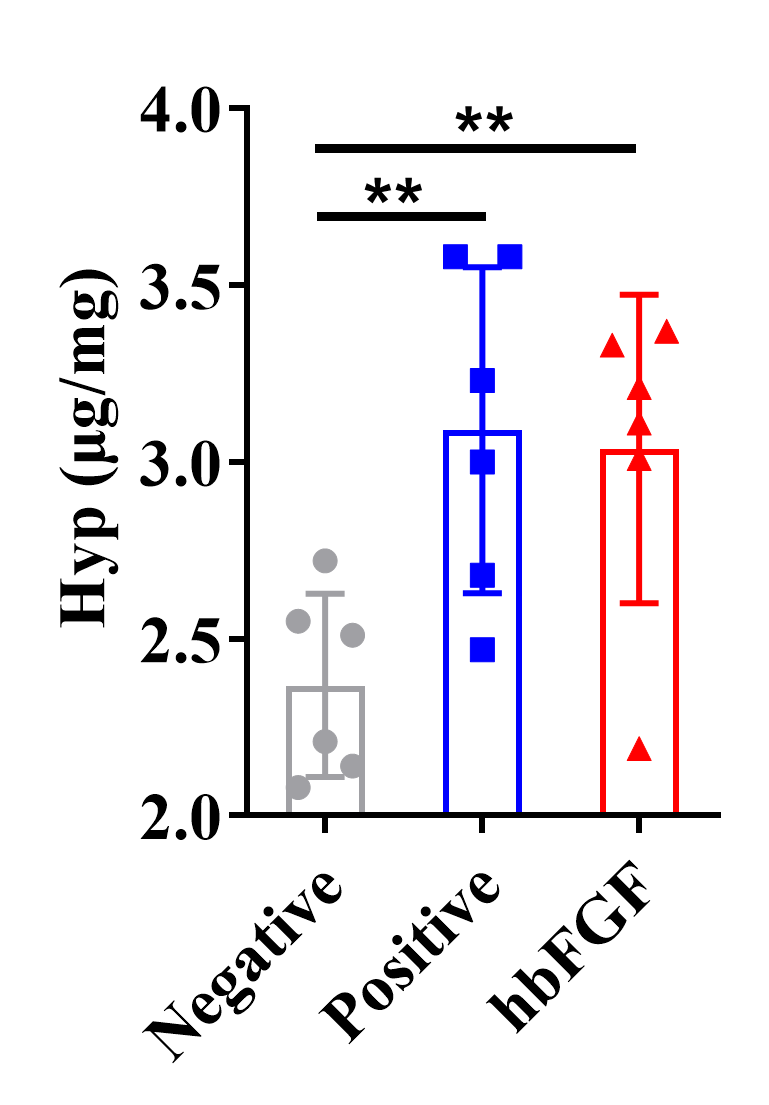

Supplement: Supplementary file 9 [file DataSheet6.ZIP › Figure 6/Hyp.tif]

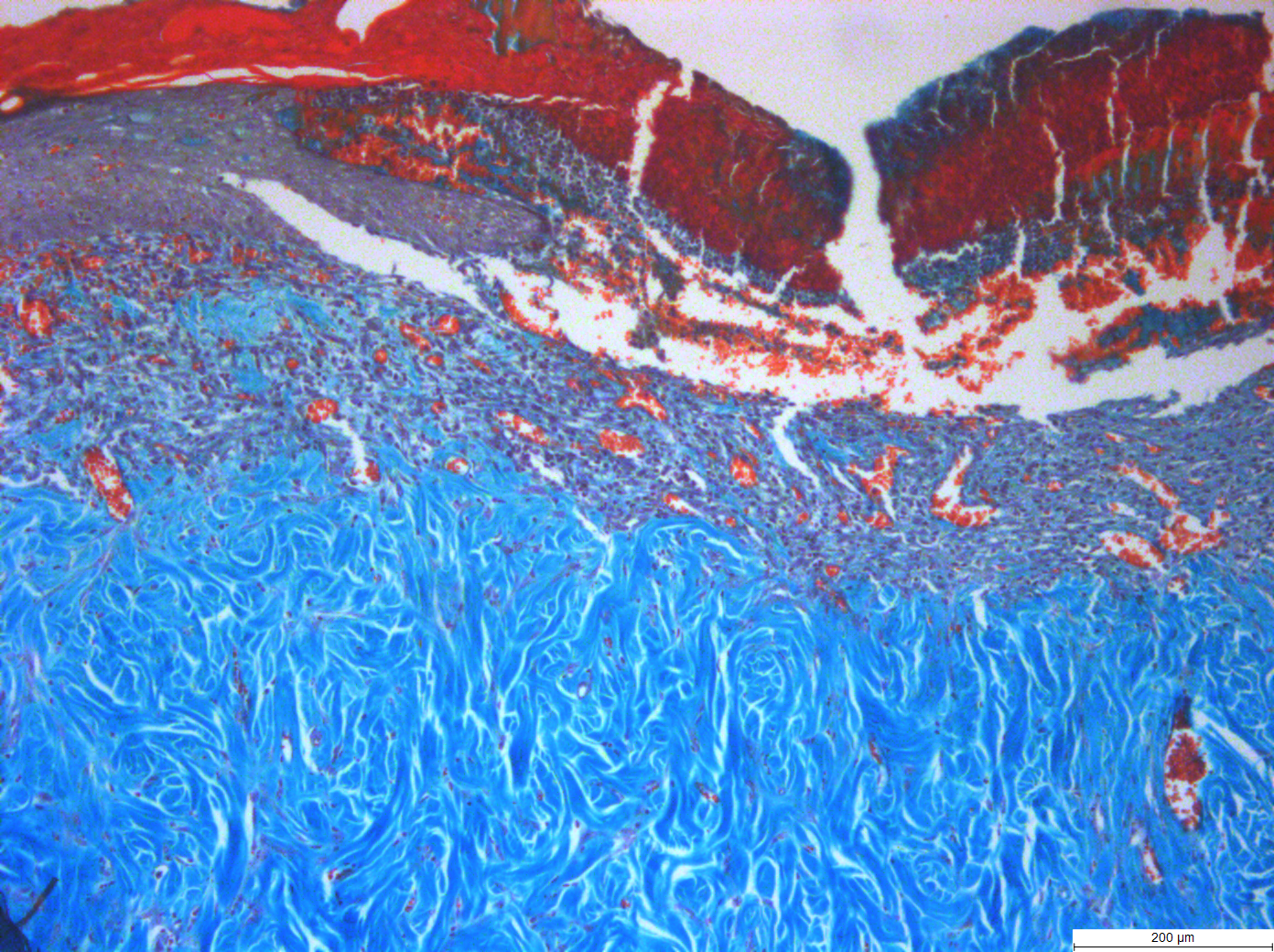

Supplement: Supplementary file 9 [file DataSheet6.ZIP › Figure 6/Masson/Negative.tif]

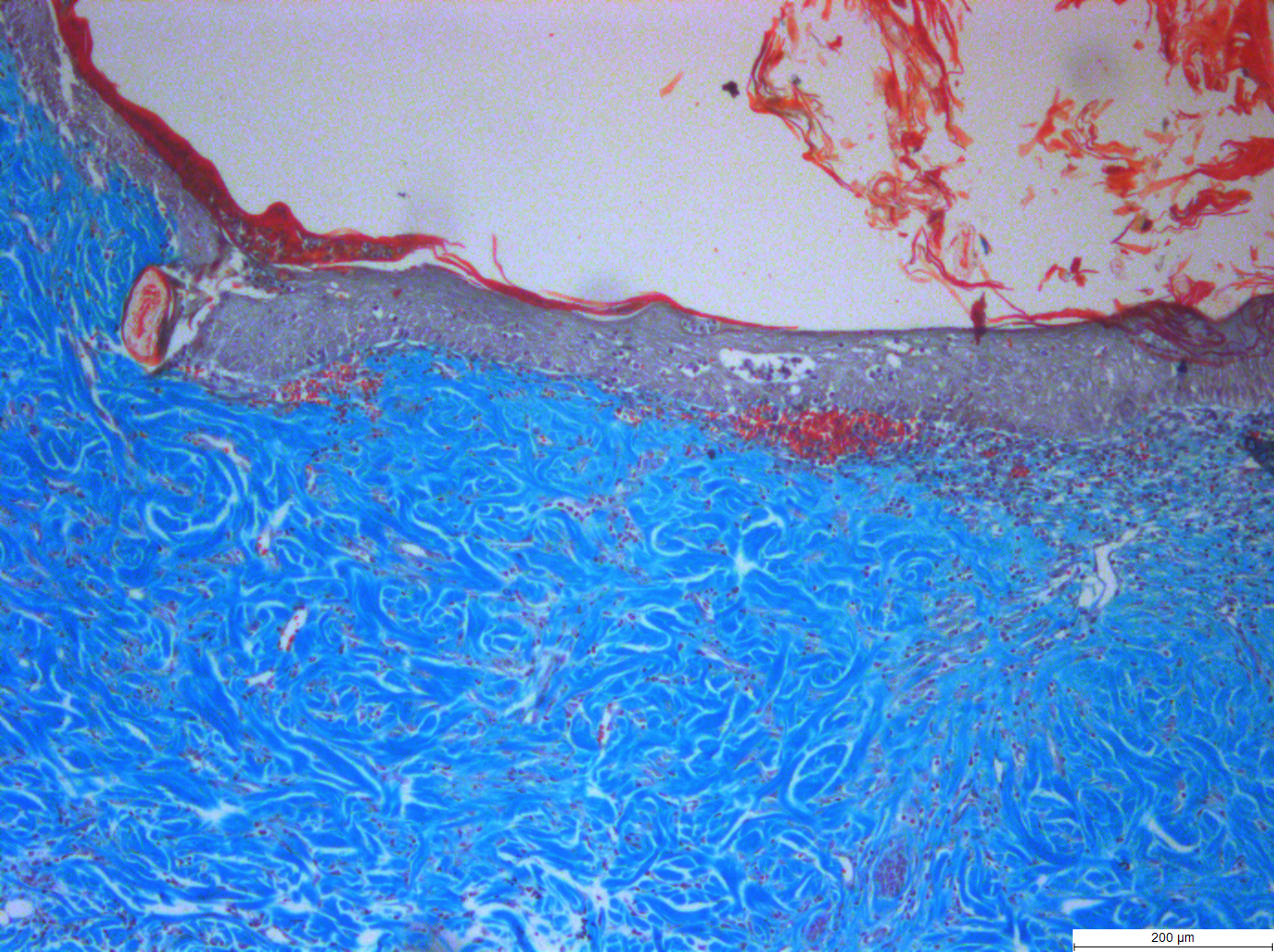

Supplement: Supplementary file 9 [file DataSheet6.ZIP › Figure 6/Masson/Positive.tif]

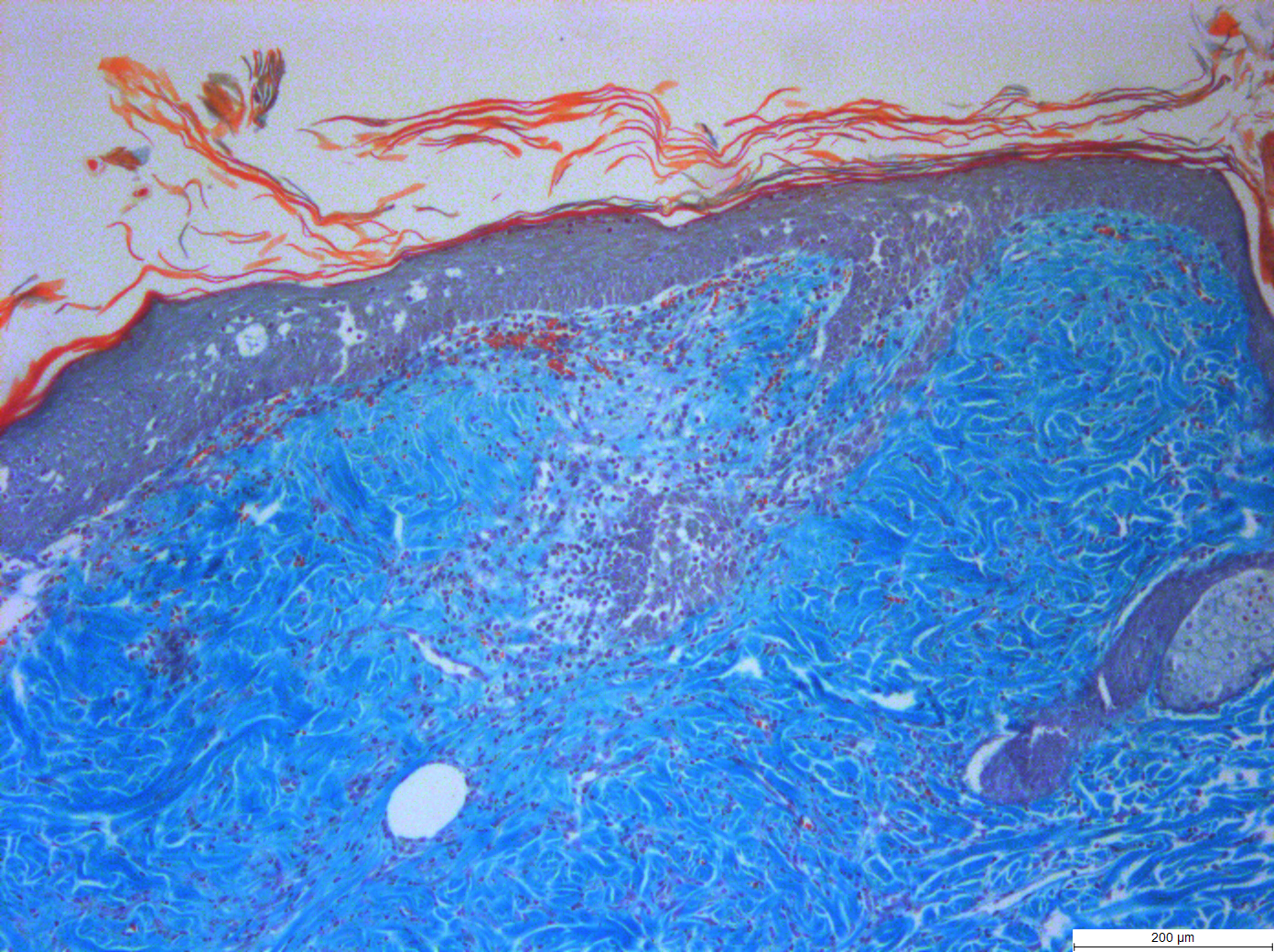

Supplement: Supplementary file 9 [file DataSheet6.ZIP › Figure 6/Masson/hbFGF.tif]

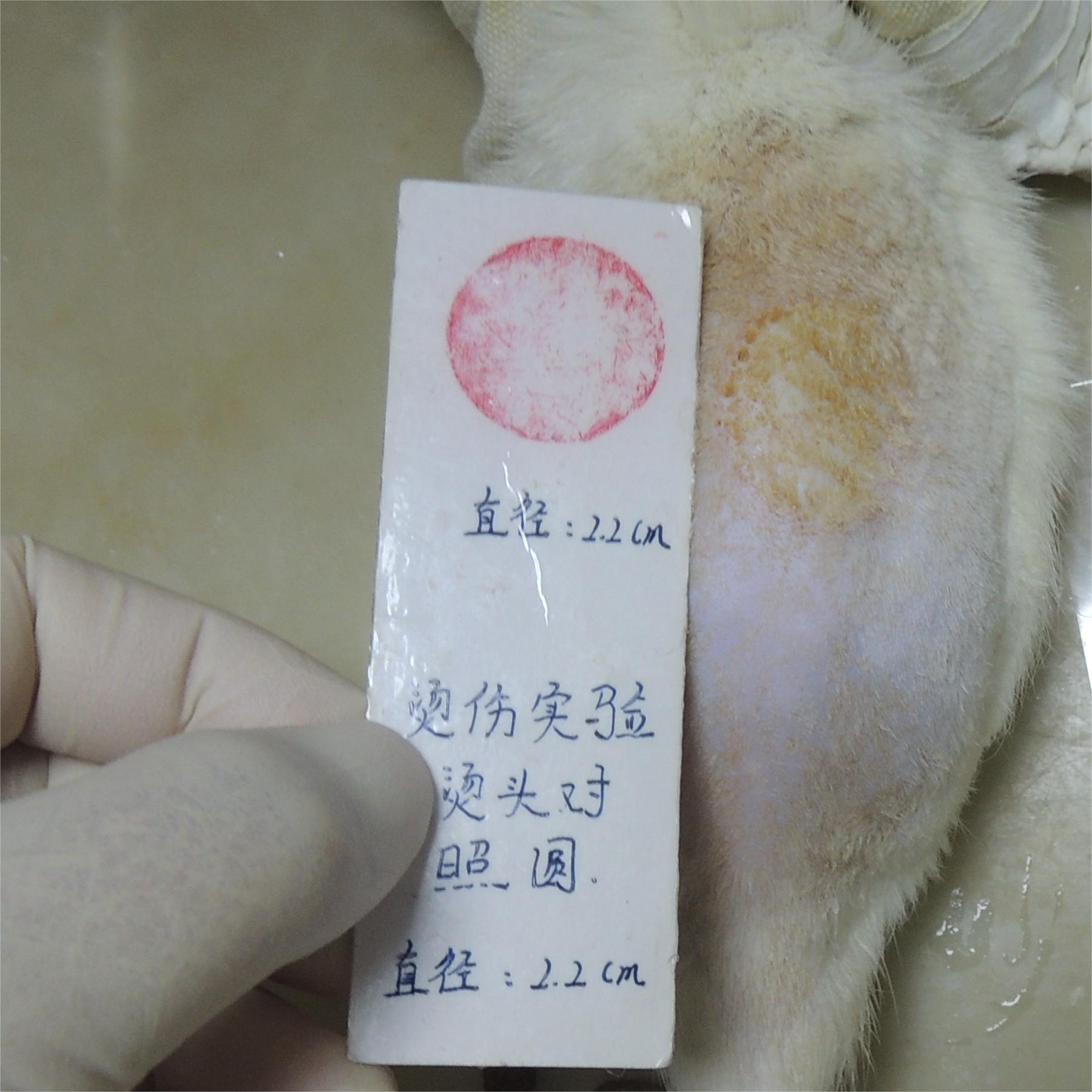

Supplement: Supplementary file 9 [file DataSheet6.ZIP › Figure 6/Photographs of wound/0d/Negative.JPG]

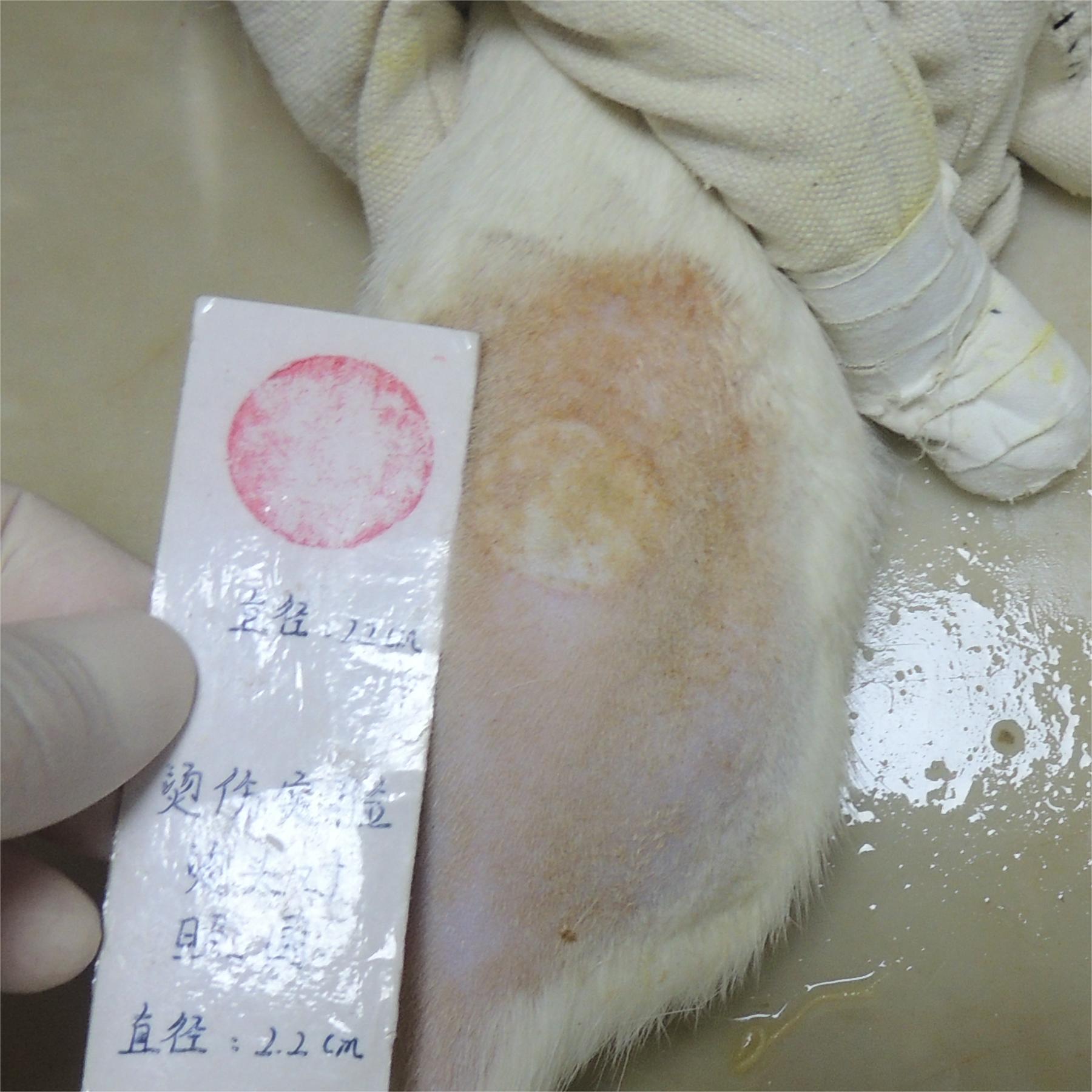

Supplement: Supplementary file 9 [file DataSheet6.ZIP › Figure 6/Photographs of wound/0d/Positive.JPG]

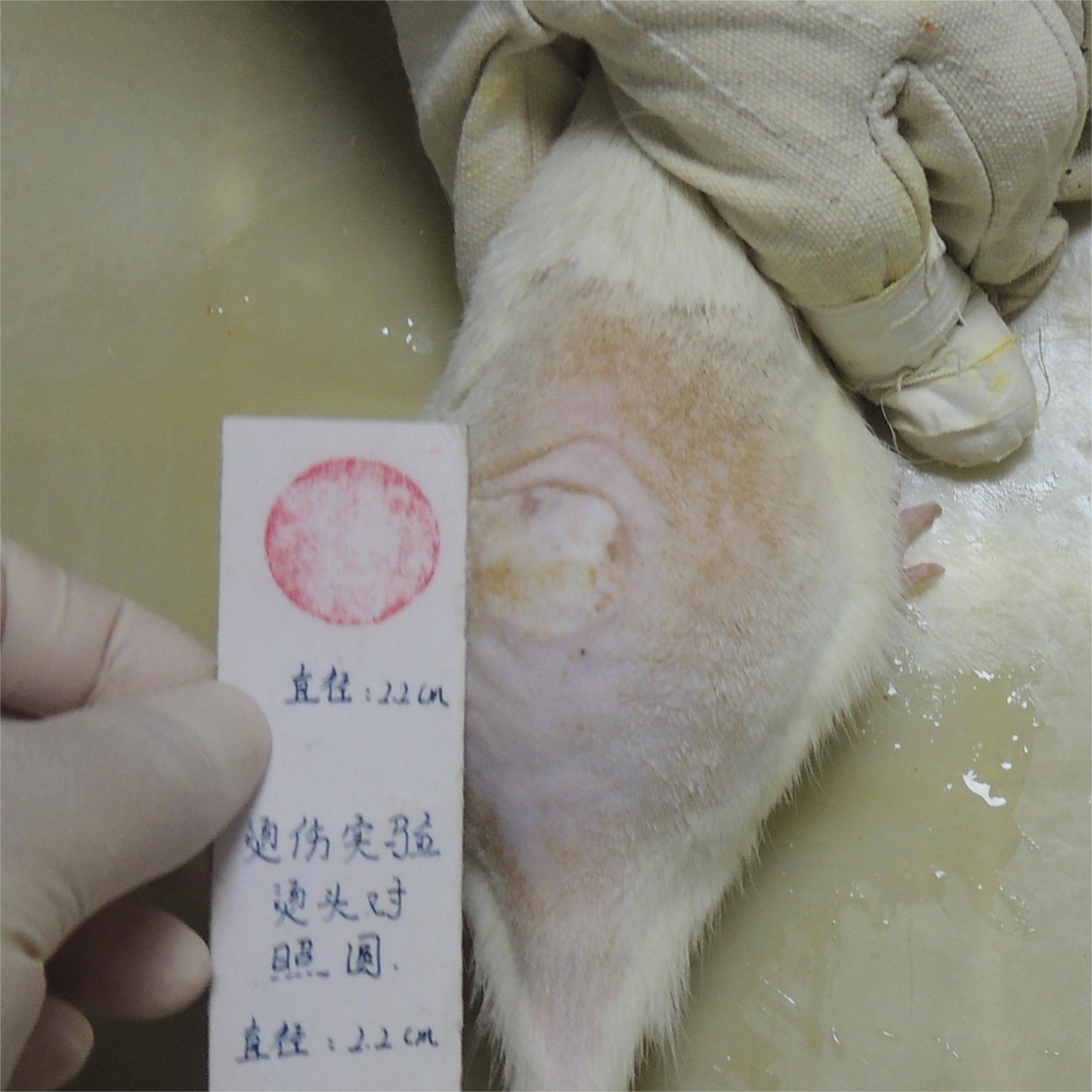

Supplement: Supplementary file 9 [file DataSheet6.ZIP › Figure 6/Photographs of wound/0d/hbFGF.JPG]

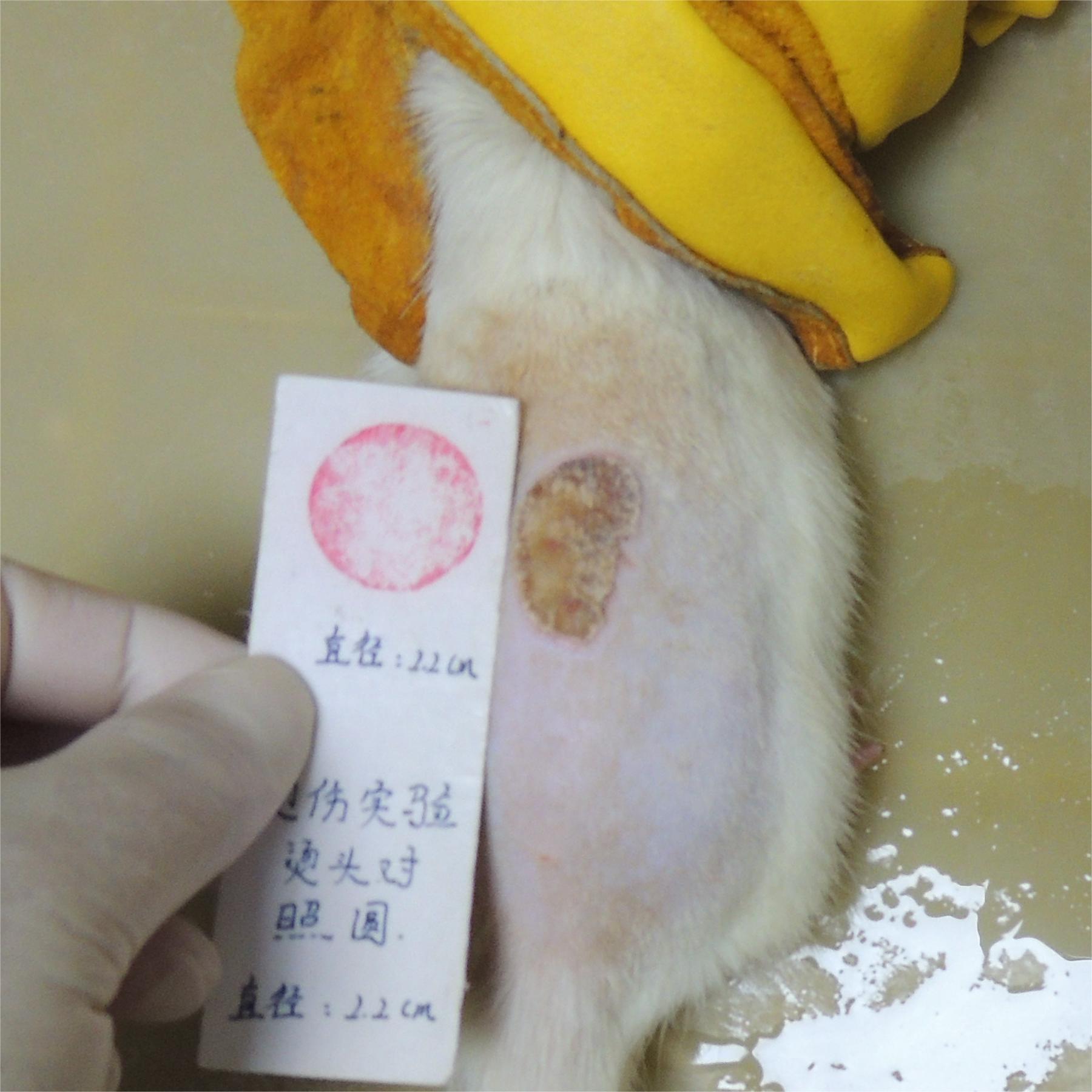

Supplement: Supplementary file 9 [file DataSheet6.ZIP › Figure 6/Photographs of wound/14d/Negative.JPG]

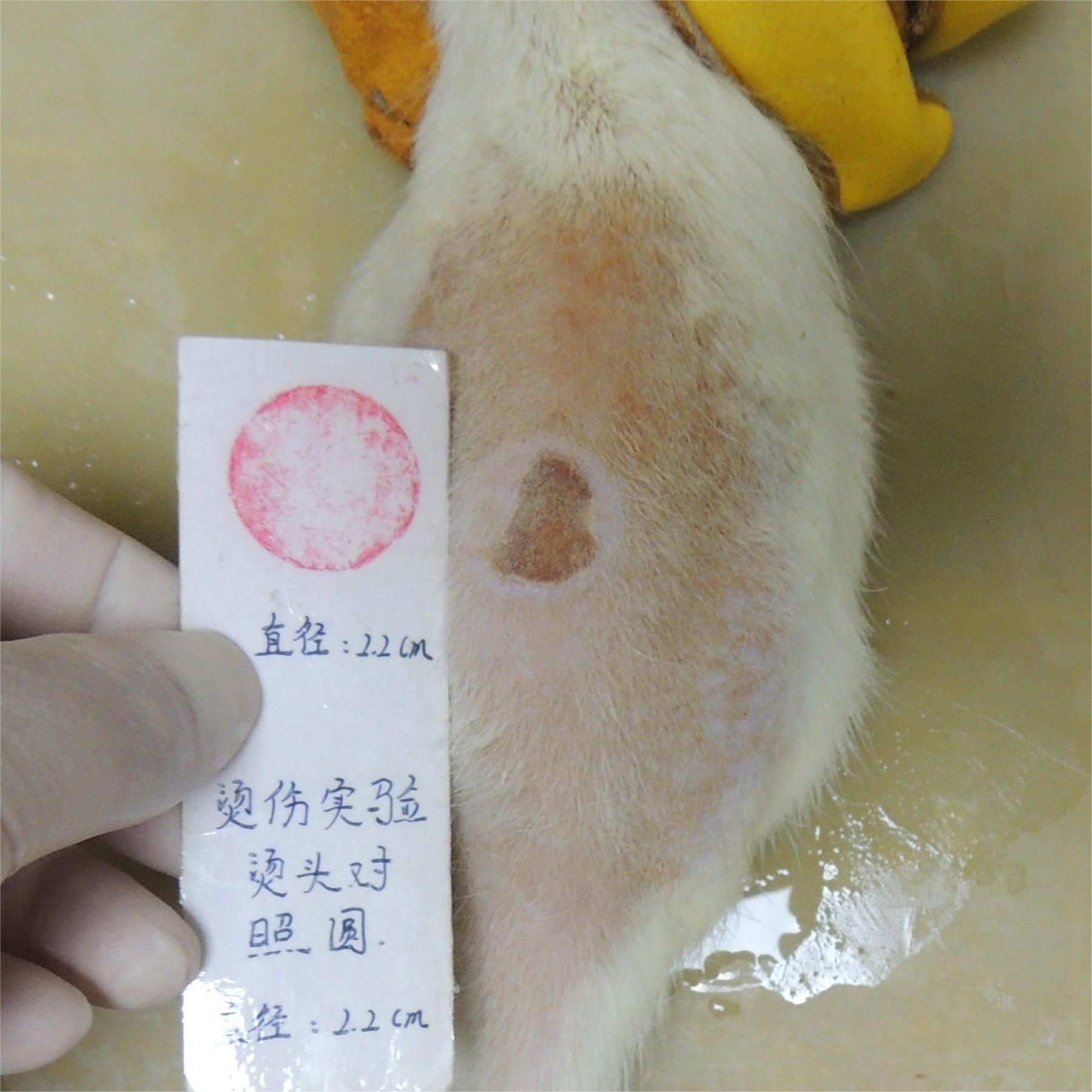

Supplement: Supplementary file 9 [file DataSheet6.ZIP › Figure 6/Photographs of wound/14d/Positive.JPG]

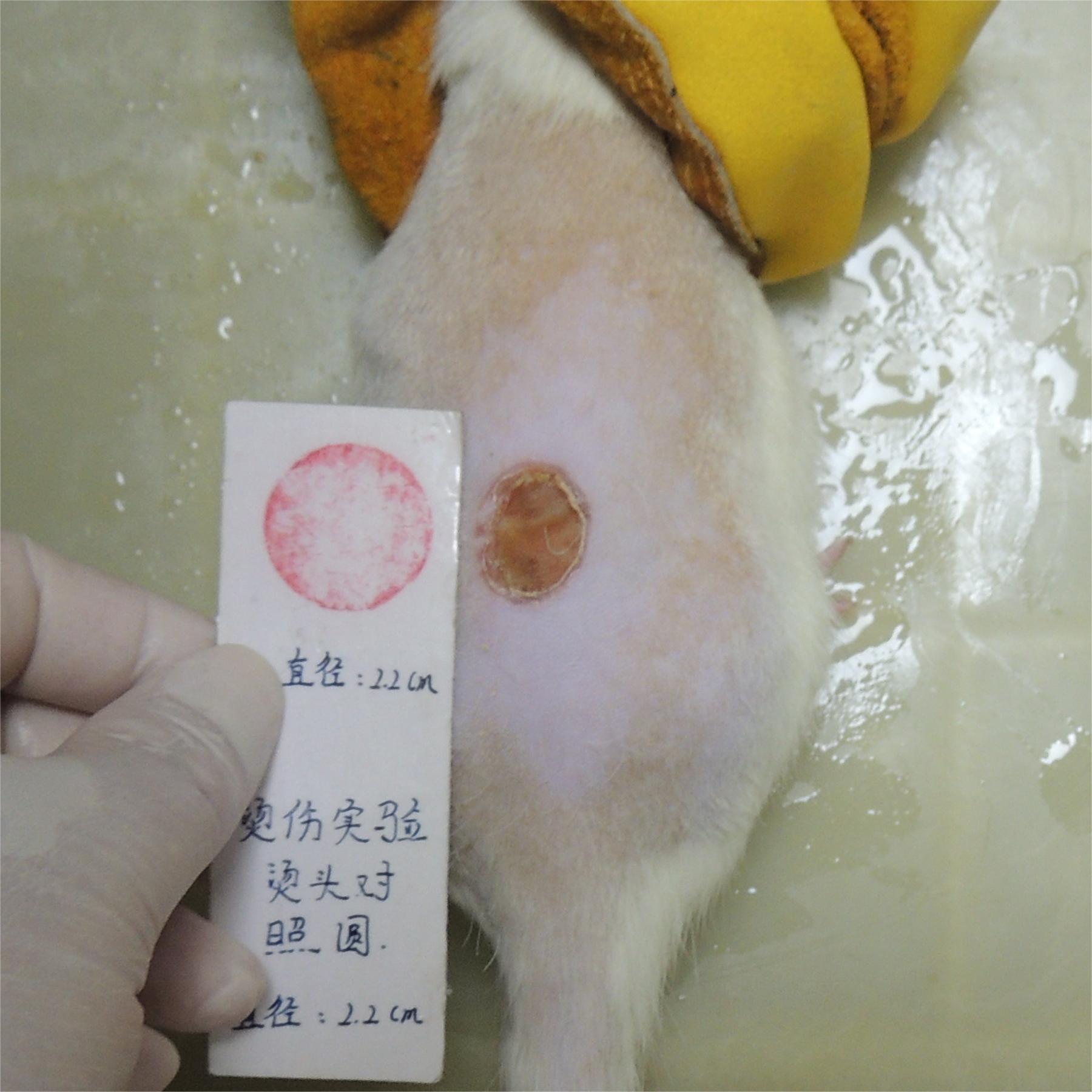

Supplement: Supplementary file 9 [file DataSheet6.ZIP › Figure 6/Photographs of wound/14d/hbFGF.JPG]

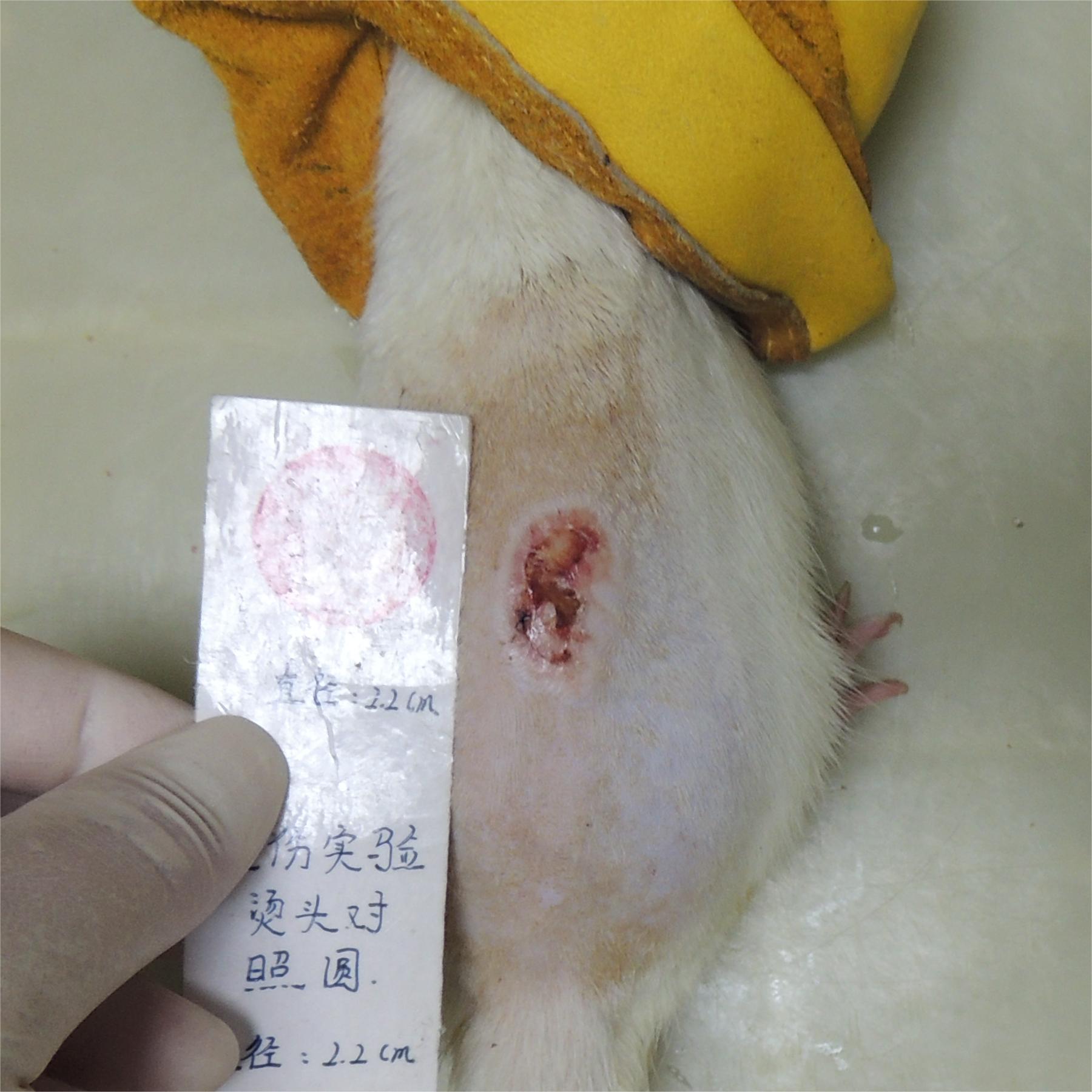

Supplement: Supplementary file 9 [file DataSheet6.ZIP › Figure 6/Photographs of wound/21d/Negative.JPG]

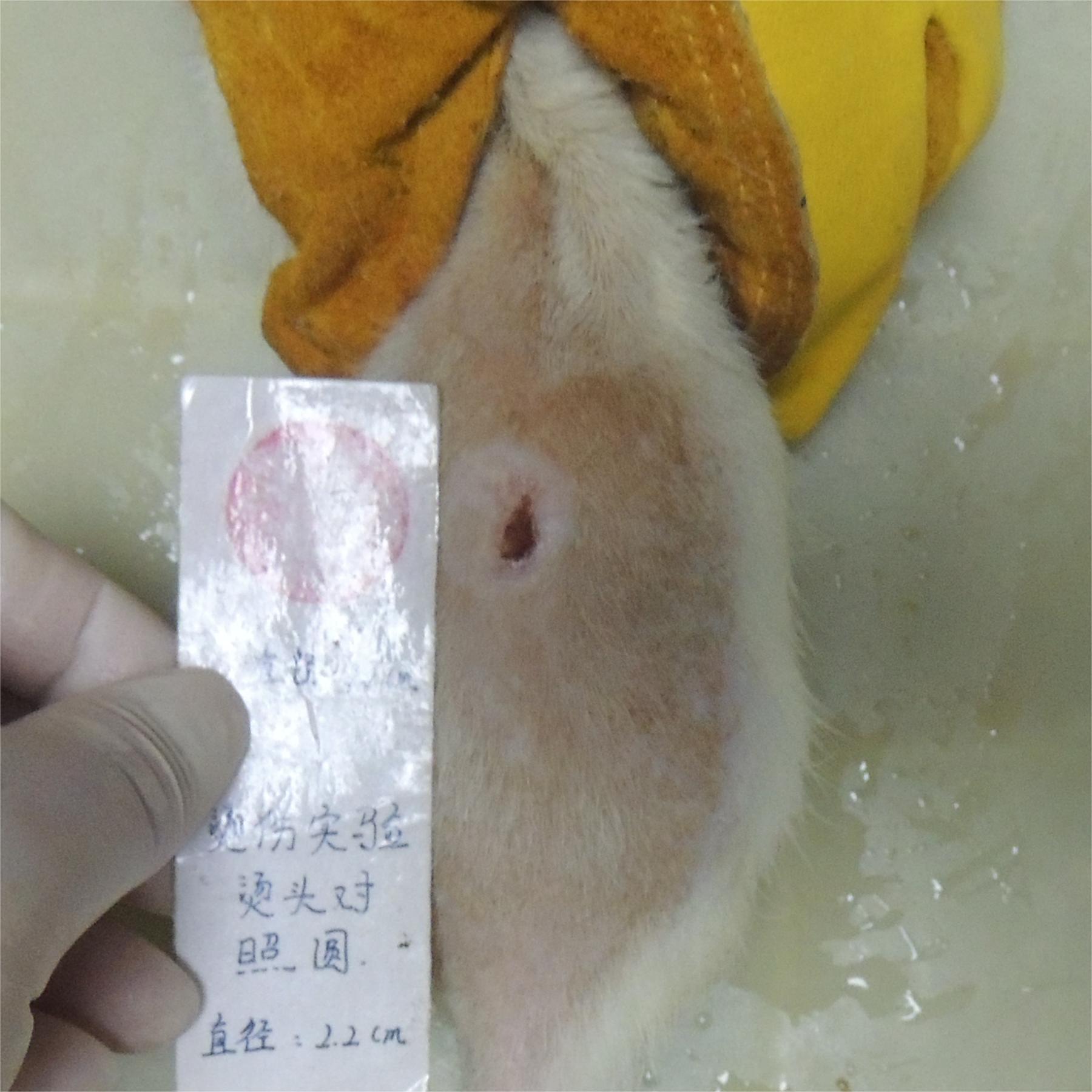

Supplement: Supplementary file 9 [file DataSheet6.ZIP › Figure 6/Photographs of wound/21d/Positive.JPG]

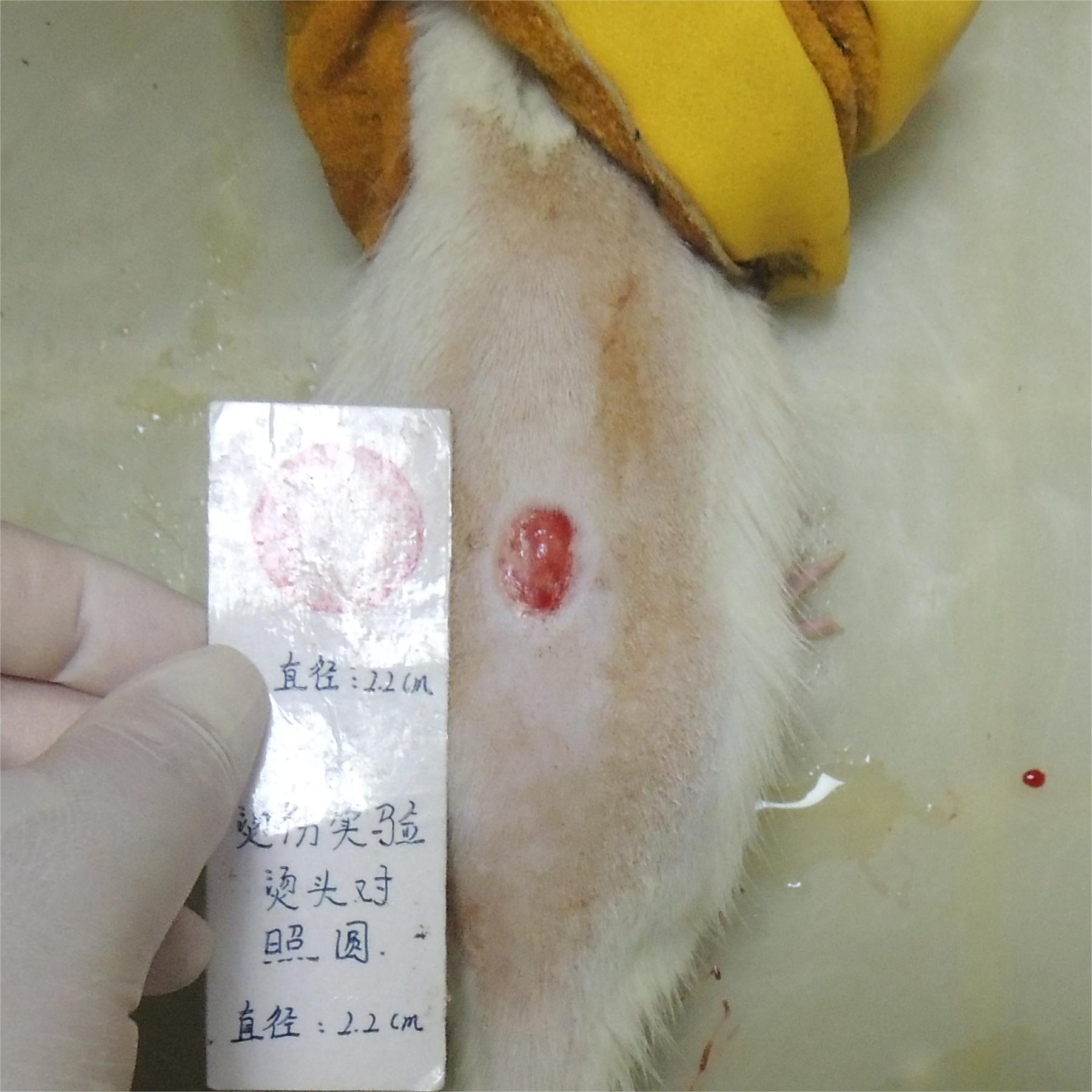

Supplement: Supplementary file 9 [file DataSheet6.ZIP › Figure 6/Photographs of wound/21d/hbFGF.JPG]

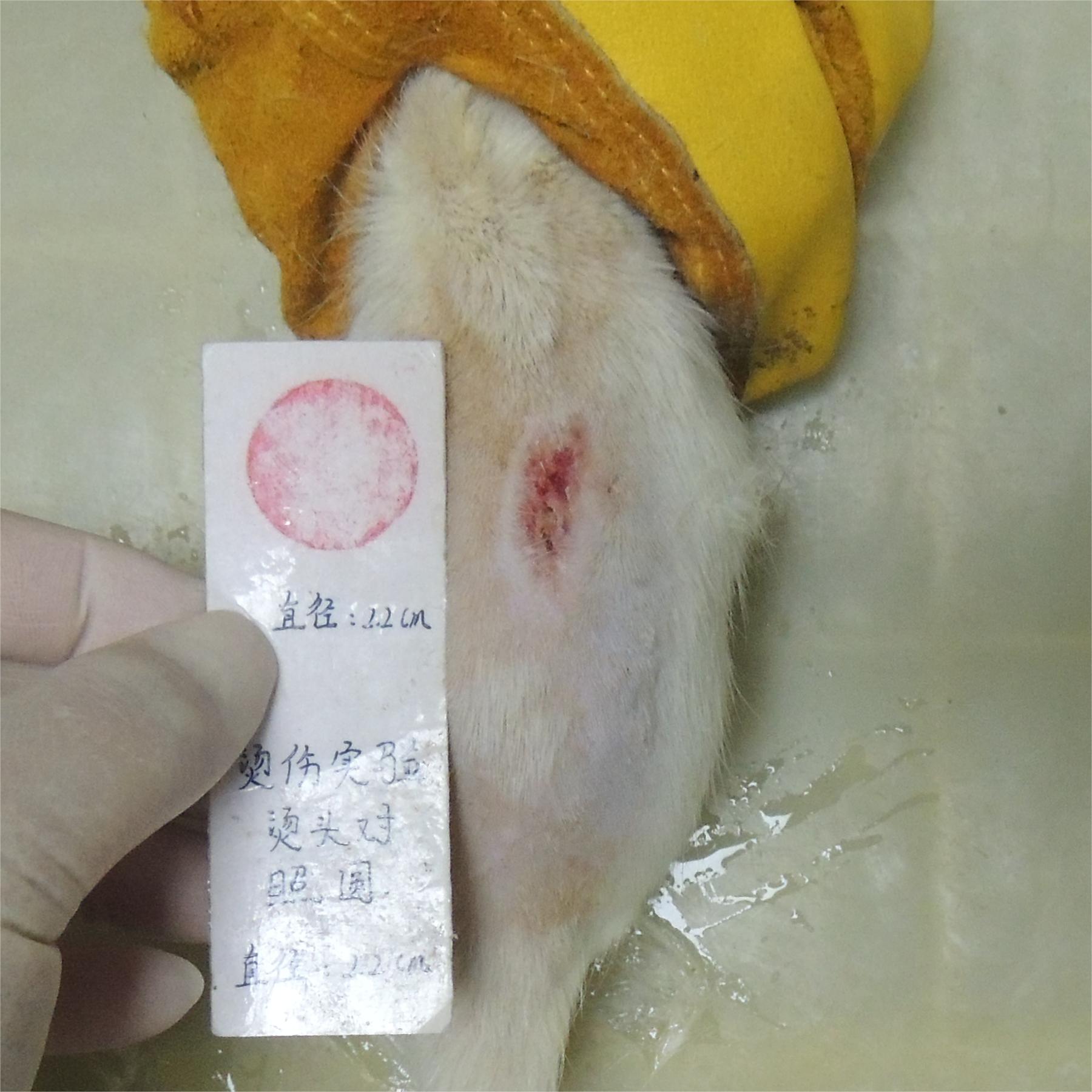

Supplement: Supplementary file 9 [file DataSheet6.ZIP › Figure 6/Photographs of wound/28d/Negative.JPG]

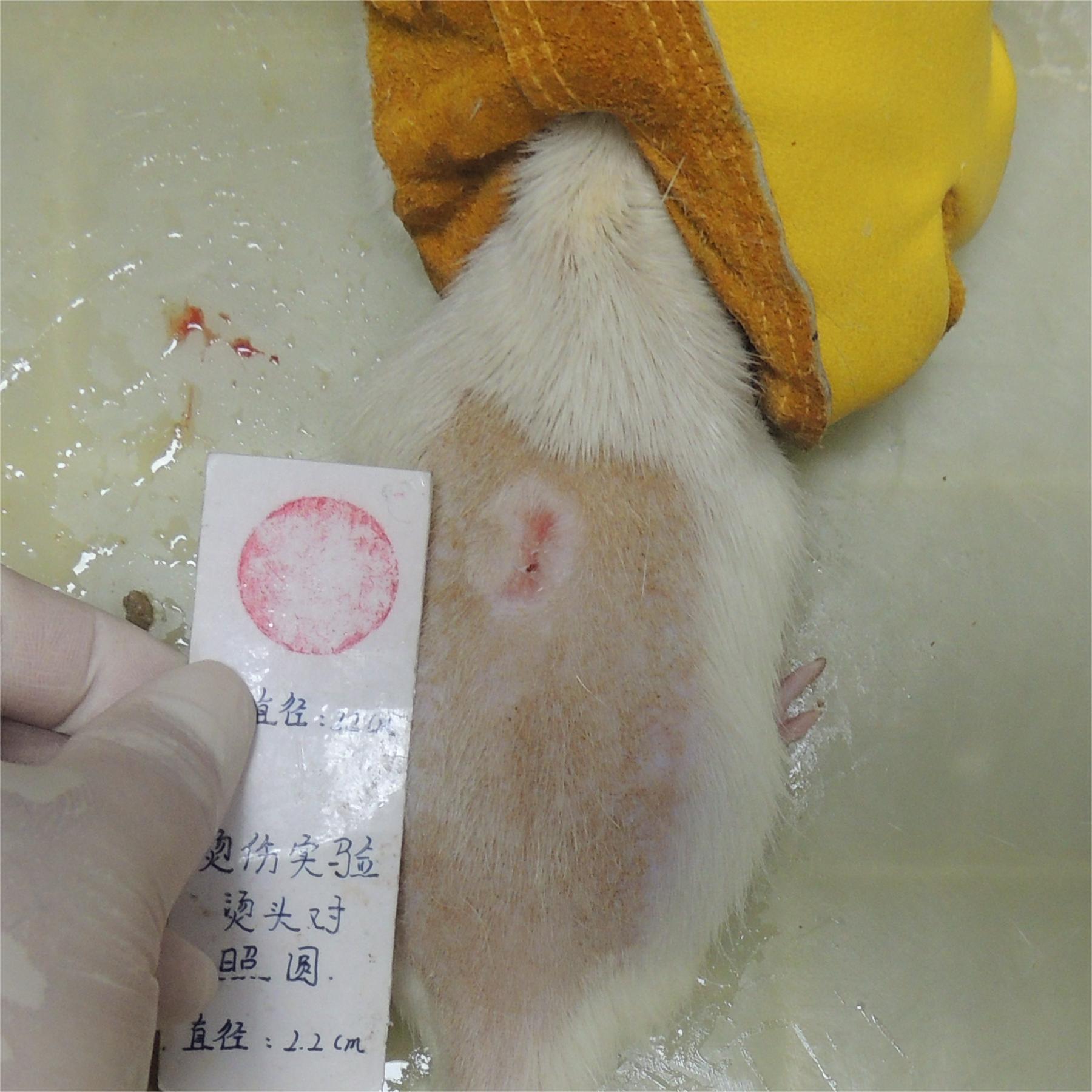

Supplement: Supplementary file 9 [file DataSheet6.ZIP › Figure 6/Photographs of wound/28d/Positive.JPG]

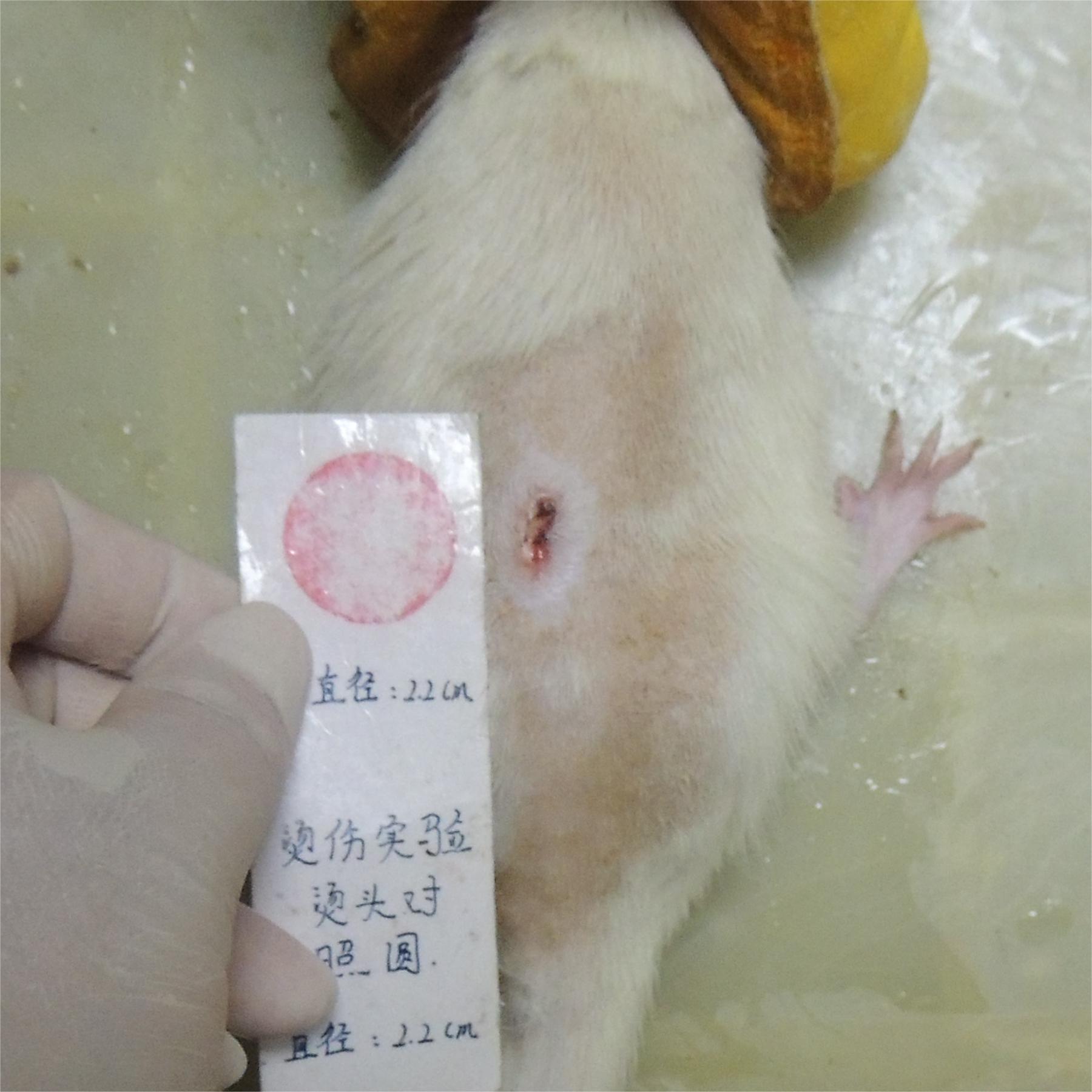

Supplement: Supplementary file 9 [file DataSheet6.ZIP › Figure 6/Photographs of wound/28d/hbFGF.JPG]

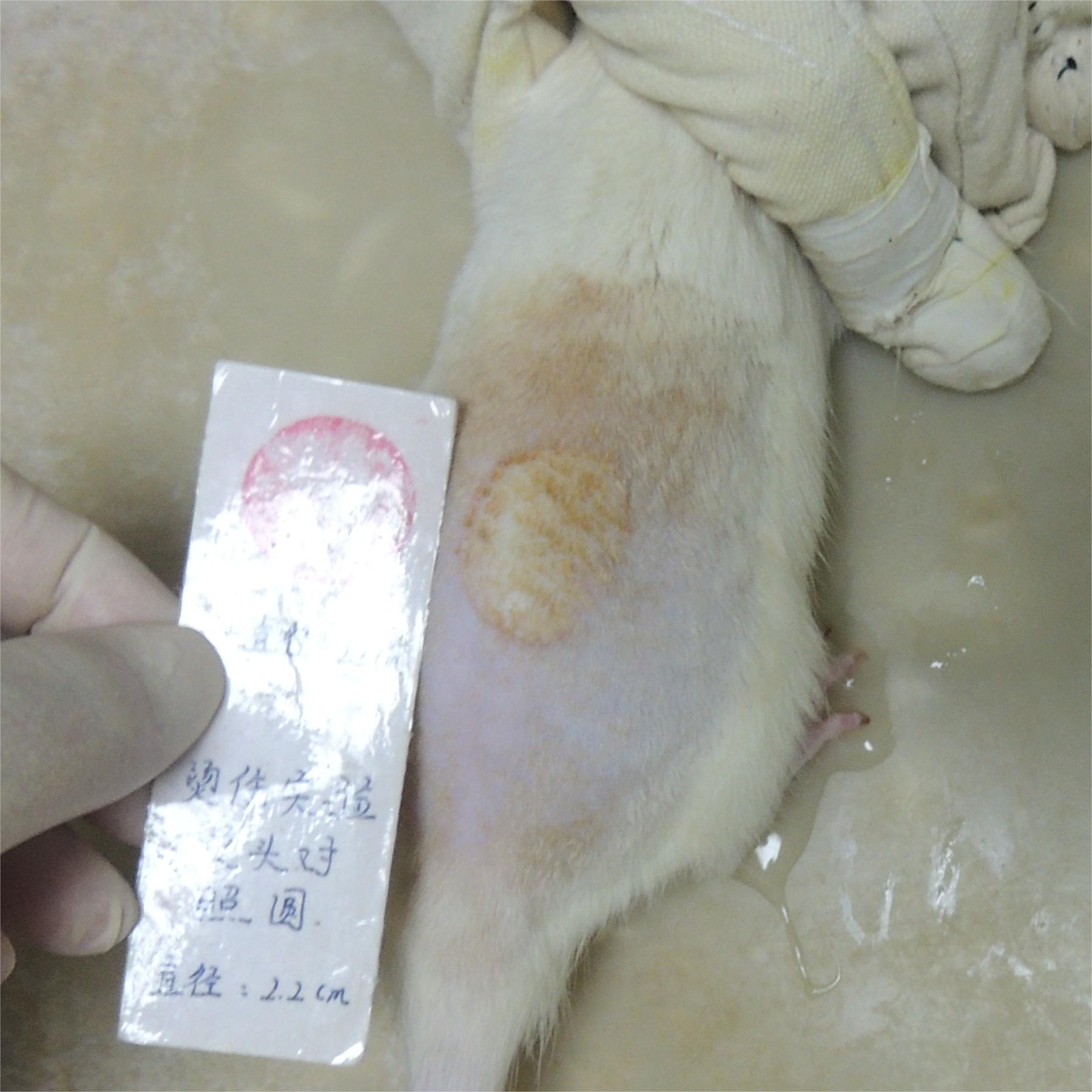

Supplement: Supplementary file 9 [file DataSheet6.ZIP › Figure 6/Photographs of wound/3d/Negative.JPG]

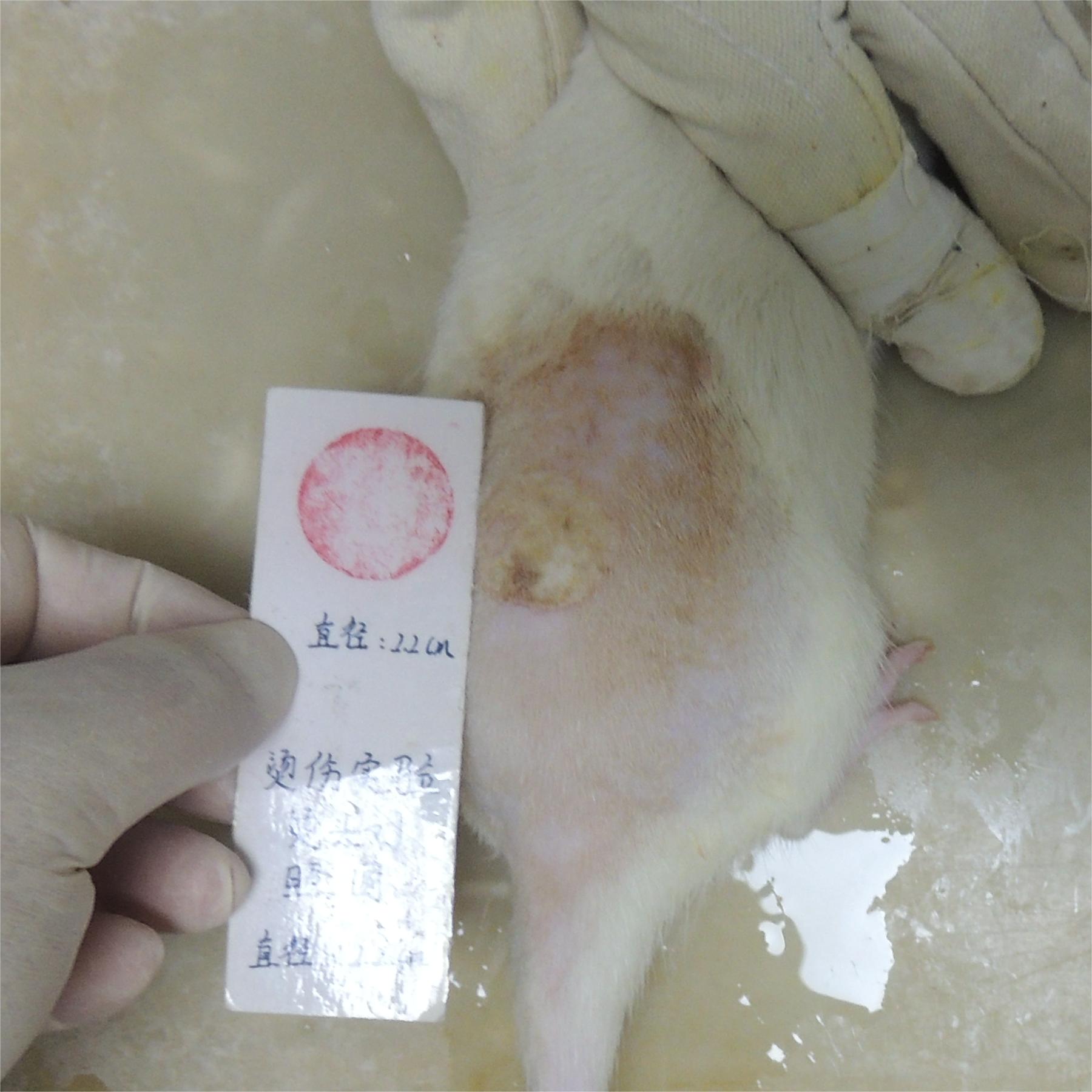

Supplement: Supplementary file 9 [file DataSheet6.ZIP › Figure 6/Photographs of wound/3d/Positive.JPG]

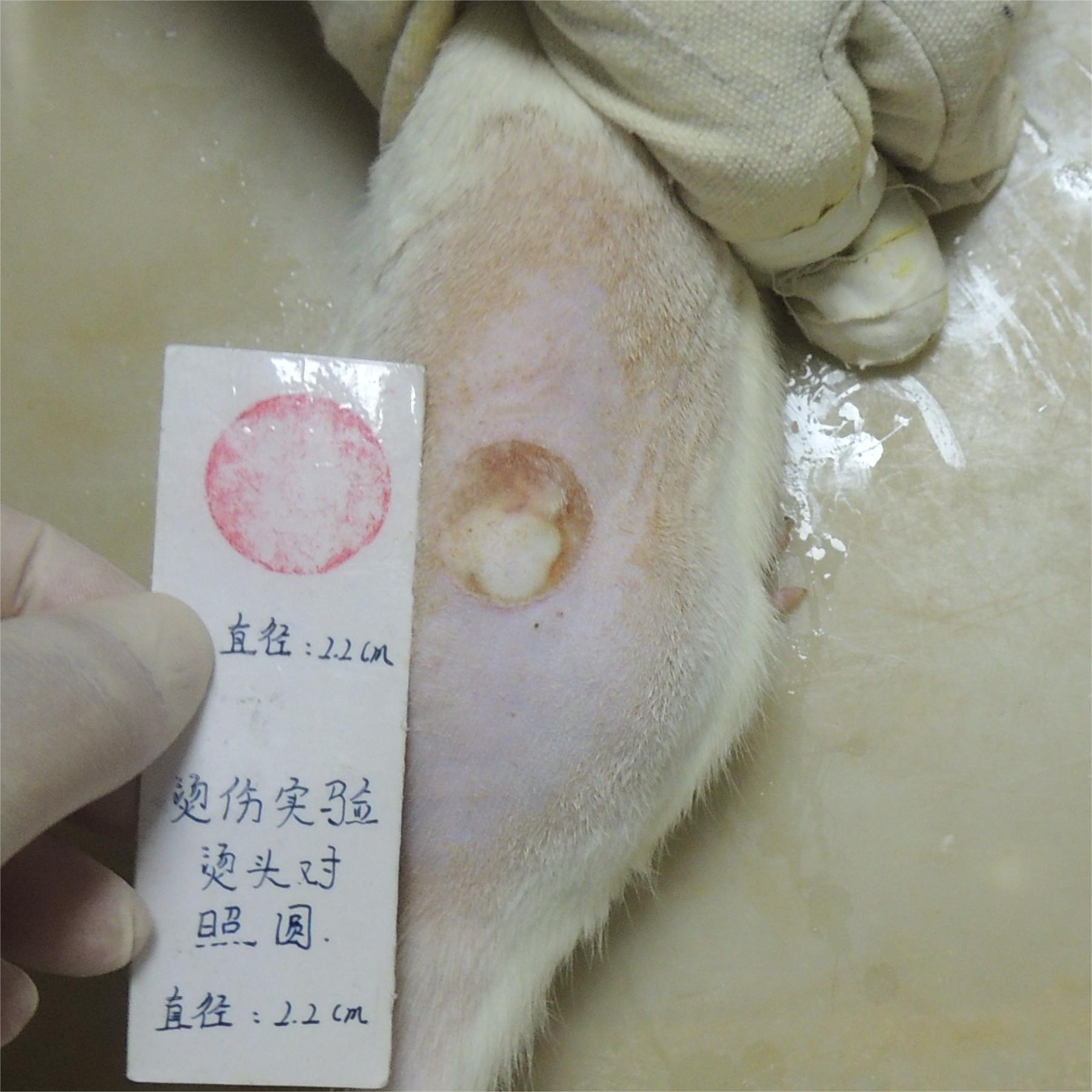

Supplement: Supplementary file 9 [file DataSheet6.ZIP › Figure 6/Photographs of wound/3d/hbFGF.JPG]

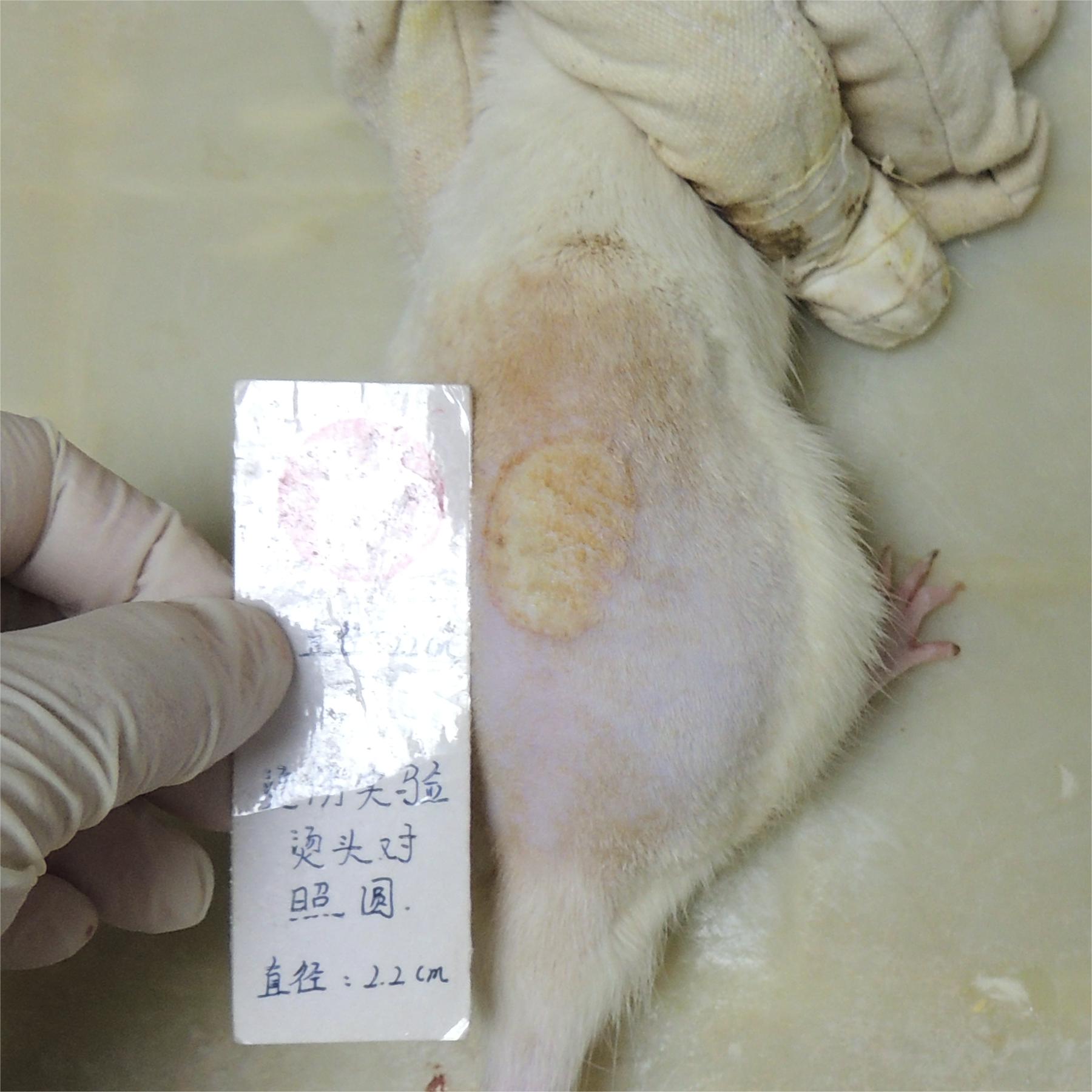

Supplement: Supplementary file 9 [file DataSheet6.ZIP › Figure 6/Photographs of wound/5d/Negative.JPG]

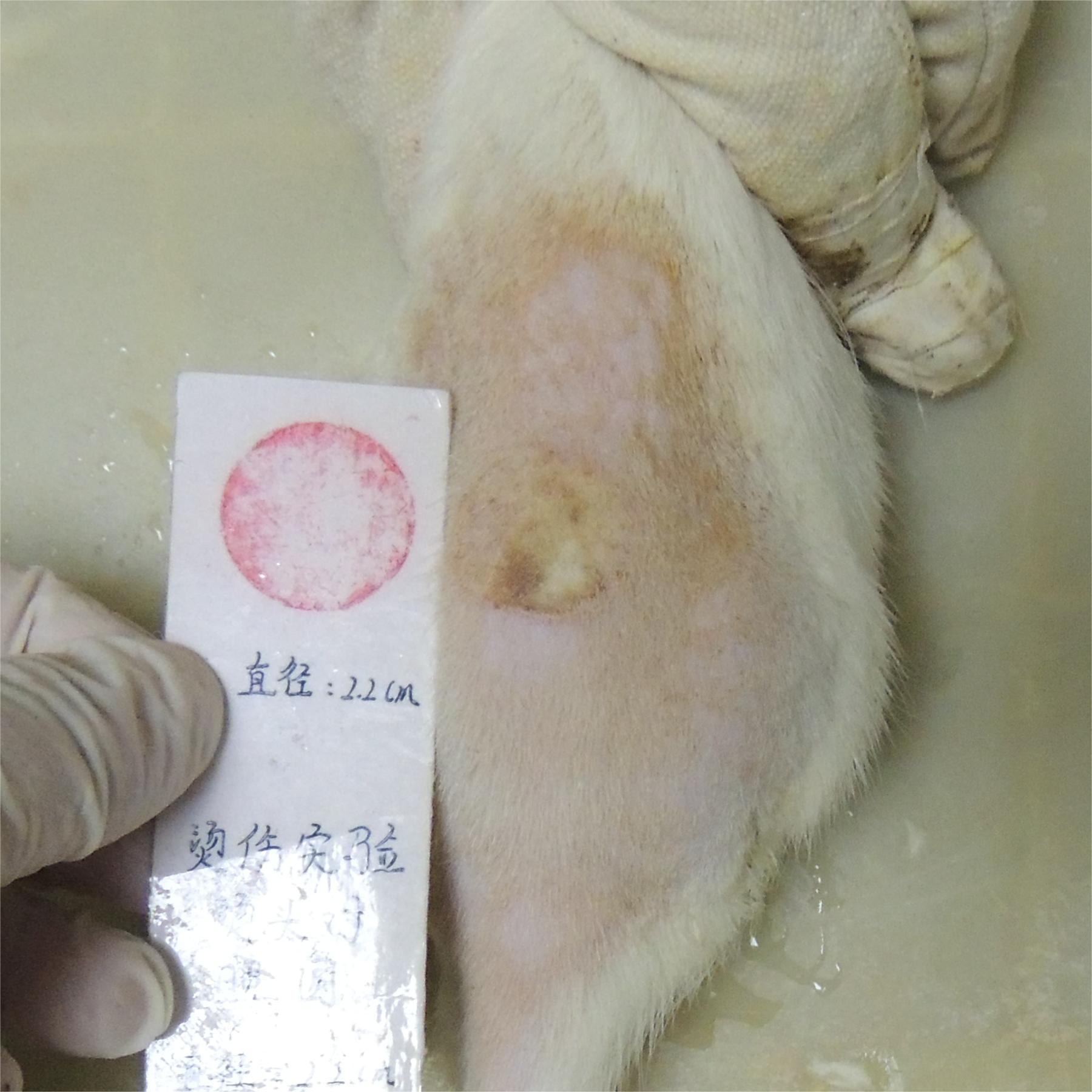

Supplement: Supplementary file 9 [file DataSheet6.ZIP › Figure 6/Photographs of wound/5d/Positive.JPG]

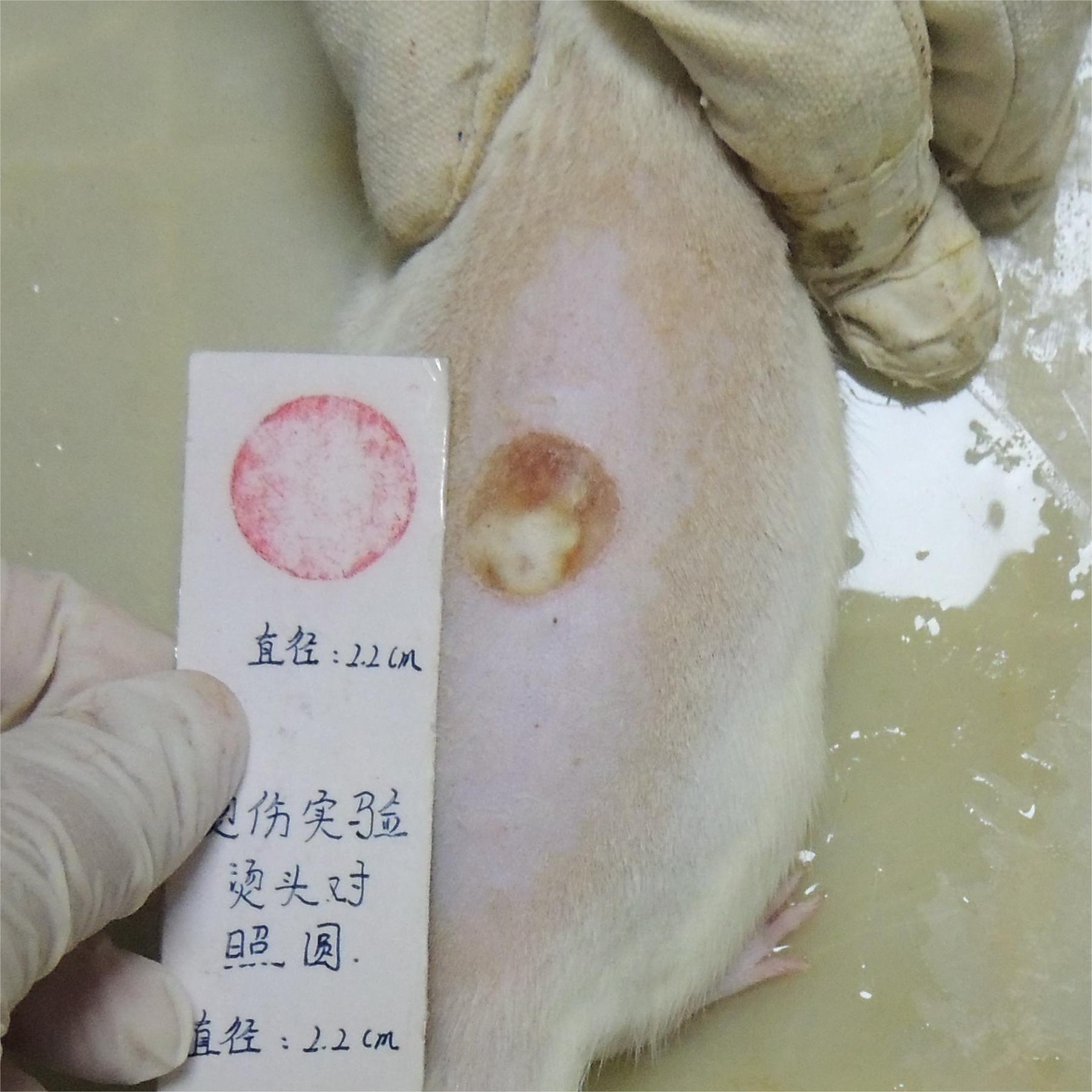

Supplement: Supplementary file 9 [file DataSheet6.ZIP › Figure 6/Photographs of wound/5d/hbFGF.JPG]

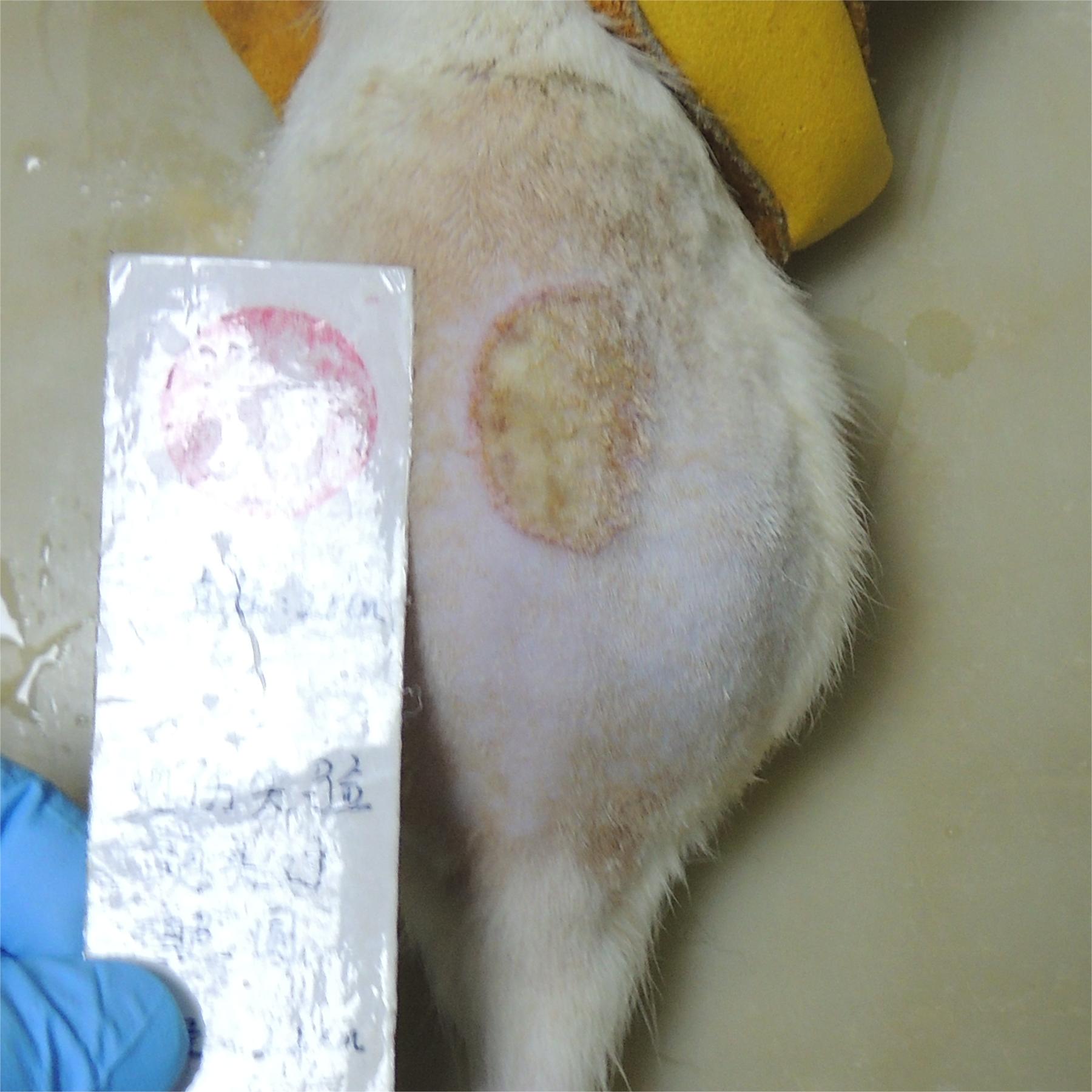

Supplement: Supplementary file 9 [file DataSheet6.ZIP › Figure 6/Photographs of wound/7d/Negative.JPG]

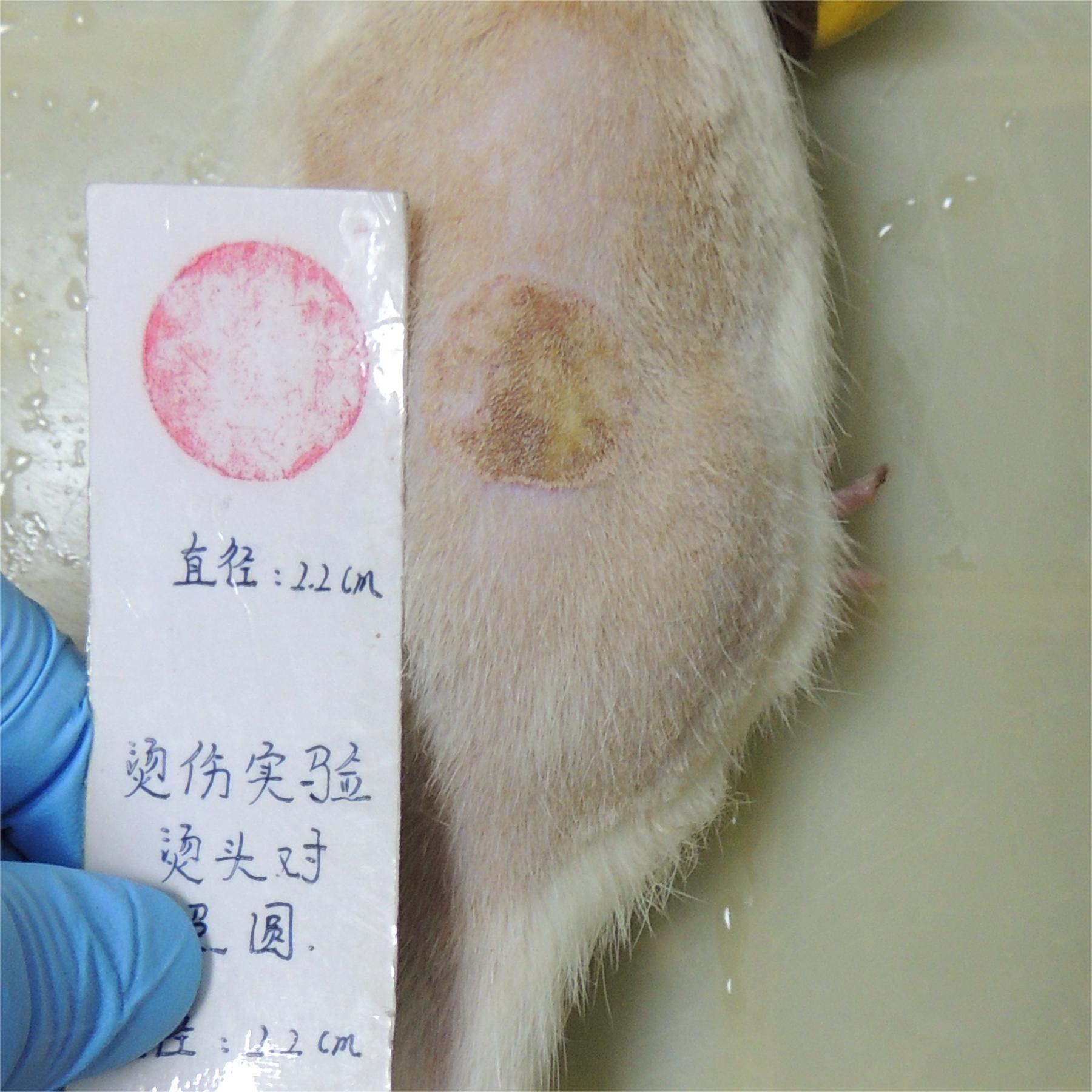

Supplement: Supplementary file 9 [file DataSheet6.ZIP › Figure 6/Photographs of wound/7d/Positive.JPG]

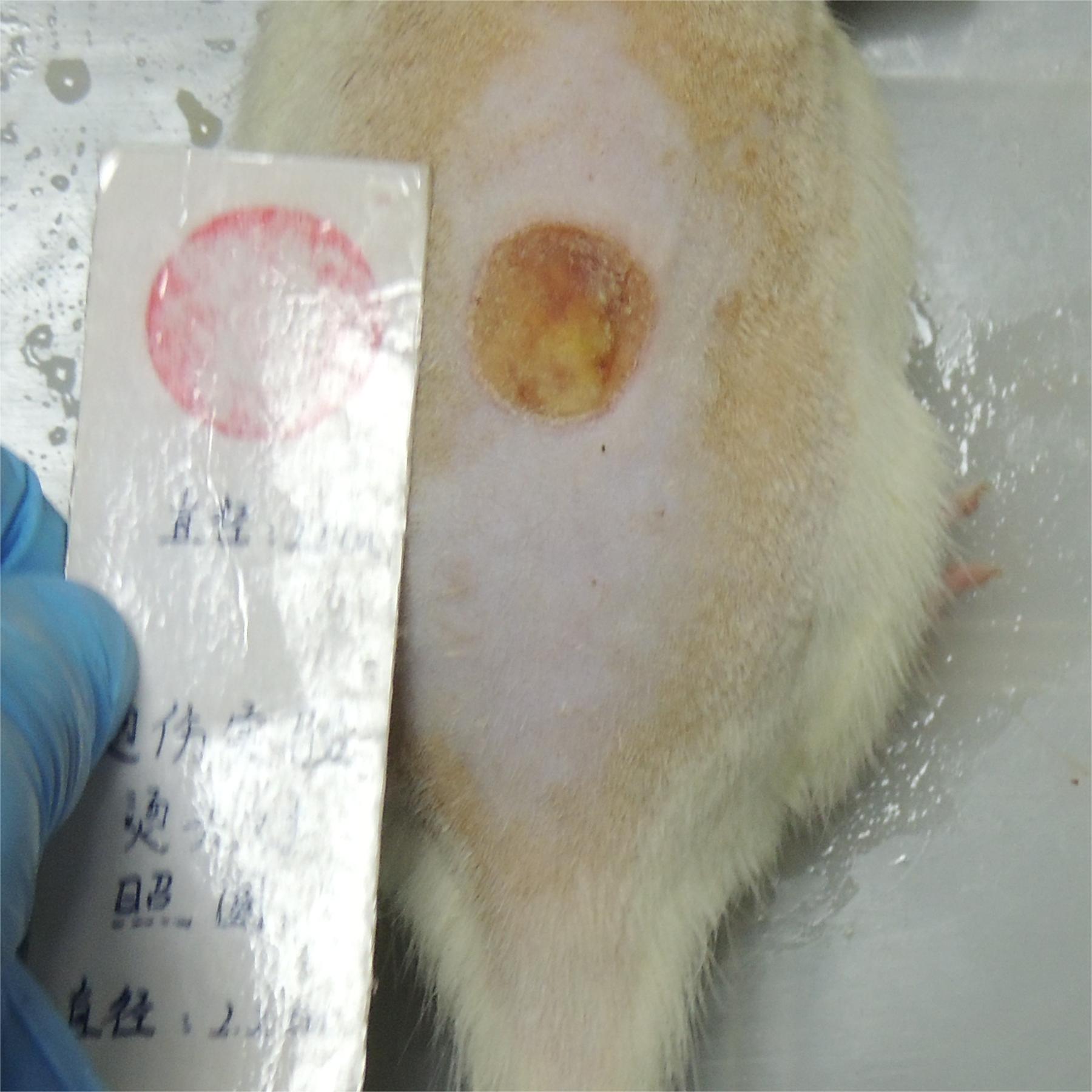

Supplement: Supplementary file 9 [file DataSheet6.ZIP › Figure 6/Photographs of wound/7d/hbFGF.JPG]

## Slide 1
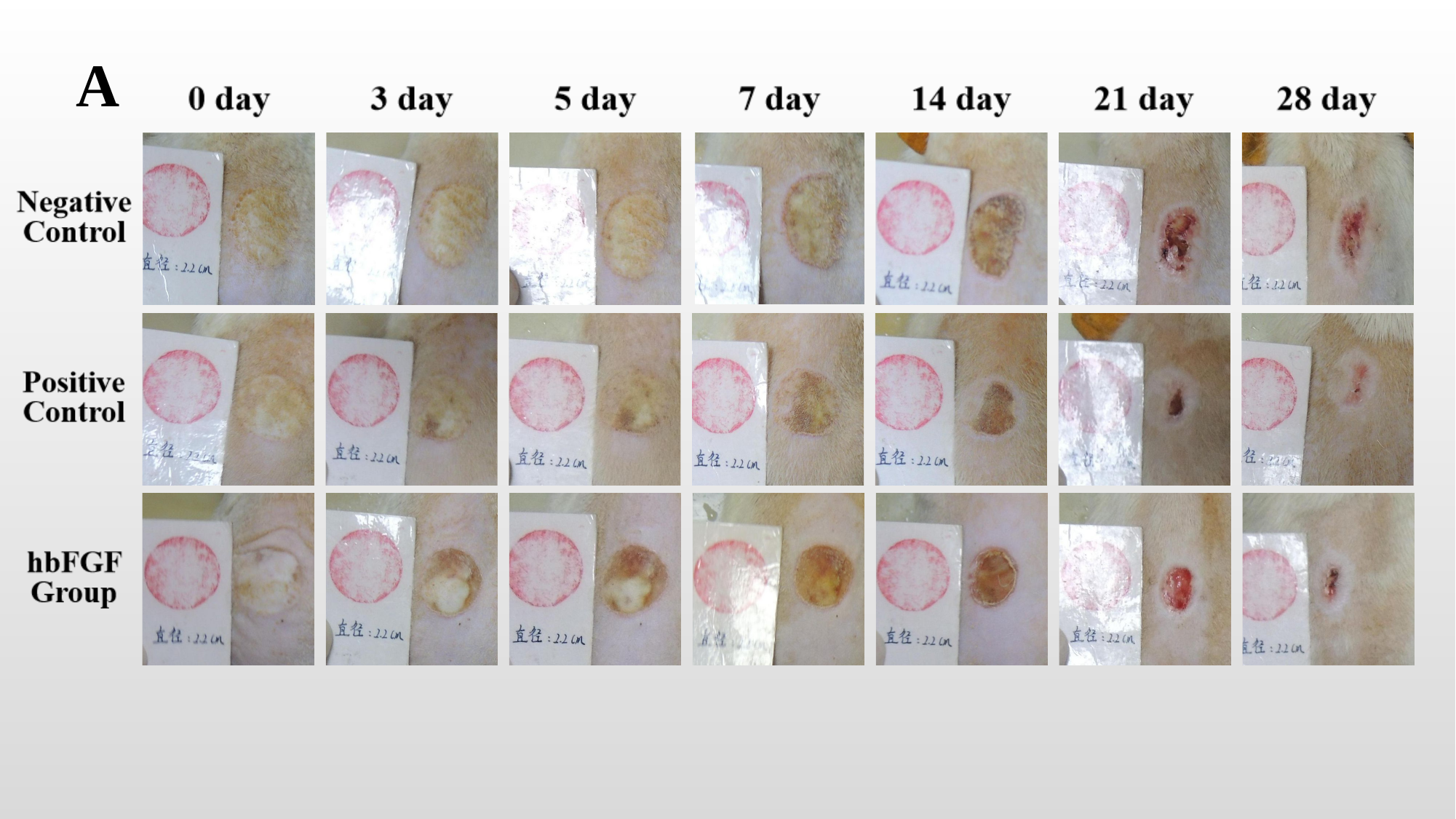

A

Supplement: Supplementary file 9 [file DataSheet6.ZIP › Figure 6/Photographs of wound.pptx]

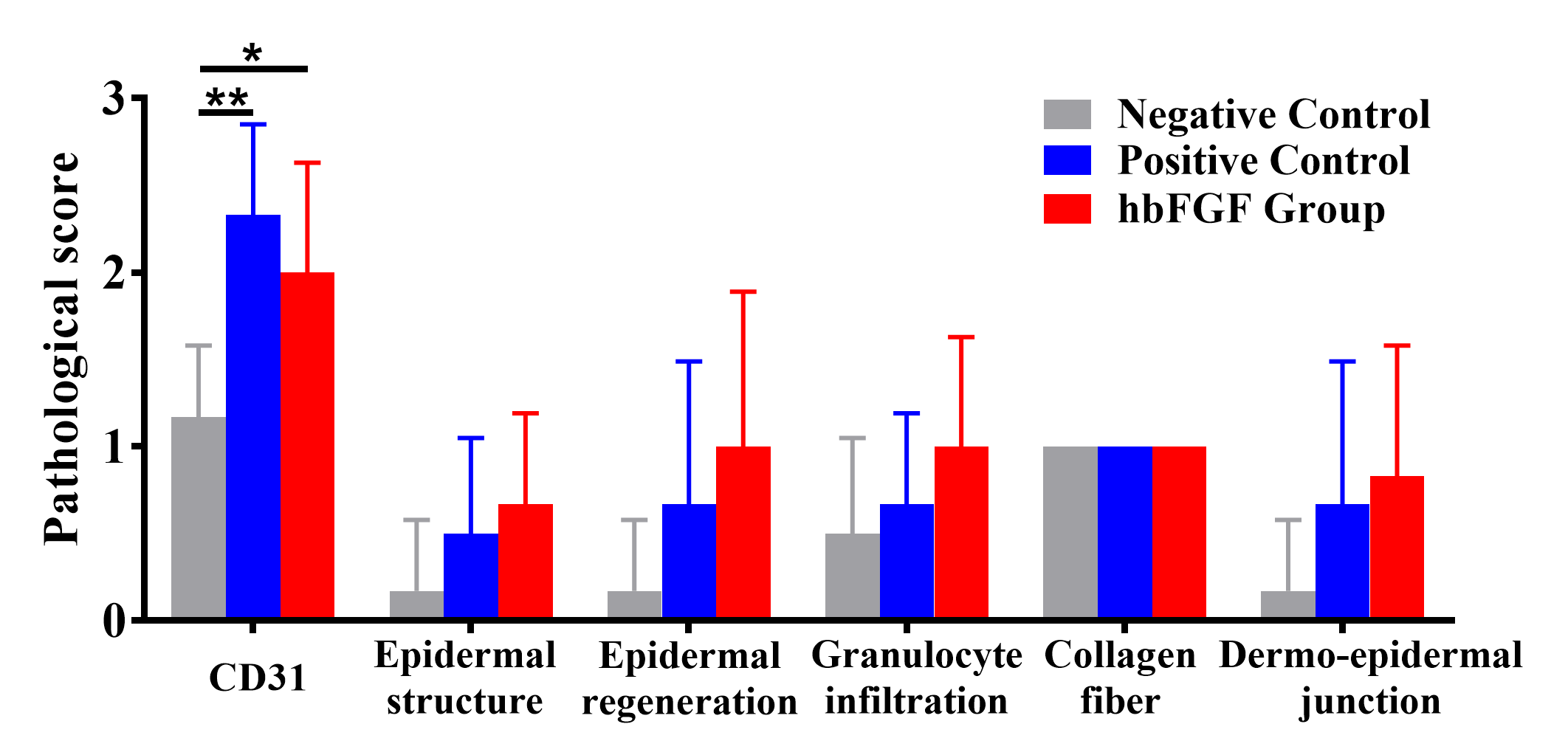

Supplement: Supplementary file 9 [file DataSheet6.ZIP › Figure 6/Score.tif]

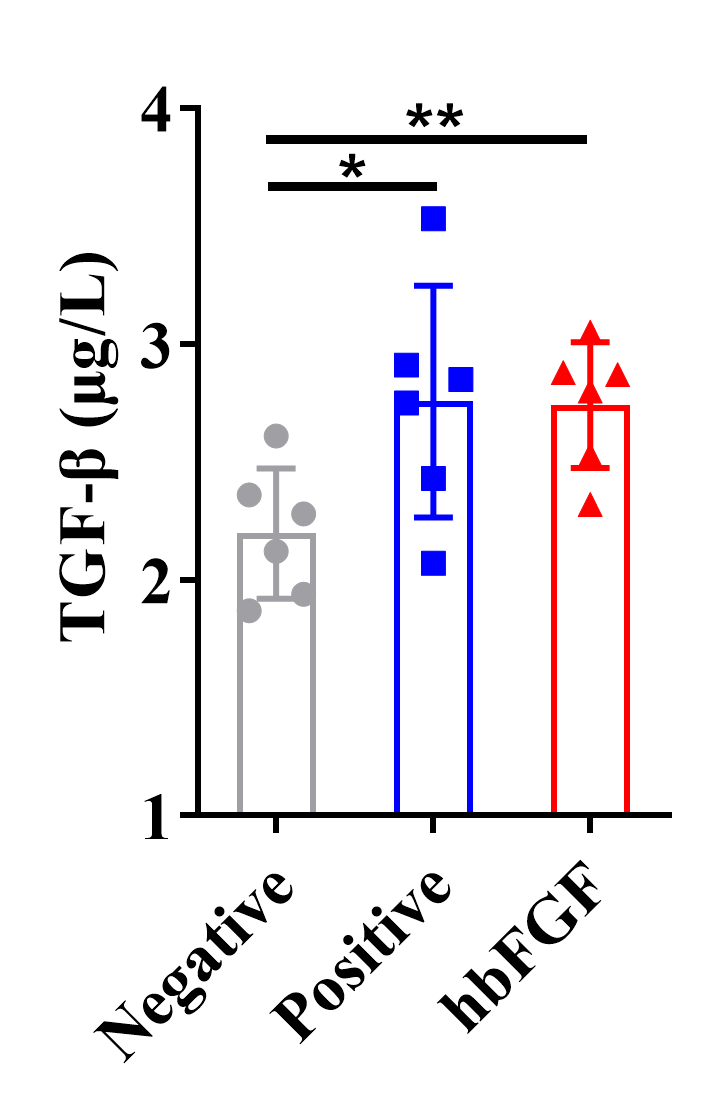

Supplement: Supplementary file 9 [file DataSheet6.ZIP › Figure 6/TGF.tif]

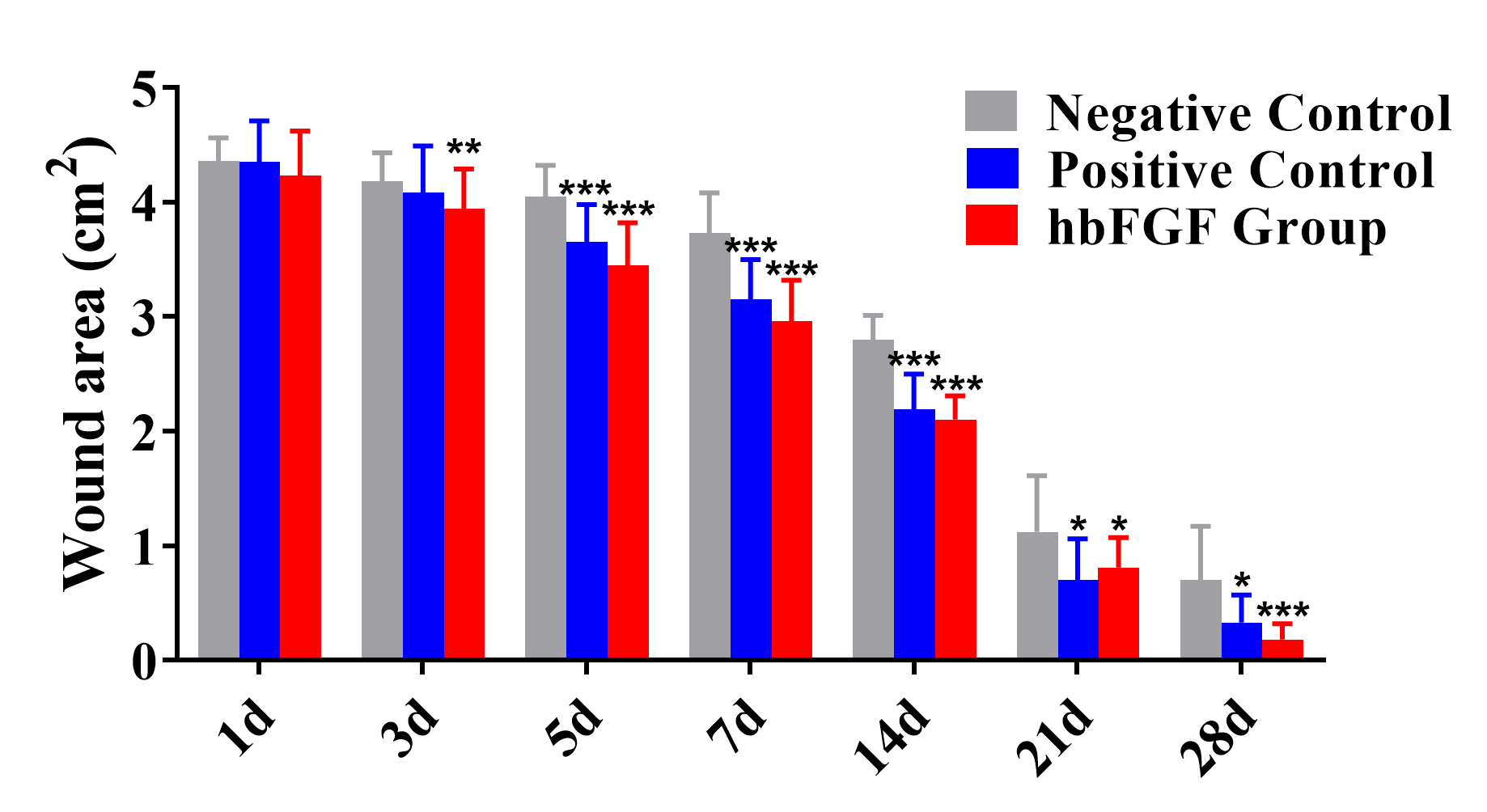

Supplement: Supplementary file 9 [file DataSheet6.ZIP › Figure 6/wound area.tif]

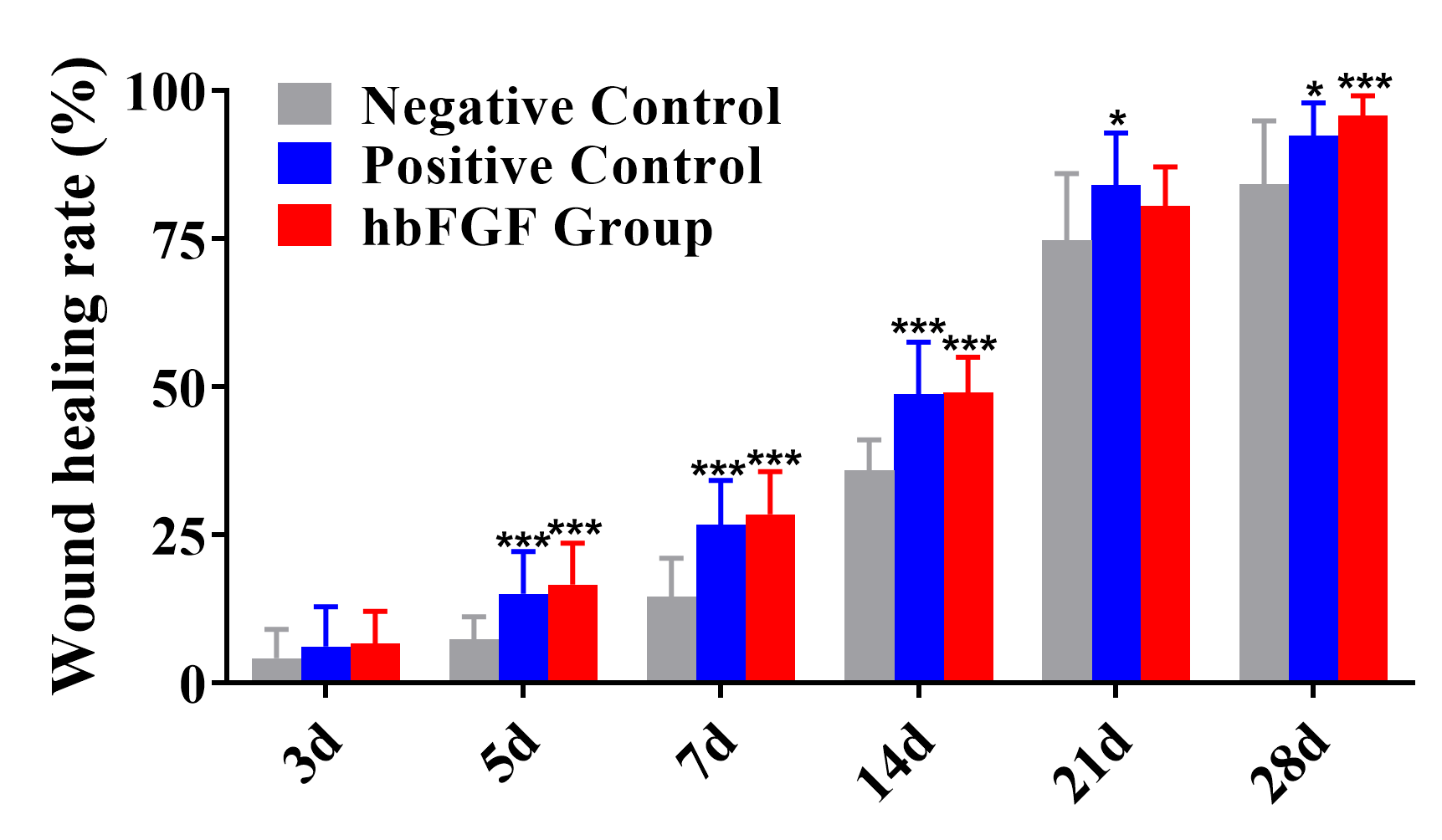

Supplement: Supplementary file 9 [file DataSheet6.ZIP › Figure 6/wound healing rate.tif]

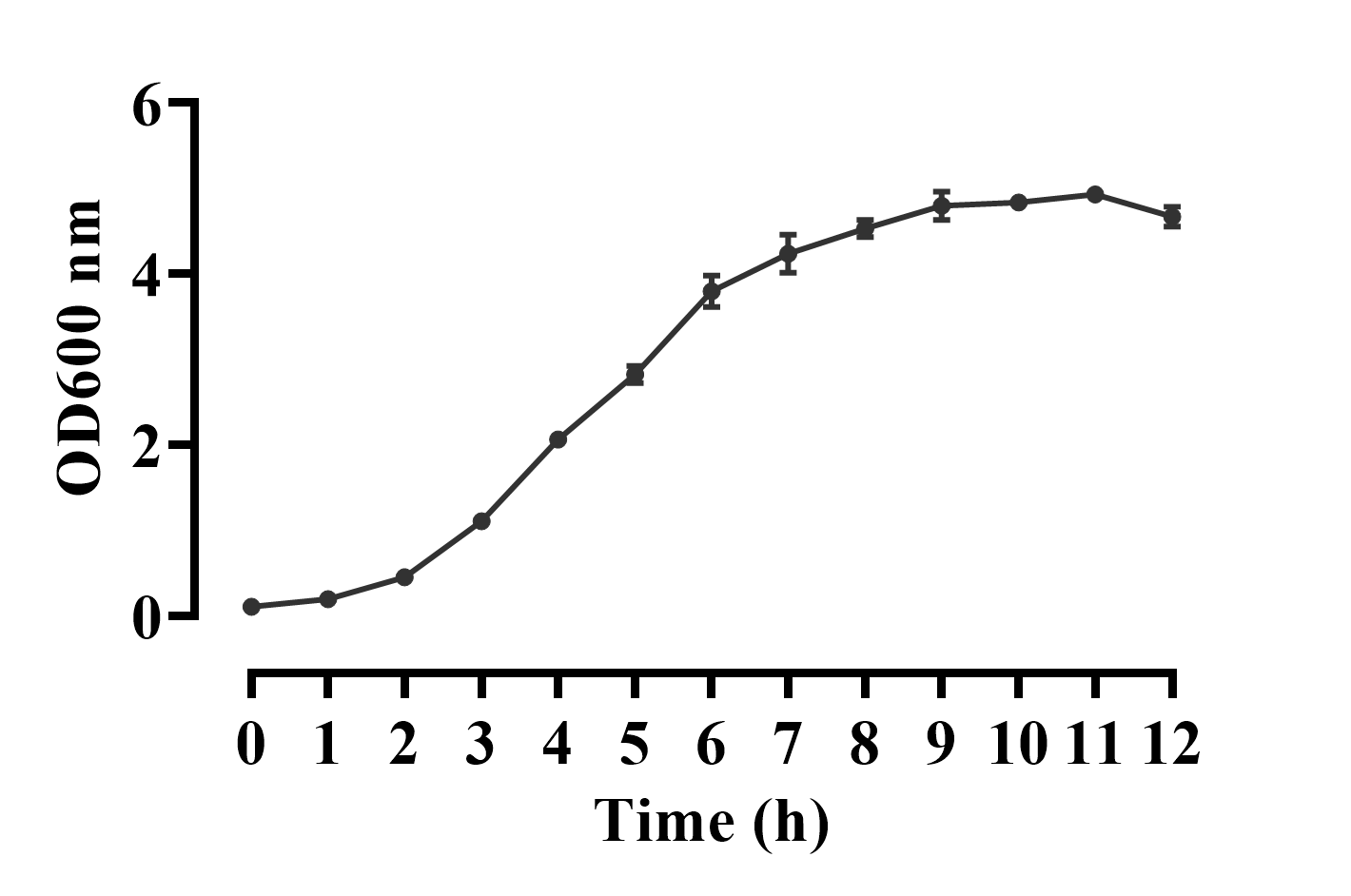

Supplement: Supplementary file 11 [file DataSheet2.ZIP › Figure 2/Fig 2A.tif]

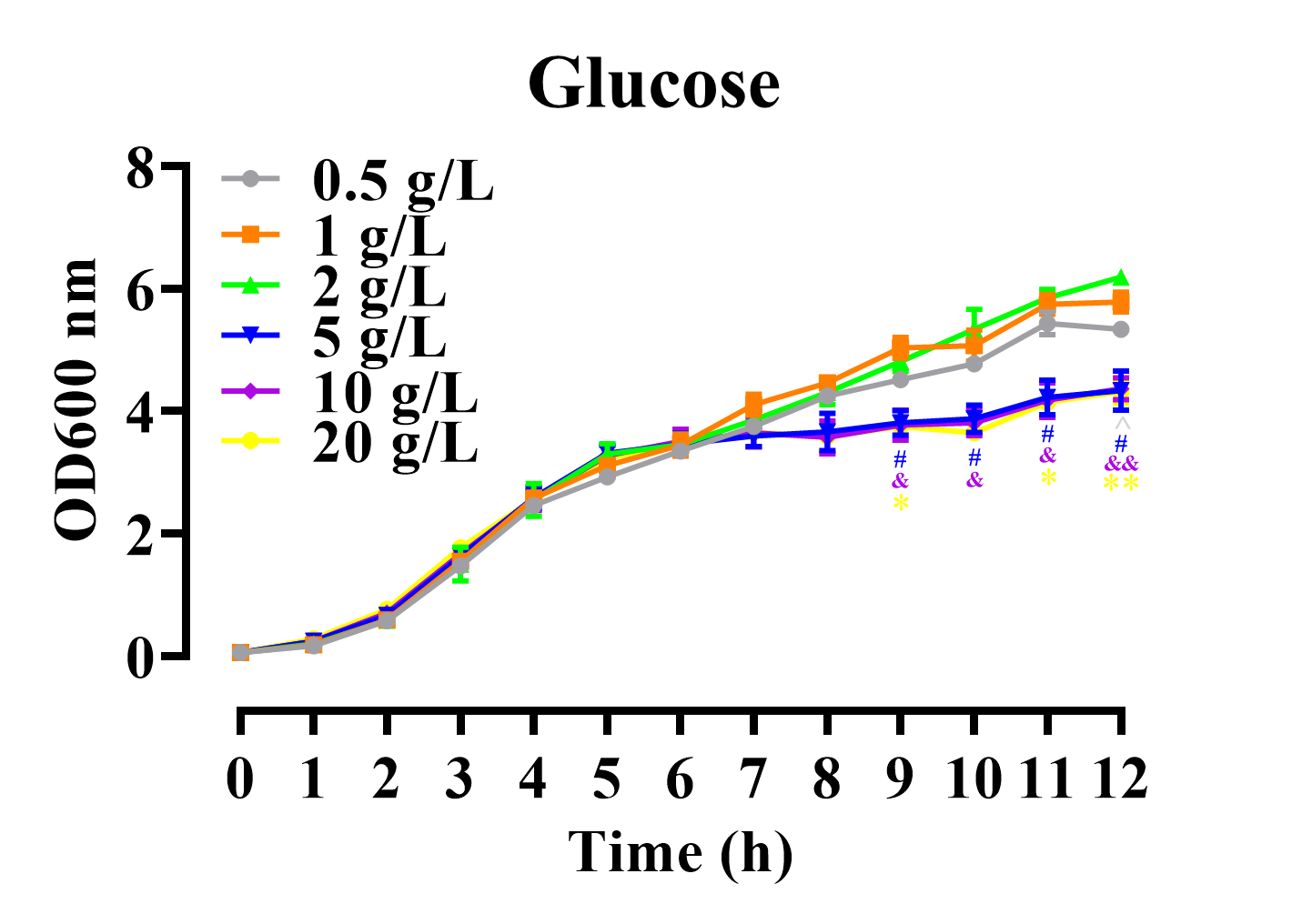

Supplement: Supplementary file 11 [file DataSheet2.ZIP › Figure 2/Fig 2B.tif]

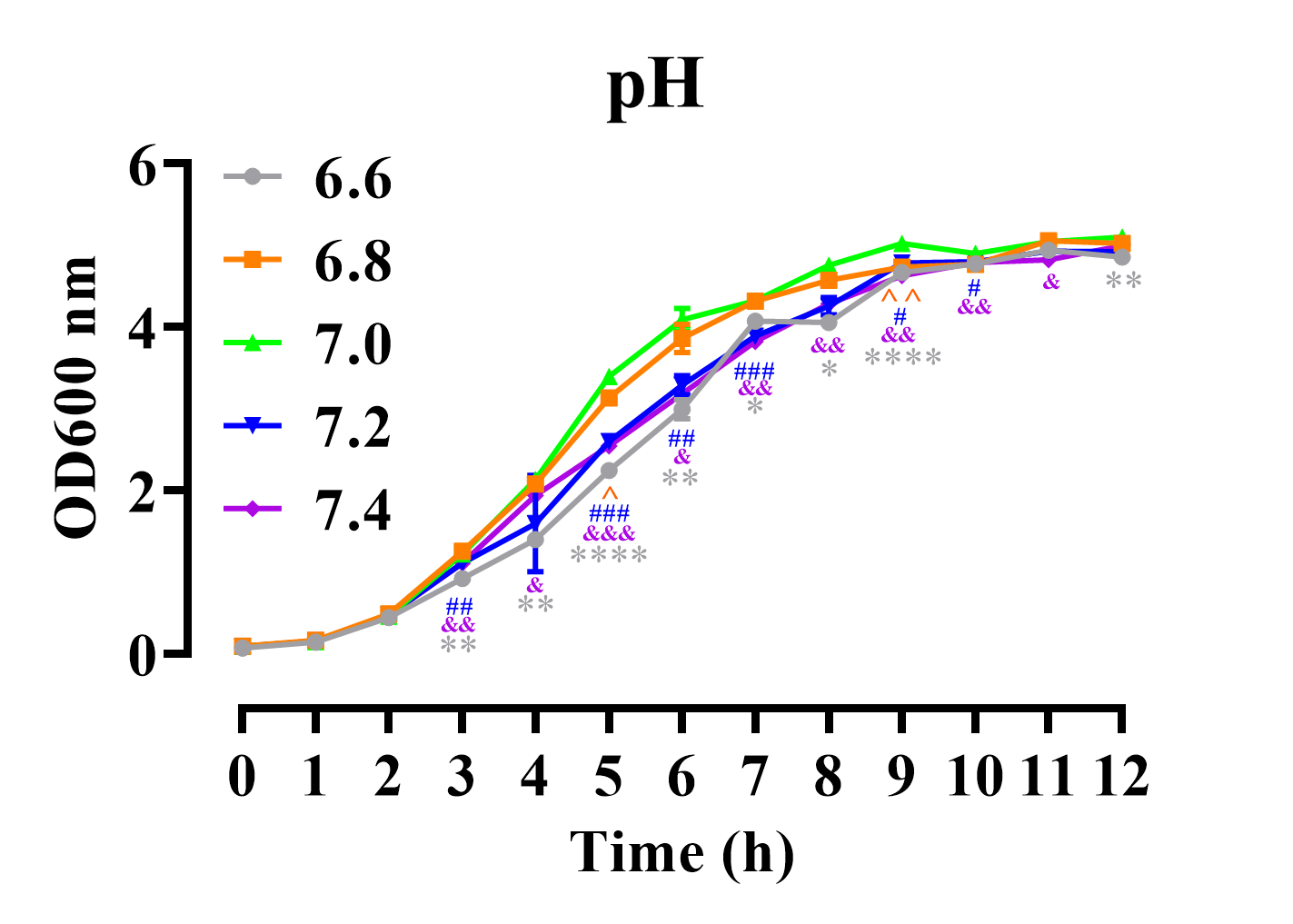

Supplement: Supplementary file 11 [file DataSheet2.ZIP › Figure 2/Fig 2C.tif]

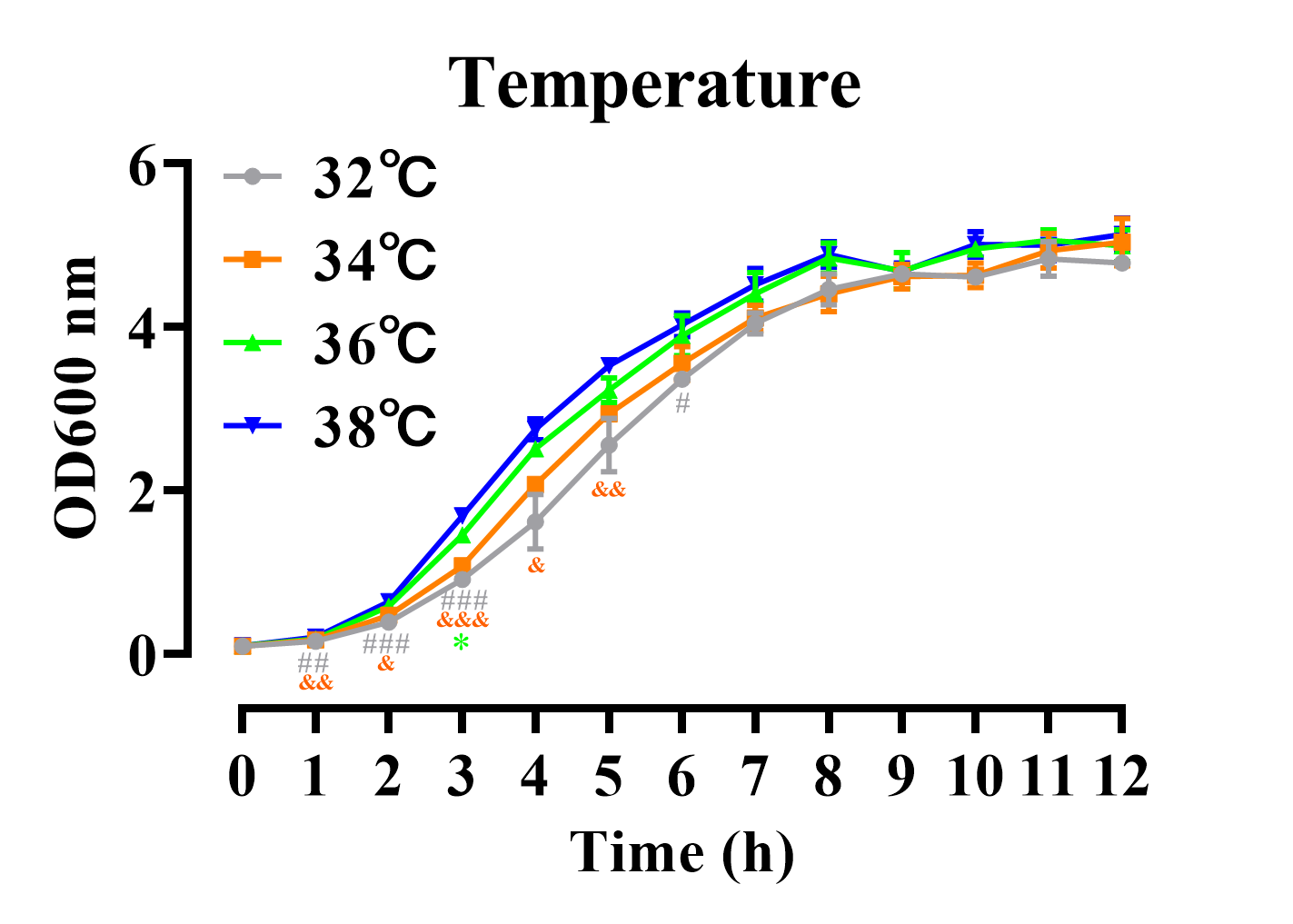

Supplement: Supplementary file 11 [file DataSheet2.ZIP › Figure 2/Fig 2D.tif]

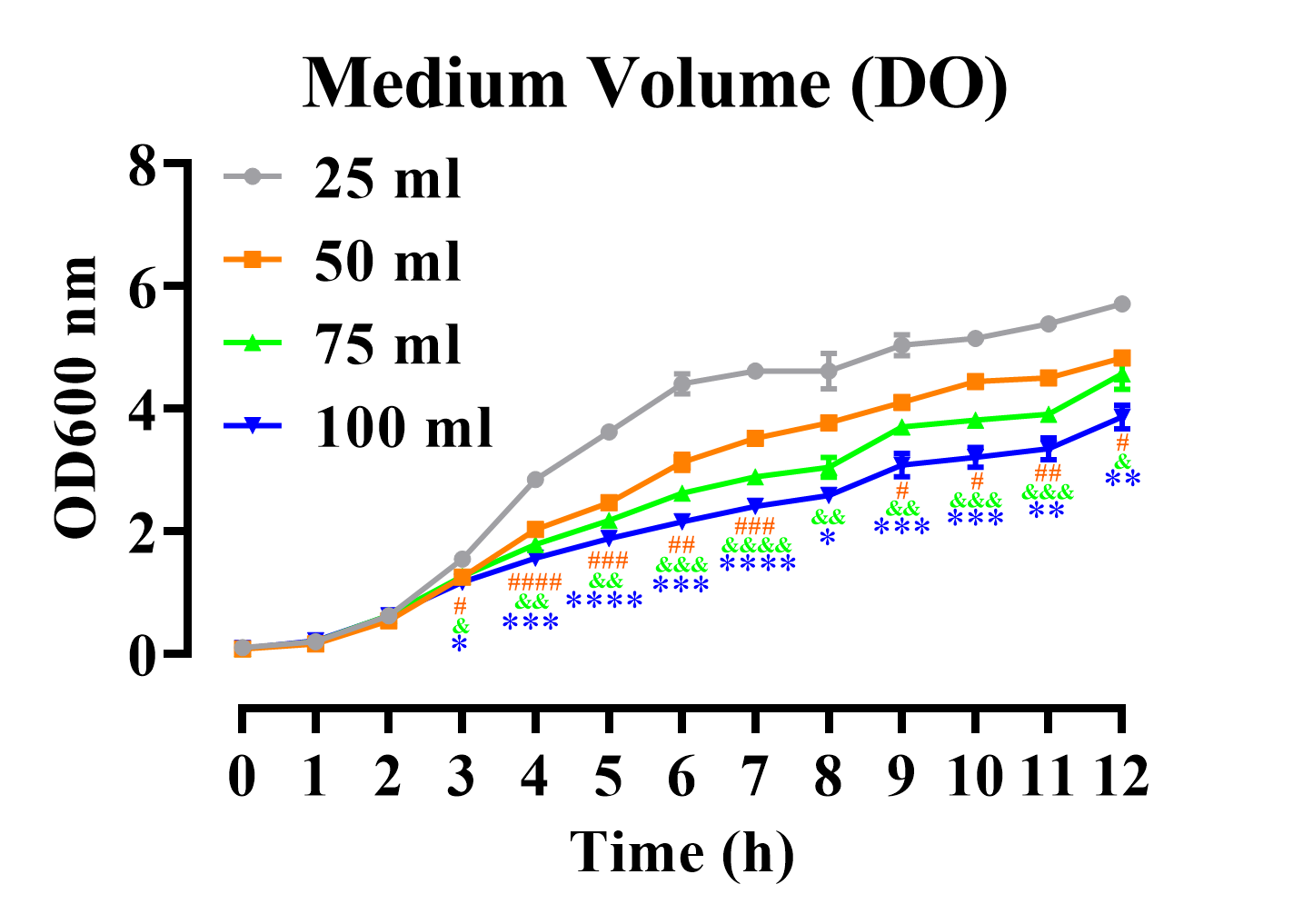

Supplement: Supplementary file 11 [file DataSheet2.ZIP › Figure 2/Fig 2E.tif]

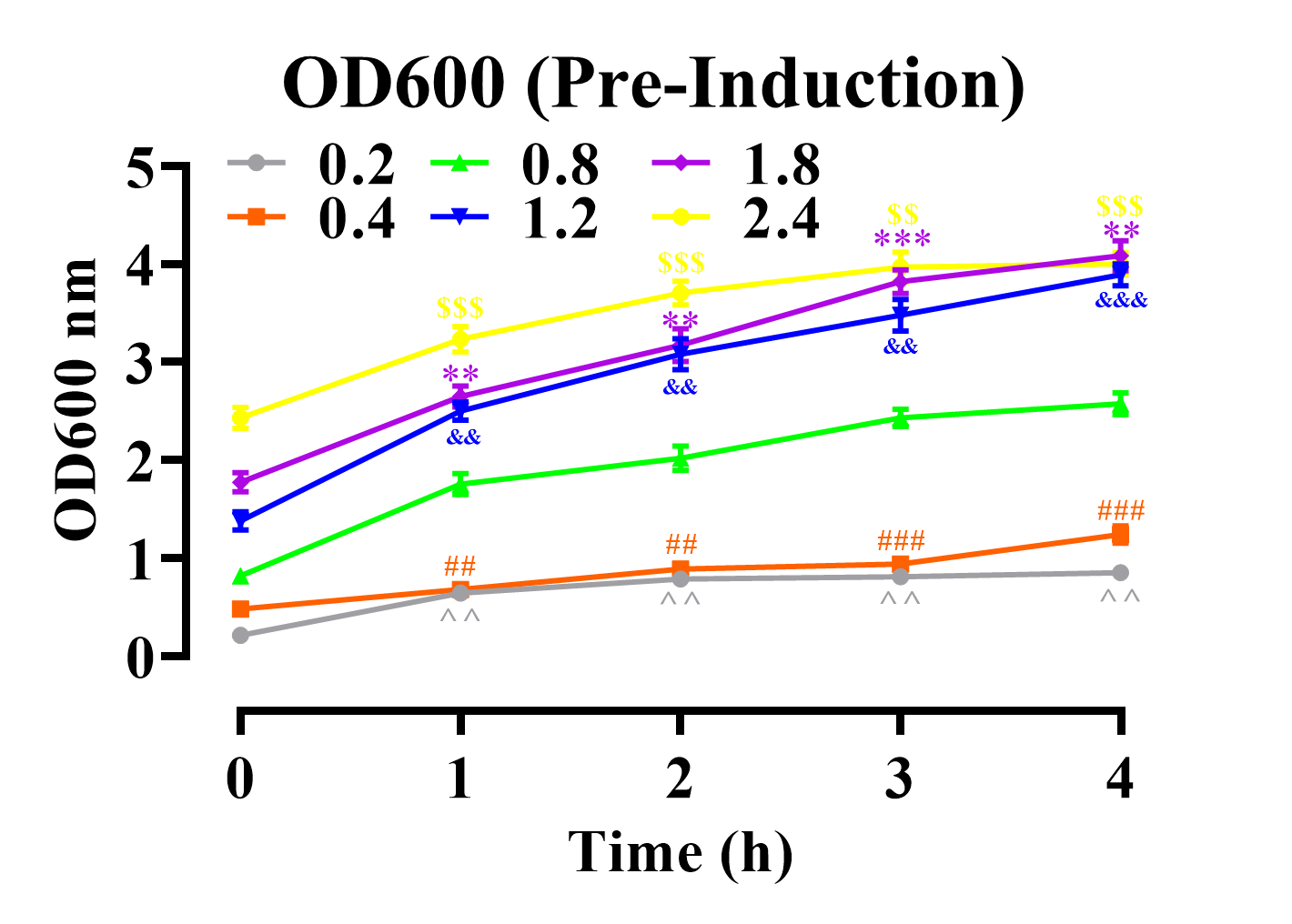

Supplement: Supplementary file 11 [file DataSheet2.ZIP › Figure 2/Fig 2F-1.tif]

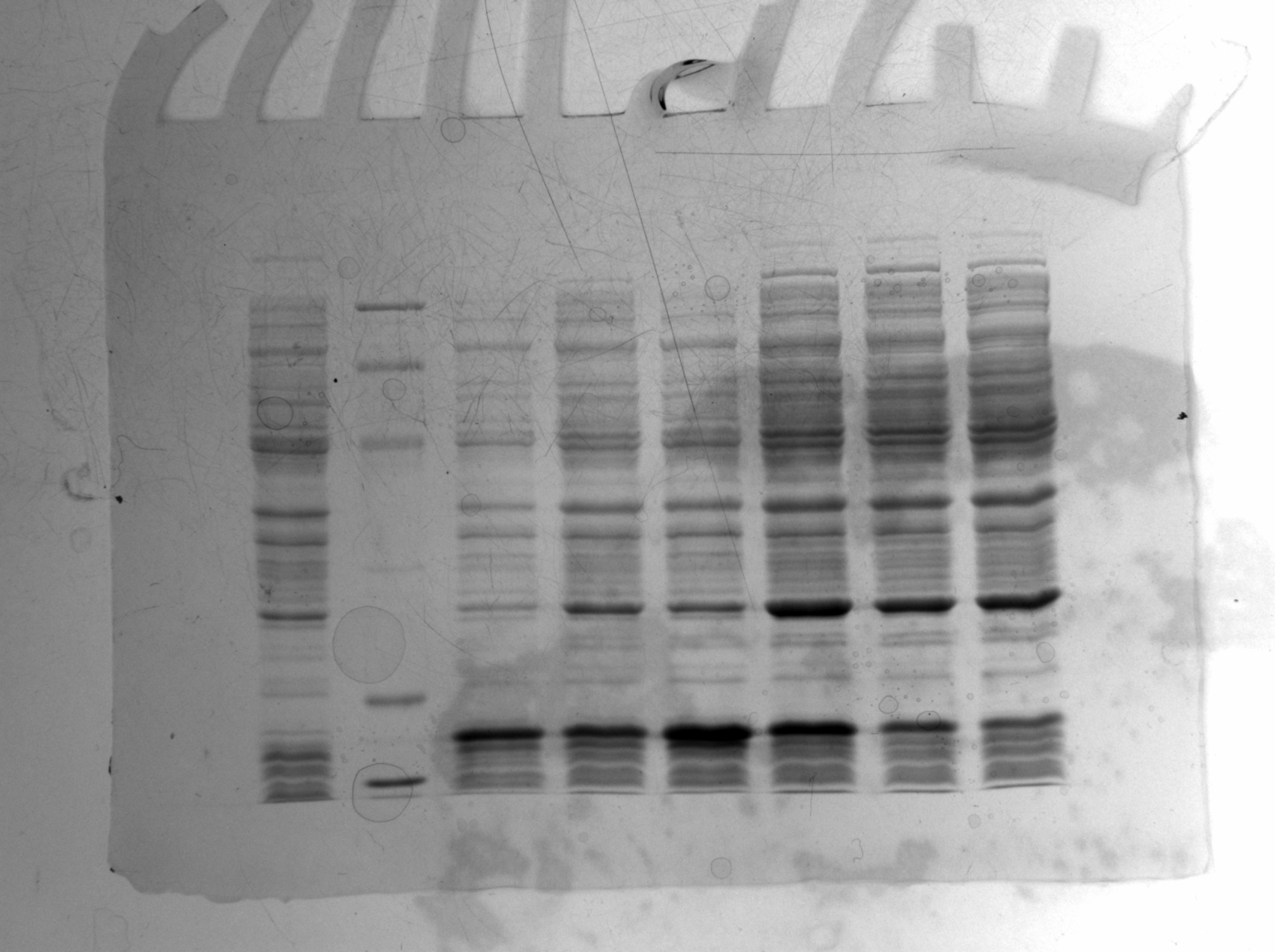

Supplement: Supplementary file 11 [file DataSheet2.ZIP › Figure 2/Fig 2F-2.jpg]

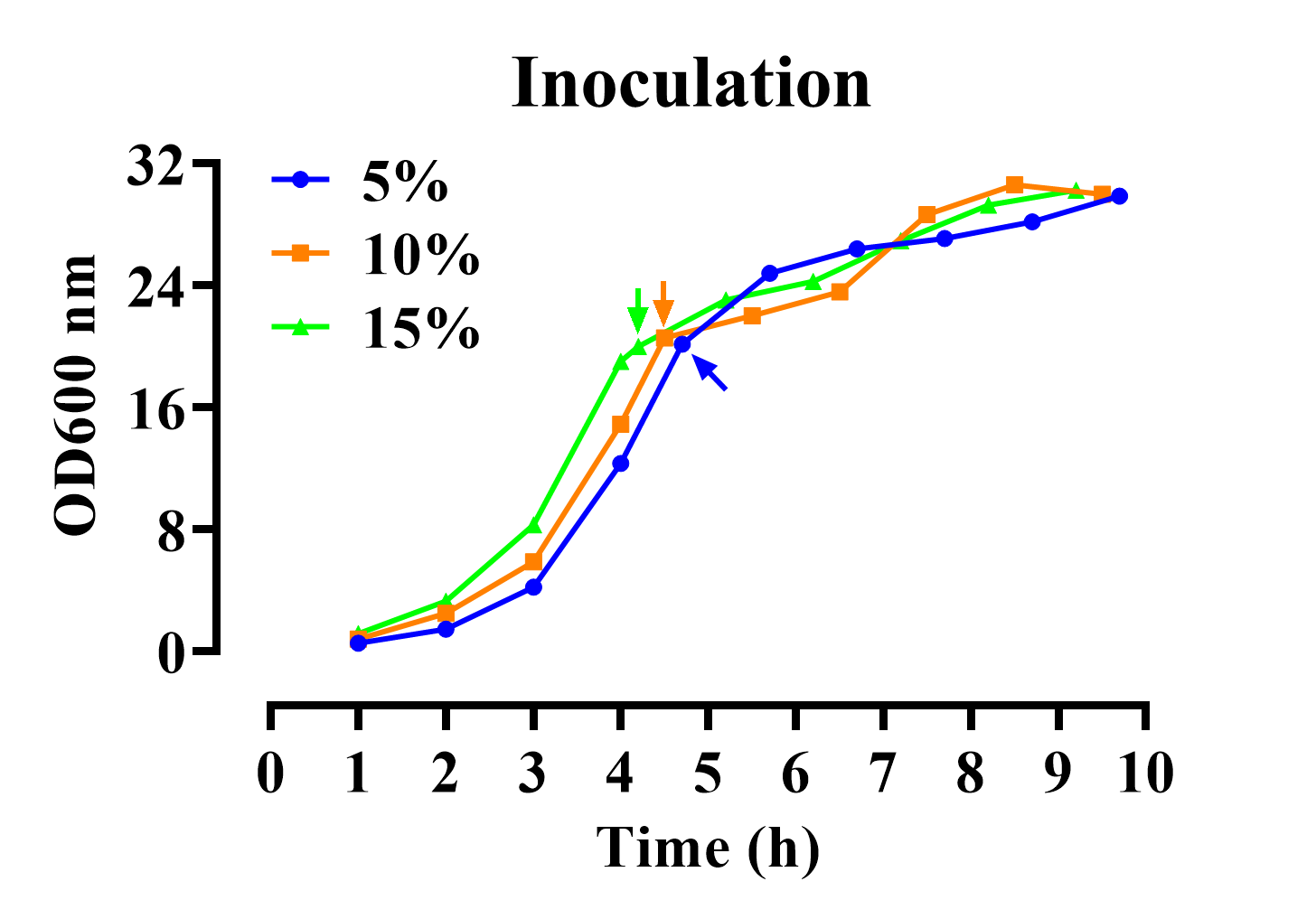

Supplement: Supplementary file 11 [file DataSheet2.ZIP › Figure 2/Fig 2G.tif]

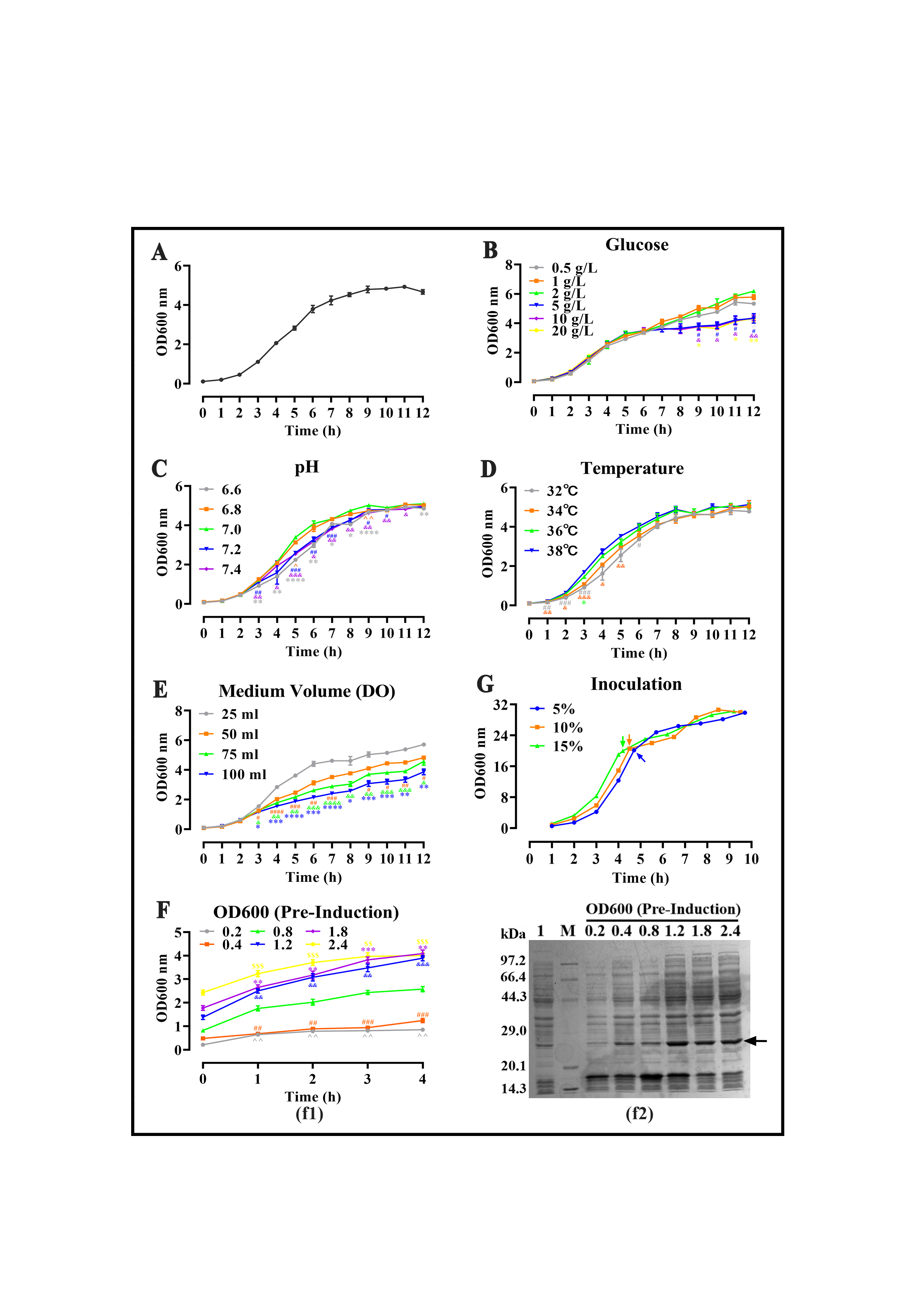

Supplement: Supplementary file 11 [file DataSheet2.ZIP › Figure 2/Figure 2.tif]

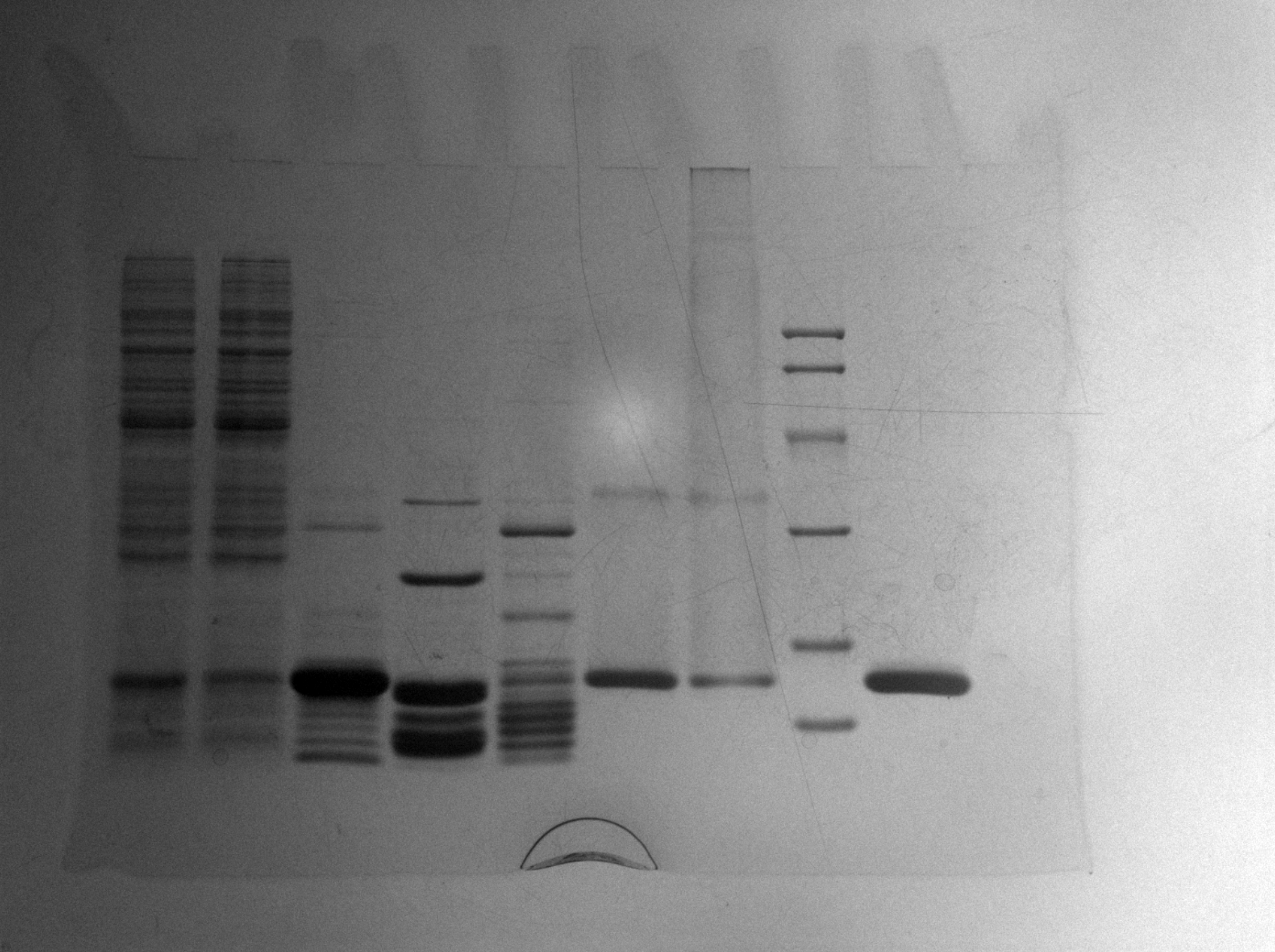

Supplement: Supplementary file 12 [file DataSheet5.ZIP › Figure 5/Fig 5A.tif]

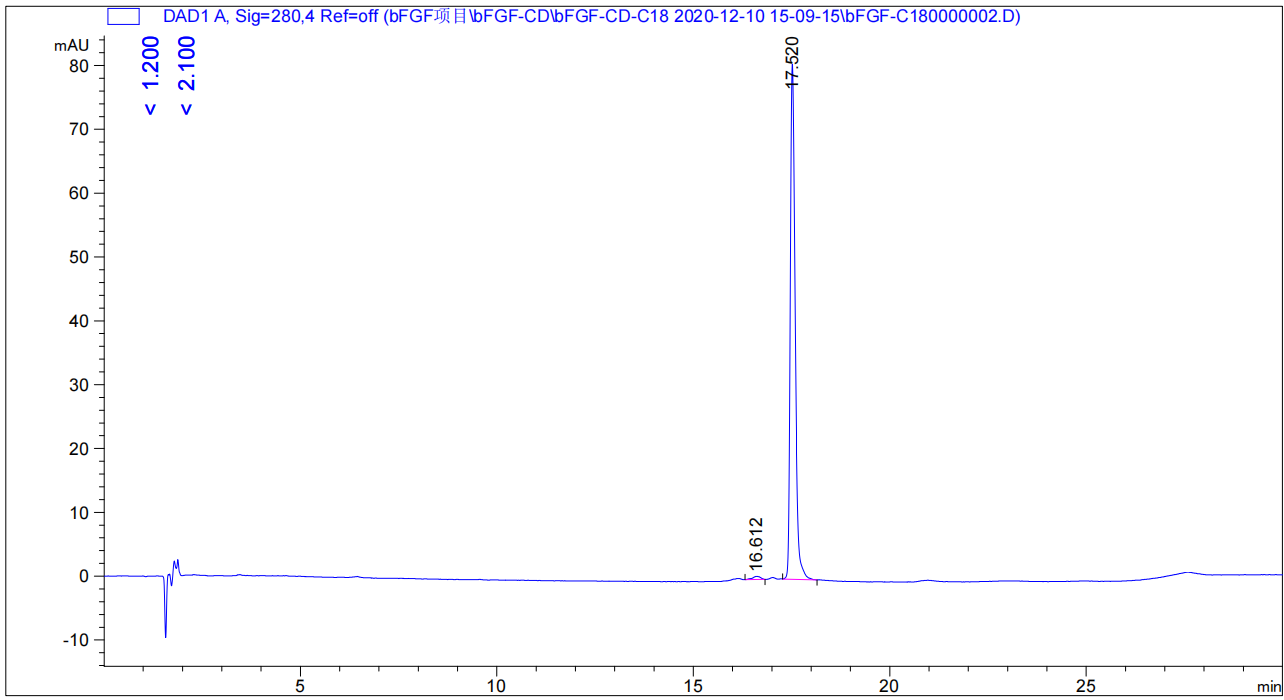

Supplement: Supplementary file 12 [file DataSheet5.ZIP › Figure 5/Fig 5B.tif]

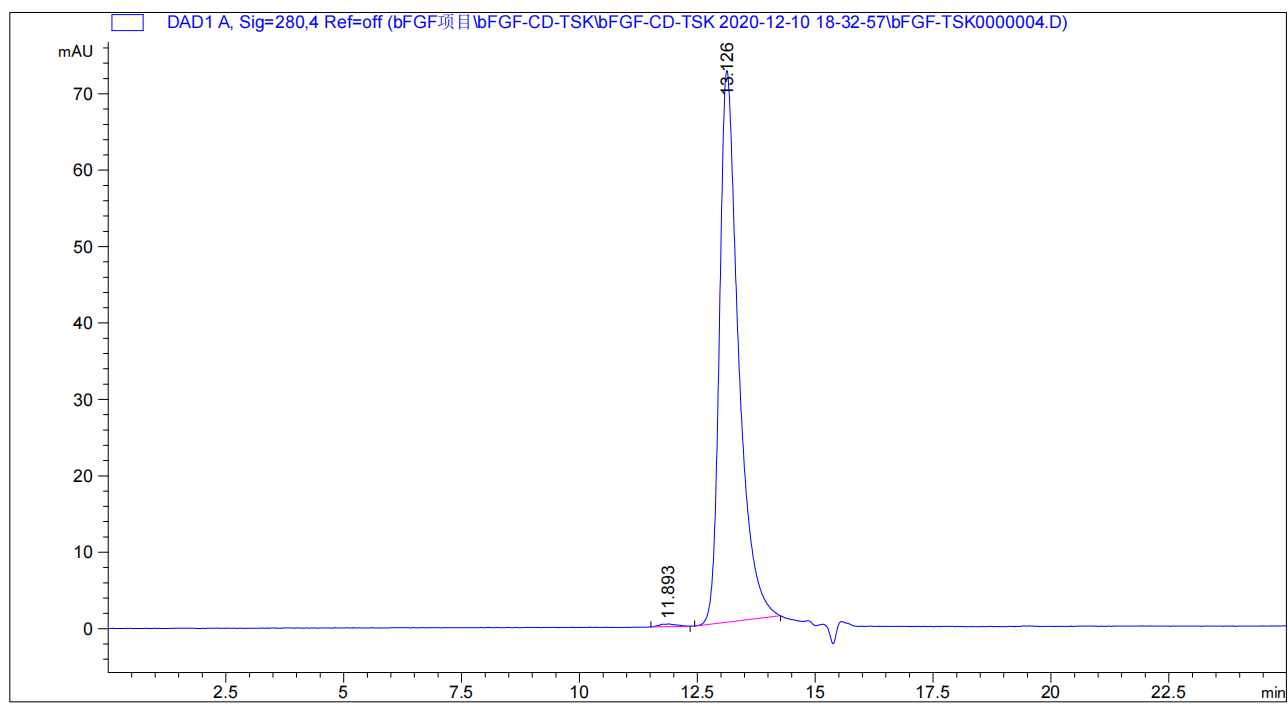

Supplement: Supplementary file 12 [file DataSheet5.ZIP › Figure 5/Fig 5C.tif]

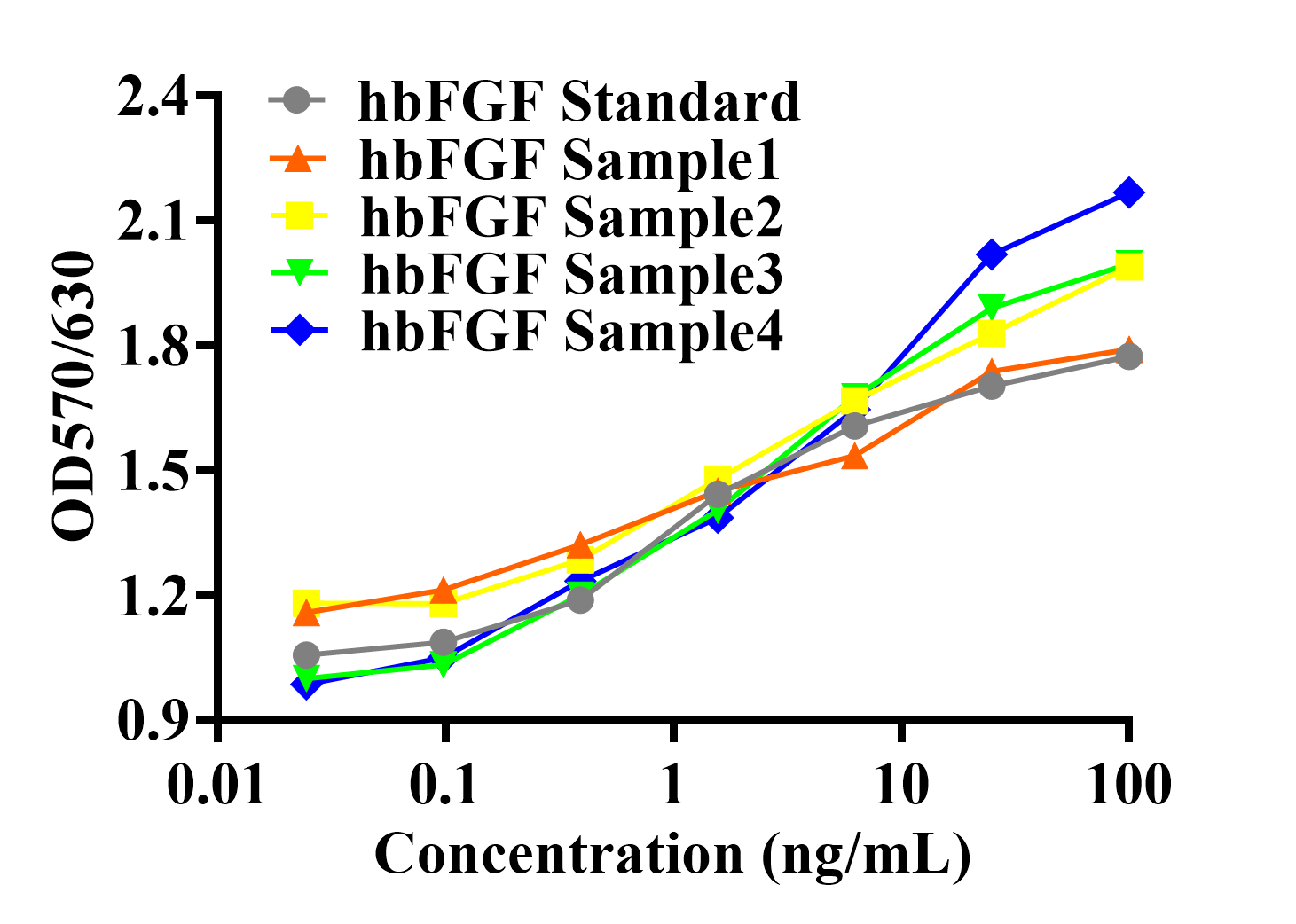

Supplement: Supplementary file 12 [file DataSheet5.ZIP › Figure 5/Fig 5D.tif]

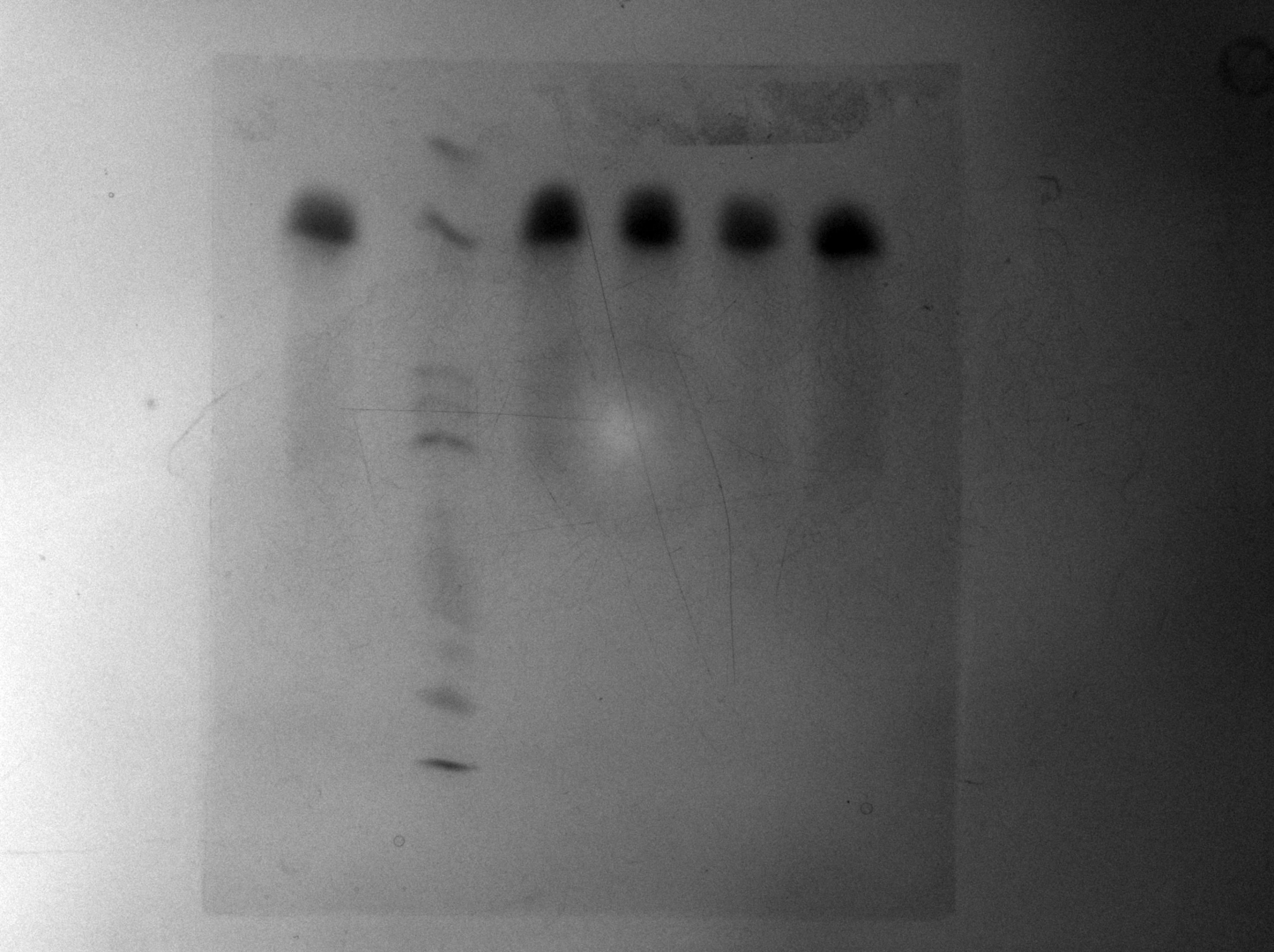

Supplement: Supplementary file 12 [file DataSheet5.ZIP › Figure 5/Fig 5E.tif]

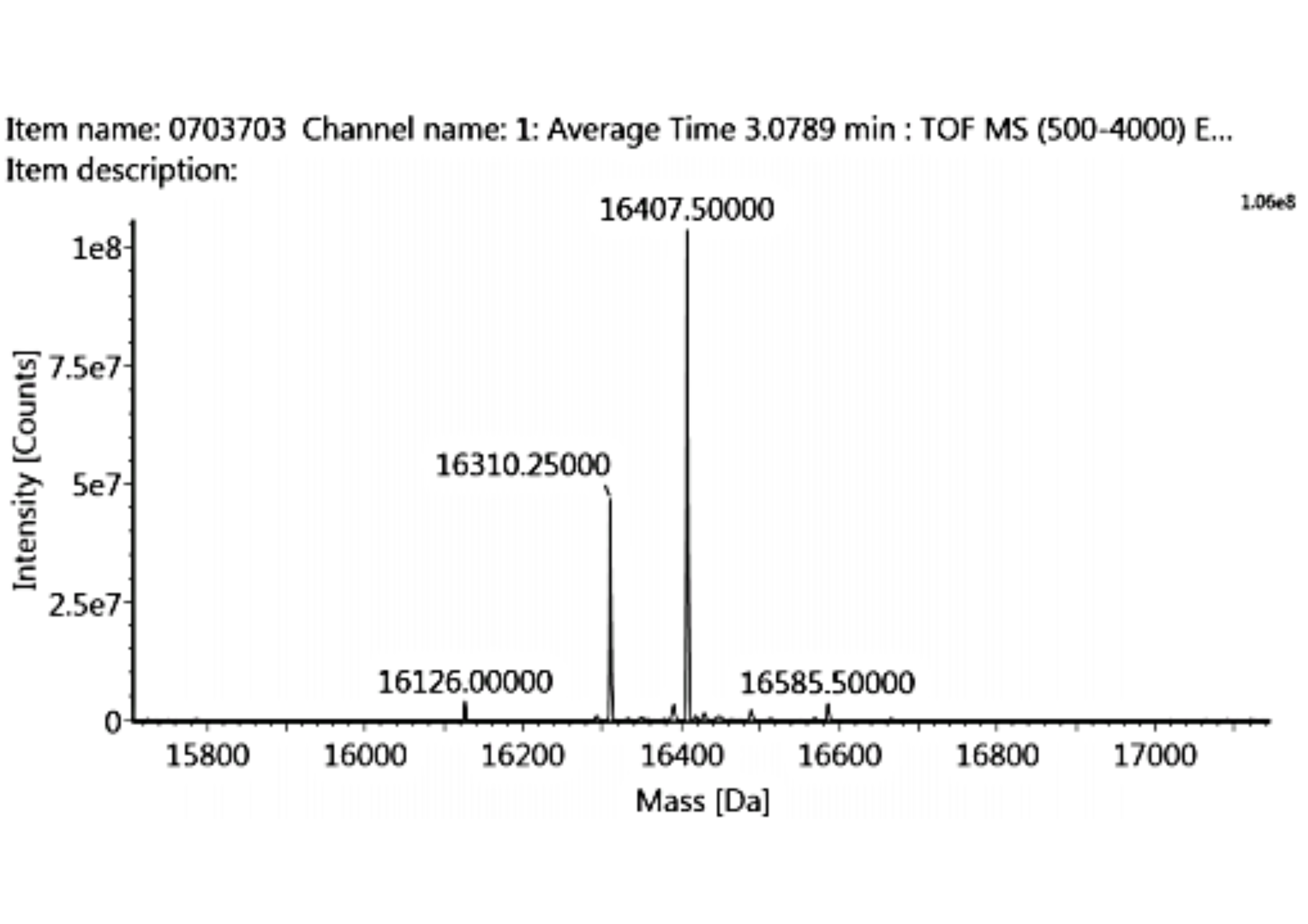

Supplement: Supplementary file 12 [file DataSheet5.ZIP › Figure 5/Fig 5F.png]

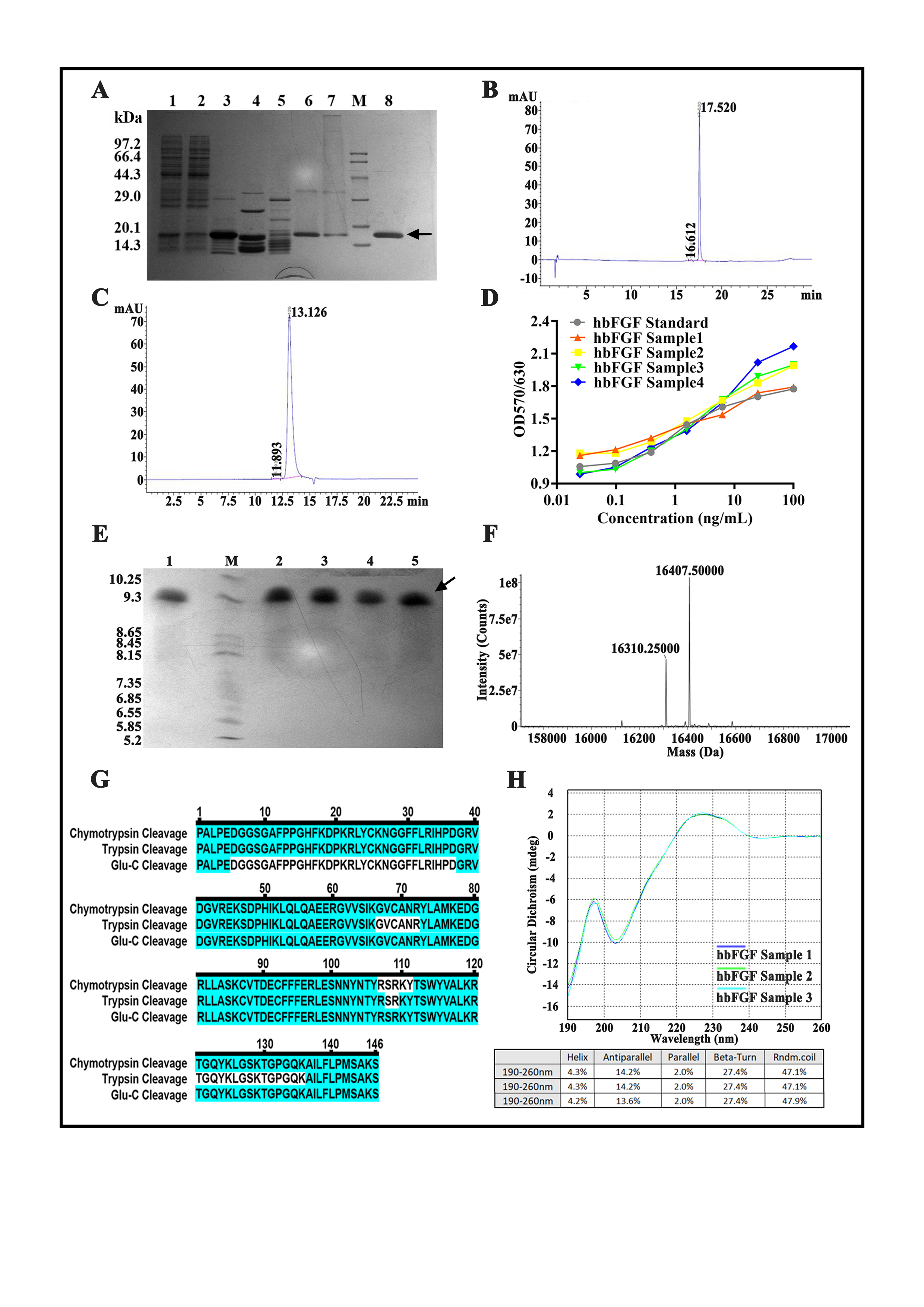

Supplement: Supplementary file 12 [file DataSheet5.ZIP › Figure 5/Figure 5.tif]

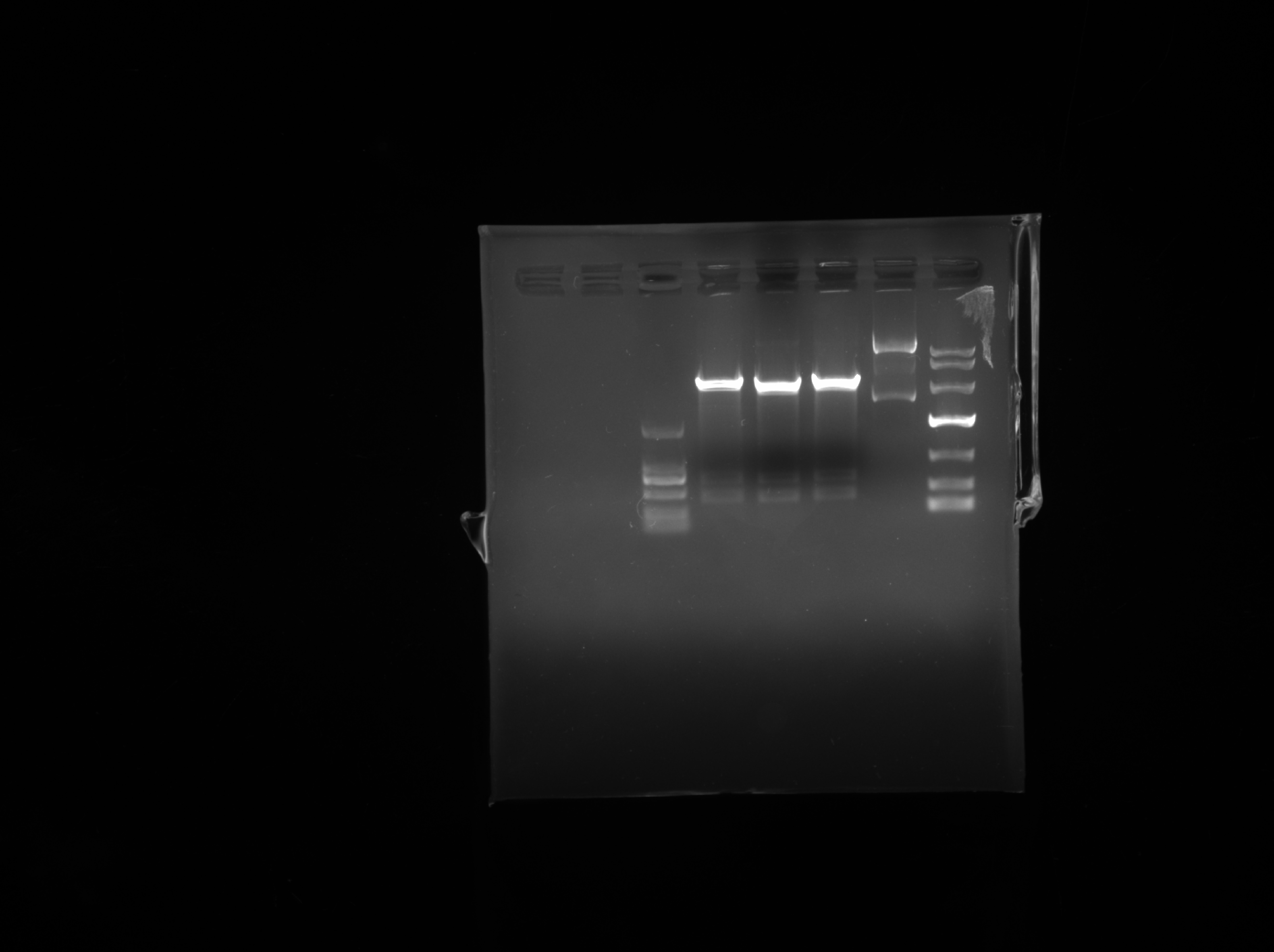

Supplement: Supplementary file 13 [file DataSheet7.ZIP › Supplementary Figure 1/Fig S1A .jpg]

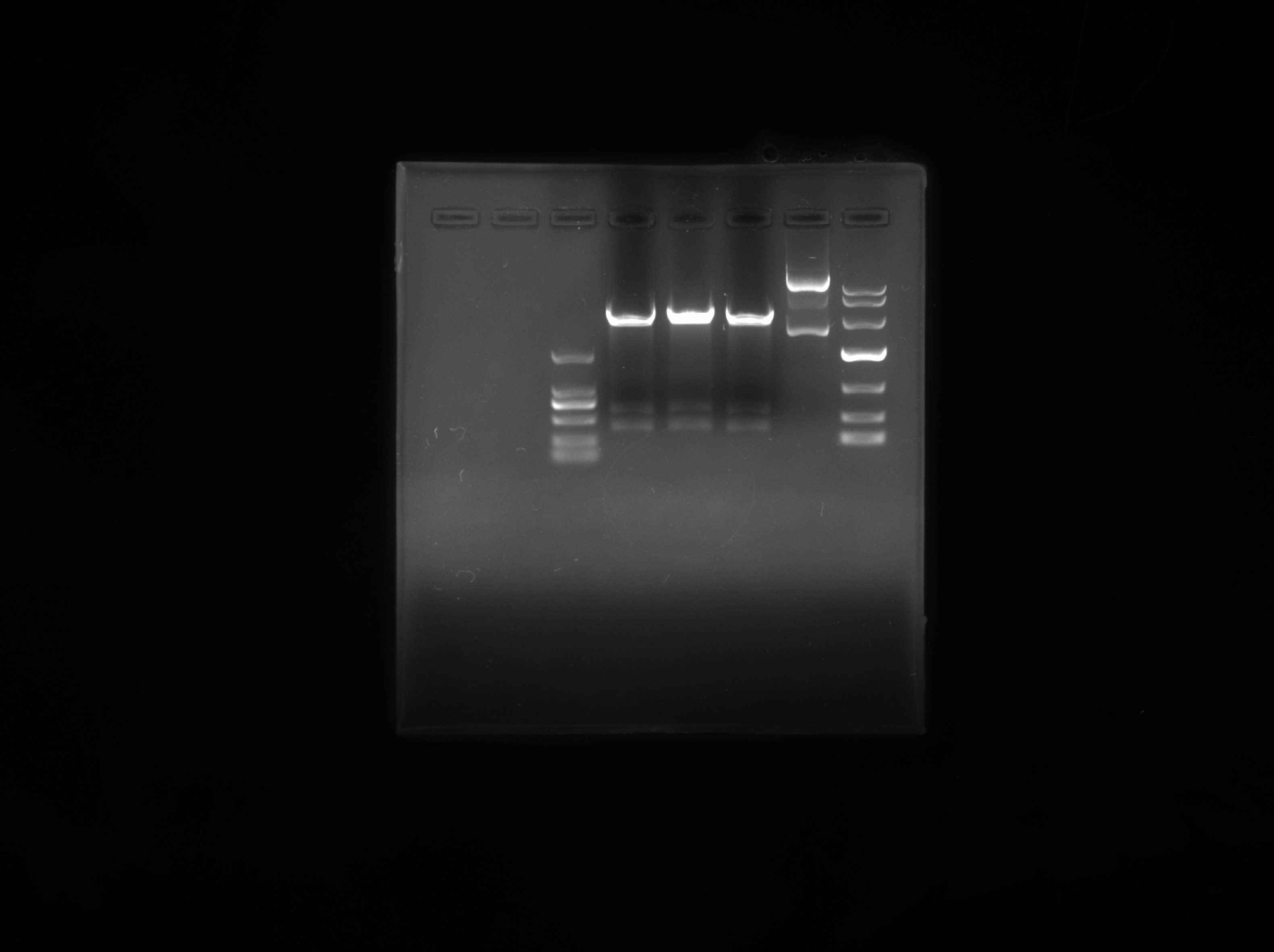

Supplement: Supplementary file 13 [file DataSheet7.ZIP › Supplementary Figure 1/Fig S1B.jpg]

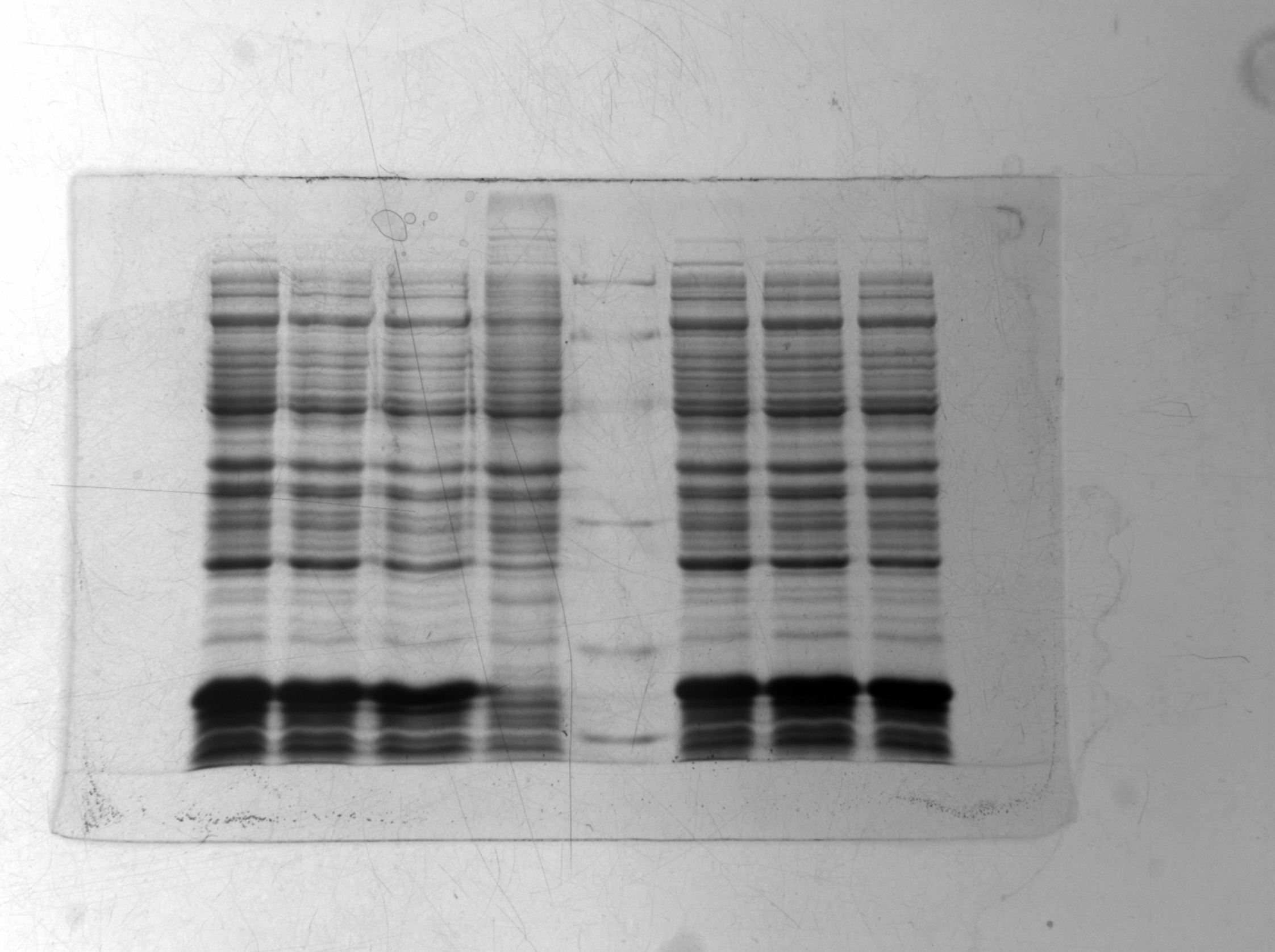

Supplement: Supplementary file 13 [file DataSheet7.ZIP › Supplementary Figure 1/Fig S1C.jpg]

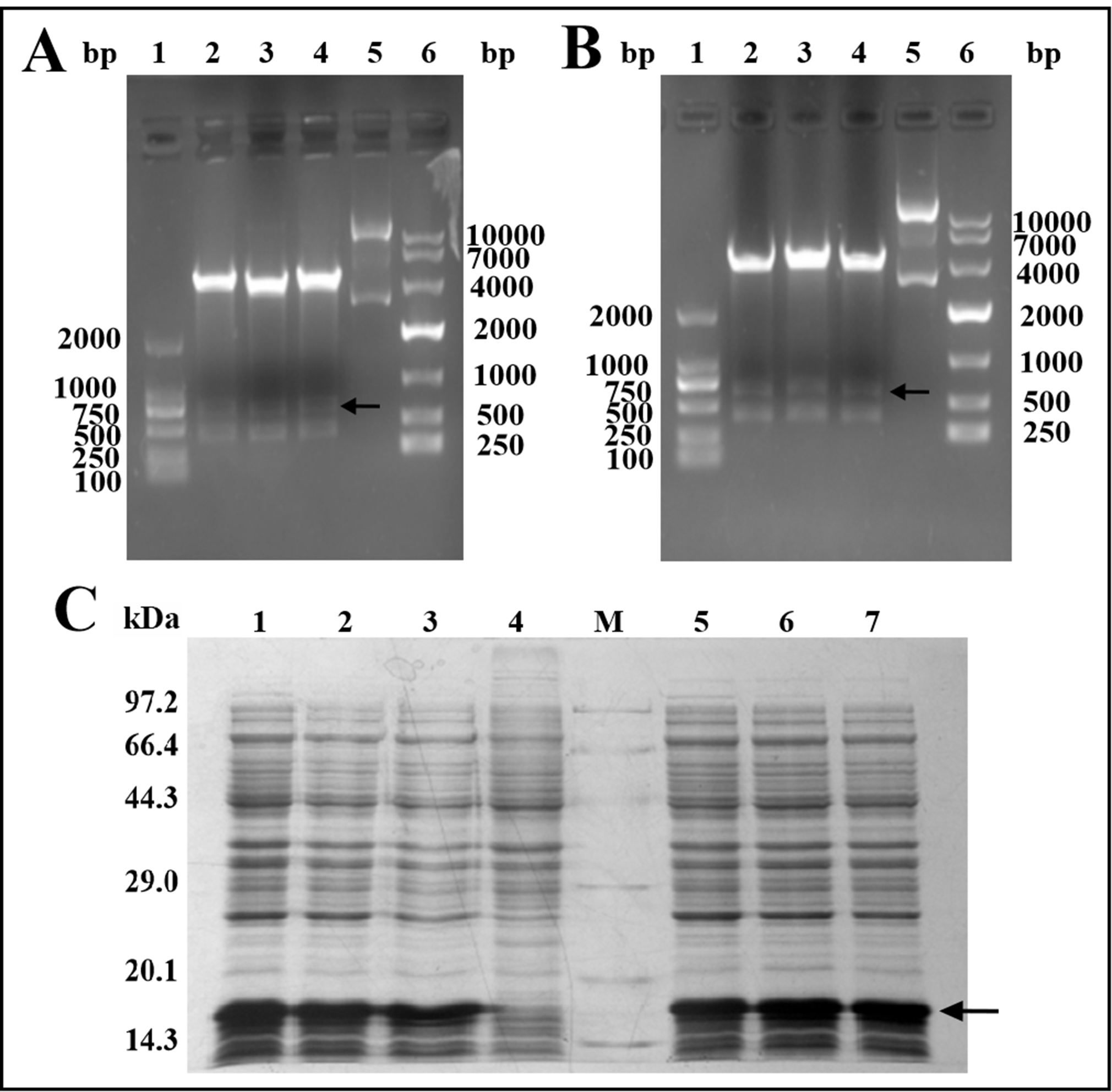

Supplement: Supplementary file 13 [file DataSheet7.ZIP › Supplementary Figure 1/Figure S1.tif]
