# Supplementary material for: Scaling up production of recombinant human basic fibroblast growth factor in an Escherichia coli BL21(DE3) plysS strain and evaluation of its pro-wound healing efficacy
Source: Front Pharmacol. 2024 Feb 5;14:1279516. doi: 10.3389/fphar.2023.1279516 (PMC10875678; doi:10.3389/fphar.2023.1279516)
Supplement: Supplementary file 5 [file Table1.DOCX]

**Supplementary Material**

**1 Methods**

**1.1 Stability test of the hbFGF engineered strain**

**(1) Genetic stability test:** The engineered strains were inoculated into 30 ml of LB liquid medium at a ratio of 1:100 (v/v) and cultured for 12 h at 37 ℃ and 150 rpm as the first generation. Subsequently, they were transferred respectively in a ratio of 1:100 (v/v) to 30 ml and 300 ml of LB liquid medium for subsequent generations and then incubated at 37 ℃ and 150 rpm. The cells were passed every 12 hours until the 30th generation. During this process, at the 8th, 18th, and 28th generations, the bacterial solution was diluted 10^5^ times and 100 μl of the bacterial solution was taken for coating on a non-resistant LB solid medium. After incubation at 37 ℃ for 12 h, single colonies were randomly picked and inoculated into both non-resistant LB solid medium and kanamycin-resistant LB solid medium (containing 100 µg/mL kanamycin sulfate). A total of 100 single colonies were picked and incubated at 37 ℃ for 12 h before being enumerated, from which the rate of plasmid loss was calculated.

**(2) Structural stability test:** The plasmids were extracted from the cells of passages 10, 20, and 30 and digested with *Nde* I and *Bam*H I. Then, these digested plasmids were detected by 1% agarose gel electrophoresis for comparative verification.

**(3) Protein expression stability test:** After passages 9, 19, and 29 of culture, the bacteria were inoculated into new 250 mL triangular flasks containing 30 mL of LB medium at a ratio of 1:100 (v/v) and induced for hbFGF expression using IPTG. Subsequently, the expression level of hbFGF was assessed through comparative verification employing a 12% SDS-PAGE analysis.

**1.2 SDS-PAGE and Western blotting**

The hbFGF samples were mixed with 4×loading buffer at a ratio of 3:1, and 10 µL of these prepared samples were loaded on the well of a 12% SDS-PAGE along with a low molecular protein marker (Beyotime). After running the gel for 20 min at 80 V and then for 90 min at 120 V, the gel was removed and placed in a fixative solution (containing 1% trichloroacetic acid and 40% methanol) for 60 min. It was then stained with coomassie brilliant blue R250 for 90 min. Subsequently, after 3 times (30 min each time) of destaining using a solution consisting of 20% methanol and 10% glacial acetic acid, the gel was photographed using the Gel Imager (Chemi Doc XRS+, Bio-RAD), and subsequent purity analysis was conducted using Image Lab Softmax 6.0.

Western blotting was performed as follows: Proteins on the gel were transferred onto a nitrocellulose membrane (Bio-RAD) after running at 300 mA for 90 min. The membrane was then blocked with a TBST blocking solution (containing 5% BSA and 0.1% Tween 20) for 2 h at room temperature. Subsequently, it was placed in a polyclonal rabbit anti-human bFGF antibody solution diluted with TBST solution (containing 0.1% Tween 20) at 1:1000 and incubated overnight at 4 ℃. After 3 times washed with TBST for 8 min each time, this membrane was incubated at room temperature in a goat anti-rabbit lgG/HRP secondary antibody solution with a dilution of 1:5000 for 1 h. After another three washes with TBST, the bands were imaged using the BeyoECL Plus kit (Beyotime) and analyzed by Image Lab Softmax 6.0.

**1.3 Reverse-phase high-performance liquid chromatography (RP-HPLC)**

The detection of RP-HPLC purity of purified hbFGF was performed on the Agilent 1260 HPLC system as described below:

| **Chromatogram condition** | | **Elution procedure** | | |
| --- | --- | --- | --- | --- |
| Column | Aglient C18 column (4.6 × 150 nm) | **Time (min)** | **A%** | **B%** |
| Column temperature | 25 ± 1 ℃ | 2 | 90 | 10 |
| Mobile phase A | 0.1% TFA-aqueous solution | 14 | 65 | 35 |
| Mobile phase B | 0.1% TFA-acetonitrile solution | 18 | 59 | 41 |
| Samples concentration | 1 mg/mL | 23 | 55 | 45 |
| loading volume | 20 μL | 25 | 90 | 10 |
| detection wavelength | 280 nm | 30 | 90 | 10 |
| Flow rate | 1 mL/min |  |  |  |

**1.4 High-performance size exclusion chromatography (SEC-HPLC)**

The detection of SEC-HPLC purity of purified hbFGF was performed on the Agilent 1260 HPLC system as described below:

| **Chromatogram condition** | | **Elution procedure** | |
| --- | --- | --- | --- |
| Column | TSK GEL G2000SWxl (7.8 × 30 cm)  TSK GEL GUARDSWxl (6.0 × 4 cm) | **Time (min)** | **A%** |
| Column temperature | 28 ± 1 ℃ | 0 | 100 |
| Mobile phase A | 100 mM citrate buffer, 0.2 M Na_2_SO_4_, pH 6.1 | 25 | 100 |
| Samples concentration | 2 mg/mL |  |  |
| loading volume | 20 μL |  |  |
| detection wavelength | 280 nm |  |  |
| Flow rate | 0.8 mL/min |  |  |

**1.5 Isoelectric focusing electrophoresis**

The tested hbFGF protein sample was diluted to a concentration of 2 mg/mL and then transferred into an ultrafiltration centrifuge tube, followed by centrifugation at 5000 g and 4 ℃ for 30 min. Subsequently, 5 μL of the prepared sample and pI standard was added to the immobilized pH gradient gel strips (pH, 5-10.5). The electrophoresis was performed using a DYY-6C electrophoresis apparatus (Liuyi Instrumental Co., China) as follows: 200 V for 30 min, 400 V for 30 min, 600 V for 1 h, 800 V for 15 min. Afterward, the gel was removed from the apparatus and placed in a fixative solution (20% trichloroacetic acid) for 30 min. Then, it was stained in a staining solution (0.07% Coomassie brilliant blue G250, 8.3% ammonium sulfate, 1.3% phosphoric acid, 33.3% methanol) for 1 h before being decolorized by purified water until achieving a colorless background. Finally, the gel was scanned using the Gel Imager (Chemi Doc XRS+, Bio-RAD) and subsequent pI calculation was conducted using Image Lab Softmax 6.0.

**1.6 Bicinchoninic acid (BCA) method**

Precisely take 0.0 mL, 0.1 mL, 0.2 mL, 0.3 mL, 0.4 mL, and 0.5 mL of BSA standard solution (0.8 mg/mL) along with a hbFGF sample solution of 0.25 mL and transfer them into stoppered test tubes followed by addition of purified water to achieve a total volume of each tube as 0.5 mL. Subsequently, add 10 mL of Copper-BCA reagent (consisting of 1 g Bicinchoninic acid disodium salt, 2 g anhydrous sodium carbonate, 16 g sodium tartrate, 0.4 g NaOH, 0.95 g NaHCO_3_, and 0.08 gGuSO_4_ in a total volume of 102 mL) to all the test tubes and mix thoroughly immediately after addition. Incubate these test tubes in a water bath at a temperature of 37 ℃ for about 30 min and subsequently measure their absorbance values at a wavelength of 562 nm once they return to room temperature. Subsequently, calculate the linear regression equation between the concentration of reference solutions and their corresponding absorbance values. Finally, substitute the obtained absorbance value from the test solution into a linear regression equation and multiply it by the dilution factor to obtain the protein concentration of purified hbFGF.

**2 Supplementary Figures and Tables**

**2.1 Supplementary Figures**


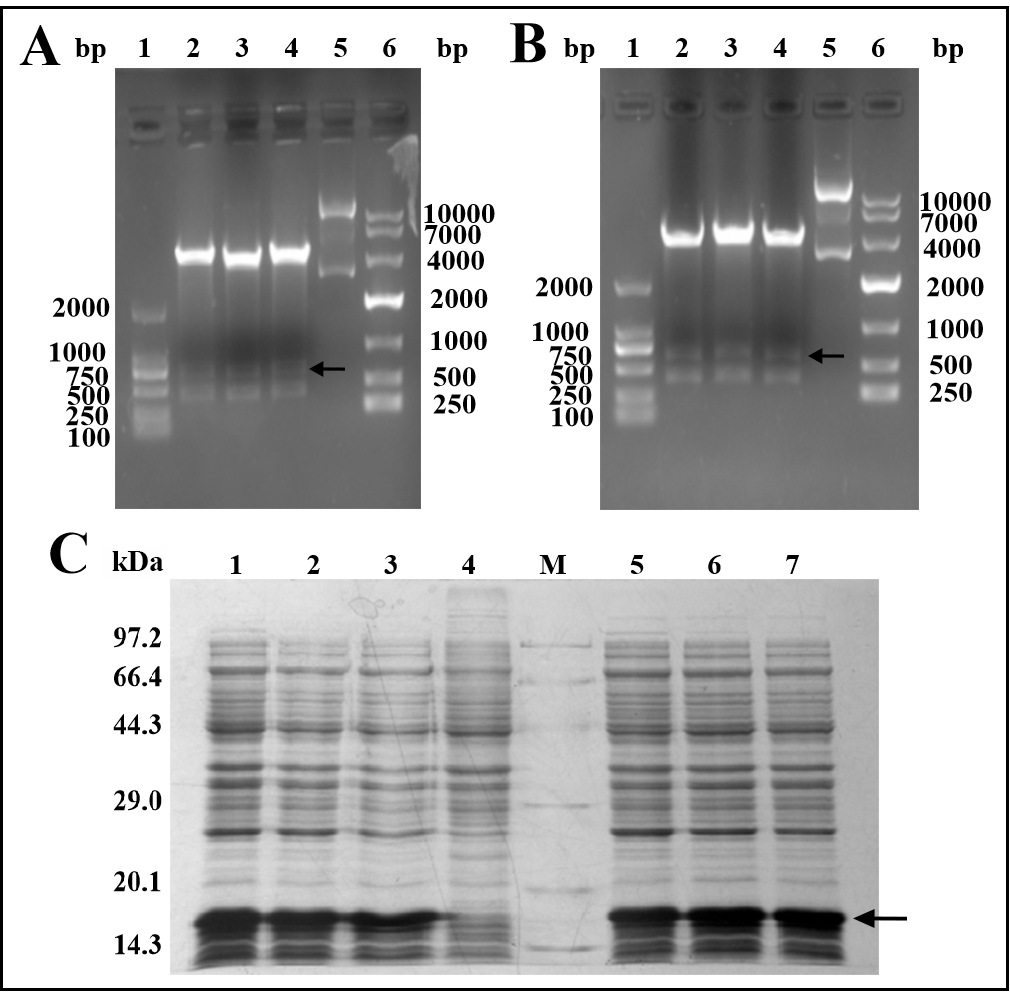


**Figure S1. Genetic stability of the rhbFGF-engineered *E. coli* strain.**

Restriction map of the mpET3c-hbFGF recombinant plasmid in **(A)** 30 mL of LB medium and **(B)** 300 mL of LB medium at different passage periods. Lanes 1 and 6, DNA molecular weight marker. Lanes 2-4, the digested recombinant plasmid at 10, 20, and 30 passages. Lane 5, pre-digestion. **(C)** The expression level of hbFGF at different passage periods. Lanes 1-3, the expression level of hbFGF in 300 mL of LB medium at 10, 20, and 30 passages. Lane 4, pre-induction. Lane M, molecular weight marker. Lanes 5-7, the expression level of rhbFGF in 30 mL of LB medium at 10, 20, and 30 passages. Black arrows indicate hbFGF.


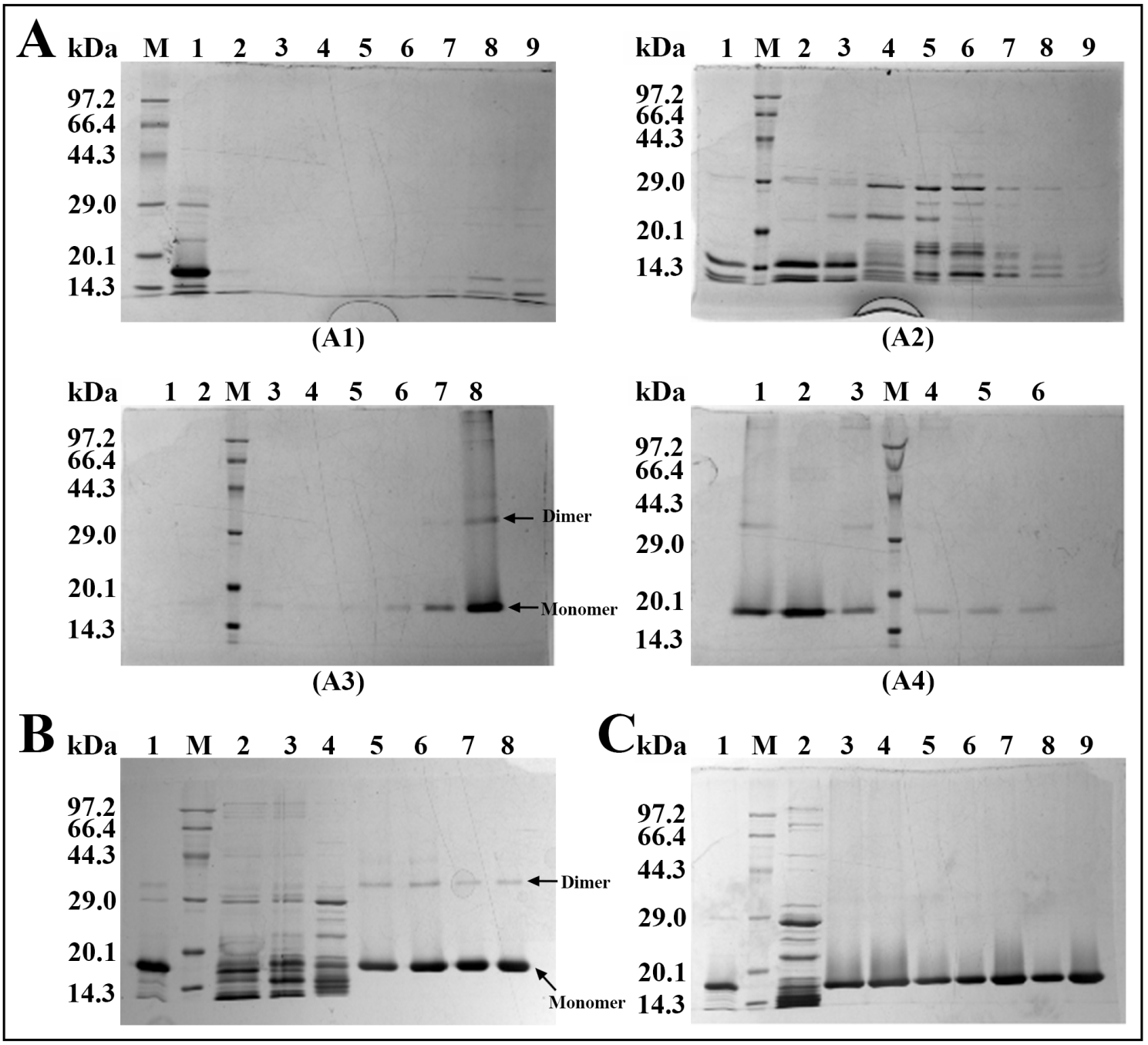


**Figure S2. SDS-PAGE analysis of the elution gradient screening on heparin affinity chromatography.**

**(A)** Non-reduced SDS-PAGE analysis of the different elution fractions on a 1 mL HiTrap Heparin HP column (GE Healthcare). Lane M, molecular weight marker. **(a1)** Lane 1, CM-Sepharose-eluted samples. Lane 2, flow-through samples. Lanes 4-9, samples eluted with a gradient of 0.3–0.6 M NaCl at a flow rate of 1 mL/min. **(a2)** Lane 1, 0.65 M NaCl-eluted samples. Lanes 2-9, samples eluted with a gradient of 0.7–1.1 M NaCl at a flow rate of 1 mL/min. **(a3)** Lane 1, 1.15 M NaCl-eluted samples. Lane 2, 1.2 M NaCl-eluted samples. Lanes 3-8, samples eluted with a gradient of 1.25–1.75 M NaCl at a flow rate of 1 mL/min. **(a4)** Lane 1, 1.8 M NaCl-eluted samples. Lane 2, 1.8 M NaCl-eluted samples (reduced). Lane 3, 1.85 M NaCl-eluted samples. Lanes 4-6, samples eluted with a gradient of 1.9–2.0 M NaCl at a flow rate of 1 mL/min. **(B)** Non-reduced SDS-PAGE analysis of heparin affinity chromatography with a purification buffer containing 5% (w/v) glycerin. Lanes 1 and 6, DNA molecular weight marker. Lane 1, CM-Sepharose-eluted samples. Lanes 2-4, different segments of the 0.72 M NaCl-eluted peak. Lanes 5-8, 2.0 M NaCl-eluted samples. Lane 9, purified hbFGF desalted by a Sephadex G25 column. Lane M, molecular weight marker. **(C)** Non-reduced SDS-PAGE analysis of heparin affinity chromatography with purification buffer containing 10% (w/v) glycerin. Lane 1, CM-Sepharose-eluted samples. Lanes 2-4, different segments of the 0.72 M NaCl-eluted peak. Lanes 5-7, different segments of the 2.0 M NaCl-eluted peak. Lanes 8-9, purified hbFGF desalted by a Sephadex G25 column. Lane M, molecular weight marker. Black arrows indicate hbFGF


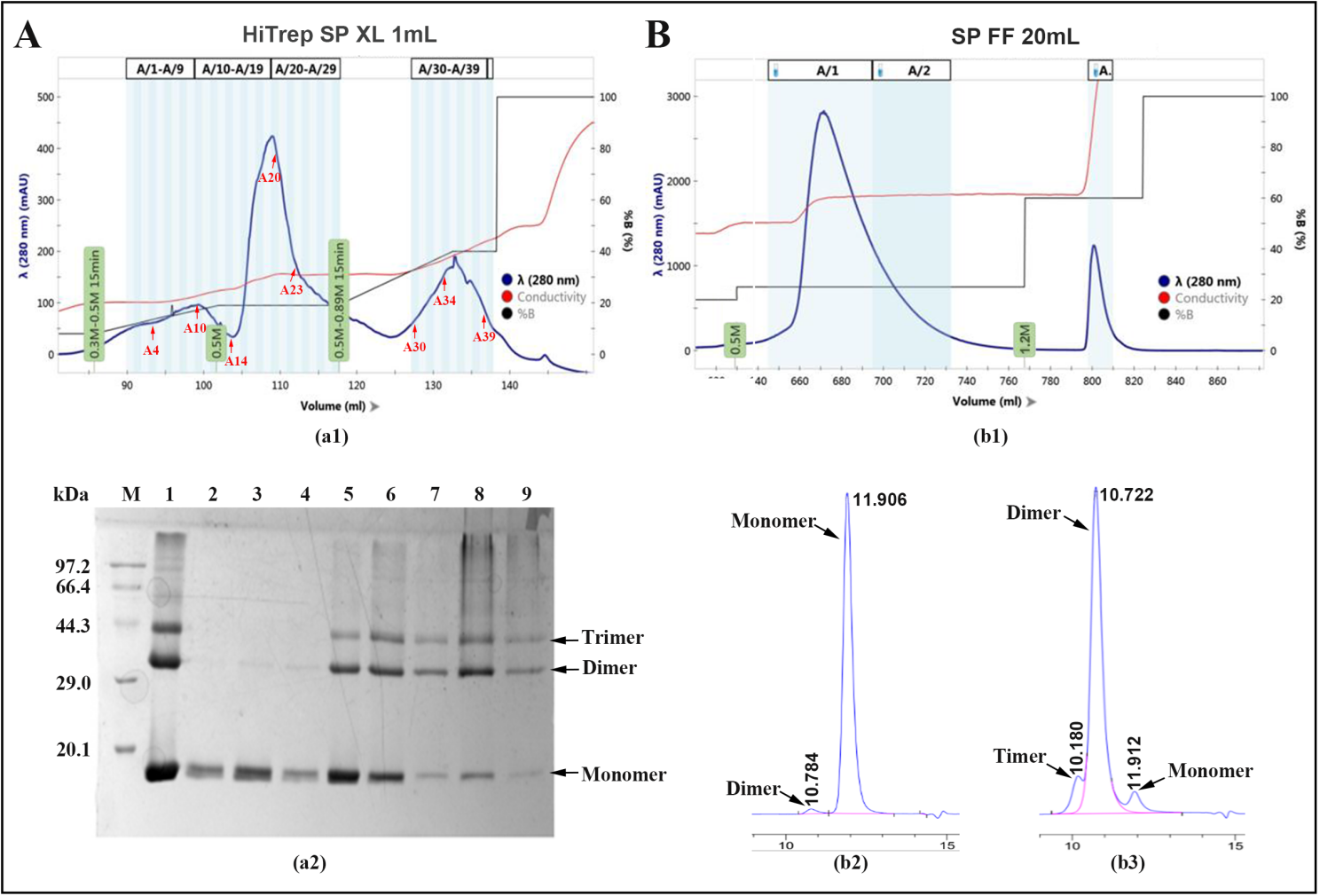


**Figure S3. Exploration of the purification of hbFGF protein by SP-Sepharose chromatography.**

**(A)** The **(a1)** purification profile on a 1-mL HiTrap SP-XL column (GE Healthcare), and **(a2)** the SDS-PAGE analysis of eluted fractions. Lane M, molecular weight marker. Lane 1, heparin affinity-eluted samples (−20 ± 5°C for 13 months). Lanes 2 (No. A4) and 3 (No. A10), fractions eluted with a 15-mL gradient with 0.3–0.5 M NaCl. Lanes 4 (No. A14), 5 (No. A20), and 6 (No. A23), 0.5 M NaCl-eluted fractions. Lanes 7 (No. A30), 8 (No. A34), and 9 (No. A39), fractions eluted with a 15-mL gradient of 0.5–0.8 M NaCl. **(B)** **(b1)** The purification profile of hbFGF protein on a 20-mL SP Sepharose FF column, and the SEC-HPLC analysis of **(b2)** 0.5 M NaCl-eluted samples and **(b3)** 1.2 M NaCl-eluted samples. Black arrows indicate hbFGF.


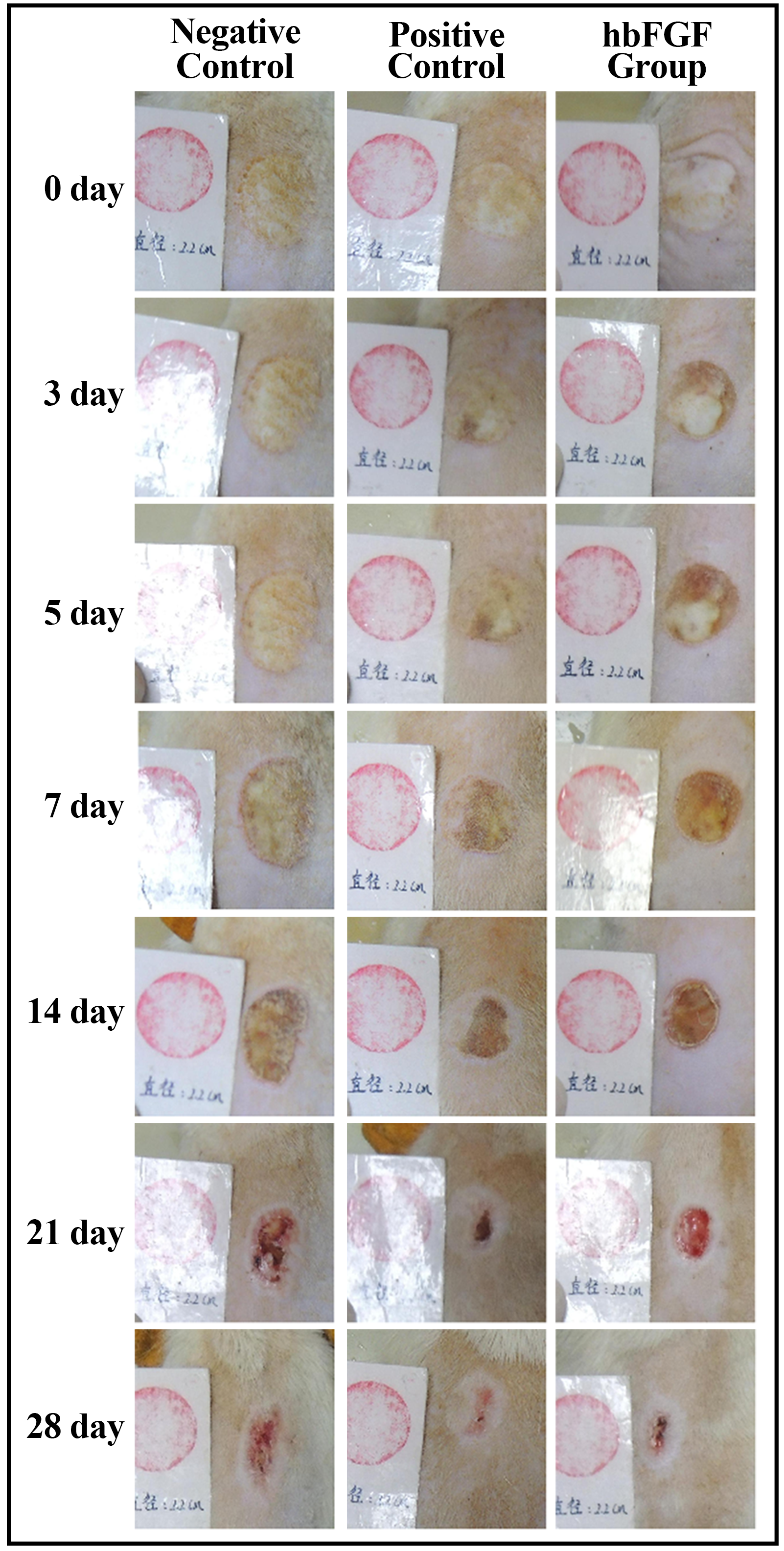


**Figure S4. Photos of the skin wound of STZ-induced SD rats with deep second-degree scald wounds.**


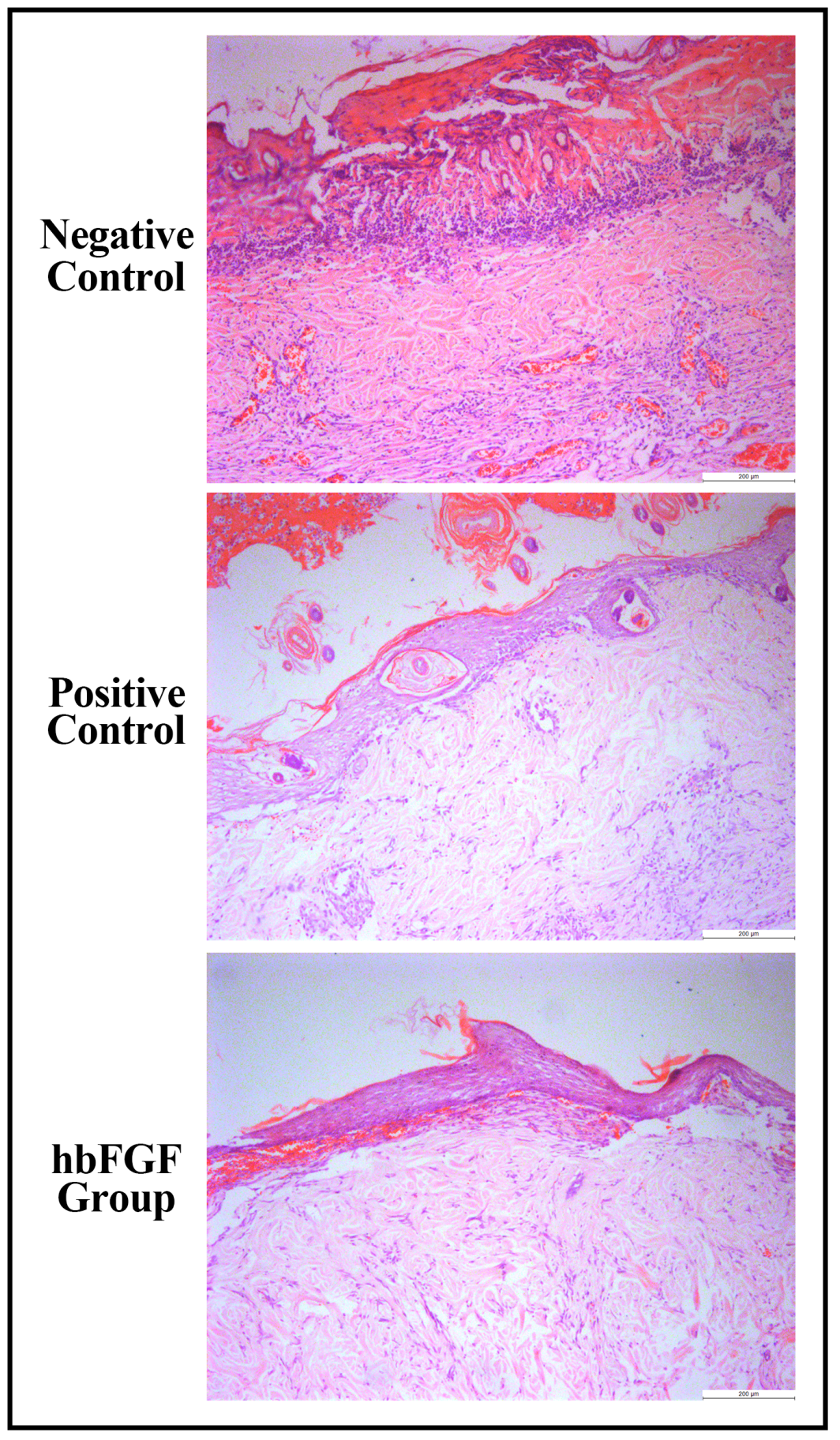


**Figure S5. HE staining of the skin wound tissue in STZ-induced SD rats with deep second-degree scald wounds (scale bar, 200 μm).**

**2.2 Supplementary Tables**

**Table S1.** The synthesis and amplification process of the hbFGF target gene

|  | **Round 1: Overlap PCR** | **Round 2: Standard PCR** |
| --- | --- | --- |
| **Purpose** | Synthesis of hbFGF target gene | Amplification of hbFGF target gene |
| **50 μL-reaction system** | 50 pmoL/μL primer PI-PVIII 0.5 μL  PCR polymerase (PV2) 0.5 μL  5 × PV2 buffer 10 μL  10 mM dNTP 1 μL  ddH_2_O 34.5 μL | Product of round 1 PCR 0.3 μL  50 pmoL/μL primer PI 0.5 μL  50 pmoL/μL primer PVIII 0.5 μL  PCR polymerase (PV2) 0.5 μL  5 × PV2 buffer 10 μL  10 mM dNTP 1 μL  ddH_2_O 37.2 μL |
| **PCR protocol** | 95°C for 3 min  95°C for 25 s  60°C for 20 s 25 cycles  72°C for 40 s  72°C for 1 min | 95°C for 3 min  95°C for 25 s  60°C for 20 s 25 cycles  72°C for 40 s  72°C for 1 min |
|  | 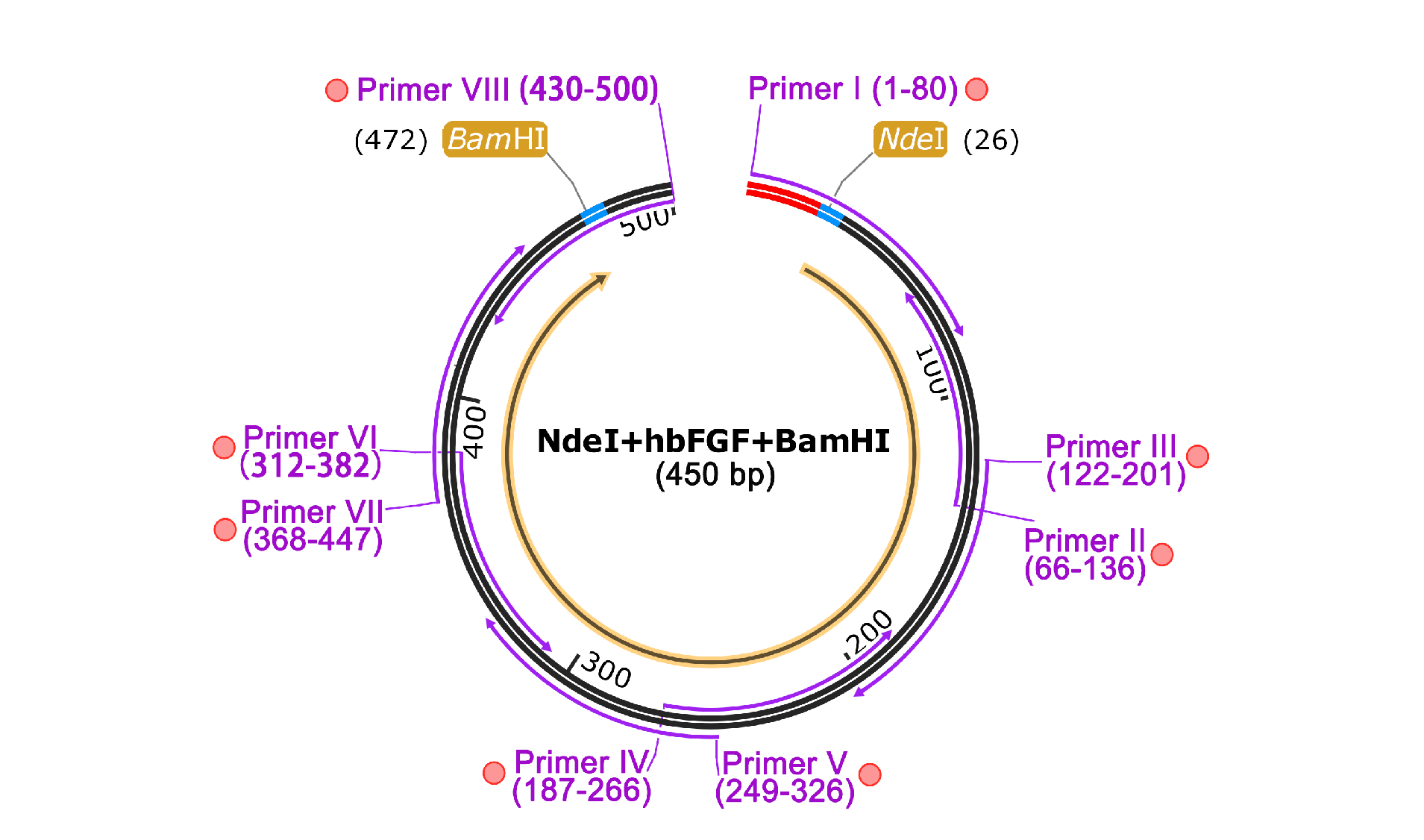 | 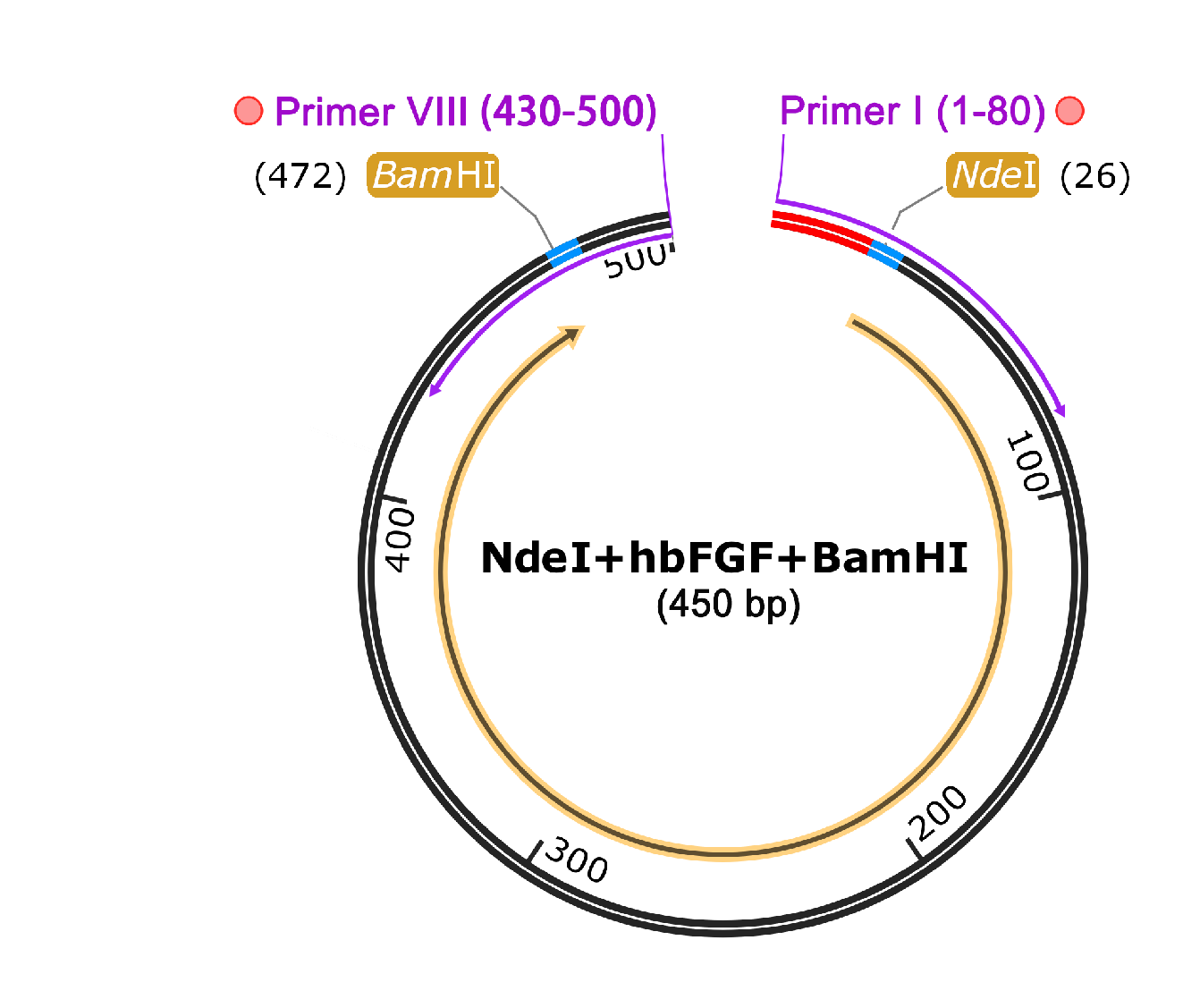 |
| **Product recovery** | The PCR products were detected by 1.0% agarose gel electrophoresis, and the target DNA fragments were recovered by a gel extraction kit. | |

**Table S2.** The PCR primers

| **Upstream Primers** | PII  (71 bp) | 5’-GGTGGATGCGCAGGAAGAAGCCCCCGTTTTTGCAGTACAGCCGCTTGGGGTCCTTGAAGTGGCCGGGCGGG-3’ |
| --- | --- | --- |
|  | PIV  (80 bp) | 5’-TCTTCCTTCATAGCCAGGTAACGGTTAGCACACACTCCTTTGATAGACACAACTCCTCTCTCTTCTGCTTGAAGTTGTAG-3’ |
|  | PVI  (71 bp) | 5’-GTGCCACATACCAACTGGTGTATTTCCTTGACCGGTAAGTATTGTAGTTATTAGATTCCAATCGTTCAAAA-3’ |
|  | PVIII (71 bp) | 5’-CCTTTCGGGCTTTGTTAGCAGCC***GGATCC***TTAGCTCTTAGCAGACATTGGAAGAAAAAGTATAGCTTTCTG-3’  ***Bam*HI** |
| **Downstream Primers** | PI  (80 bp) | 5’-GTTTAACTTTAAGAAGGAGATATA***CATATG***CCAGCTTTGCCCGAGGATGGT  ***Nde*I**  GGTAGCGGCGCCTTCCCGCCCGGCCACTT-3’ |
|  | PIII  (80 bp) | 5’-TCCTGCGCATCCACCCCGACGGCCGAGTTGACGGGGTCCGGGAGAAGAGCGACCCTCATATAAAGCTACAACTTCAAGCA-3’ |
|  | PV  (78 bp) | 5’-CCTGGCTATGAAGGAAGATGGAAGATTACTGGCTTCTAAATGTGTTACGGATGAGTGTTTCTTTTTTGAACGATTGGA-3’ |
|  | PVII  (80 bp) | 5’-GTTGGTATGTGGCACTGAAACGAACTGGGCAGTATAAGCTTGGGTCTAAAACAGGACCTGGGCAGAAAGCTATACTTTTT-3’ |

**Table S3.** Design and results of the response surface experiment

| **Run** | **Factor** | | | | | **Response** | |
| --- | --- | --- | --- | --- | --- | --- | --- |
|  | **Temperature (°C)** | **pH** | **IPTG (mmol/L)** | **NH_4_Cl (g/L)** | **Induced time (h)** | **OD_600_** | **Expression level (%)** |
| 1 | 30 | 7 | 1 | 4 | 5 | 2.1 | 16.4 |
| 2 | 30 | 6 | 1 | 4 | 4 | 1.59 | 17.4 |
| 3 | 30 | 7 | 1 | 4 | 3 | 1.955 | 16.4 |
| 4 | 30 | 7 | 1 | 0 | 4 | 1.85 | 16.8 |
| 5 | 30 | 7 | 1.8 | 4 | 4 | 2.05 | 15.5 |
| 6 | 30 | 7 | 0.2 | 4 | 4 | 2.61 | 17.2 |
| 7 | 30 | 8 | 1 | 4 | 4 | 1.7 | 15.3 |
| 8 | 30 | 7 | 1 | 8 | 4 | 1.81 | 15.8 |
| 9 | 34 | 8 | 1 | 4 | 3 | 1.915 | 18.8 |
| 10 | 34 | 6 | 1.8 | 4 | 4 | 1.96 | 26.4 |
| 11 | 34 | 6 | 1 | 0 | 4 | 1.74 | 28.4 |
| 12 | 34 | 7 | 1 | 4 | 4 | 2.495 | 22.4 |
| 13 | 34 | 8 | 1 | 8 | 4 | 1.715 | 19 |
| 14 | 34 | 6 | 1 | 8 | 4 | 1.735 | 26.8 |
| 15 | 34 | 8 | 0.2 | 4 | 4 | 2.595 | 19.7 |
| 16 | 34 | 7 | 1 | 4 | 4 | 2.455 | 23 |
| 17 | 34 | 7 | 1 | 0 | 3 | 1.805 | 22.1 |
| 18 | 34 | 7 | 0.2 | 0 | 4 | 2.745 | 25.7 |
| 19 | 34 | 8 | 1.8 | 4 | 4 | 1.99 | 17.9 |
| 20 | 34 | 7 | 0.2 | 4 | 3 | 2.885 | 24.8 |
| 21 | 34 | 7 | 1.8 | 8 | 4 | 2.23 | 20.3 |
| 22 | 34 | 7 | 0.2 | 8 | 4 | 2.65 | 25 |
| 23 | 34 | 6 | 0.2 | 4 | 4 | 2.595 | 29.1 |
| 24 | 34 | 7 | 0.2 | 4 | 5 | 2.945 | 25.8 |
| 25 | 34 | 7 | 1.8 | 4 | 5 | 2.53 | 20.4 |
| 26 | 34 | 6 | 1 | 4 | 3 | 1.855 | 26.6 |
| 27 | 34 | 8 | 1 | 4 | 5 | 2.08 | 19.5 |
| 28 | 34 | 7 | 1.8 | 0 | 4 | 2.24 | 20.3 |
| 29 | 34 | 7 | 1 | 0 | 5 | 2.115 | 24.8 |
| 30 | 34 | 8 | 1 | 0 | 4 | 1.725 | 19.3 |
| 31 | 34 | 6 | 1 | 4 | 5 | 2.08 | 28.7 |
| 32 | 34 | 7 | 1 | 4 | 4 | 2.37 | 23 |
| 33 | 34 | 7 | 1 | 4 | 4 | 2.365 | 23.6 |
| 34 | 34 | 7 | 1 | 4 | 4 | 2.365 | 24 |
| 35 | 34 | 7 | 1.8 | 4 | 3 | 2.22 | 20 |
| 36 | 34 | 7 | 1 | 8 | 5 | 2.115 | 24.7 |
| 37 | 34 | 7 | 1 | 4 | 4 | 2.355 | 24.3 |
| 38 | 34 | 7 | 1 | 8 | 3 | 1.775 | 21.1 |
| 39 | 38 | 7 | 1.8 | 4 | 4 | 2.575 | 31.5 |
| 40 | 38 | 7 | 1 | 4 | 5 | 2.58 | 32.8 |
| 41 | 38 | 8 | 1 | 4 | 4 | 2.19 | 30.3 |
| 42 | 38 | 7 | 1 | 0 | 4 | 2.3 | 33.3 |
| 43 | 38 | 7 | 0.2 | 4 | 4 | 3 | 34.2 |
| 44 | 38 | 7 | 1 | 4 | 3 | 2.34 | 31.7 |
| 45 | 38 | 7 | 1 | 8 | 4 | 2.25 | 31.7 |
| 46 | 38 | 6 | 1 | 4 | 4 | 2.165 | 36.5 |

**Table S4.** ANOVA of the response surface quadratic model of the response expression level value

| **Factor** | **Sum of Squares** | **df** | **Mean Square** | **F value** | ***P* value** |
| --- | --- | --- | --- | --- | --- |
| Model | 1393.70 | 20 | 69.68 | 54.45 | < 0.0001^****^ |
| A-Temperature | 1075.84 | 1 | 1075.84 | 840.66 | < 0.0001^****^ |
| B-pH | 225.75 | 1 | 225.75 | 176.40 | < 0.0001^****^ |
| C-IPTG | 53.29 | 1 | 53.29 | 41.64 | < 0.0001^****^ |
| D-NH_4_Cl | 2.48 | 1 | 2.48 | 1.94 | 0.1761 |
| E-Induced time | 8.41 | 1 | 8.41 | 6.57 | 0.0168^*^ |
| AB | 4.20 | 1 | 4.20 | 3.28 | 0.0820 |
| AC | 0.25 | 1 | 0.25 | 0.20 | 0.6623 |
| AD | 0.090 | 1 | 0.090 | 0.070 | 0.7930 |
| AE | 0.30 | 1 | 0.30 | 0.24 | 0.6311 |
| BC | 0.20 | 1 | 0.20 | 0.16 | 0.6942 |
| BD | 0.42 | 1 | 0.42 | 0.33 | 0.5707 |
| BE | 0.49 | 1 | 0.49 | 0.38 | 0.5417 |
| CD | 0.12 | 1 | 0.12 | 0.096 | 0.7596 |
| CE | 0.090 | 1 | 0.090 | 0.070 | 0.7930 |
| DE | 0.20 | 1 | 0.20 | 0.16 | 0.6942 |
| A^2^ | 14.56 | 1 | 14.56 | 11.38 | 0.0024^**^ |
| B^2^ | 0.35 | 1 | 0.35 | 0.27 | 0.6061 |
| C^2^ | 0.74 | 1 | 0.74 | 0.58 | 0.4534 |
| D^2^ | 0.29 | 1 | 0.29 | 0.23 | 0.6363 |
| E^2^ | 0.44 | 1 | 0.44 | 0.35 | 0.5621 |
| Residual | 31.99 | 25 | 1.28 |  |  |
| *Lack of Fit* | 29.47 | 20 | 1.47 | 2.91 | 0.1195 |
| *Pure Error* | 2.53 | 5 | 0.51 |  |  |
| Cor Total | 1425.69 | 45 |  |  |  |

Expression level (%) = 23.38 + 8.20*A − 3.76*B − 1.83*C − 0.39*D + 0.72*E − 1.03*AB − 0.25*AC − 0.15*AD + 0.27*AE + 0.23*BC − 0.32*BD − 0.35*BE + 0.17*CD − 0.15*CE + 0.22*DE + 1.29*A^2^ + 0.2*B^2^ − 0.29*C^2^ − 0.18*D^2^ − 0.23*E^2^ ($\text{R}^{2}$= 0.9790, ^^= 0.9622)

**Table S5.** ANOVA of the response surface quadratic model of the response value of OD_600_

| **Factor** | **Sum of Squares** | **df** | **Mean Square** | **F value** | ***P* value** |
| --- | --- | --- | --- | --- | --- |
| Model | 5.76 | 20 | 5.76 | 58.31 | < 0.0001^****^ |
| A-Temperature | 0.87 | 1 | 0.87 | 176.40 | < 0.0001^****^ |
| B-pH | 2.256E-003 | 1 | 2.256E-003 | 0.46 | 0.5055 |
| C-IPTG | 1.12 | 1 | 1.12 | 226.26 | < 0.0001^****^ |
| D-NH_4_Cl | 3.600E-003 | 1 | 3.600E-003 | 0.73 | 0.4015 |
| E-Induced time | 0.20 | 1 | 0.20 | 40.74 | < 0.0001^****^ |
| AB | 1.806E-003 | 1 | 1.806E-003 | 0.37 | 0.5509 |
| AC | 4.556E-003 | 1 | 4.556E-003 | 0.92 | 0.3462 |
| AD | 2.500E-005 | 1 | 2.500E-005 | 5.058E-003 | 0.9439 |
| AE | 2.256E-003 | 1 | 2.256E-003 | 0.46 | 0.5055 |
| BC | 2.250E-004 | 1 | 2.250E-004 | 0.046 | 0.8328 |
| BD | 6.250E-006 | 1 | 6.250E-006 | 1.264E-003 | 0.9719 |
| BE | 9.000E-004 | 1 | 9.000E-004 | 0.18 | 0.6732 |
| CD | 1.806E-003 | 1 | 1.806E-003 | 0.37 | 0.5509 |
| CE | 0.016 | 1 | 0.016 | 3.16 | 0.0876 |
| DE | 2.250E-004 | 1 | 2.250E-004 | 0.046 | 0.8328 |
| A^2^ | 0.082 | 1 | 0.082 | 16.68 | 0.0004^***^ |
| B^2^ | 1.28 | 1 | 1.28 | 259.60 | < 0.0001^****^ |
| C^2^ | 0.78 | 1 | 0.78 | 157.92 | < 0.0001^****^ |
| D^2^ | 0.71 | 1 | 0.71 | 144.36 | < 0.0001^****^ |
| E^2^ | 0.053 | 1 | 0.053 | 10.75 | 0.0031^**^ |
| Residual | 31.99 | 25 | 1.28 |  |  |
| *Lack of Fit* | 29.47 | 20 | 1.47 | 2.91 | 0.1195 |
| *Pure Error* | 2.53 | 5 | 0.51 |  |  |
| Cor Total | 1425.69 | 45 |  |  |  |

OD_600_ = 2.4 + 0.23*A + 0.012*B − 0.26*C − 0.015*D + 0.11*E − 0.021*AB + 0.034*AC − 0.0025*AD + 0.024*AE + 0.0075*BC − 0.00125*BD − 0.015*BE + 0.021*CD + 0.062*CE + 0.0075*DE − 0.097*A^2^ − 0.38*B^2^ + 0.30*C^2^ − 0.29*D^2^ − 0.078*E^2^ ($\text{R}^{2}$= 0.9776, ^^= 0.9596)

**Table S6.** Genetic stability of the engineered strain in different volumes of LB medium

| **LB medium** | **Passage number** | **Number of single colonies in plates** | | | **Plasmid loss rate (%)** | **Plasmid stabilization rate (%)** |
| --- | --- | --- | --- | --- | --- | --- |
|  |  | **Non-resistant** | **Resistant^#^** | |  |  |
| 30 mL | 10 | 100 | | 100 | 0 | 100 |
|  | 20 | 100 | | 100 | 0 | 100 |
|  | 30 | 100 | | 100 | 0 | 100 |
| 300 mL | 10 | 100 | | 100 | 0 | 100 |
|  | 20 | 100 | | 100 | 0 | 100 |
|  | 30 | 100 | | 100 | 0 | 100 |

**^#^** The LB solid plate containing 100 µg/mL kanamycin sulfate.

**Table S7.** Plasmid loss rate during 200-L fermentation

| **Batch number** | **Number of single colonies in plates** | | | **Plasmid loss rate (%)** | **Plasmid stabilization rate (%)** |
| --- | --- | --- | --- | --- | --- |
|  | **Non-resistant** | **Resistant** | |  |  |
| 1 | 100 | | 91 | 9 | 91 |
| 3 | 100 | | 92 | 8 | 92 |
| 3 | 100 | | 90 | 10 | 90 |

**^#^** The LB solid plate containing 100 µg/mL kanamycin sulfate.

**Table S8.** The expression level of hbFGF of the engineered strain with storage time (Mean±SD)

|  | **Expression level of hbFGF (%)** | | | | | |
| --- | --- | --- | --- | --- | --- | --- |
|  | **0 month** | **1 month** | **3 months** | **6 months** | **9 months** | **12 months** |
| **Master seed strain^a^** | 26.0 | 25.9 | 26.0 | 25.9 | 26.0 | 26.0 |
| **Working seed strain^a^** | 26.0 | 26.0 | 25.5 | 25.0 | 24.8 | 23.0 |
| **Bacteria pellets^b^** | 28.2 ± 0.2 | 28.2 ± 0.2 | 28.0 ± 0.1 | 26.4 ± 0.3 | 23.2 ± 0.4 | / |

^a^ The bacteria pellets were harvested from 500-L fermentations and stored at −20 ± 5 °C.

^b^ The seed strain was stored at −70 ± 5 °C.

**Table S9.** Summary of the data of the inoculation optimization in a 30-L fermentor

| **Inoculation (%)** | **Volume of fermentation (L)** | **Bacterial wet weight (g)** | **Bacterial density (g/L)** | **Expression level (%)** |
| --- | --- | --- | --- | --- |
| 5 | 15.5 | 650 | 41.9 | 26.1 |
| 10 | 16.0 | 670 | 41.8 | 24.3 |
| 15 | 16.0 | 600 | 37.5 | 21.5 |
