# Supplementary material for: Scaling up production of recombinant human basic fibroblast growth factor in an Escherichia coli BL21(DE3) plysS strain and evaluation of its pro-wound healing efficacy
Source: Front Pharmacol. 2024 Feb 5;14:1279516. doi: 10.3389/fphar.2023.1279516 (PMC10875678; doi:10.3389/fphar.2023.1279516)
Supplement: Supplementary file 10 [file DataSheet12.ZIP › Table/Supplementary Table 1-9.docx]

**Table S1.** The synthesis and amplification process of hbFGF target gene

|  | Round 1: Overlap PCR | Round 2: Standard PCR |
| --- | --- | --- |
| Purpose | Synthesis of hbFGF target gene | Amplification of hbFGF target gene |
| 50 μL reaction system | 50 pmoL/μL primer PI-PVIII 0.5 μL  PCR polymerase (PV2) 0.5 μL  5 × PV2 buffer 10 μL  10 mM dNTP 1 μL  ddH_2_O 34.5 μL | Product of round 1 PCR 0.3 μL  50 pmoL/μL primer PI 0.5 μL  50 pmoL/μL primer PVIII 0.5 μL  PCR polymerase (PV2) 0.5 μL  5 × PV2 buffer 10 μL  10 mM dNTP 1 μL  ddH2O 37.2 μL |
| PCR protocol | 95℃ for 3 min  95℃ for 25 s  60℃ for 20 s 25 cycles  72℃ for 40 s  72℃ for 1 min | 95℃ for 3 min  95℃ for 25 s  60℃ for 20 s 25 cycles  72℃ for 40 s  72℃ for 1 min |
|  | 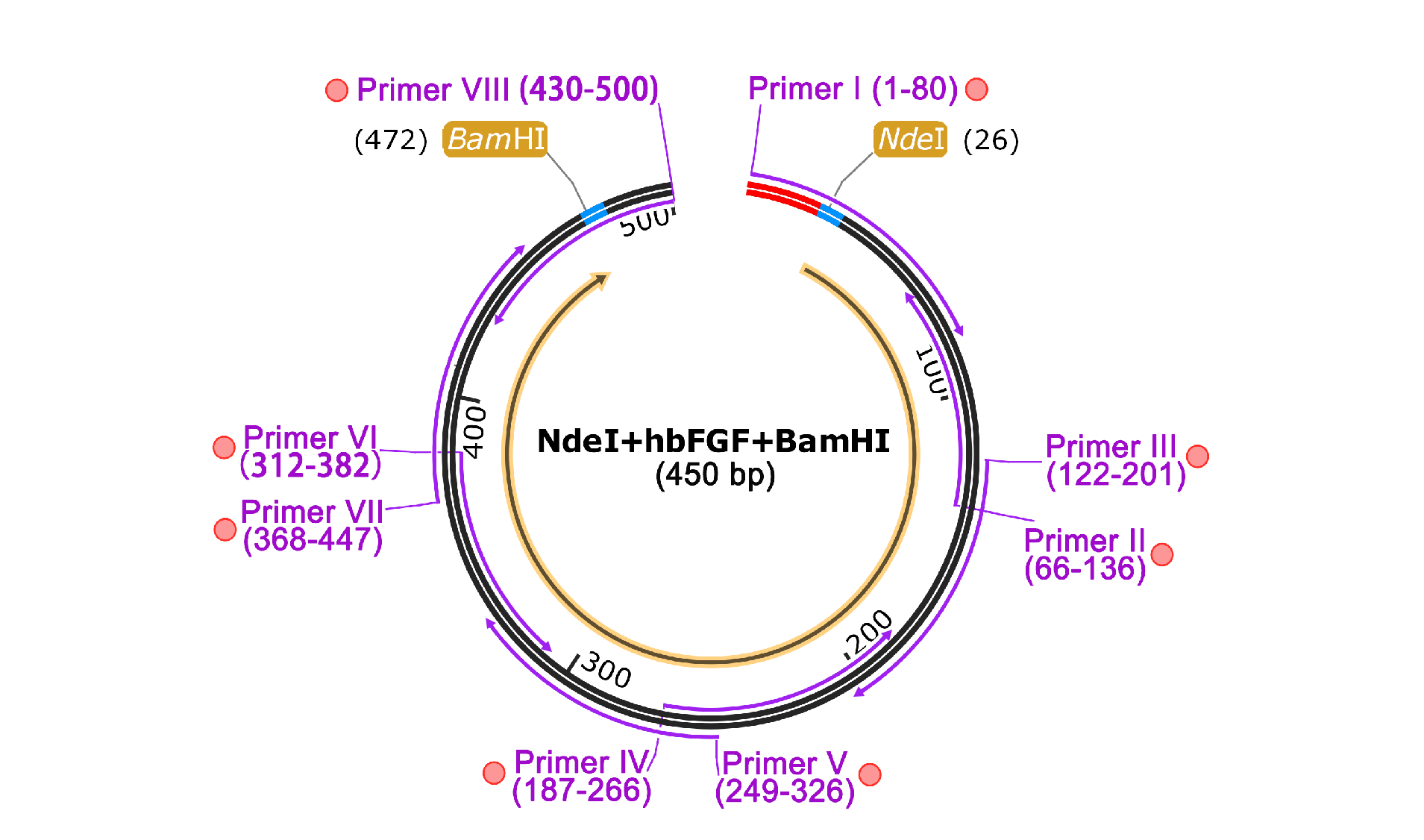 | 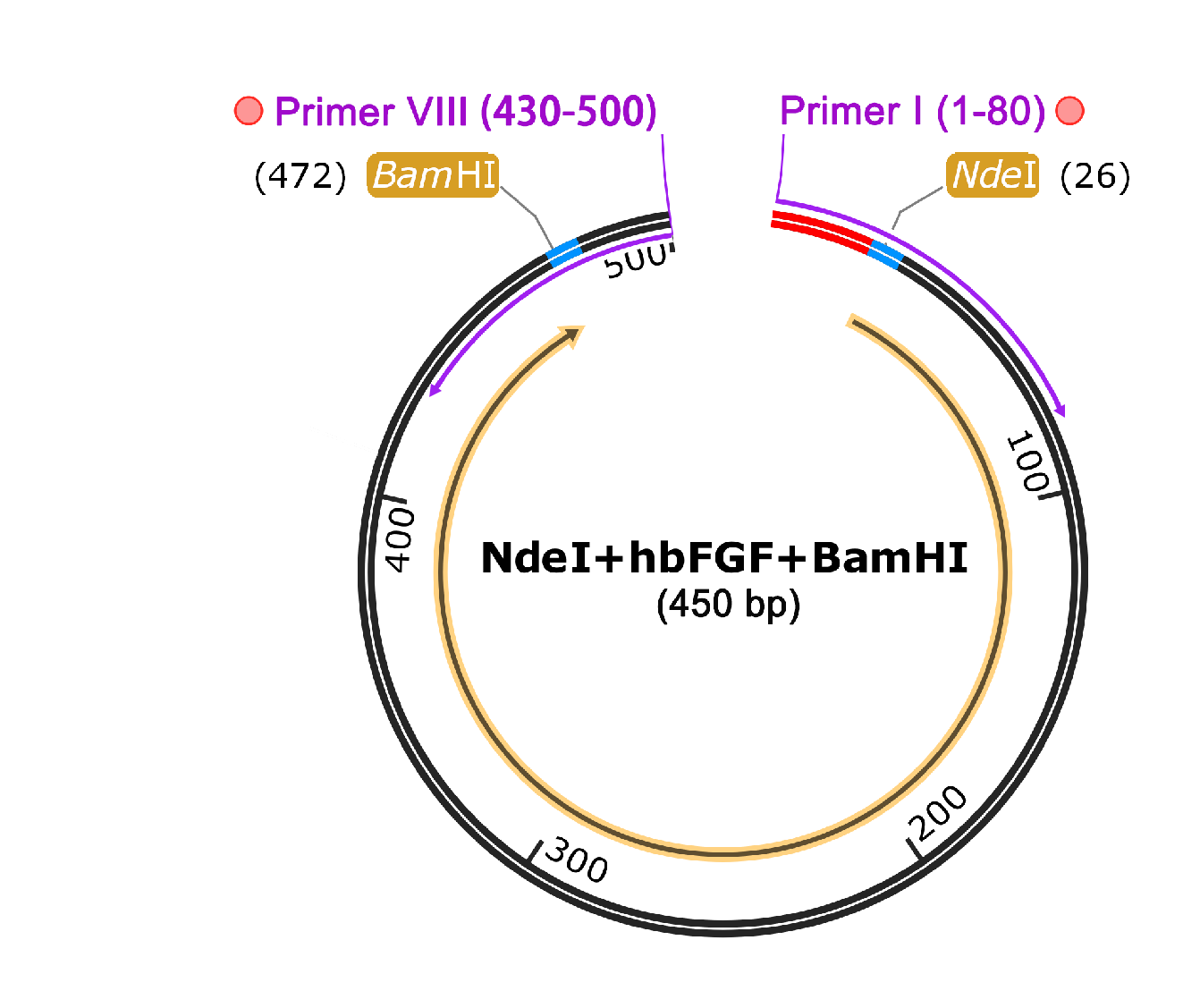 |
| Product recovery | The PCR products were detected by 1.0% agarose gel electrophoresis and the target DNA fragments were recovered by the gel extraction kit. | |

**Table S2.** The PCR primers

| Upstream Primers | PII  (71 bp) | 5’-GGTGGATGCGCAGGAAGAAGCCCCCGTTTTTGCAGTACAGCCGCTTGGGGTCCTTGAAGTGGCCGGGCGGG-3’ |
| --- | --- | --- |
|  | PIV  (80 bp) | 5’-TCTTCCTTCATAGCCAGGTAACGGTTAGCACACACTCCTTTGATAGACACAACTCCTCTCTCTTCTGCTTGAAGTTGTAG-3’ |
|  | PVI  (71 bp) | 5’-GTGCCACATACCAACTGGTGTATTTCCTTGACCGGTAAGTATTGTAGTTATTAGATTCCAATCGTTCAAAA-3’ |
|  | PVIII (71 bp) | 5’-CCTTTCGGGCTTTGTTAGCAGCC***GGATCC***TTAGCTCTTAGCAGACATTGGAAGAAAAAGTATAGCTTTCTG-3’  ***Bam*HI** |
| Downstream Primers | PI  (80 bp) | 5’-GTTTAACTTTAAGAAGGAGATATA***CATATG***CCAGCTTTGCCCGAGGATGGT  ***Nde*I**  GGTAGCGGCGCCTTCCCGCCCGGCCACTT-3’ |
|  | PIII  (80 bp) | 5’-TCCTGCGCATCCACCCCGACGGCCGAGTTGACGGGGTCCGGGAGAAGAGCGACCCTCATATAAAGCTACAACTTCAAGCA-3’ |
|  | PV  (78 bp) | 5’-CCTGGCTATGAAGGAAGATGGAAGATTACTGGCTTCTAAATGTGTTACGGATGAGTGTTTCTTTTTTGAACGATTGGA-3’ |
|  | PVII  (80 bp) | 5’-GTTGGTATGTGGCACTGAAACGAACTGGGCAGTATAAGCTTGGGTCTAAAACAGGACCTGGGCAGAAAGCTATACTTTTT-3’ |

**Table S3.** Design and result for the response surface experiment

| **Run** | **Factor** | | | | | **Response** | |
| --- | --- | --- | --- | --- | --- | --- | --- |
|  | **Temperature (℃)** | **pH** | **IPTG (mmol/L)** | **NH_4_Cl (g/L)** | **Induced time (h)** | **OD_600_** | **Expression level（%）** |
| 1 | 30 | 7 | 1 | 4 | 5 | 2.1 | 16.4 |
| 2 | 30 | 6 | 1 | 4 | 4 | 1.59 | 17.4 |
| 3 | 30 | 7 | 1 | 4 | 3 | 1.955 | 16.4 |
| 4 | 30 | 7 | 1 | 0 | 4 | 1.85 | 16.8 |
| 5 | 30 | 7 | 1.8 | 4 | 4 | 2.05 | 15.5 |
| 6 | 30 | 7 | 0.2 | 4 | 4 | 2.61 | 17.2 |
| 7 | 30 | 8 | 1 | 4 | 4 | 1.7 | 15.3 |
| 8 | 30 | 7 | 1 | 8 | 4 | 1.81 | 15.8 |
| 9 | 34 | 8 | 1 | 4 | 3 | 1.915 | 18.8 |
| 10 | 34 | 6 | 1.8 | 4 | 4 | 1.96 | 26.4 |
| 11 | 34 | 6 | 1 | 0 | 4 | 1.74 | 28.4 |
| 12 | 34 | 7 | 1 | 4 | 4 | 2.495 | 22.4 |
| 13 | 34 | 8 | 1 | 8 | 4 | 1.715 | 19 |
| 14 | 34 | 6 | 1 | 8 | 4 | 1.735 | 26.8 |
| 15 | 34 | 8 | 0.2 | 4 | 4 | 2.595 | 19.7 |
| 16 | 34 | 7 | 1 | 4 | 4 | 2.455 | 23 |
| 17 | 34 | 7 | 1 | 0 | 3 | 1.805 | 22.1 |
| 18 | 34 | 7 | 0.2 | 0 | 4 | 2.745 | 25.7 |
| 19 | 34 | 8 | 1.8 | 4 | 4 | 1.99 | 17.9 |
| 20 | 34 | 7 | 0.2 | 4 | 3 | 2.885 | 24.8 |
| 21 | 34 | 7 | 1.8 | 8 | 4 | 2.23 | 20.3 |
| 22 | 34 | 7 | 0.2 | 8 | 4 | 2.65 | 25 |
| 23 | 34 | 6 | 0.2 | 4 | 4 | 2.595 | 29.1 |
| 24 | 34 | 7 | 0.2 | 4 | 5 | 2.945 | 25.8 |
| 25 | 34 | 7 | 1.8 | 4 | 5 | 2.53 | 20.4 |
| 26 | 34 | 6 | 1 | 4 | 3 | 1.855 | 26.6 |
| 27 | 34 | 8 | 1 | 4 | 5 | 2.08 | 19.5 |
| 28 | 34 | 7 | 1.8 | 0 | 4 | 2.24 | 20.3 |
| 29 | 34 | 7 | 1 | 0 | 5 | 2.115 | 24.8 |
| 30 | 34 | 8 | 1 | 0 | 4 | 1.725 | 19.3 |
| 31 | 34 | 6 | 1 | 4 | 5 | 2.08 | 28.7 |
| 32 | 34 | 7 | 1 | 4 | 4 | 2.37 | 23 |
| 33 | 34 | 7 | 1 | 4 | 4 | 2.365 | 23.6 |
| 34 | 34 | 7 | 1 | 4 | 4 | 2.365 | 24 |
| 35 | 34 | 7 | 1.8 | 4 | 3 | 2.22 | 20 |
| 36 | 34 | 7 | 1 | 8 | 5 | 2.115 | 24.7 |
| 37 | 34 | 7 | 1 | 4 | 4 | 2.355 | 24.3 |
| 38 | 34 | 7 | 1 | 8 | 3 | 1.775 | 21.1 |
| 39 | 38 | 7 | 1.8 | 4 | 4 | 2.575 | 31.5 |
| 40 | 38 | 7 | 1 | 4 | 5 | 2.58 | 32.8 |
| 41 | 38 | 8 | 1 | 4 | 4 | 2.19 | 30.3 |
| 42 | 38 | 7 | 1 | 0 | 4 | 2.3 | 33.3 |
| 43 | 38 | 7 | 0.2 | 4 | 4 | 3 | 34.2 |
| 44 | 38 | 7 | 1 | 4 | 3 | 2.34 | 31.7 |
| 45 | 38 | 7 | 1 | 8 | 4 | 2.25 | 31.7 |
| 46 | 38 | 6 | 1 | 4 | 4 | 2.165 | 36.5 |

**Table S4.** ANOVA for response surface quadratic model on the response value of expression level

| **Factor** | **Sum of Squares** | **df** | **Mean Square** | **F value** | ***P* value** |
| --- | --- | --- | --- | --- | --- |
| Model | 1393.70 | 20 | 69.68 | 54.45 | < 0.0001^****^ |
| A-Temperature | 1075.84 | 1 | 1075.84 | 840.66 | < 0.0001^****^ |
| B-pH | 225.75 | 1 | 225.75 | 176.40 | < 0.0001^****^ |
| C-IPTG | 53.29 | 1 | 53.29 | 41.64 | < 0.0001^****^ |
| D-NH_4_Cl | 2.48 | 1 | 2.48 | 1.94 | 0.1761 |
| E-Induced time | 8.41 | 1 | 8.41 | 6.57 | 0.0168^*^ |
| AB | 4.20 | 1 | 4.20 | 3.28 | 0.0820 |
| AC | 0.25 | 1 | 0.25 | 0.20 | 0.6623 |
| AD | 0.090 | 1 | 0.090 | 0.070 | 0.7930 |
| AE | 0.30 | 1 | 0.30 | 0.24 | 0.6311 |
| BC | 0.20 | 1 | 0.20 | 0.16 | 0.6942 |
| BD | 0.42 | 1 | 0.42 | 0.33 | 0.5707 |
| BE | 0.49 | 1 | 0.49 | 0.38 | 0.5417 |
| CD | 0.12 | 1 | 0.12 | 0.096 | 0.7596 |
| CE | 0.090 | 1 | 0.090 | 0.070 | 0.7930 |
| DE | 0.20 | 1 | 0.20 | 0.16 | 0.6942 |
| A^2^ | 14.56 | 1 | 14.56 | 11.38 | 0.0024^**^ |
| B^2^ | 0.35 | 1 | 0.35 | 0.27 | 0.6061 |
| C^2^ | 0.74 | 1 | 0.74 | 0.58 | 0.4534 |
| D^2^ | 0.29 | 1 | 0.29 | 0.23 | 0.6363 |
| E^2^ | 0.44 | 1 | 0.44 | 0.35 | 0.5621 |
| Residual | 31.99 | 25 | 1.28 |  |  |
| *Lack of Fit* | 29.47 | 20 | 1.47 | 2.91 | 0.1195 |
| *Pure Error* | 2.53 | 5 | 0.51 |  |  |
| Cor Total | 1425.69 | 45 |  |  |  |

Expression level (%) = 23.38 + 8.20*A - 3.76*B - 1.83*C - 0.39*D + 0.72*E - 1.03*AB - 0.25*AC - 0.15*AD + 0.27*AE + 0.23*BC - 0.32*BD - 0.35*BE + 0.17*CD - 0.15*CE + 0.22*DE + 1.29*A^2^ + 0.2*B^2^ - 0.29*C^2^ - 0.18*D^2^ - 0.23*E^2^ ($\text{R}^{2}$=0.9790, ^^=0.9622)

**Table S5.** ANOVA for response surface quadratic model on the response value of OD_600_

| **Factor** | **Sum of Squares** | **df** | **Mean Square** | **F value** | ***P* value** |
| --- | --- | --- | --- | --- | --- |
| Model | 5.76 | 20 | 5.76 | 58.31 | < 0.0001^****^ |
| A-Temperature | 0.87 | 1 | 0.87 | 176.40 | < 0.0001^****^ |
| B-pH | 2.256E-003 | 1 | 2.256E-003 | 0.46 | 0.5055 |
| C-IPTG | 1.12 | 1 | 1.12 | 226.26 | < 0.0001^****^ |
| D-NH_4_Cl | 3.600E-003 | 1 | 3.600E-003 | 0.73 | 0.4015 |
| E-Induced time | 0.20 | 1 | 0.20 | 40.74 | < 0.0001^****^ |
| AB | 1.806E-003 | 1 | 1.806E-003 | 0.37 | 0.5509 |
| AC | 4.556E-003 | 1 | 4.556E-003 | 0.92 | 0.3462 |
| AD | 2.500E-005 | 1 | 2.500E-005 | 5.058E-003 | 0.9439 |
| AE | 2.256E-003 | 1 | 2.256E-003 | 0.46 | 0.5055 |
| BC | 2.250E-004 | 1 | 2.250E-004 | 0.046 | 0.8328 |
| BD | 6.250E-006 | 1 | 6.250E-006 | 1.264E-003 | 0.9719 |
| BE | 9.000E-004 | 1 | 9.000E-004 | 0.18 | 0.6732 |
| CD | 1.806E-003 | 1 | 1.806E-003 | 0.37 | 0.5509 |
| CE | 0.016 | 1 | 0.016 | 3.16 | 0.0876 |
| DE | 2.250E-004 | 1 | 2.250E-004 | 0.046 | 0.8328 |
| A^2^ | 0.082 | 1 | 0.082 | 16.68 | 0.0004^***^ |
| B^2^ | 1.28 | 1 | 1.28 | 259.60 | < 0.0001^****^ |
| C^2^ | 0.78 | 1 | 0.78 | 157.92 | < 0.0001^****^ |
| D^2^ | 0.71 | 1 | 0.71 | 144.36 | < 0.0001^****^ |
| E^2^ | 0.053 | 1 | 0.053 | 10.75 | 0.0031^**^ |
| Residual | 31.99 | 25 | 1.28 |  |  |
| *Lack of Fit* | 29.47 | 20 | 1.47 | 2.91 | 0.1195 |
| *Pure Error* | 2.53 | 5 | 0.51 |  |  |
| Cor Total | 1425.69 | 45 |  |  |  |

OD_600_= 2.4 + 0.23*A + 0.012*B - 0.26*C - 0.015*D + 0.11*E - 0.021*AB + 0.034*AC - 0.0025*AD + 0.024*AE + 0.0075*BC - 0.00125*BD - 0.015*BE + 0.021*CD + 0.062*CE + 0.0075*DE - 0.097*A^2^ - 0.38*B^2^ + 0.30*C^2^ - 0.29*D^2^ - 0.078*E^2^ ($\text{R}^{2}$=0.9776, ^^=0.9596)

**Table S6.** Genetic stability of the engineered strain in different volumes of LB medium

| **LB Medium** | **Passage number** | **Number of single colonies in plates** | | | **Plasmid loss rate (%)** | **Plasmid stabilization rate (%)** |
| --- | --- | --- | --- | --- | --- | --- |
|  |  | **non-resistant** | **Resistant^#^** | |  |  |
| 30 mL | 10 | 100 | | 100 | 0 | 100 |
|  | 20 | 100 | | 100 | 0 | 100 |
|  | 30 | 100 | | 100 | 0 | 100 |
| 300 mL | 10 | 100 | | 100 | 0 | 100 |
|  | 20 | 100 | | 100 | 0 | 100 |
|  | 30 | 100 | | 100 | 0 | 100 |

**^#^** The LB solid plate containing 100 µg/mL kanamycin sulfate.

**Table S7.** Plasmid loss rate during 200-L scale fermentation.

| **Batch number** | **Number of single colonies in plates** | | | **Plasmid loss rate (%)** | **Plasmid stabilization rate (%)** |
| --- | --- | --- | --- | --- | --- |
|  | **non-resistant** | **resistant** | |  |  |
| 1 | 100 | | 91 | 9 | 91 |
| 3 | 100 | | 92 | 8 | 92 |
| 3 | 100 | | 90 | 10 | 90 |

**^#^** The LB solid plate containing 100 µg/mL kanamycin sulfate.

**Table S8.** The expression level of rhbFGF in the engineered strain with storage time (Mean±SD).

|  | **Expression level of rhbFGF (%)** | | | | | |
| --- | --- | --- | --- | --- | --- | --- |
|  | **0 month** | **1 month** | **3 months** | **6 months** | **9 months** | **12 months** |
| **Mater seed strain^a^** | 26.0 | 25.9 | 26.0 | 25.9 | 26.0 | 26.0 |
| **Working seed strain^a^** | 26.0 | 26.0 | 25.5 | 25.0 | 24.8 | 23.0 |
| **Bacteria pellets^b^** | 28.2 ± 0.2 | 28.2 ± 0.2 | 28.0 ± 0.1 | 26.4 ± 0.3 | 23.2 ± 0.4 | / |

^a^ The bacteria pellets were harvested from 500-L scale fermentation and stored at -20 ± 5 ℃.

^b^ The seed strain was stored at -70 ± 5 ℃.

**Table S9.** Summary of the data of inoculation optimization at 30-L fermenter

| **Inoculation (%)** | **Volume of fermentation (L)** | **Bacterial wet weight (g)** | **Bacterial density (g/L)** | **Expression level (%)** |
| --- | --- | --- | --- | --- |
| 5 | 15.5 | 650 | 41.9 | 26.1 |
| 10 | 16.0 | 670 | 41.8 | 24.3 |
| 15 | 16.0 | 600 | 37.5 | 21.5 |
