# Supplementary material for: Scaling up production of recombinant human basic fibroblast growth factor in an Escherichia coli BL21(DE3) plysS strain and evaluation of its pro-wound healing efficacy
Source: Front Pharmacol. 2024 Feb 5;14:1279516. doi: 10.3389/fphar.2023.1279516 (PMC10875678; doi:10.3389/fphar.2023.1279516)
Supplement: Supplementary file 10 [file DataSheet12.ZIP › Table/Supplementary Table 1.docx]

**Table S1.** The synthesis and amplification process of hbFGF target gene

|  | Round 1: Overlap PCR | Round 2: Standard PCR |
| --- | --- | --- |
| Purpose | Synthesis of hbFGF target gene | Amplification of hbFGF target gene |
| 50 μL reaction system | 50 pmoL/μL primer PI-PVIII 0.5 μL  PCR polymerase (PV2) 0.5 μL  5 × PV2 buffer 10 μL  10 mM dNTP 1 μL  ddH_2_O 34.5 μL | Product of round 1 PCR 0.3 μL  50 pmoL/μL primer PI 0.5 μL  50 pmoL/μL primer PVIII 0.5 μL  PCR polymerase (PV2) 0.5 μL  5 × PV2 buffer 10 μL  10 mM dNTP 1 μL  ddH2O 37.2 μL |
| PCR protocol | 95℃ for 3 min  95℃ for 25 s  60℃ for 20 s 25 cycles  72℃ for 40 s  72℃ for 1 min | 95℃ for 3 min  95℃ for 25 s  60℃ for 20 s 25 cycles  72℃ for 40 s  72℃ for 1 min |
|  | 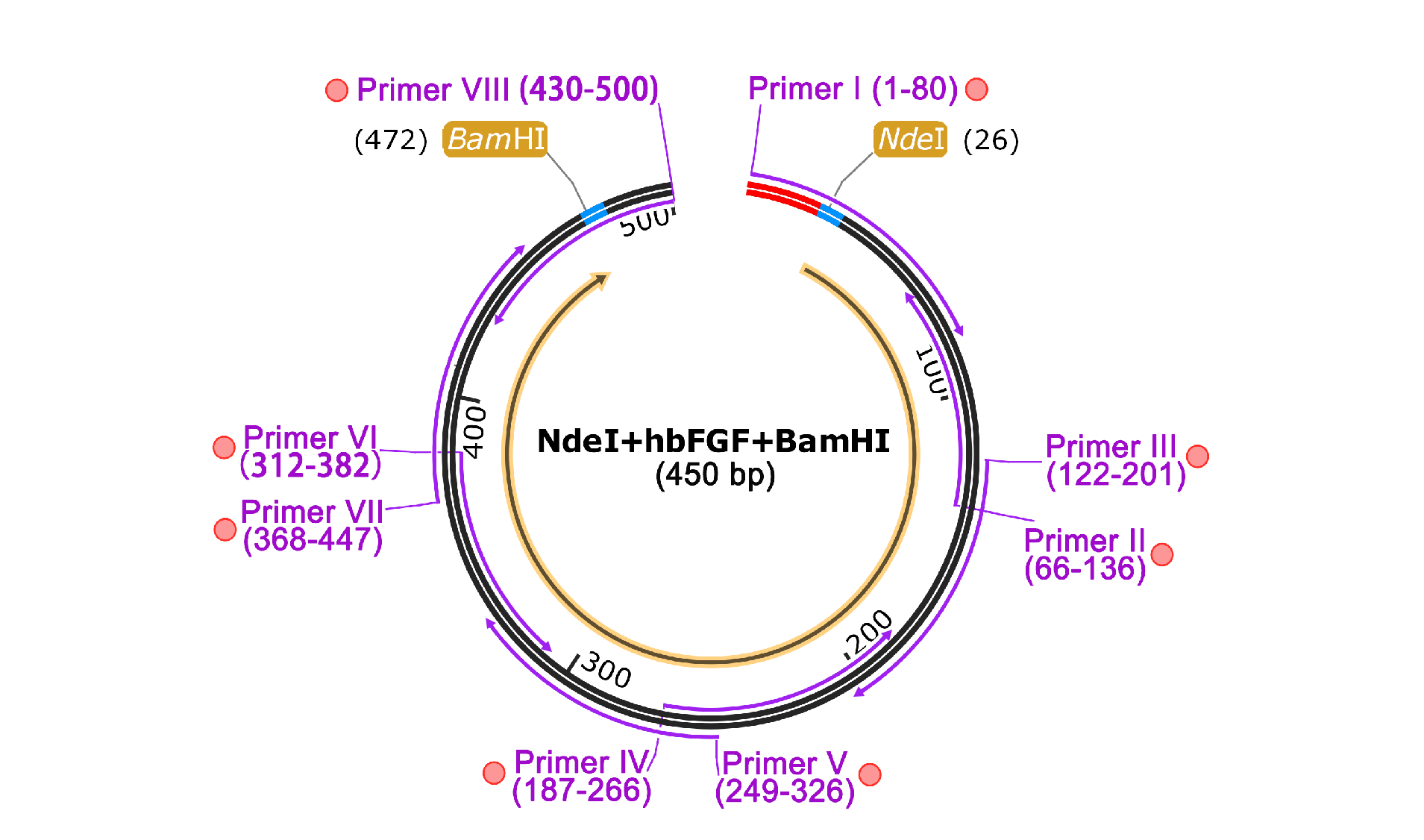 | 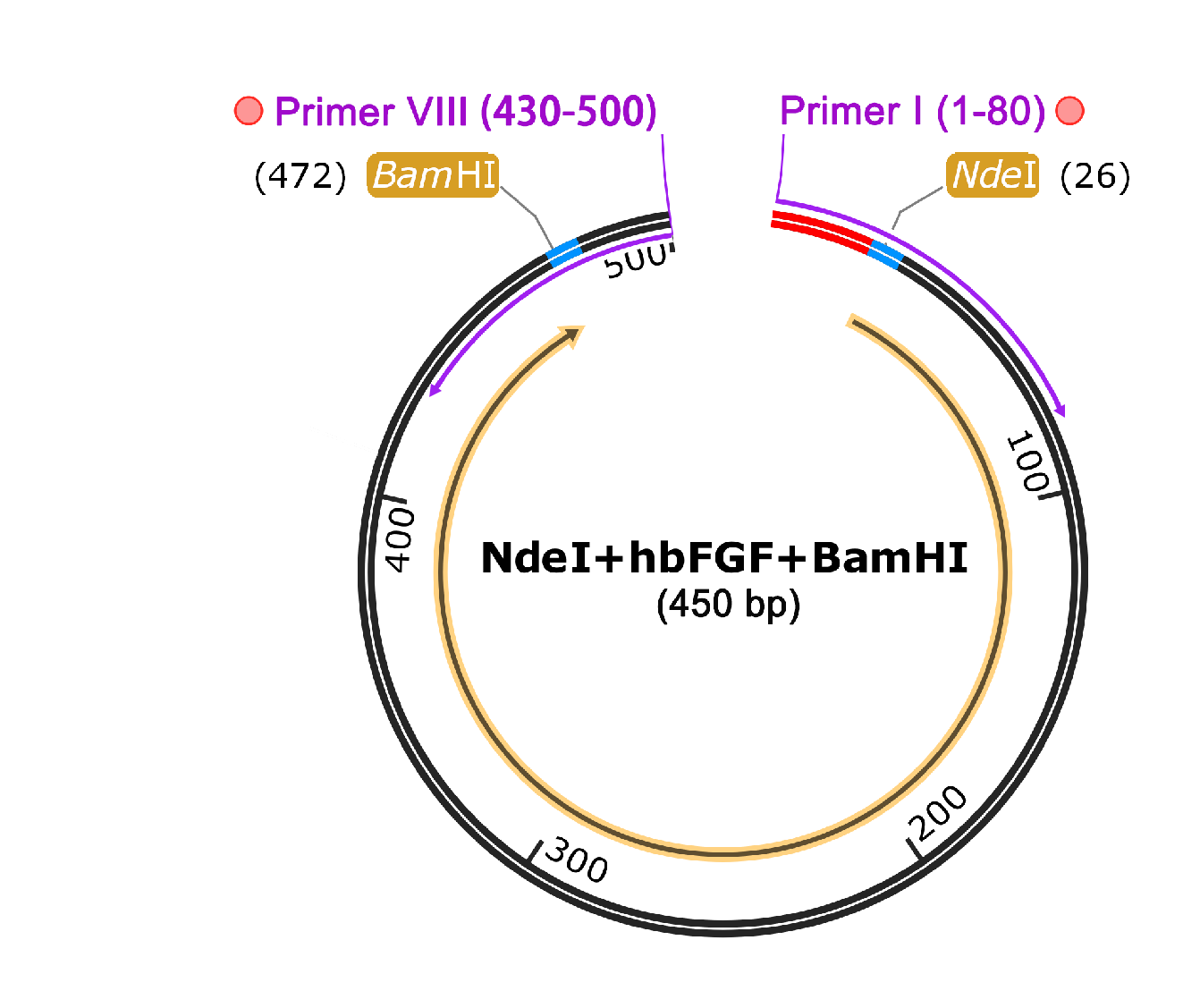 |
| Product recovery | The PCR products were detected by 1.0% agarose gel electrophoresis and the target DNA fragments were recovered by the gel extraction kit. | |
