# Supplementary material for: Scaling up production of recombinant human basic fibroblast growth factor in an Escherichia coli BL21(DE3) plysS strain and evaluation of its pro-wound healing efficacy
Source: Front Pharmacol. 2024 Feb 5;14:1279516. doi: 10.3389/fphar.2023.1279516 (PMC10875678; doi:10.3389/fphar.2023.1279516)
Supplement: Supplementary file 10 [file DataSheet12.ZIP › Table/Supplementary Table 2.docx]

**Table S2.** The PCR primers

| Upstream Primers | PII  (71 bp) | 5’-GGTGGATGCGCAGGAAGAAGCCCCCGTTTTTGCAGTACAGCCGCTTGGGGTCCTTGAAGTGGCCGGGCGGG-3’ |
| --- | --- | --- |
|  | PIV  (80 bp) | 5’-TCTTCCTTCATAGCCAGGTAACGGTTAGCACACACTCCTTTGATAGACACAACTCCTCTCTCTTCTGCTTGAAGTTGTAG-3’ |
|  | PVI  (71 bp) | 5’-GTGCCACATACCAACTGGTGTATTTCCTTGACCGGTAAGTATTGTAGTTATTAGATTCCAATCGTTCAAAA-3’ |
|  | PVIII (71 bp) | 5’-CCTTTCGGGCTTTGTTAGCAGCC***GGATCC***TTAGCTCTTAGCAGACATTGGAAGAAAAAGTATAGCTTTCTG-3’  ***Bam*HI** |
| Downstream Primers | PI  (80 bp) | 5’-GTTTAACTTTAAGAAGGAGATATA***CATATG***CCAGCTTTGCCCGAGGATGGT  ***Nde*I**  GGTAGCGGCGCCTTCCCGCCCGGCCACTT-3’ |
|  | PIII  (80 bp) | 5’-TCCTGCGCATCCACCCCGACGGCCGAGTTGACGGGGTCCGGGAGAAGAGCGACCCTCATATAAAGCTACAACTTCAAGCA-3’ |
|  | PV  (78 bp) | 5’-CCTGGCTATGAAGGAAGATGGAAGATTACTGGCTTCTAAATGTGTTACGGATGAGTGTTTCTTTTTTGAACGATTGGA-3’ |
|  | PVII  (80 bp) | 5’-GTTGGTATGTGGCACTGAAACGAACTGGGCAGTATAAGCTTGGGTCTAAAACAGGACCTGGGCAGAAAGCTATACTTTTT-3’ |
