# Supplementary material for: Scaling up production of recombinant human basic fibroblast growth factor in an Escherichia coli BL21(DE3) plysS strain and evaluation of its pro-wound healing efficacy
Source: Front Pharmacol. 2024 Feb 5;14:1279516. doi: 10.3389/fphar.2023.1279516 (PMC10875678; doi:10.3389/fphar.2023.1279516)
Supplement: Supplementary file 10 [file DataSheet12.ZIP › Table/Supplementary Table 3.docx]

| **Run** | **Factor** | | | | | **Response** | |
| --- | --- | --- | --- | --- | --- | --- | --- |
|  | **Temperature (℃)** | **pH** | **IPTG (mmol/L)** | **NH_4_Cl (g/L)** | **Induced time (h)** | **OD_600_** | **Expression level（%）** |
| 1 | 30 | 7 | 1 | 4 | 5 | 2.1 | 16.4 |
| 2 | 30 | 6 | 1 | 4 | 4 | 1.59 | 17.4 |
| 3 | 30 | 7 | 1 | 4 | 3 | 1.955 | 16.4 |
| 4 | 30 | 7 | 1 | 0 | 4 | 1.85 | 16.8 |
| 5 | 30 | 7 | 1.8 | 4 | 4 | 2.05 | 15.5 |
| 6 | 30 | 7 | 0.2 | 4 | 4 | 2.61 | 17.2 |
| 7 | 30 | 8 | 1 | 4 | 4 | 1.7 | 15.3 |
| 8 | 30 | 7 | 1 | 8 | 4 | 1.81 | 15.8 |
| 9 | 34 | 8 | 1 | 4 | 3 | 1.915 | 18.8 |
| 10 | 34 | 6 | 1.8 | 4 | 4 | 1.96 | 26.4 |
| 11 | 34 | 6 | 1 | 0 | 4 | 1.74 | 28.4 |
| 12 | 34 | 7 | 1 | 4 | 4 | 2.495 | 22.4 |
| 13 | 34 | 8 | 1 | 8 | 4 | 1.715 | 19 |
| 14 | 34 | 6 | 1 | 8 | 4 | 1.735 | 26.8 |
| 15 | 34 | 8 | 0.2 | 4 | 4 | 2.595 | 19.7 |
| 16 | 34 | 7 | 1 | 4 | 4 | 2.455 | 23 |
| 17 | 34 | 7 | 1 | 0 | 3 | 1.805 | 22.1 |
| 18 | 34 | 7 | 0.2 | 0 | 4 | 2.745 | 25.7 |
| 19 | 34 | 8 | 1.8 | 4 | 4 | 1.99 | 17.9 |
| 20 | 34 | 7 | 0.2 | 4 | 3 | 2.885 | 24.8 |
| 21 | 34 | 7 | 1.8 | 8 | 4 | 2.23 | 20.3 |
| 22 | 34 | 7 | 0.2 | 8 | 4 | 2.65 | 25 |
| 23 | 34 | 6 | 0.2 | 4 | 4 | 2.595 | 29.1 |
| 24 | 34 | 7 | 0.2 | 4 | 5 | 2.945 | 25.8 |
| 25 | 34 | 7 | 1.8 | 4 | 5 | 2.53 | 20.4 |
| 26 | 34 | 6 | 1 | 4 | 3 | 1.855 | 26.6 |
| 27 | 34 | 8 | 1 | 4 | 5 | 2.08 | 19.5 |
| 28 | 34 | 7 | 1.8 | 0 | 4 | 2.24 | 20.3 |
| 29 | 34 | 7 | 1 | 0 | 5 | 2.115 | 24.8 |
| 30 | 34 | 8 | 1 | 0 | 4 | 1.725 | 19.3 |
| 31 | 34 | 6 | 1 | 4 | 5 | 2.08 | 28.7 |
| 32 | 34 | 7 | 1 | 4 | 4 | 2.37 | 23 |
| 33 | 34 | 7 | 1 | 4 | 4 | 2.365 | 23.6 |
| 34 | 34 | 7 | 1 | 4 | 4 | 2.365 | 24 |
| 35 | 34 | 7 | 1.8 | 4 | 3 | 2.22 | 20 |
| 36 | 34 | 7 | 1 | 8 | 5 | 2.115 | 24.7 |
| 37 | 34 | 7 | 1 | 4 | 4 | 2.355 | 24.3 |
| 38 | 34 | 7 | 1 | 8 | 3 | 1.775 | 21.1 |
| 39 | 38 | 7 | 1.8 | 4 | 4 | 2.575 | 31.5 |
| 40 | 38 | 7 | 1 | 4 | 5 | 2.58 | 32.8 |
| 41 | 38 | 8 | 1 | 4 | 4 | 2.19 | 30.3 |
| 42 | 38 | 7 | 1 | 0 | 4 | 2.3 | 33.3 |
| 43 | 38 | 7 | 0.2 | 4 | 4 | 3 | 34.2 |
| 44 | 38 | 7 | 1 | 4 | 3 | 2.34 | 31.7 |
| 45 | 38 | 7 | 1 | 8 | 4 | 2.25 | 31.7 |
| 46 | 38 | 6 | 1 | 4 | 4 | 2.165 | 36.5 |

**Table S3.** Design and result for the response surface experiment
