# Supplementary material for: Scaling up production of recombinant human basic fibroblast growth factor in an Escherichia coli BL21(DE3) plysS strain and evaluation of its pro-wound healing efficacy
Source: Front Pharmacol. 2024 Feb 5;14:1279516. doi: 10.3389/fphar.2023.1279516 (PMC10875678; doi:10.3389/fphar.2023.1279516)
Supplement: Supplementary file 10 [file DataSheet12.ZIP › Table/Supplementary Table 4.docx]

**Table S4.** ANOVA for response surface quadratic model on the response value of expression level

| **Factor** | **Sum of Squares** | **df** | **Mean Square** | **F value** | ***P* value** |
| --- | --- | --- | --- | --- | --- |
| Model | 1393.70 | 20 | 69.68 | 54.45 | < 0.0001^****^ |
| A-Temperature | 1075.84 | 1 | 1075.84 | 840.66 | < 0.0001^****^ |
| B-pH | 225.75 | 1 | 225.75 | 176.40 | < 0.0001^****^ |
| C-IPTG | 53.29 | 1 | 53.29 | 41.64 | < 0.0001^****^ |
| D-NH_4_Cl | 2.48 | 1 | 2.48 | 1.94 | 0.1761 |
| E-Induced time | 8.41 | 1 | 8.41 | 6.57 | 0.0168^*^ |
| AB | 4.20 | 1 | 4.20 | 3.28 | 0.0820 |
| AC | 0.25 | 1 | 0.25 | 0.20 | 0.6623 |
| AD | 0.090 | 1 | 0.090 | 0.070 | 0.7930 |
| AE | 0.30 | 1 | 0.30 | 0.24 | 0.6311 |
| BC | 0.20 | 1 | 0.20 | 0.16 | 0.6942 |
| BD | 0.42 | 1 | 0.42 | 0.33 | 0.5707 |
| BE | 0.49 | 1 | 0.49 | 0.38 | 0.5417 |
| CD | 0.12 | 1 | 0.12 | 0.096 | 0.7596 |
| CE | 0.090 | 1 | 0.090 | 0.070 | 0.7930 |
| DE | 0.20 | 1 | 0.20 | 0.16 | 0.6942 |
| A^2^ | 14.56 | 1 | 14.56 | 11.38 | 0.0024^**^ |
| B^2^ | 0.35 | 1 | 0.35 | 0.27 | 0.6061 |
| C^2^ | 0.74 | 1 | 0.74 | 0.58 | 0.4534 |
| D^2^ | 0.29 | 1 | 0.29 | 0.23 | 0.6363 |
| E^2^ | 0.44 | 1 | 0.44 | 0.35 | 0.5621 |
| Residual | 31.99 | 25 | 1.28 |  |  |
| *Lack of Fit* | 29.47 | 20 | 1.47 | 2.91 | 0.1195 |
| *Pure Error* | 2.53 | 5 | 0.51 |  |  |
| Cor Total | 1425.69 | 45 |  |  |  |

Expression level (%) = 23.38 + 8.20*A - 3.76*B - 1.83*C - 0.39*D + 0.72*E - 1.03*AB - 0.25*AC - 0.15*AD + 0.27*AE + 0.23*BC - 0.32*BD - 0.35*BE + 0.17*CD - 0.15*CE + 0.22*DE + 1.29*A^2^ + 0.2*B^2^ - 0.29*C^2^ - 0.18*D^2^ - 0.23*E^2^ ($\text{R}^{2}$=0.9790, ^^=0.9622)
