# Supplementary material for: Scaling up production of recombinant human basic fibroblast growth factor in an Escherichia coli BL21(DE3) plysS strain and evaluation of its pro-wound healing efficacy
Source: Front Pharmacol. 2024 Feb 5;14:1279516. doi: 10.3389/fphar.2023.1279516 (PMC10875678; doi:10.3389/fphar.2023.1279516)
Supplement: Supplementary file 10 [file DataSheet12.ZIP › Table/Supplementary Table 5.docx]

**Table S5.** ANOVA for response surface quadratic model on the response value of OD_600_

| **Factor** | **Sum of Squares** | **df** | **Mean Square** | **F value** | ***P* value** |
| --- | --- | --- | --- | --- | --- |
| Model | 5.76 | 20 | 5.76 | 58.31 | < 0.0001^****^ |
| A-Temperature | 0.87 | 1 | 0.87 | 176.40 | < 0.0001^****^ |
| B-pH | 2.256E-003 | 1 | 2.256E-003 | 0.46 | 0.5055 |
| C-IPTG | 1.12 | 1 | 1.12 | 226.26 | < 0.0001^****^ |
| D-NH_4_Cl | 3.600E-003 | 1 | 3.600E-003 | 0.73 | 0.4015 |
| E-Induced time | 0.20 | 1 | 0.20 | 40.74 | < 0.0001^****^ |
| AB | 1.806E-003 | 1 | 1.806E-003 | 0.37 | 0.5509 |
| AC | 4.556E-003 | 1 | 4.556E-003 | 0.92 | 0.3462 |
| AD | 2.500E-005 | 1 | 2.500E-005 | 5.058E-003 | 0.9439 |
| AE | 2.256E-003 | 1 | 2.256E-003 | 0.46 | 0.5055 |
| BC | 2.250E-004 | 1 | 2.250E-004 | 0.046 | 0.8328 |
| BD | 6.250E-006 | 1 | 6.250E-006 | 1.264E-003 | 0.9719 |
| BE | 9.000E-004 | 1 | 9.000E-004 | 0.18 | 0.6732 |
| CD | 1.806E-003 | 1 | 1.806E-003 | 0.37 | 0.5509 |
| CE | 0.016 | 1 | 0.016 | 3.16 | 0.0876 |
| DE | 2.250E-004 | 1 | 2.250E-004 | 0.046 | 0.8328 |
| A^2^ | 0.082 | 1 | 0.082 | 16.68 | 0.0004^***^ |
| B^2^ | 1.28 | 1 | 1.28 | 259.60 | < 0.0001^****^ |
| C^2^ | 0.78 | 1 | 0.78 | 157.92 | < 0.0001^****^ |
| D^2^ | 0.71 | 1 | 0.71 | 144.36 | < 0.0001^****^ |
| E^2^ | 0.053 | 1 | 0.053 | 10.75 | 0.0031^**^ |
| Residual | 31.99 | 25 | 1.28 |  |  |
| *Lack of Fit* | 29.47 | 20 | 1.47 | 2.91 | 0.1195 |
| *Pure Error* | 2.53 | 5 | 0.51 |  |  |
| Cor Total | 1425.69 | 45 |  |  |  |

OD_600_= 2.4 + 0.23*A + 0.012*B - 0.26*C - 0.015*D + 0.11*E - 0.021*AB + 0.034*AC - 0.0025*AD + 0.024*AE + 0.0075*BC - 0.00125*BD - 0.015*BE + 0.021*CD + 0.062*CE + 0.0075*DE - 0.097*A^2^ - 0.38*B^2^ + 0.30*C^2^ - 0.29*D^2^ - 0.078*E^2^ ($\text{R}^{2}$=0.9776, ^^=0.9596)
