# Supplementary material for: Scaling up production of recombinant human basic fibroblast growth factor in an Escherichia coli BL21(DE3) plysS strain and evaluation of its pro-wound healing efficacy
Source: Front Pharmacol. 2024 Feb 5;14:1279516. doi: 10.3389/fphar.2023.1279516 (PMC10875678; doi:10.3389/fphar.2023.1279516)
Supplement: Supplementary file 10 [file DataSheet12.ZIP › Table/Supplementary Table 6.docx]

**Table S6.** Genetic stability of the engineered strain in different volumes of LB medium

| **LB Medium** | **Passage number** | **Number of single colonies in plates** | | | **Plasmid loss rate (%)** | **Plasmid stabilization rate (%)** |
| --- | --- | --- | --- | --- | --- | --- |
|  |  | **non-resistant** | **Resistant^#^** | |  |  |
| 30 mL | 10 | 100 | | 100 | 0 | 100 |
|  | 20 | 100 | | 100 | 0 | 100 |
|  | 30 | 100 | | 100 | 0 | 100 |
| 300 mL | 10 | 100 | | 100 | 0 | 100 |
|  | 20 | 100 | | 100 | 0 | 100 |
|  | 30 | 100 | | 100 | 0 | 100 |

**^#^** The LB solid plate containing 100 µg/mL kanamycin sulfate.
