# Supplementary material for: Scaling up production of recombinant human basic fibroblast growth factor in an Escherichia coli BL21(DE3) plysS strain and evaluation of its pro-wound healing efficacy
Source: Front Pharmacol. 2024 Feb 5;14:1279516. doi: 10.3389/fphar.2023.1279516 (PMC10875678; doi:10.3389/fphar.2023.1279516)
Supplement: Supplementary file 10 [file DataSheet12.ZIP › Table/Supplementary Table 7.docx]

**Table S7.** Plasmid loss rate during 200-L scale fermentation.

| **Batch number** | **Number of single colonies in plates** | | | **Plasmid loss rate (%)** | **Plasmid stabilization rate (%)** |
| --- | --- | --- | --- | --- | --- |
|  | **non-resistant** | **resistant** | |  |  |
| 1 | 100 | | 91 | 9 | 91 |
| 3 | 100 | | 92 | 8 | 92 |
| 3 | 100 | | 90 | 10 | 90 |

**^#^** The LB solid plate containing 100 µg/mL kanamycin sulfate.
