# Supplementary material for: Scaling up production of recombinant human basic fibroblast growth factor in an Escherichia coli BL21(DE3) plysS strain and evaluation of its pro-wound healing efficacy
Source: Front Pharmacol. 2024 Feb 5;14:1279516. doi: 10.3389/fphar.2023.1279516 (PMC10875678; doi:10.3389/fphar.2023.1279516)
Supplement: Supplementary file 10 [file DataSheet12.ZIP › Table/Supplementary Table 8.docx]

**Table S8.** The expression level of rhbFGF in the engineered strain with storage time (Mean±SD).

|  | **Expression level of hbFGF (%)** | | | | | |
| --- | --- | --- | --- | --- | --- | --- |
|  | **0 month** | **1 month** | **3 months** | **6 months** | **9 months** | **12 months** |
| **Mater seed strain^a^** | 26.0 | 25.9 | 26.0 | 25.9 | 26.0 | 26.0 |
| **Working seed strain^a^** | 26.0 | 26.0 | 25.5 | 25.0 | 24.8 | 23.0 |
| **Bacteria pellets^b^** | 28.2 ± 0.2 | 28.2 ± 0.2 | 28.0 ± 0.1 | 26.4 ± 0.3 | 23.2 ± 0.4 | / |

^a^ The bacteria pellets were harvested from 500-L scale fermentation and stored at -20 ± 5 ℃.

^b^ The seed strain was stored at -70 ± 5 ℃.
