# Supplementary material for: Scaling up production of recombinant human basic fibroblast growth factor in an Escherichia coli BL21(DE3) plysS strain and evaluation of its pro-wound healing efficacy
Source: Front Pharmacol. 2024 Feb 5;14:1279516. doi: 10.3389/fphar.2023.1279516 (PMC10875678; doi:10.3389/fphar.2023.1279516)
Supplement: Supplementary file 10 [file DataSheet12.ZIP › Table/Supplementary Table 9.docx]

**Table S9.** Summary of the data of inoculation optimization at 30-L fermenter

| **Inoculation (%)** | **Volume of fermentation (L)** | **Bacterial wet weight (g)** | **Bacterial density (g/L)** | **Expression level (%)** |
| --- | --- | --- | --- | --- |
| 5 | 15.5 | 650 | 41.9 | 26.1 |
| 10 | 16.0 | 670 | 41.8 | 24.3 |
| 15 | 16.0 | 600 | 37.5 | 21.5 |
