# Supplementary material for: Scaling up production of recombinant human basic fibroblast growth factor in an Escherichia coli BL21(DE3) plysS strain and evaluation of its pro-wound healing efficacy
Source: Front Pharmacol. 2024 Feb 5;14:1279516. doi: 10.3389/fphar.2023.1279516 (PMC10875678; doi:10.3389/fphar.2023.1279516)
Supplement: Supplementary file 10 [file DataSheet12.ZIP › Table/Table 1-5.docx]

**Table 1a.** Tested cultural conditions for hbFGF production on a flask scale

|  | **Factors** | **Level** | | | | | |
| --- | --- | --- | --- | --- | --- | --- | --- |
|  |  | **1** | **2** | **3** | **4** | **5** | **6** |
| **Growth parameter optimization** | inoculum volume (%, v/v) | 5 | 10 | 15 | / | / | / |
|  | Temperature (°C) | 32 | 34 | 36 | 38 | / | / |
|  | Dissolved oxygen ^a^ (mL) | 25 | 50 | 75 | 100 | / | / |
|  | pH | 6.6 | 6.8 | 7.0 | 7.2 | 7.4 | / |
|  | Glucose (g/L) | 0.5 | 1 | 2 | 5 | 10 | 20 |
|  | Induced OD_600_ | 0.2 | 0.4 | 0.8 | 1.2 | 1.8 | 2.4 |
| **Table 1b.** Independent variables and their levels used in the Box–Behnken design (BBD) | | | | | | | |
| **Expression parameter optimization (RSM)** |  | **Level** | | |  |  |  |
|  |  | **-1** | **0** | **1** |  |  |  |
|  | Temperature (°C) | 30 | 34 | 38 |  |  |  |
|  | pH | 6 | 7 | 8 |  |  |  |
|  | IPTG (mmol/L) | 0.2 | 1 | 1.8 |  |  |  |
|  | NH_4_Cl (g/L) | 0 | 0.09 | 0.18 |  |  |  |
|  | Induced time (h) | 3 | 4 | 5 |  |  |  |

(a: During optimization, following measurement with dissolved oxygen electrode, the dissolved oxygen ≥25%, when the volume of medium was 30 mL in 250-mL shake flask; the dissolved oxygen <25%, when the volume of medium was 50, 70, or 100 mL in 250-mL shake flask.)

**Table 2.** The optimal induction conditions for the hbFGF fermentation

| **Temperature (°C)** | **pH** | **IPTG (mmol/L)** | **NH_4_Cl (g/L)** | **Induced time (h)** |
| --- | --- | --- | --- | --- |
| 38.00 | 6.515 | 0.2 | 0.079 | 4.858 |

**Table 3.** Summary of scale-up fermentation data for hbFGF (Mean±SD, n≥3)

|  |  | **Expression level (%)** | **Bacterial density (g/L)** | **Bacterial wet weight (g)** |
| --- | --- | --- | --- | --- |
| **Conventional** | 200-L | 18.2 ± 2.2 | 40.4 ± 2.1 | 2624 ± 129 |
| **Post-optimization** | 200-L | 27.2 ± 0.8**** | 43.6 ± 0.6** | 2912 ± 137** |
|  | 500-L | 28.2 ± 0.2**** | 46.8 ± 0.3*** | 7797 ± 73**** |

Compared with the 200-L conventional fermentation (temperature 37 °C, pH 7.0, IPTG 1.0 mM, induction time 4 h), 0.001 < ***p* < 0.01, 0.0001 < ****p* < 0.001, *****p* < 0.0001.

**Table 4** Summary of the purification process for hbFGF (Mean±SD, n = 4)

| **Steps of purification** | **Volume of purification (mL)** | **Total protein (mg)** | **Target protein (mg)** | **SDS-PAGE Purity (%)** | **Recovery (%)** |
| --- | --- | --- | --- | --- | --- |
| Bacteria lysis | 8000 ± 0  (893.6 ± 23.2 g)^a^ | 153,420 ± 28,430.8 | 37,532.5 ± 6097.8 | 24.6 ± 1.8 | / |
| CM-Sepharose | 1422 ±278.3 | 3236.6 ± 97.8 | 2458.4 ± 223.6 | 75.9 ± 5.0 | 2.2 ± 0.4 |
| Heparin affinity | 274.8 ± 23.1 | 2501.7 ± 141.9 | 2279.0 ± 133.4 | 91.2 ± 5.9 | 77.4 ± 5.6 |
| SP-Sepharose | 858.5 ± 88.3 | 2036.3 ± 92.9 | 2013.9 ± 93.6 | 98.9 ± 0.9 | 81.7 ± 7.8 |
| Protein yield (mg/1 L culture): 114.6 ± 5.9 | | | | | |

a: The wet weight of bacteria for a single batch purification process.

**Table 5.** Comparison of hbFGF expressed for various hosts

| **Host** | **Fermentation Scale** | **Yield** | **Purity** | **Reference** |
| --- | --- | --- | --- | --- |
| *E. coli* DH5α ^a^ | 5 L | / | / | Sheng et al. (1999) |
| *E. coli* JM109 ^a^ | 40 L | 63.7 mg/L | 98.4% | Zhang et al. (2002) |
| *E. coli* DH5α ^a^ | 30 L | / | / | Bai et al. (2002) |
| *E. coli* JM109 ^a^ | 40 L | 97.5 mg/L | / | Feng et al. (2004) |
| *E. coli* JM109 ^a^ | 150 L | / | / | Wang et al. (2007) |
| *E. coli* BL21(DE3) ^a^ | 2 L | 105.3 mg/L | 98% | Chen et al. (2012) |
| *E. coli* BL(DE3)plysS ^a^ | 5 L | 94.8 mg/L | / | Liao et al. (2002) |
| 1. *coli* BL21(DE3) ^b^ | 1 L | 25–35 mg/L | / | Sheng et al. (2003) |
| *E. coli* BL21(DE3) ^c^ | / | 60–80 mg/L | 95% | Imsoonthornruksa et al. (2015) |
| *E. coli* BL21(DE3) ^d^ | / | / | / | Rassouli et al. (2013) |
| *E. coli* BL21(DE3) ^d^ | / | / | / | Soleyman et al. (2016) |
| *E. coli* BL21(DE3) ^e^ | 0.25 L | / | / | Dong et al. (2021) |
| *E. coli* BL21(DE3) ^f^ | 5 L | 1.42 g/L | 96% | Rahman et al. (2020) |
| *Pichia pastoris* ^g^ | / | 91 mg/L | > 94% | Mu et al. (2008) |
| *Pichia pastoris* ^g^ | / | 0.85 mg/L | 98.8% | Le et al. (2020) |
| *Bacillus subtilis* ^a^ | / | 40 mg/L | / | Kwong et al. (2013) |
| *Bacillus subtilis* ^g^ | 2 L | 84 mg/L | / | Hu et al. (2018) |
| *A. thaliana* ^g^ | / | 89.95 ng/mg oil body | / | Yang et al. (2018) |
| Soybean seed ^h^ | / | / | / | Ding et al. (2006) |
| *E. coli* BL(DE3)plysS | 500 L | 114.6 ± 5.9 mg/L | > 99% | Present study |

^a^ hbFGF_155_; ^b^ GST-hbFGF_155_; ^c^ 6His-hbFGF & Trx-6His-hbFGF; ^d^ His-hbFGF_146_; ^e^ Trx-hbFGF_146_; ^f^ Scl2-M-hbFGF;

^g^ hbFGF_146_; ^h^ bbFGF_155_
