# Supplementary material for: Scaling up production of recombinant human basic fibroblast growth factor in an Escherichia coli BL21(DE3) plysS strain and evaluation of its pro-wound healing efficacy
Source: Front Pharmacol. 2024 Feb 5;14:1279516. doi: 10.3389/fphar.2023.1279516 (PMC10875678; doi:10.3389/fphar.2023.1279516)
Supplement: Supplementary file 10 [file DataSheet12.ZIP › Table/Table 1.docx]

**Table 1a.** Tested cultural conditions for hbFGF production on a flask scale

|  | **Factors** | **Level** | | | | | |
| --- | --- | --- | --- | --- | --- | --- | --- |
|  |  | **1** | **2** | **3** | **4** | **5** | **6** |
| **Growth parameter optimization** | inoculum volume (%, v/v) | 5 | 10 | 15 | / | / | / |
|  | Temperature (°C) | 32 | 34 | 36 | 38 | / | / |
|  | Dissolved oxygen ^a^ (mL) | 25 | 50 | 75 | 100 | / | / |
|  | pH | 6.6 | 6.8 | 7.0 | 7.2 | 7.4 | / |
|  | Glucose (g/L) | 0.5 | 1 | 2 | 5 | 10 | 20 |
|  | Induced OD_600_ | 0.2 | 0.4 | 0.8 | 1.2 | 1.8 | 2.4 |
| **Table 1b.** Independent variables and their levels used in the Box–Behnken design (BBD) | | | | | | | |
| **Expression parameter optimization (RSM)** |  | **Level** | | |  |  |  |
|  |  | **-1** | **0** | **1** |  |  |  |
|  | Temperature (°C) | 30 | 34 | 38 |  |  |  |
|  | pH | 6 | 7 | 8 |  |  |  |
|  | IPTG (mmol/L) | 0.2 | 1 | 1.8 |  |  |  |
|  | NH_4_Cl (g/L) | 0 | 0.09 | 0.18 |  |  |  |
|  | Induced time (h) | 3 | 4 | 5 |  |  |  |

(a: During optimization, following measurement with dissolved oxygen electrode, the dissolved oxygen ≥25%, when the volume of medium was 30 mL in 250-mL shake flask; the dissolved oxygen <25%, when the volume of medium was 50, 70, or 100 mL in 250-mL shake flask.)
