# Supplementary material for: Scaling up production of recombinant human basic fibroblast growth factor in an Escherichia coli BL21(DE3) plysS strain and evaluation of its pro-wound healing efficacy
Source: Front Pharmacol. 2024 Feb 5;14:1279516. doi: 10.3389/fphar.2023.1279516 (PMC10875678; doi:10.3389/fphar.2023.1279516)
Supplement: Supplementary file 10 [file DataSheet12.ZIP › Table/Table 2.docx]

**Table 2.** The optimal induction conditions for the hbFGF fermentation

| **Temperature (°C)** | **pH** | **IPTG (mmol/L)** | **NH_4_Cl (g/L)** | **Induced time (h)** |
| --- | --- | --- | --- | --- |
| 38.00 | 6.515 | 0.2 | 0.079 | 4.858 |
