# Supplementary material for: Scaling up production of recombinant human basic fibroblast growth factor in an Escherichia coli BL21(DE3) plysS strain and evaluation of its pro-wound healing efficacy
Source: Front Pharmacol. 2024 Feb 5;14:1279516. doi: 10.3389/fphar.2023.1279516 (PMC10875678; doi:10.3389/fphar.2023.1279516)
Supplement: Supplementary file 10 [file DataSheet12.ZIP › Table/Table 3.docx]

**Table 3.** Summary of scale-up fermentation data for hbFGF (Mean±SD, n≥3)

|  |  | **Expression level (%)** | **Bacterial density (g/L)** | **Bacterial wet weight (g)** |
| --- | --- | --- | --- | --- |
| **Conventional** | 200-L | 18.2 ± 2.2 | 40.4 ± 2.1 | 2624 ± 129 |
| **Post-optimization** | 200-L | 27.2 ± 0.8**** | 43.6 ± 0.6** | 2912 ± 137** |
|  | 500-L | 28.2 ± 0.2**** | 46.8 ± 0.3*** | 7797 ± 73**** |

Compared with the 200-L conventional fermentation (temperature 37 °C, pH 7.0, IPTG 1.0 mM, induction time 4 h), 0.001 < ***p* < 0.01, 0.0001 < ****p* < 0.001, *****p* < 0.0001.
