# Supplementary material for: Scaling up production of recombinant human basic fibroblast growth factor in an Escherichia coli BL21(DE3) plysS strain and evaluation of its pro-wound healing efficacy
Source: Front Pharmacol. 2024 Feb 5;14:1279516. doi: 10.3389/fphar.2023.1279516 (PMC10875678; doi:10.3389/fphar.2023.1279516)
Supplement: Supplementary file 10 [file DataSheet12.ZIP › Table/Table 4.docx]

**Table 4** Summary of the purification process for hbFGF (Mean±SD, n = 4)

| **Steps of purification** | **Volume of purification (mL)** | **Total protein (mg)** | **Target protein (mg)** | **SDS-PAGE Purity (%)** | **Recovery (%)** |
| --- | --- | --- | --- | --- | --- |
| Bacteria lysis | 8000 ± 0  (893.6 ± 23.2 g)^a^ | 153,420 ± 28,430.8 | 37,532.5 ± 6097.8 | 24.6 ± 1.8 | / |
| CM-Sepharose | 1422 ±278.3 | 3236.6 ± 97.8 | 2458.4 ± 223.6 | 75.9 ± 5.0 | 2.2 ± 0.4 |
| Heparin affinity | 274.8 ± 23.1 | 2501.7 ± 141.9 | 2279.0 ± 133.4 | 91.2 ± 5.9 | 77.4 ± 5.6 |
| SP-Sepharose | 858.5 ± 88.3 | 2036.3 ± 92.9 | 2013.9 ± 93.6 | 98.9 ± 0.9 | 81.7 ± 7.8 |
| Protein yield (mg/1 L culture): 114.6 ± 5.9 | | | | | |

a: The wet weight of bacteria for a single batch purification process.
