# Supplementary material for: Scaling up production of recombinant human basic fibroblast growth factor in an Escherichia coli BL21(DE3) plysS strain and evaluation of its pro-wound healing efficacy
Source: Front Pharmacol. 2024 Feb 5;14:1279516. doi: 10.3389/fphar.2023.1279516 (PMC10875678; doi:10.3389/fphar.2023.1279516)
Supplement: Supplementary file 10 [file DataSheet12.ZIP › Table/Table 5.docx]

**Table 5.** Comparison of hbFGF expressed for various hosts

| **Host** | **Fermentation Scale** | **Yield** | **Purity** | **Reference** |
| --- | --- | --- | --- | --- |
| *E. coli* DH5α ^a^ | 5 L | / | / | Sheng et al. (1999) |
| *E. coli* JM109 ^a^ | 40 L | 63.7 mg/L | 98.4% | Zhang et al. (2002) |
| *E. coli* DH5α ^a^ | 30 L | / | / | Bai et al. (2002) |
| *E. coli* JM109 ^a^ | 40 L | 97.5 mg/L | / | Feng et al. (2004) |
| *E. coli* JM109 ^a^ | 150 L | / | / | Wang et al. (2007) |
| *E. coli* BL21(DE3) ^a^ | 2 L | 105.3 mg/L | 98% | Chen et al. (2012) |
| *E. coli* BL(DE3)plysS ^a^ | 5 L | 94.8 mg/L | / | Liao et al. (2002) |
| 1. *coli* BL21(DE3) ^b^ | 1 L | 25–35 mg/L | / | Sheng et al. (2003) |
| *E. coli* BL21(DE3) ^c^ | / | 60–80 mg/L | 95% | Imsoonthornruksa et al. (2015) |
| *E. coli* BL21(DE3) ^d^ | / | / | / | Rassouli et al. (2013) |
| *E. coli* BL21(DE3) ^d^ | / | / | / | Soleyman et al. (2016) |
| *E. coli* BL21(DE3) ^e^ | 0.25 L | / | / | Dong et al. (2021) |
| *E. coli* BL21(DE3) ^f^ | 5 L | 1.42 g/L | 96% | Rahman et al. (2020) |
| *Pichia pastoris* ^g^ | / | 91 mg/L | > 94% | Mu et al. (2008) |
| *Pichia pastoris* ^g^ | / | 0.85 mg/L | 98.8% | Le et al. (2020) |
| *Bacillus subtilis* ^a^ | / | 40 mg/L | / | Kwong et al. (2013) |
| *Bacillus subtilis* ^g^ | 2 L | 84 mg/L | / | Hu et al. (2018) |
| *A. thaliana* ^g^ | / | 89.95 ng/mg oil body | / | Yang et al. (2018) |
| Soybean seed ^h^ | / | / | / | Ding et al. (2006) |
| *E. coli* BL(DE3)plysS | 500 L | 114.6 ± 5.9 mg/L | > 99% | Present study |

^a^ hbFGF_155_; ^b^ GST-hbFGF_155_; ^c^ 6His-hbFGF & Trx-6His-hbFGF; ^d^ His-hbFGF_146_; ^e^ Trx-hbFGF_146_; ^f^ Scl2-M-hbFGF;

^g^ hbFGF_146_; ^h^ bbFGF_155_
