# Supplementary material for: Scaling up production of recombinant human basic fibroblast growth factor in an Escherichia coli BL21(DE3) plysS strain and evaluation of its pro-wound healing efficacy
Source: Front Pharmacol. 2024 Feb 5;14:1279516. doi: 10.3389/fphar.2023.1279516 (PMC10875678; doi:10.3389/fphar.2023.1279516)
Supplement: Supplementary file 12 [file DataSheet5.ZIP › Figure 5/Fig 5G.pdf]

报告编号: 202103286

肽段覆盖率分析

上海中科新生命生物科技有限公司

2021-05-14

声　明

1、本分析报告无本公司检验检测专用章和技术负责人签字无效。

2、本分析报告仅对本次所检样品负责，检测结果仅反映对所检样品的评价，对于检测结果的使用、使用所产生的直接或间接损失及一切法律后果，本公司不承担任何经济和法律责任。

3、委托单位对测试结果如有异议并在检测前有留样者，请于分析报告完成之日十五日内向本公司书面提出复检申请，同时附上分析报告原件与并预付复检费。委托单位办理完毕以上手续后，本公司会尽快安排复检。如果复检结果与异议内容相符，本公司将退还委托单位的检测费和复检费，否则委托单位的复检费应照常交纳。不可重复性试验、不能进行复检的，不进行复检，委托单位放弃异议权利。

4、委托单位对样品的代表性和资料的真实性负责，本公司不承担任何相关责任。

5、本公司有权在分析报告发出两周后按客户委托样品处理方式处理剩余样品。

6、未经本公司允许，不得全部或部分复制本分析报告，私自转让、盗用、冒用、涂改或以其他任何形式篡改的均属无效，本公司将对上述行为严究其相应的法律责任。本分析报告仅用于数据参考,不具有对社会的证明作用。

7、除非相关政府部门、法律或法院要求，否则未经本公司书面同意，本公司无须并无义务到法院对相关结果作证。若测试结果被不当使用，本公司将保留撤回测试结果的权利，并有权要求其他适当额外赔偿。

8、如需要在法院审理程序或仲裁过程中使用测试结果，客户必须在向本公司提交测试样品前告知该意图，如果没有告知本公司，出现任何损失、纠纷等等，本公司概不负责，并有权要求其他适当额外赔偿。

9、本公司可以将全部或部分服务委派给代理人或分包方，客户授权给本公司，使本公司有权向代理人或分包方透露相关客户信息，以便更好的完成服务。

10、由于客户自身的错误、疏忽或违约造成服务无法完成或其它损失，本公司将不承担任何责任，并有权要求适当额外赔偿。

11、本公司保证检测的客观公正性，对委托单位的商业信息、技术文件、分析报告等商业秘密履行保密义务。

报告编号: 202103286

重组人碱性成纤维细胞生长因子原液的肽段覆盖率分析

供试品名称: 重组人碱性成纤维细胞生长因子原液

供试品批号: C20201102

委托单位: 温州医科大学

检测人员: 邹倩

核验人员: 周mark[illegible]

技术负责人: 阮宏强

报告编号: 202103286

目 录

1. 供试品信息（客户提供） 3
2. 实验目的 3
3. 实验仪器 3
4. 材料和试剂 4
5. 实验原理和方法 4
5.1实验原理 4
5.2实验方法 5
6. 实验结果和分析 7
7. 结论 9

报告编号: 202103286

1.供试品信息(客户提供)

供试品名称:重组人碱性成纤维细胞生长因子原液

供试品批号:C20201102

供试品状态:液体

理论相对分子质量:16408Da

理论序列:

PALPEDGGSGAFPPGHFKDPKRLYCKNGGFFLRIHPDGRVDGVREKSDPHIKLQLQAEERG

VVSIKGVCANRYLAMKEDGRLLASKCVTDECFFFERLESNNYNTYRSRKYTSWYVALKRT

GQYKLGSKTGPGQKAILFLPMSAKS

2.实验目的

肽段覆盖率是指检测到的肽段的氨基酸数量占该蛋白质总氨基酸数量的比例。蛋白质供试品肽段覆盖率的检测,对于蛋白质类药物的一级氨基酸序列的确证,保证蛋白质类药物的高级结构的形成及维持蛋白质类药物性质都具有很重要的意义。目前对蛋白质肽段覆盖率的检测根据药物申报要求的规定,均采用质谱法进行检测。可以快速,准确,高效的完成肽段覆盖率的检测。本实验是对蛋白质供试品的肽段覆盖率进行分析,采用的实验方法是使用蛋白酶对蛋白质供试品进行酶解,然后使用LC-MS/MS(XevoG2-XS QTof , Waters)对酶解后的肽段样品进行分析。最后使用UNIFI(Waters)软件对LC-MS/MS数据进行分析,根据算法结果确定供试品的肽段覆盖率。

3.实验仪器

报告编号: 202103286

1) 高分辨质谱仪: XevoG2-XS QTof (Waters)

2) 超高效液相色谱:UPLC(Acquity UPLC I-Class) (Waters)

4.材料和试剂

1) Guanidine HCl(Sigma)

2) Urea (Bio-Rad)

3) Tris-base (Bio-Rad)

4) DTT (Bio-Rad)

5) IAM (Sigma)

6) Zeba Spin column (Pierce)

7) ACQUITY UPLC Peptide BEH C18 Column, 300Å, 1.7 μm, 2.1 mm X 150 mm(Waters)

8) UNIFI(Waters)

9) Trypsin(Promega)

10) Chymotrypsin(Sigma)

11) Glu-C(Wako)

12) LysC(Wako)

备注:本实验使用的蛋白酶是根据供试品的理论序列选择的,具体以实验方法中描述为准。

5.实验原理和方法

a)实验原理

报告编号: 202103286

随着生物科技的不断进步,蛋白质药物在新药研发中正在占据越来越大的比重,而蛋白质分子结构的复杂性又要求对蛋白质药物必须进行全面的表征,以满足新药报批、工艺改进和专利保护的要求。目前蛋白质药物的研发和表征还面临很多挑战,尤其是在重组蛋白的序列确证、不同批次间产品的比较和质量控制等方面。质谱在蛋白质的表征方面发挥着至关重要的作用,它不仅可以测定蛋白质药物的分子量和产品的异质性,还可以通过肽图分析确证蛋白质分子的一级结构,包括氨基酸序列、突变和修饰、二硫键定位等信息。蛋白质多肽类药物分子的氨基酸序列分析中包括以下几种实验,N端氨基酸序列分析、C端氨基酸序列分析和肽段覆盖率分析。本文中介绍的方法是基于现代质谱技术,对肽段覆盖率进行分析。使用质谱方法对蛋白质多肽药物分子的肽段覆盖率进行分析的基本原理是将蛋白质分子选用特异性蛋白酶进行切割,酶解后的供试品为复杂的肽段组成的混合物。然后经过液相联用质谱仪进行检测。需要对大分子的蛋白质或多肽进行酶解的原因是现在质谱的分析能力集中在800-4000Da范围内。在这个范围内,质谱仪能够有效的分析多肽分子的分子量,并将其破碎后成为MS2。只有经过酶解后的供试品的肽段范围在检测能力之内,供试品中所有的母离子及其碎片离子信息才能被记录下来。检测的结果文件再经过与理论序列的比对,就可确定该序列中的氨基酸组成。因此要进行肽段覆盖率的检测需要提供蛋白质多肽的理论序列,以便用来查库。在大多数的酶解实验中,常用的蛋白质内切酶有胰蛋白酶、胰凝乳蛋白酶、Arg-C酶、Lys-C酶和Glu-C酶等等。不同的蛋白质内切酶作用于不同的氨基酸残基末端,形成不同长度的多肽分子。因此对于肽段覆盖率的检测一般会采用多种酶解联合使用的策略,以提高质谱仪对肽段覆盖率的检测。

b)实验方法

1) Trypsin、Chymotrypsin、Glu-C酶解:取适量供试品经适当前处理后加入Trypsin、Chymotrypsin、Glu-C, 37°C酶切20小时。

报告编号: 202103286

2) 高效液相色谱:供试品经酶解处理后采超高效液相系统Acquity UPLC I-Class进行分离。液相A液为0.1%FA水溶液,B液为0.1%FA乙腈溶液。供试品由自动进样器上样到Column,再经色谱柱分离,柱温为55°C,流速为300μl/min,TUV检测器波长为214nm。相关液相梯度如下:

	Time/min	A/%	B/%
1	3	98	2
2	63	60	40
3	63. 1	2	98
4	66	2	98
5	66. 1	98	2
6	75	98	2

3) 质谱鉴定:供试品经超高效液相色谱脱盐及分离后用XevoG2-XS QTof质谱仪(Waters)进行质谱检测分析。分析时长:63min,检测方式:正离子,MS^E,扫描范围(m/z):300-2000。

4) 质谱数据处理:原始使用UNIFI(1.8.2,Waters)软件查库,主要参数如(表1):

Item	Value
Enzyme	Trypsin、Chymotrypsin、Glu-C
modifications	Carbamidomethyl(C), Deamidated(NQ), Oxidation(M)
M/Z tolerance	±15ppm
Fragment tolerance	±20ppm
Database	20210402916.fasta

报告编号: 202103286

Filter	Minimum fragment ions:3

6.实验结果和分析

在使用Trypsin、Chymotrypsin、Glu-C分别对供试品进行溶液内酶解后,得到的肽段样品,经过LC-MS/MS设备的分析,得到的原始数据经过UNIFI软件进行查库,所使用的数据库为客户提供的供试品理论序列。

1) 供试品的酶解后的BPI图谱见下图。

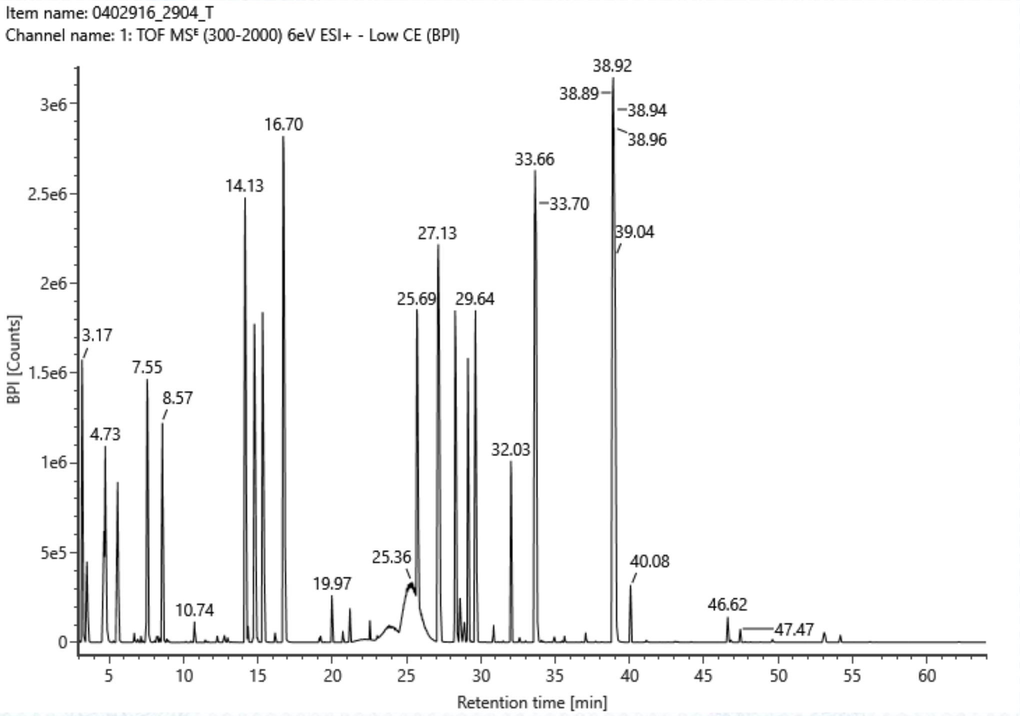

图1供试品Trypsin酶解后的BPI图谱

报告编号: 202103286

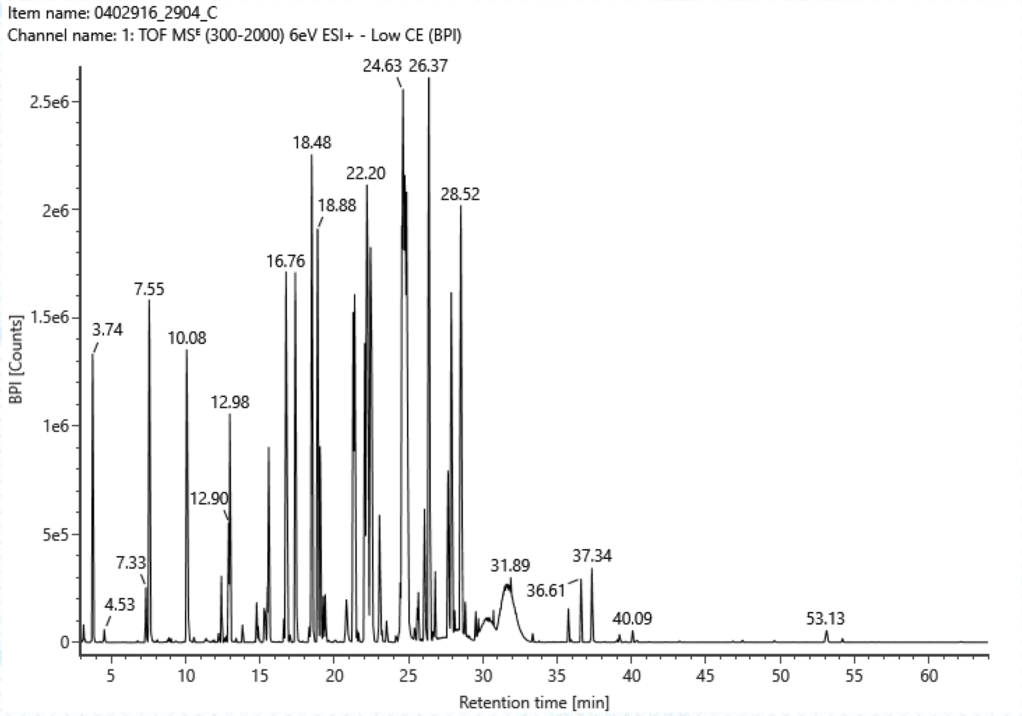

图2供试品Chymotrypsin酶解后的BPI图谱

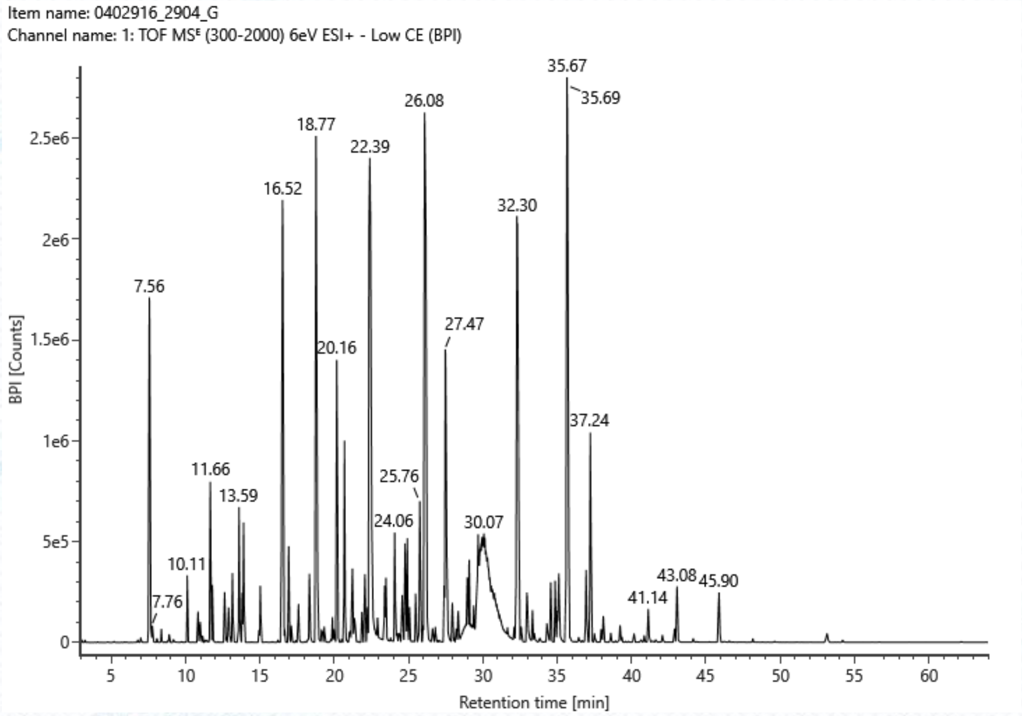

图3供试品Glu-C酶解后的BPI图谱

2) Trypsin、Chymotrypsin、Glu-C酶解覆盖率分别见下图

报告编号: 202103286

PALPEDGGSG	AFPPGHFKDP	KRLYCKNGGF	FLRIHPDGRV	DGVREKSDPH	IKLQLQAEER	GVVSIKGVCA	NRYLAMKEDG
RLLASKCVTD	ECFFFERLES	NNYNTYRSRK	YTSWYVALKR	TGQYKLGSKT	GPGQKAILFL	PMSAKS	

图4供试品的Trypsin酶解后的覆盖率

PALPEDGGSG	AFPPGHFKDP	KRLYCKNGGF	FLRIHPDGRV	DGVREKSDPH	IKLQLQAEER	GVVSIKGVCA	NRYLAMKEDG
RLLASKCVTD	ECFFFERLES	NNYNTYRSRK	YTSWYVALKR	TGQYKLGSKT	GPGQKAILFL	PMSAKS	

图5供试品的Chymotrypsin酶解后的覆盖率

PALPEDGGSG	AFPPGHFKDP	KRLYCKNGGF	FLRIHPDGRV	DGVREKSDPH	IKLQLQAEER	GVVSIKGVCA	NRYLAMKEDG
RLLASKCVTD	ECFFFERLES	NNYNTYRSRK	YTSWYVALKR	TGQYKLGSKT	GPGQKAILFL	PMSAKS	

图6供试品的Glu-C酶解后的覆盖率

酶解后的样品经过LC-MS/MS分析后查库结果进行整合,最终得到该供试品的肽段覆盖率为100%(146/146)。

7.结论

综上所述,供试品重组人碱性成纤维细胞生长因子原液(批号:C20201102)的肽段覆盖率为:100%(146/146)。
