# Supplementary material for: Scaling up production of recombinant human basic fibroblast growth factor in an Escherichia coli BL21(DE3) plysS strain and evaluation of its pro-wound healing efficacy
Source: Front Pharmacol. 2024 Feb 5;14:1279516. doi: 10.3389/fphar.2023.1279516 (PMC10875678; doi:10.3389/fphar.2023.1279516)
Supplement: Supplementary file 12 [file DataSheet5.ZIP › Figure 5/Fig 5H.pdf]

报告编号: 202103292

圆二色谱紫外扫描分析(合并)

上海中科新生命生物科技有限公司

2021-05-12

声　　明

1、本分析报告无本公司检验检测专用章和技术负责人签字无效。

2、本分析报告仅对本次所检样品负责，检测结果仅反映对所检样品的评价，对于检测结果的使用、使用所产生的直接或间接损失及一切法律后果，本公司不承担任何经济和法律责任。

3、委托单位对测试结果如有异议并在检测前有留样者，请于分析报告完成之日十五日内向本公司书面提出复检申请，同时附上分析报告原件与并预付复检费。委托单位办理完毕以上手续后，本公司会尽快安排复检。如果复检结果与异议内容相符，本公司将退还委托单位的检测费和复检费，否则委托单位的复检费应照常交纳。不可重复性试验、不能进行复检的，不进行复检，委托单位放弃异议权利。

4、委托单位对样品的代表性和资料的真实性负责，本公司不承担任何相关责任。

5、本公司有权在分析报告发出两周后按客户委托样品处理方式处理剩余样品。

6、未经本公司允许，不得全部或部分复制本分析报告，私自转让、盗用、冒用、涂改或以其他任何形式篡改的均属无效，本公司将对上述行为严究其相应的法律责任。本分析报告仅用于数据参考，不具有对社会的证明作用。

7、除非相关政府部门、法律或法院要求，否则未经本公司书面同意，本公司无须并无义务到法院对相关结果作证。若测试结果被不当使用，本公司将保留撤回测试结果的权利，并有权要求其他适当额外赔偿。

8、如需要在法院审理程序或仲裁过程中使用测试结果，客户必须在向本公司提交测试样品前告知该意图，如果没有告知本公司，出现任何损失、纠纷等等，本公司概不负责，并有权要求其他适当额外赔偿。

9、本公司可以将全部或部分服务委派给代理人或分包方，客户授权给本公司，使本公司有权向代理人或分包方透露相关客户信息，以便更好的完成服务。

10、由于客户自身的错误、疏忽或违约造成服务无法完成或其它损失，本公司将不承担任何责任，并有权要求适当额外赔偿。

11、本公司保证检测的客观公正性，对委托单位的商业信息、技术文件、分析报告等商业秘密履行保密义务。

报告编号: 202103292

重组人碱性成纤维细胞生长因子原液的圆二色谱紫外扫描分析(合并)

供试品名称: 重组人碱性成纤维细胞生长因子原液

供试品批号: C20201102、C20201201、C20201202

委托单位: 温州医科大学

检测人员: 廖邓雷

核验人员: 易路

技术负责人: 阮宏强

报告编号: 202103292

目 录

1. 供试品信息（客户提供）........ 3
2. 实验目的........ 3
3. 实验仪器........ 3
4. 材料和试剂........ 3
5. 实验原理和方法........ 4
5.1实验原理........ 4
5.2实验方法........ 4
6. 实验结果和分析........ 5
6.1标准品的远近紫外图谱分析........ 5
6.2供试品图谱分析........ 6
7. 结论........ 12

报告编号: 202103292

1.供试品信息(客户提供)

样品编号	样品名称	样品批号
20210402913	重组人碱性成纤维细胞生长因子原液	C20201102
20210402914	重组人碱性成纤维细胞生长因子原液	C20201201
20210402915	重组人碱性成纤维细胞生长因子原液	C20201202

2.实验目的

蛋白质、多肽的圆二色谱扫描分析对于研究它们的二级结构和高级结构非常有意义。研究蛋白质、多肽的二级及高级结构有几种方法,如X-ray晶体衍射技术、核磁共振技术和圆二色谱技术等。其中,前两种方法较复杂,并且受到很多因素的影响,分析起来比较困难,但是能够采集到足够且有效的数据能对蛋白质、多肽的空间结构解析得比较清楚。相对而言,圆二色谱技术是一种比较简单且有效的技术,为基因工程表达的蛋白质类产品的高级结构确认提供了便捷的手段,是研究稀溶液中蛋白质构象的一种快速、简单、较准确的方法。可以在溶液状态下测定,较接近其生理状态。而且测定方法快速简便,对构象变化灵敏,是目前研究蛋白质二级结构的主要手段之一,并广泛应用于蛋白质的构象研究中。本实验的目的是通过采集蛋白质供试品和对照品在远紫外(190-260nm)和近紫外(250-340nm)的圆二色(CD)吸收图谱并通过软件对二级结构及一致性进行分析。

3.实验仪器

1) Chirascan Plus V100圆二色光谱仪(英国应用光物理)

2) 比色皿(Hellma)

报告编号: 202103292

4.材料和试剂

1) CSA(Sigma)

2) PB磷酸盐缓冲液(北京绿源伯德)

3) 硝酸(国药)

5.实验原理和方法

a)实验原理

一束入射的平面偏振光可以看做由两束振幅相同相位相反的左、右偏振光组成的偏振光,当其通过光学活性分子后,光学活性分子对左、右圆偏振光的吸收也不同,引起左、右圆偏振光振幅的变化,使左、右圆偏振光透过后组成了椭圆偏振光,振动平面偏离入射振动平面,形成一定夹角,这种现象称为圆二色性。圆二色谱扫描就是利用蛋白质的圆二色性及不对称分子对左右圆偏振光吸收的不同来进行结构分析。蛋白质是由氨基酸通过肽键连接而成的具有特定结构的生物大分子。在蛋白质或多肽中主要的光活性基团是肽链骨架中的肽键、芳香氨基酸残基及二硫键等。当平面圆偏振光的吸收不相同时,产生吸收差值。由于这种吸收差的存在,造成了偏振光矢量的振幅差,圆偏振光变成了椭圆偏振光,这就是蛋白质的圆二色性。

b)实验方法

1)仪器参数设定

Band width:	1.0nm
Step:	1.0nm
Measurement range:	190-260nm(far-UV region scan)/250-340nm(near-UV region scan)
Time-per-point:	0.5s

报告编号: 202103292

Repeats:	3
Cell Length:	0.5mm/10mm
Temperature:	Room Temperature

2)标准品远近紫外扫描

设定扫描波长180-340nm进行背景测试、空白buffer测试,然后采集1mg/mL CSA 标准品溶液在180-340nm范围的圆二色远近紫外吸收。

3)远近紫外

样品远紫外扫描:将比色皿用2M HNO3浸泡过夜,去离子水冲洗干净后晾干,先采集背景,再采集空白缓冲液,然后在比色皿中加入供试品按照上述参数进行190-260nm的远紫外扫描并采集数据。

样品近紫外扫描:将比色皿用2M HNO3浸泡过夜,去离子水冲洗干净后晾干,先采集背景,再采集空白缓冲液,然后在比色皿中加入供试品按照上述参数进行250-340nm的近紫外扫描并采集数据。

4)扫描图谱处理

对扫描后的所有图谱用软件Pro-Data Viewer进行subtract baseline、smoothing处理。

对标准品波峰波谷CD值的比值进行计算,有效比值范围为2.08±0.06。

6.实验结果和分析

1)标准品的远近紫外图谱分析

此次标准品图谱波峰波谷CD值的比值为2.10,标准品的远近紫外CD图谱如下:

报告编号: 202103292

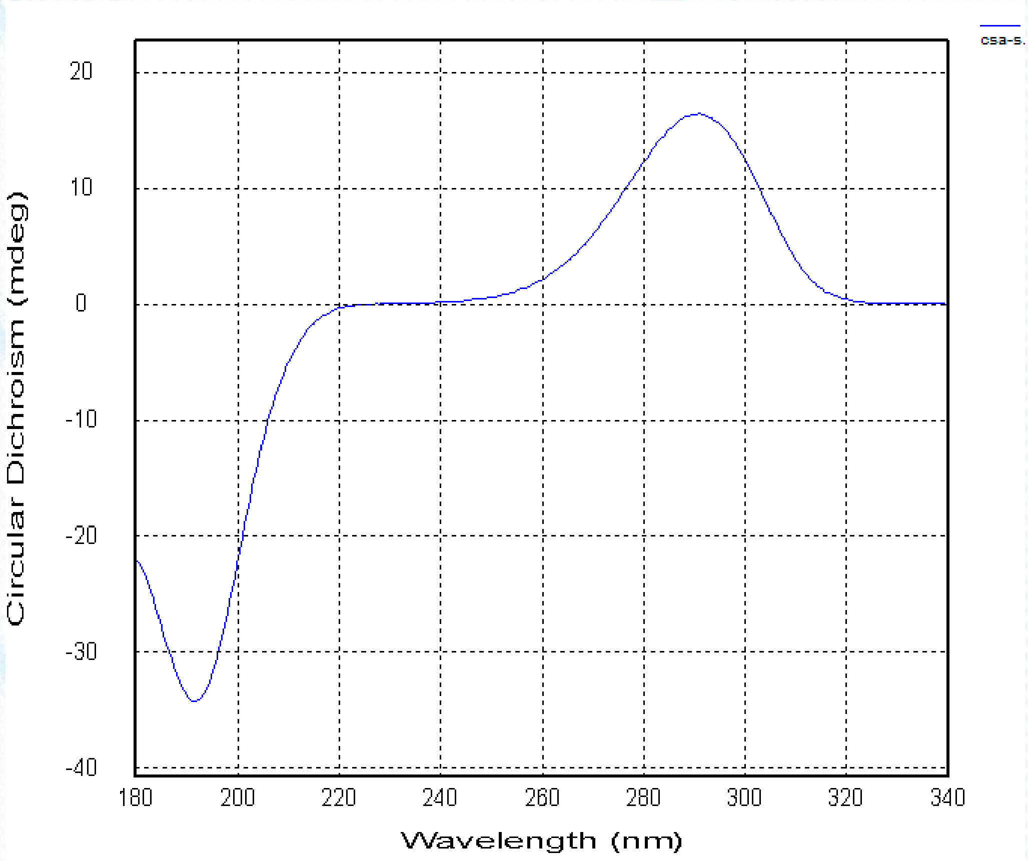

图1标准品的远近紫外CD图谱

2)供试品图谱分析

供试品远紫外CD扫描图谱和overlay图谱如下图:

报告编号: 202103292

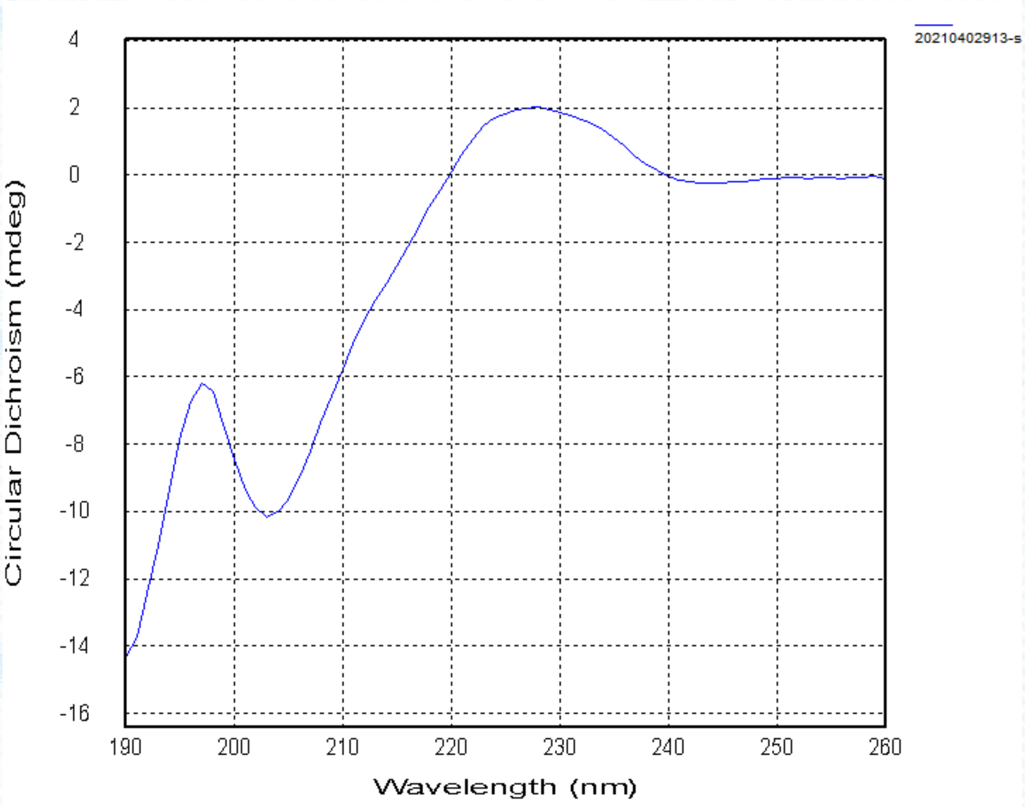

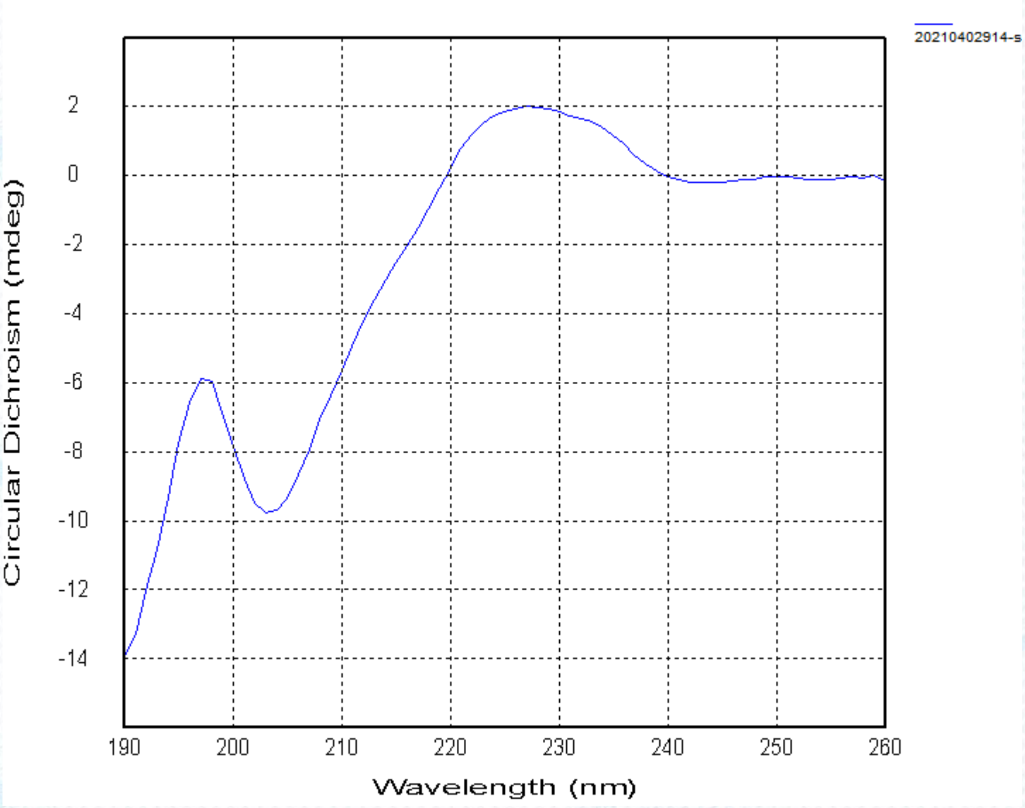

报告编号: 202103292

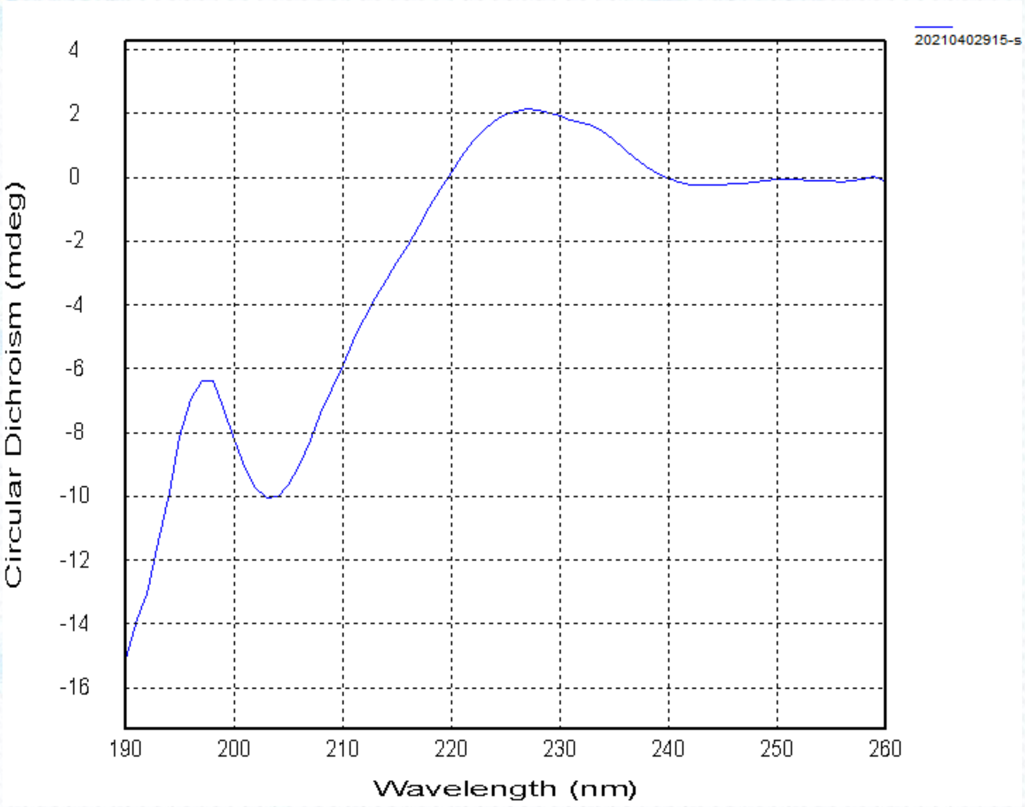

图2供试品远紫外CD图谱

报告编号: 202103292

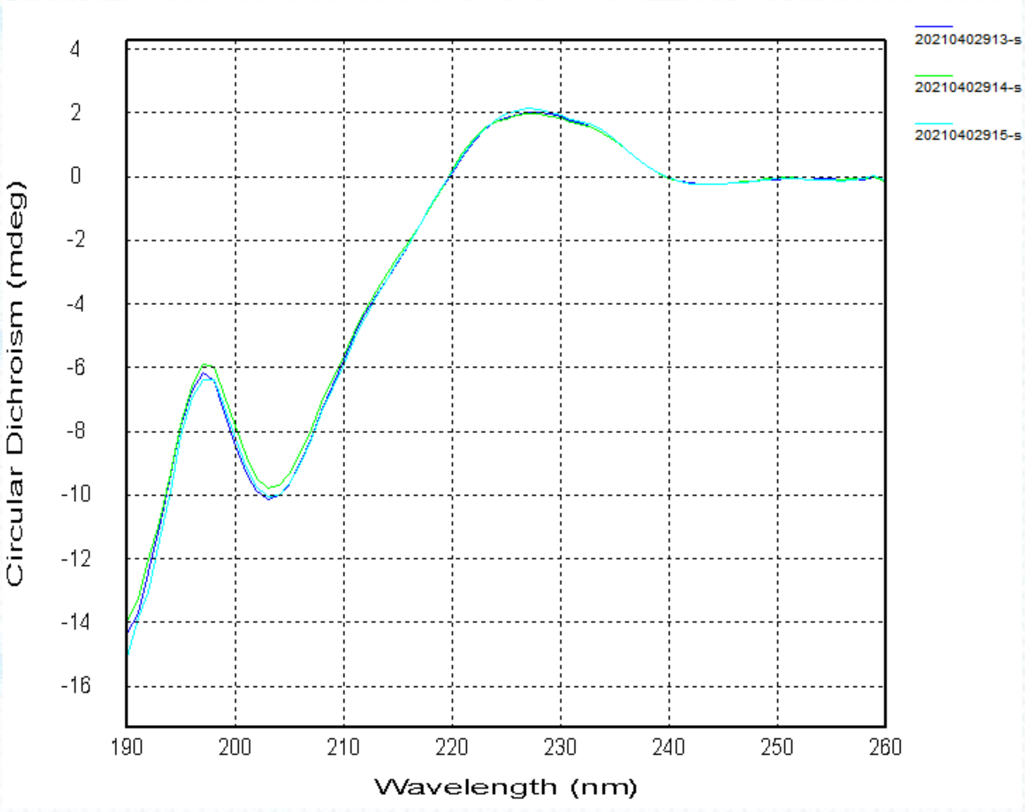

图3供试品的远紫外CD扫描overlay图谱

供试品的二级结构预测圆二色谱在远紫外区的扫描图谱,反映的是蛋白质肽键的排布信息,计算所得的是蛋白质二级结构比例,即螺旋(Helix)、折叠(Antiparallel+Parallel)、转角(Beta-turn)和不规则卷曲(Random coil)的比例。目前用于拆分和估算的软件不下十几种,得到研究者广泛认同的计算模式有几种,但由于不同的计算模式的理论基础不同,所计算得到的蛋白质二级结构比例并不完全相同,但多数软件对α-螺旋的估计还是比较准确的。我们采用CDNN软件对供试品二级结构进行拟合计算,在Milli-Degress模式下分别计算出不同波长区间螺旋(Helix)、折叠(Antiparallel+Parallel)、转角(Beta-turn)和不规则卷曲(Random coil)的比例。如使用q-Bic软件进行供试品与参比品的一致性分析时,分析结果以similarity(供试品与参比品的相似度)表示。由于本预测结果可能存在误差,仅供参考之用。供试品二级结构测算值分析图表如下。

报告编号: 202103292

	Helix	Antiparallel	Parallel	Beta-Turn	Rndm.coil
190-260nm	4.3%	14.2%	2.0%	27.4%	47.1%

20210402913 (重组人碱性成纤维细胞生长因子原液，C20201102)

	Helix	Antiparallel	Parallel	Beta-Turn	Rndm.coil
190-260nm	4.3%	15.1%	2.0%	26.8%	46.4%

20210402914 (重组人碱性成纤维细胞生长因子原液，C20201201)

	Helix	Antiparallel	Parallel	Beta-Turn	Rndm.coil
190-260nm	4.2%	13.6%	2.0%	27.4%	47.9%

20210402915 (重组人碱性成纤维细胞生长因子原液，C20201202)

图4供试品的二级结构测算值分析图表

供试品近紫外CD扫描图谱和overlay图谱如下图:

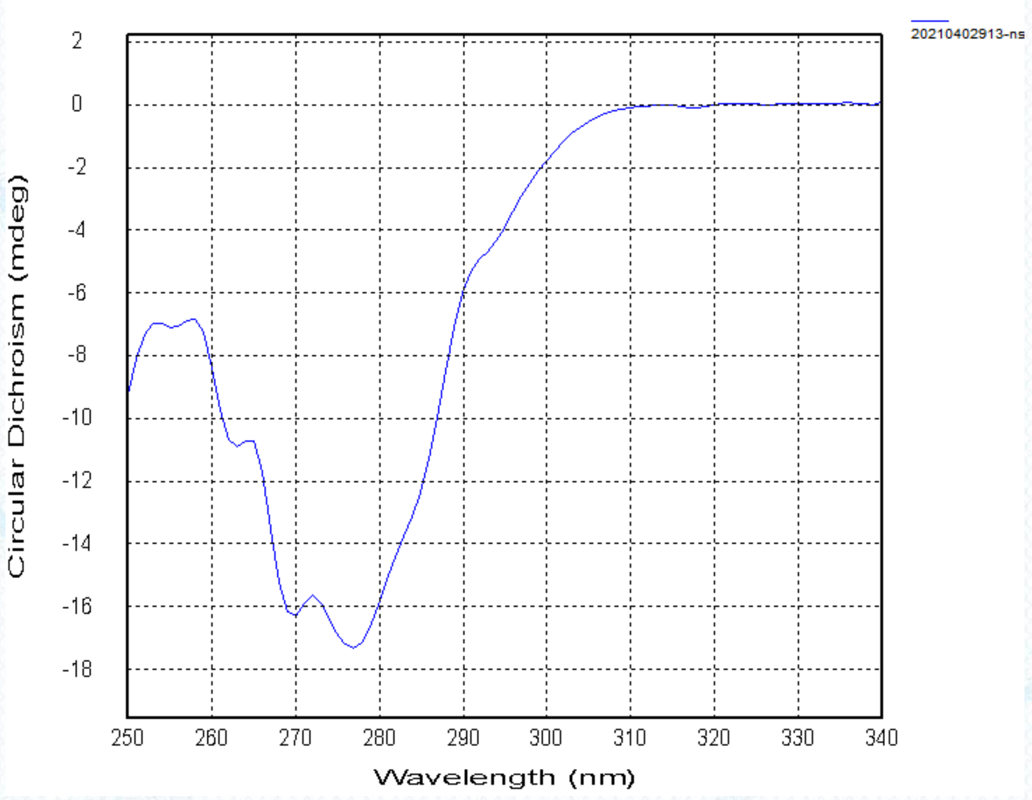

报告编号: 202103292

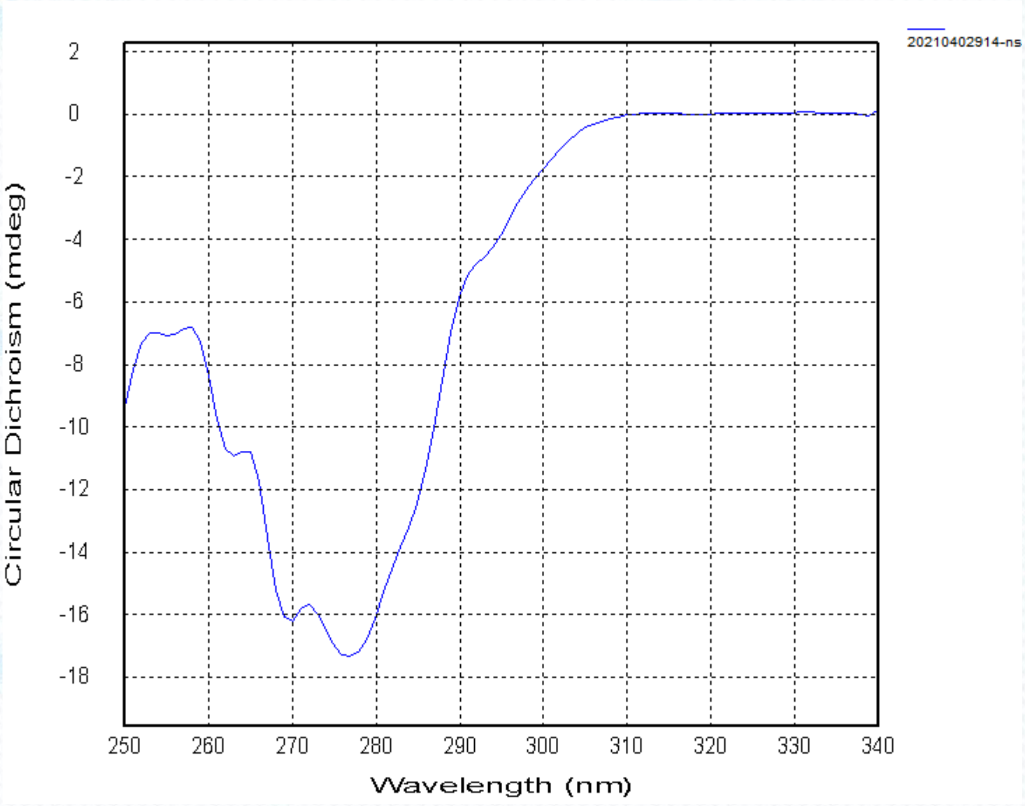

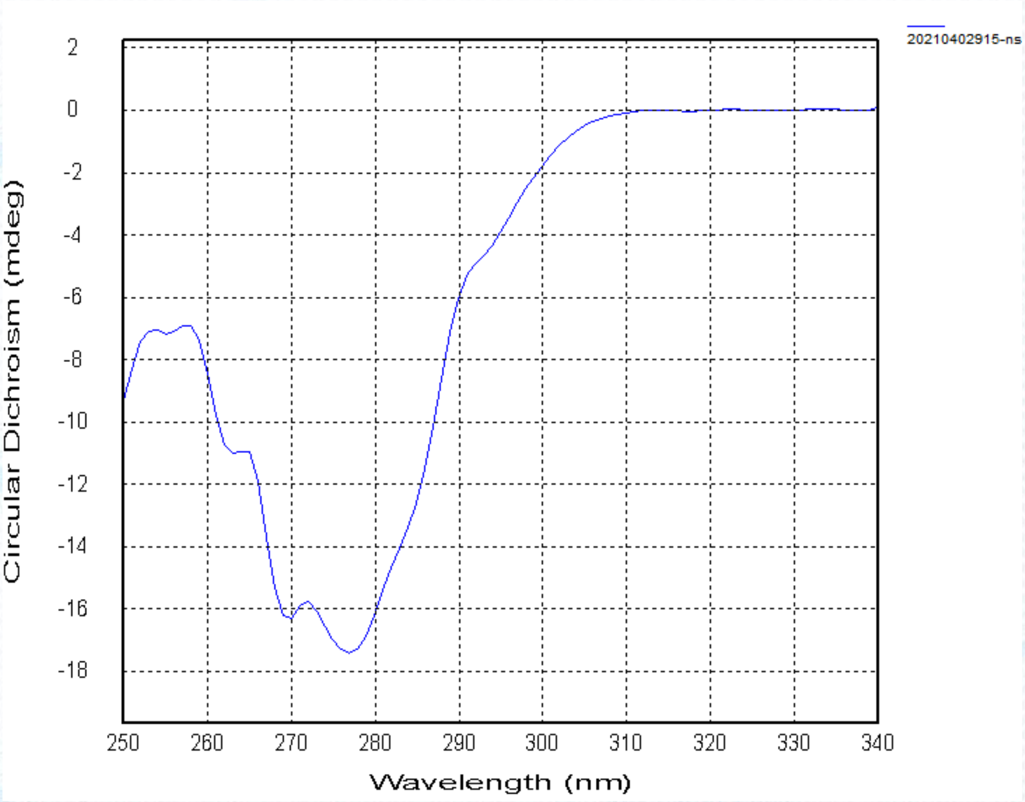

图5供试品近紫外CD图谱

报告编号: 202103292

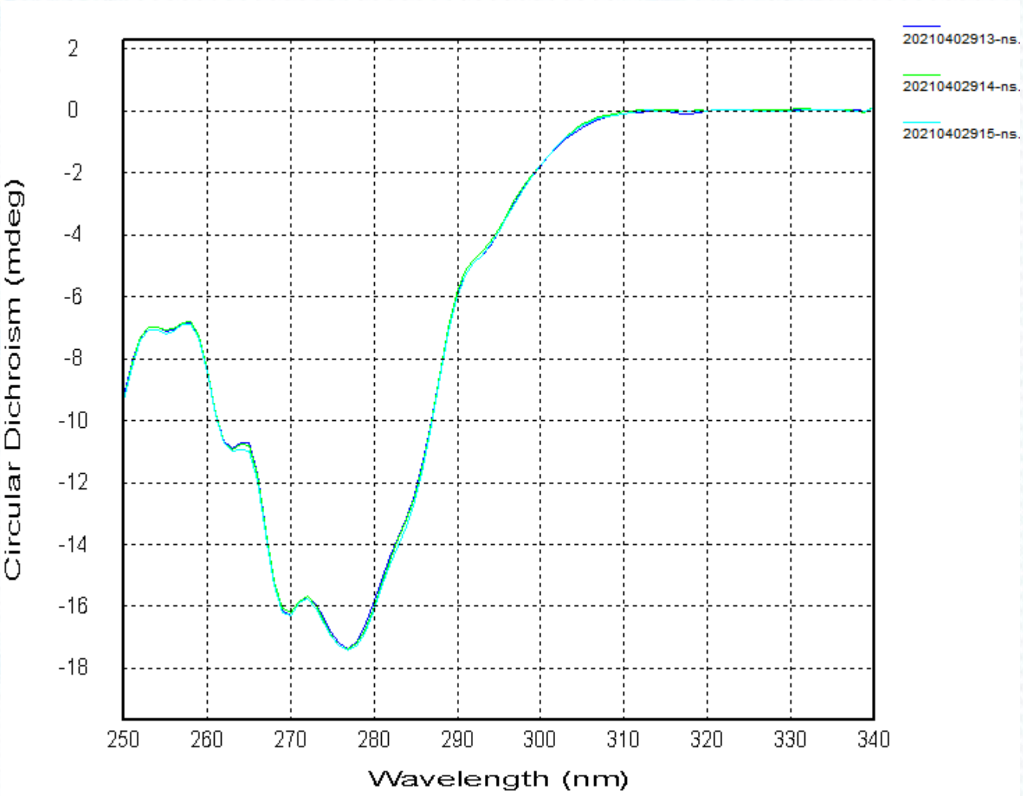

图6供试品的近紫外CD扫描overlay图谱

7.结论

供试品重组人碱性成纤维细胞生长因子原液(C20201102、C20201201、C20201202)在远紫外区和近紫外区的圆二色谱图,Overlay处理后一致性图谱如上图所示。
